# Modular Enantioselective Total Syntheses of the *erythro*-7,9-Dihydroxy- and 9-Hydroxy-7-Keto-8,4′-Oxyneolignans

**DOI:** 10.1021/acs.joc.4c00710

**Published:** 2024-07-03

**Authors:** Meghan C. Benda, Caria Evans, Shaoren Yuan, Ian M. McClish, William J. Berkey, Hailey E. Areheart, Emily S. Arnold, Michelle L. Tang, Stefan France

**Affiliations:** † School of Chemistry and Biochemistry, Georgia Institute of Technology, Atlanta, Georgia 30332, United States; ‡ School of Biological Sciences, Georgia Institute of Technology, Atlanta, Georgia 30332, United States; § Renewable Bioproducts Institute, Georgia Institute of Technology, Atlanta, Georgia 30332, United States; ∥ Center for a Renewables-based Economy from WOOD (ReWOOD), Georgia Institute of Technology, Atlanta, Georgia 30332, United States

## Abstract

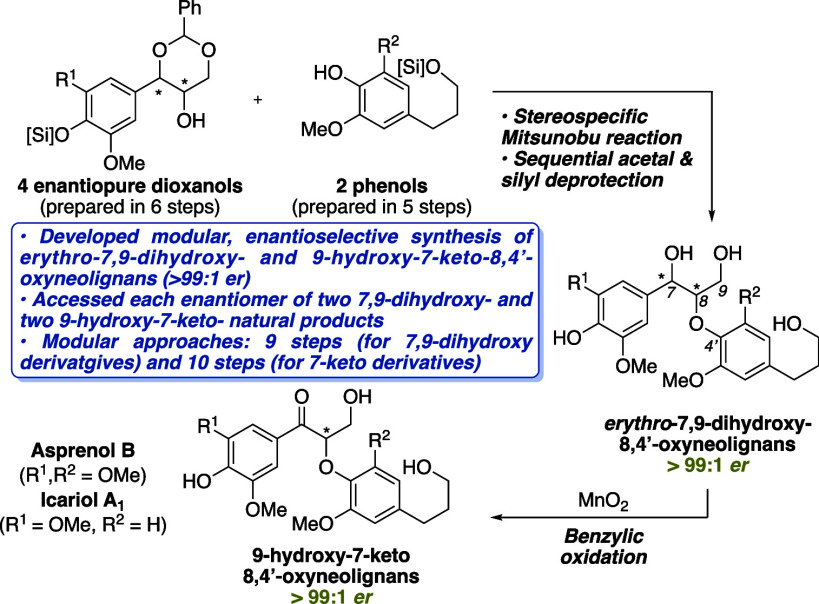

A modular, enantioselective approach to access the bioactive 7,9-dihydroxy- and 9-hydroxy-7-keto-8,4′-oxyneolignans is disclosed, which employs stereoselective Mitsunobu reactions of enantiopure 2-aryl-1,3-dioxan-5-ols and functionalized phenols. The enantiopure dioxanols are prepared through Sharpless asymmetric dihydroxylation of protected coniferyl or sinapyl alcohols and subsequent benzylidene acetal formation. Through a mix-and-match coupling approach, six of the eight possible *erythro*-7,9-dihydroxy-8,4′-oxyneolignan enantiomeric natural products (bearing a C-1′ hydroxypropyl chain) were generated following sequential deprotection. Subsequent benzylic oxidation afforded the 7-keto-derivatives, resulting in enantioselective syntheses of each enantiomer of the natural products asprenol B and icariol A_1_.

## Introduction

**Figure 1:**
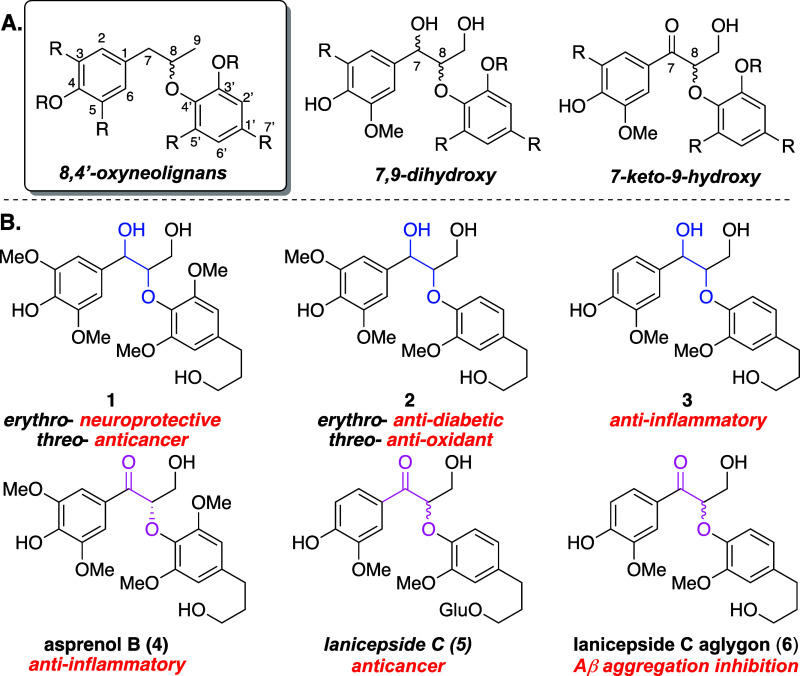
8,4′-Oxyneolignan subclasses (A) and representative bioactive derivatives (B).

Over the past several decades, the 8,4′-oxyneolignans have been heavily featured in natural product isolation literature as they constitute a major group of secondary plant metabolites^1^ that can be found in nature existing as both enantiomers.^2^ They are characterized by two phenylpropanoid units bound by a C^8^–O–C^4^′ aryl ether linkage and have been shown to possess anti-inflammatory,^3^ antioxidant,^4^ anticancer,^5^ and neuroprotective^6^ properties among other biologically relevant functions (Figure 1). Representative examples of the 7,9-dihydroxy-8,4′-oxyneolignans include: oxyneolignan **1**, whose *erythro* and *threo* diastereomers (isolated from the fruits of *Crataegus pinnatifida* Bge) demonstrated promising neuroprotective and anticancer activity, respectively;^7^ oxyneolignan **2**, whose *erythro* diastereomer (isolated from *Livistona chinensis*)^8^ showed antioxidant activity as compared to antidiabetic activity shown by its *threo* isomer (isolated from the roots of *Alangium chinense*);^9^ and neolignan **3**, which displayed weak anti-inflammatory activity for both diastereomers (isolated from *P. sylvestris* L. and *L. chinensis*).^10^ For the 7-keto-9-hydroxy-8,4′-neolignans, representative examples include: asprenol B **4** (isolated from the stems of *Ilex asprella* as part of the fractions that displayed anti-inflammatory potential);^12^ lanicepside C **5** (isolated as the (*S*)-enantiomer from *Clematis chinensis*),^13^ which showed modest cytotoxic activities against three different human cancer cell lines; and the lanicepside C aglycon **6** (isolated from the seeds of *P. tormentosa* as the racemate)^6b^ whose separated enantiomers each demonstrated more potent inhibitory activity on Aβ aggregation than curcumin, the positive control.

Given their interesting bioactive properties, 8,4′-oxyneolignans have been frequent subjects in total synthesis literature.^14^ The vast majority of syntheses specifically target oxyneolignans containing hydroxyl groups at just the 7-position or at both the 7- and 9-positions, whereas the 9-hydroxy-7-keto subclass of 8,4′-oxyneolignans has been rather understudied. To achieve asymmetric syntheses, the use of chiral auxiliaries to ensure enantioselective aldol addition has become standard.^15^ This is particularly true for the 7,9-dihydroxy cogeners. It is possible to avoid the use of these chiral auxiliaries through the use of either Sharpless asymmetric epoxidation^16,17^ (SAE) or Sharpless asymmetric dihydroxylation^18,19^ (SAD). These alternatives, unfortunately, also have their own drawbacks. In the case of the SAE route, racemic secondary allylic alcohols were used to generate enantiomerically enriched epoxides and unreacted enantiomerically pure alcohols. As expected in a formal resolution process, the highest possible yield is 50% from a racemic mixture. As for the route utilizing SAD,^19b^ there has been no formal verification of enantiomeric purity of the final targets. Optical rotation was employed, but no comparisons against the natural product isolation characterization were discussed.^19^

There have been two reports that access protected 7-keto-9-hydroxy-8,4′-oxyneolignans en route to their 7-hydroxy-8,4′-oxyneolignan counterparts.^15d,20^ Unfortunately, neither of these pathways enantioselectively forms the β-aryl ether linkage. Katayama^20^ and co-workers featured two sequential aldol addition reactions to form the racemic 7-keto intermediates, whereas Banwell^15d^ and co-workers installed the desired β-aryl ether linkage via SAD but observed epimerization following base-mediated S_N_2 C–O bond forming conditions. Furthermore, Hishiyama^21^ and co-workers employed a kinetic resolution to form products that feature both the 7-keto and 9-hydroxy functional groups but lack the C-1′ substituent that is essential for the compounds to be characterized as lignans. Thus, scarce literature exists on these compounds being successfully targeted in an enantioselective fashion as compared to access through resolution. This provides an ideal opportunity for the development of an enantioselective approach to both of these classes of oxyneolignans.

**Figure 2:**
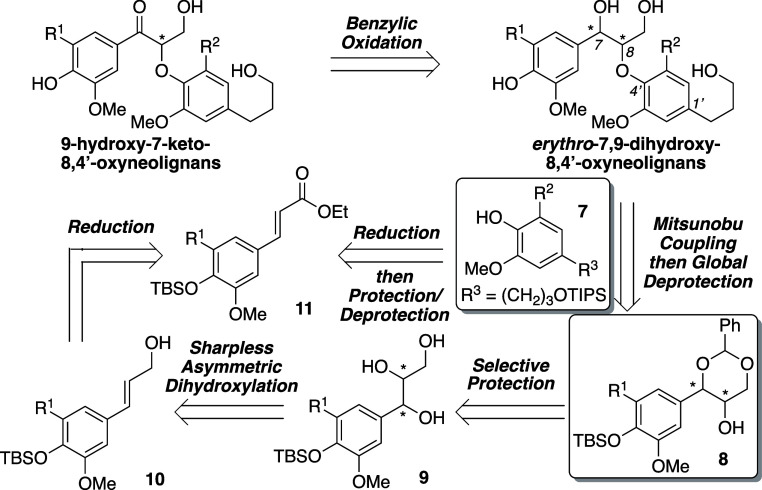
Retrosynthetic analysis for the *erythro*-7,9-dihydroxy- and 9-hydroxy-7-keto-8,4′-oxyneolignans.

We envisioned using a “mix-and-match” Mitsunobu^22^ coupling strategy that could serve as the template reaction to form the 8-*O*-4′ linkages that are key to the 7,9-dihydroxy-8,4′-oxyneolignans, followed by benzylic oxidation to access the structurally related 7-keto-8,4′-oxyneolignans. The proposed approach enables the exquisite control of three key features of the oxyneolignan architecture: (1) the absolute configuration of the 8-*O*-4′-aryl ether linkage; (2) the number of methoxy substituents (or other desired substituents) on each aryl ring; and (3) the composition of the C-1′ substituent (Figure 2). Thus, we disclose a modular enantioselective approach to several naturally occurring 7,9-dihydroxy- and 7-keto-9-hydroxy-8,4′-oxyneolignans.

## Results and Discussion

**Scheme 1:**
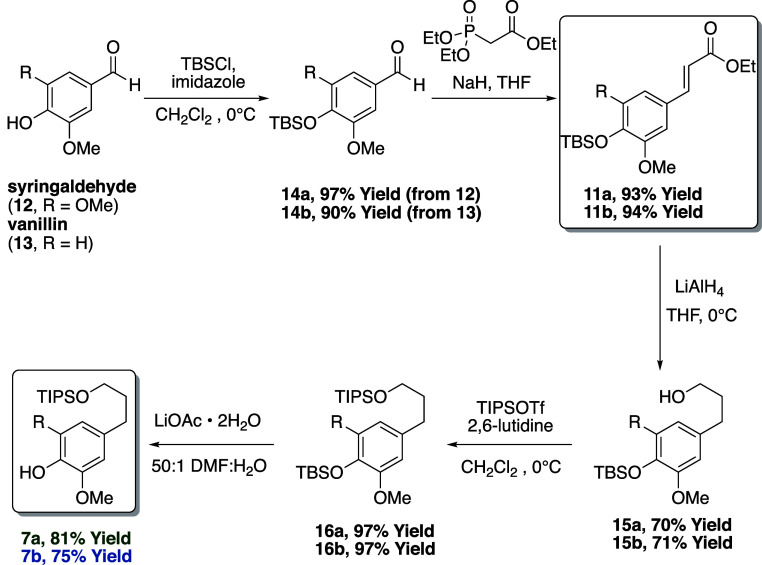
Preparation of the Mitsunobu Nucleophiles **7**

To create the core scaffold of the 7,9-dihydroxy- and 9-hydroxy-7-keto-8,4′-oxyneolignans through Mitsunobu coupling, we first needed to synthesize both the functionalized phenols **7** and their Mitsunobu coupling partners **8**. Given that both coupling partners contain aryl moieties with either one or two methoxy groups, we outlined a divergent synthetic approach to each related component from one of two α,β-unsaturated ester precursors **11** (Scheme 1). Synthesis of **11** was achieved through silyl protection of either commercial syringaldehyde (**12**) or vanillin (**13**)^23^ followed by a Horner–Wadsworth–Emmons^24^ olefination.^25^ It is important to note that enoates **11** can also be prepared from commercial sinapic or ferulic acid,^19b^ albeit for a higher cost and no reduction in step count. Reduction of enoates **11** with LiAlH_4_ readily generates arylpropanols **15a** and **15b** in 70 and 71% yield, respectively. Sequential primary alcohol protection using triisopropylsilyl chloride followed by selective *tert*-butyldimethylsilyl aryl ether deprotection provided substituted phenols **7** as Mitsunobu nucleophiles.

**Scheme 2:**
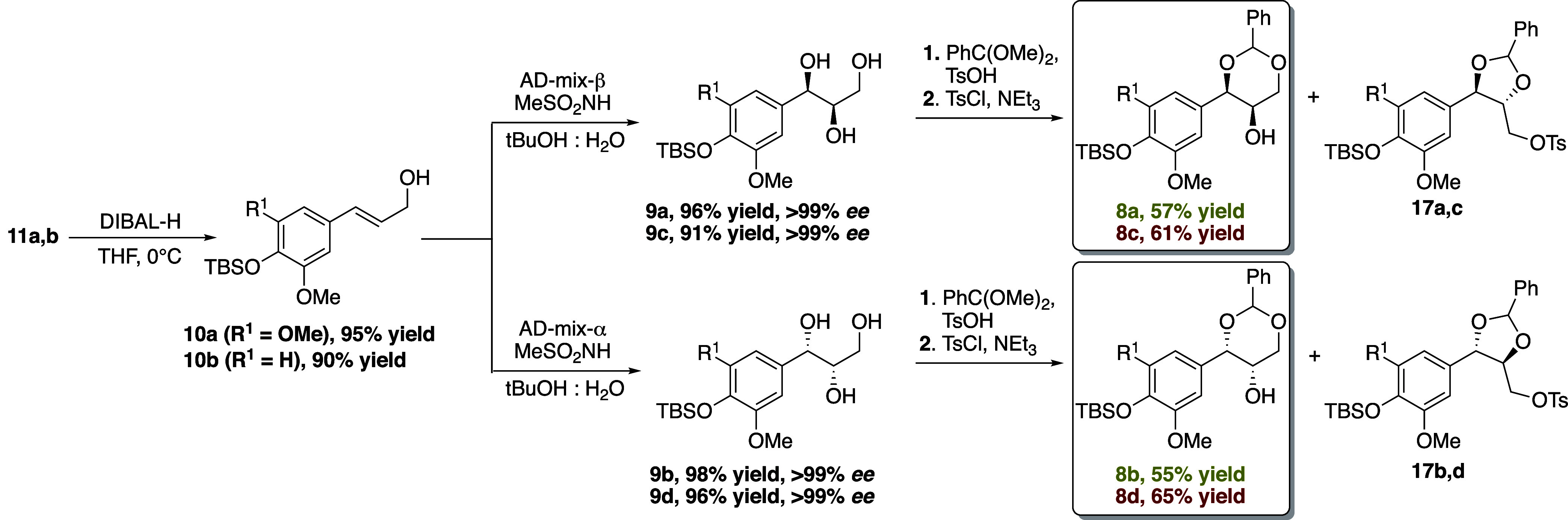
Sharpless Asymmetric Dihydroxylations and Preparation of the Enantiopure Dioxanols **8**

Next, we sought to prepare functional Mitsunobu electrophiles **8** (Scheme 2). Enoates **11a** and **11b** were, respectively, reduced to the corresponding allylic alcohols **10a** and **10b** in 95 and 90% yield using DIBAL-H (Scheme 2). Our initial approach employed Sharpless asymmetric epoxidation of **10** followed by a variety of ring-opening functionalization proposals.^26^ While these efforts failed to provide a viable pathway to the desired oxyneolignans, they highlighted the following critical observations: (1) Mitsunou reaction performance plummeted as the sterics around the coupling site and the bulkiness of the nucleophile increased; (2) when dealing with α-hydroxy ketones as the Mitsunobu electrophiles, epimerization presented a major issue during coupling; and (3) benzylic methylene oxidation gave undesired side reactions and degradation.

Thus, it became obvious that an updated synthetic design should include both a less hindered site of coupling and an oxidizable functional group at C-7. To this end, we employed the Sharpless asymmetric dihydroxylation of allylic alcohols **10** with AD-mix-β to furnish enantiopure (7*R*,8*R*)-triols **9a** and **9c** in >96% yield and >99% ee.^19b^ Similarly, the (7*S*,8*S*)-enantiomers **9b** and **9d** were readily accessible in high yields and enantiomeric excesses (ee’s) using AD-mix-α. In order to transform compounds **9** into the appropriate Mitsunobu electrophiles, we became inspired by Pan^27^ and co-workers who disclosed a general enantioselective approach to the 8-*O*-4′ linkage of neolignans using an enantiopure 2,4-diaryl dioxan-5-ol as the Mitsunobu partner. Embedded in the dioxanol framework was a benzylidene acetal^28^ protecting group (for the 7- and 9-hydroxyl functionalities) that afforded both a less hindered coupling site for nucleophilic substitution and access to a hydroxyl group at C-7 upon deprotection. Subsequent treatment of triols **9** with benzaldehyde dimethyl acetal and pTsOH provided inseparable mixtures of dioxanols **8** and dioxolanes **17**. Selective tosylation^27^ of the dioxolanes enabled chromatographic separation of the mixture and isolation of enantiopure dioxanols **8a–d** in 55–65% yield.^29^

**Scheme 3:**
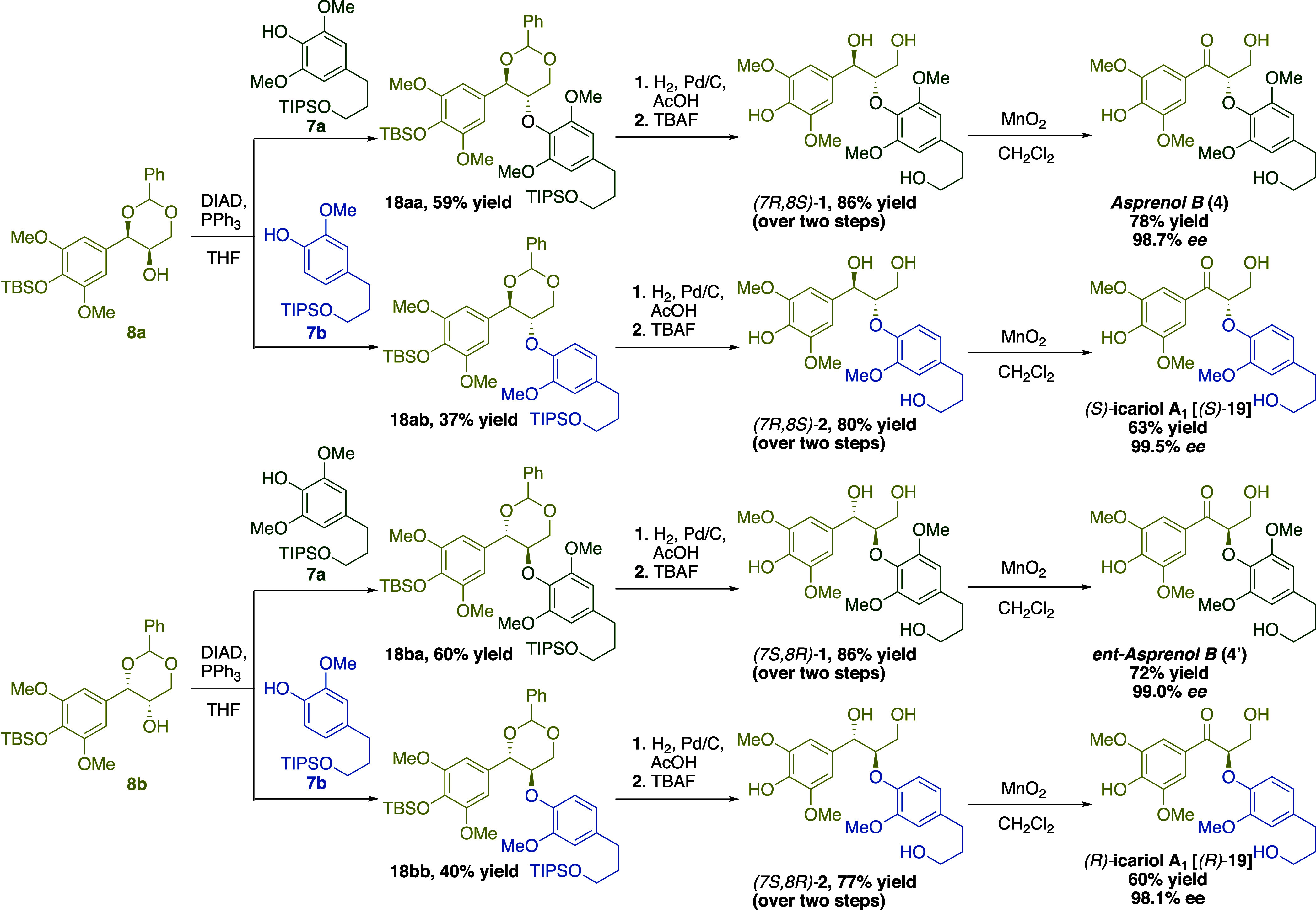
Modular, Mix-and-Match Approach toward the Enantioselective Total Syntheses of 7,9-Dihydroxy-8,4′-Oxyneolignans **1** and **2** and 9-Hydroxy-7-Keto-8,4′-Oxyneolignans Asprenol B and Icariol A_1_

With enantiomeric pairs of dioxanols **8a–d** and two phenol derivatives **7a** and **7b** in hand, we attempted the mix-and-match sequence for Mitsunobu coupling using DIAD and PPh_3_ at room temperature (Scheme 3). Syringaldehyde-derived (7*R*,8*R*)-dioxanol **8a** gave the respective (7*R*,8*S*)-aryl ethers **18aa** and **18ab** in 59 and 37% yield using phenols **7a** and **7b**. Likewise, (7*S*,8*S*)-dioxanol **8b** provided (7*S*,8*R*)-aryl ethers **18ba** and **18bb** in similar yields. In the cases of **18ab** and **18bb**, DIAD–dioxolane and dioxolane–PPh_3_ adducts appeared to be the major components following the Mitsunobu. Further addition of phenol or heating the reactions failed to improve the yields. Moving forward toward target synthesis, sequential deprotection of aryl ethers **18** was achieved by first removing the benzylidene acetals via hydrogenation followed by fluoride-mediated silyl ether deprotection to reveal the desired *erythro*-7,9-dihydroxy-8,4′-oxyneolignan enantiomers in up to 86% yield over the two steps. Attempts to cleave all three protecting groups simultaneously under various acidic conditions proved to be fruitless. From dioxanols **8a** or **8b** with phenol **7a**, the (7*R*,8*S*)- and (7*S*,8*R*)-enantiomers of oxyneolignans **1** were obtained separately. Likewise, for phenol **7b**, the (7*R*,8*S*)- and (7*S*,8*R*)-enantiomers of oxyneolignans **2** were generated.

To complete the synthesis of the 9-hydroxy-7-keto derivatives, selective benzylic oxidation^30^ of 7,9-dihydroxy-8,4′-oxyneolignans **1** and **2** was performed using MnO_2_. Mapping this sequence out from enantiopure dioxanols **8a** or **8b** with phenol **7a** afforded asprenol B **4** and its enantiomer **4′** with 98.7 and 99.0% ee, respectively. Similarly, using dioxanols **8a** and **8b** with phenol **7b**, the (*R*)- and (*S*)-enantiomers of icariol A_1_**19**,^31^ an oxyneolignan from *Epimedium sagittatum*, were prepared with high enantioselectivities (e.g., 98.1 and 99.5% ee, respectively).

**Scheme 4:**
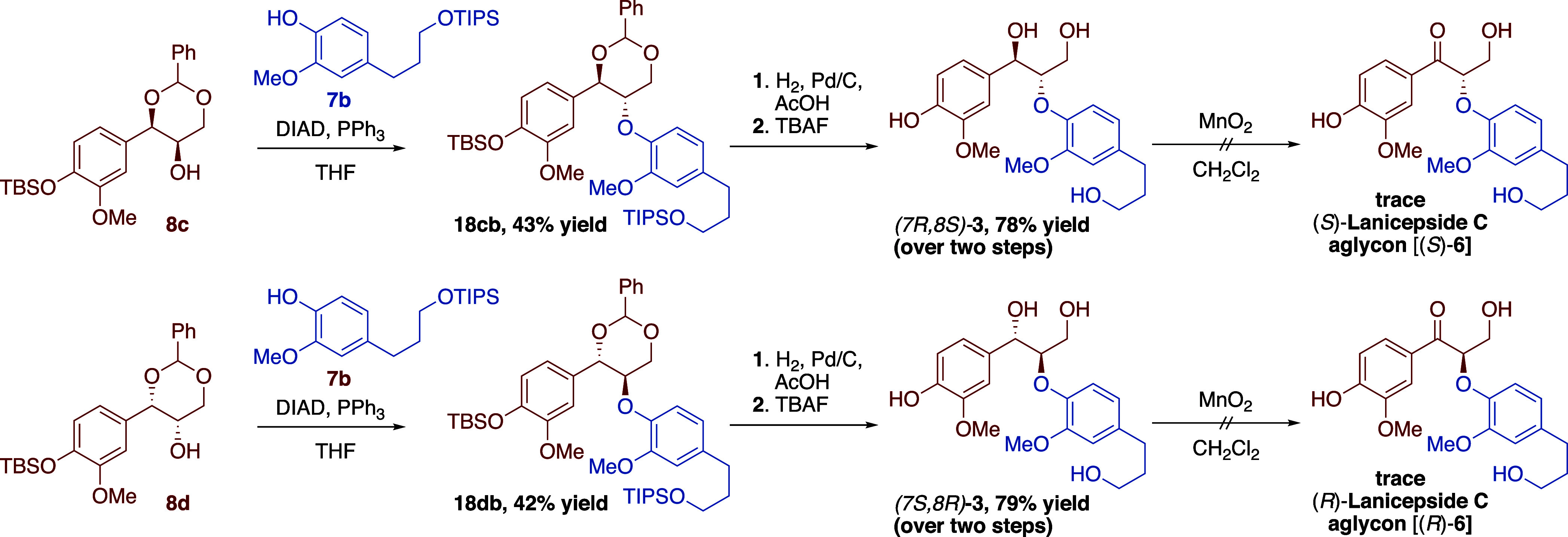
Synthetic Efforts toward the Enantiomers of Lanicepside C Aglycon **6**

As described above, the vanillin-derived, enantiopure dioxanols **8c** and **8d** provided the corresponding enantiomeric aryl ethers **18cb** and **18db** upon reaction with phenol **7b** (Scheme 4). Sequential deprotection readily provided the desired *erythro*-7,9-dihydroxy-8,4′-oxyneolignan enantiomers (*7R,8S*)- and (*7S,8R*)-**3** in 78 and 79% yields, respectively, over two steps. Unfortunately, benzylic oxidation of oxyneolignans **3** did not proceed smoothly as was the case for the other 7,9-dihydroxy-8,4′-oxyneolignan derivatives.^32^ Though it appeared that a small amount of oxidation product was present, as observed via thin-layer chromatography (TLC) and crude ^1^H Nuclear magnetic resonance (NMR), no desired lanicepside C aglycon **6** could be isolated. It was primarily observed that degradation of **3** occurred, which has been shown to take place by a variety of known mechanisms.^33^ As compared to the previous examples, the observed degradation is likely due to the loss of one of the electron-withdrawing *meta*-substituted methoxy groups. The change in substituent allows more electron donation from the *para*-substituted hydroxy group electronics of the aromatic ring, allowing for unwanted side reactions to take place. Lowering the reaction temperature or changing the solvent (i.e., acetone) gave the same outcome. Similarly, attempts to change the order of steps (by performing benzylidene deprotection, followed by benzylic oxidation and then silyl deprotection) only provided a complex mixture with only trace target material. Nonetheless, enantioselective synthesis of lanicepside C aglycon remains a worthwhile endeavor as the enantiomers have been shown to possess promising antioxidant activity.

## Conclusions

**Figure 3:**
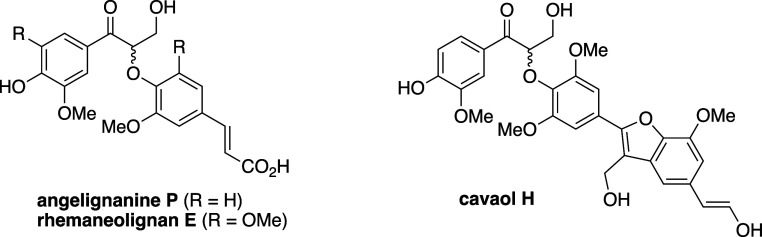
Representative 8,4′-Oxyneoligans with C(sp^2^)-substituents at C-1′.

In conclusion, we have established a general and modular Mitsunobu coupling approach for the enantioselective synthesis of *erythro*-7,9-dihydroxy- and 9-hydroxy-7-keto-8,4′-oxyneolignans. Using a templated, mix-and-match approach with enantioenriched dioxanols and functionalized phenols, both of which were derived from either syringaldehyde or vanillin, we have achieved the enantioselective total syntheses of each enantiomer of three *erythro*-7,9-dihydroxy-8,4′-oxyneolignans and two 7-keto-8,4′-oxyneolignans. Each *erythro*-7,9-dihydroxy-8,4′-oxyneolignan enantiomer was prepared in 9 steps in as high as 25% overall yield. Subsequent benzylic oxidation provided both enantiomers of the 9-hydroxy-7-keto-8,4′-oxyneolignans, asprenol B and icariol A_1_, in up to 19% overall yield. Given the modularity of the method, we have begun the enantioselective syntheses of the *threo*-isomers (from a *cis*-enoate) and oxyneolignan derivatives containing C(sp^2^)-substituents at C-1′ (Figure 3).^34^ Efforts are also underway to address the unwanted degradation observed during the benzylic oxidation of one set of oxyneolignan derivatives. Furthermore, biological activity studies of these systems are currently underway with a focus on structure–activity relationship determination made possible by the modularity of our synthetic approach. These studies will be reported in due course.

## Experimental Section

### General Information

Chromatographic purification was performed as flash chromatography with silicycle silica gel (40–65 μm) or preparative TLC (prep-TLC) using silicycle silica gel F_254_ (1000 μm) plates and solvents indicated as eluent with 0.1–0.5 bar pressure. For quantitative flash chromatography, technical-grade solvents were utilized. Analytical TLC was performed on alumina F_254_ TLC glass plates. Visualization was accomplished with ultraviolet light. Proton and carbon NMR spectra (^1^H NMR, ^13^C NMR) were recorded on a Bruker 700 MHz spectrometer or a Bruker 500 MHz spectrometer with solvent resonances as the internal standard (^1^H NMR: CDCl_3_ at 7.26 ppm; ^13^C NMR: CDCl_3_ at 77.0 ppm). MestReNova (version 11.0) was used to analyze NMR spectra. ^1^H NMR data are reported as follows: chemical shift (ppm), multiplicity (s = singlet, d = doublet, dd = doublet of doublets, dt = doublet of triplets, ddd = doublet of doublet of doublets, t = triplet, m = multiplet, and br = broad), coupling constants (Hz), and integration. ^13^C NMR data were recorded as proton-decoupled spectra. Mass spectra were obtained MicroMass Autospec M. Mass spectra were obtained through EI on Agilent 1260 Liquid chromatograph coupled to mass spectrometry 6120BMS MS. The accurate mass analyses run in electrospray ionization mode with a range of 100–2000 *m*/*z*.

### General Procedure for Silyl Ether Protection

To a clean, dry round-bottom flask was added alcohol (1 equiv) and anhydrous solvent (0.1 M). The mix was stirred under a nitrogen atmosphere and cooled to 0 °C. Base (1.2–2.5 equiv) and optional DMAP (catalytic) were then added followed by addition of silyl chloride or triflate (1.1–1.2 equiv) and the reaction was then allowed to warm to room temperature and stirred overnight. Upon consumption of starting material as monitored by TLC, the mix was then diluted with solvent (CH_2_Cl_2_ or EtOAc) and DI H_2_O. The organic layer was then washed with saturated NH_4_Cl (aq) (1×), followed by extraction of the aqueous layer with a solvent (3×) before being dried over Na_2_SO_4_. The organics were then filtered over a pad of Celite, concentrated in vacuo, and purified by flash column chromatography on SiO_2_.

### 4-((*tert*-Butyldimethylsilyl)oxy)-3,5-dimethoxybenzaldehyde (**14a**)

The product was prepared by the general silyl ether protection procedure using commercially available syringaldehyde **12** (20.0 g, 0.111 mol), imidazole (15.0 g, 0.220 mol), DMAP (0.672 g, 5.50 mmol), and TBSCl (20.0 g, 0.132 mol) in CH_2_Cl_2_ (550 mL). After workup, the residue was purified by column chromatography on silica gel (8% EtOAc/hexanes) to afford compound **14a** as a white solid (31.6 g, 97% yield). ^1^H NMR (500 MHz, CDCl_3_): δ 9.81 (s, 1H), 7.09 (s, 2H), 3.85 (s, 6H) 1.00 (s, 9H), 0.15 (s, 6H) ppm. ^13^C{^1^H} NMR (125 MHz, CDCl_3_): δ 191.0, 152.0, 140.6, 129.3, 106.7, 55.8, 25.7, 18.8, −4.6 ppm. All spectroscopic data were consistent with those previously reported.^35^

### 4-((*tert*-Butyldimethylsilyl)oxy)-3-methoxybenzaldehyde (**14b**)

The product was prepared by the general silyl ether protection procedure using commercially available vanillin **13** (20.1 g, 0.132 mol), imidazole (18.0 g, 0.264 mol), DMAP (0.805 g, 6.59 mmol), and TBSCl (23.9 g, 0.158 mol) in CH_2_Cl_2_ (650 mL). After workup, the residue was purified by column chromatography on silica gel (8% EtOAc/hexanes) to afford compound **14b** as a pale yellow oil (31.7 g, 90% yield). ^1^H NMR (500 MHz, CDCl_3_): δ 9.78 (s, 1H), 7.34 (d, *J* = 1.9 Hz, 1H), 7.31(dd, *J* = 8.0, 1.9 Hz, 1H) 6.90 (d, *J* = 8.0 Hz, 1H), 3.80 (s, 3H), 0.95 (s, 9H), 0.14 (s, 6H) ppm. ^13^C{^1^H} NMR (125 MHz, CDCl_3_): δ: 191.0, 151.6, 151.3, 130.9, 126.3, 120.7, 110.1, 55.4, 25.6, 18.5, −4.6 ppm. All spectroscopic data were consistent with those previously reported.^36^

### General Horner–Wadsworth–Emmons Olefination Procedure

To a stirring suspension of NaH (60% in mineral oil, 1.4 equiv) in anhydrous THF (0.5 M) under a nitrogen atmosphere at 0 °C was added triethyl phosphonoacetate (1.3 equiv) dropwise. The mixture was stirred at this temperature for 30 min followed by the addition of aldehyde (1 equiv) and stirring was continued at room temperature until full conversion of starting material as monitored by TLC. The reaction was then cooled to 0 °C, quenched by the slow addition of H_2_O, and the phases were separated. The aqueous phase was extracted with EtOAc (2×), and the combined organic extracts were washed with brine, dried over anhydrous Na_2_SO_4_, filtered, and concentrated in vacuo. The crude product was then purified by flash column chromatography on SiO_2_.

### Ethyl (*E*)-3-(4-((*tert*-Butyldimethylsilyl)oxy)-3,5-dimethoxyphenyl)acrylate (**11a**)

The product was prepared following the general olefination procedure from aldehyde **14a** (12.2 g, 41.0 mmol), NaH (2.27 g, 56.7 mmol), and triethyl phosphonoacetate (11.7 g, 52.0 mmol) in THF (80 mL). After workup, the residue was purified by column chromatography on silica gel (5% EtOAc/hexanes) to afford compound **11a** as a white solid (14.1 g, 93% yield). ^1^H NMR (500 MHz, CDCl_3_): δ 7.59 (d, *J* = 15.9 Hz, 1H), 6.72 (s, 2H), 6.30 (d, *J* = 15.8 Hz, 1H), 4.25 (q, *J* = 7.1 Hz, 2H), 3.81 (s, 6H), 1.33 (t, *J* = 7.1 Hz, 3H), 1.00 (s, 9H), 0.13 (s, 6H) ppm. ^13^C{^1^H} NMR (125 MHz, CDCl_3_): δ 167.2, 151.7, 145.0, 136.8, 127.1, 116.1, 105.3, 60.3, 55.7, 25.7, 18.7, 14.4, −4.6 ppm. All spectroscopic data were consistent with those previously reported.^37^

### Ethyl (*E*)-3-(4-((*tert*-Butyldimethylsilyl)oxy)-3-methoxyphenyl)acrylate (**11b**)

The product was prepared following the general olefination procedure from aldehyde **14b** (15.1 g, 56.8 mmol), NaH (3.19 g, 79.7 mmol), and triethyl phosphonoacetate (16.5 g, 73.6 mmol) in THF (110 mL). After workup, the residue was purified by column chromatography on silica gel (5% EtOAc/hexanes) to afford compound **11b** as a clear oil (18.0 g, 94% yield). ^1^H NMR (500 MHz, CDCl_3_): δ 7.61 (d, *J* = 15.9 Hz, 1H), 7.03–6.98 (m, 1H), 6.83 (d, *J* = 8.6 Hz, 1H), 6.29 (d, *J* = 15.9 Hz, 1H), 4.24 (q, *J* = 7.1 Hz, 2H), 3.82 (s, 3H), 1.32 (t, *J* = 7.1 Hz, 3H), 0.99 (s, 9H), 0.16 (s, 6) ppm. ^13^C{^1^H} NMR (125 MHz, CDCl_3_): δ 167.3, 151.2, 147.5, 144.7, 128.3, 122.2, 121.1, 116.0, 110.8, 60.3, 55.4, 25.7, 18.5, 14.4, −4.6 ppm. All spectroscopic data were consistent with those previously reported.^38^

### General LiAlH_4_ Reduction Procedure

To a stirring solution of enoate **11** (1 equiv) in anhydrous THF (0.2 M) under a nitrogen atmosphere and cooled to 0 °C was added LiAlH_4_ (2 equiv) in small portions. The reaction was then allowed to warm to room temperature and stirred an additional 5 h before being cooled to 0 °C and quenched with ice cold DI water, 15% NaOH (aq), and then ice cold DI water again. The slurry was then filtered, the aqueous phase was extracted with EtOAc (3×), and the combined organics were dried over Na_2_SO_4_, filtered over a pad of Celite, and concentrated in vacuo. The crude product was then purified by flash column chromatography on SiO_2_.

### 3-(4-((*tert*-Butyldimethylsilyl)oxy)-3,5-dimethoxyphenyl)propan-1-ol (**15a**)

The product was prepared by the general LiAlH_4_ reduction procedure from enoate **11a** (5.01 g, 13.7 mmol) and LiAlH_4_ (1.04 g, 27.3 mmol) in THF (70 mL). After workup, the residue was purified by column chromatography on silica gel (10 → 30% EtOAc/hexanes) to afford compound **15a** as a pale yellow oil (3.12 g, 70% yield). ^1^H NMR (500 MHz, CDCl_3_): δ 6.37 (s, 2H), 3.76 (s, 6H), 3.65 (t, *J* = 6.4 Hz, 2H), 2.62 (t, *J* = 7.4 Hz, 2H), 1.94–1.79 (m, 2H), 1.00 (s, 9H), 0.11 (s, 6H) ppm. ^13^C{^1^H} NMR (125 MHz, CDCl_3_): δ 151.4, 134.3, 132.4, 105.4, 62.4, 55.8, 34.3, 32.4, 25.8, 18.7, −4.7 ppm. All spectroscopic data were consistent with those previously reported.^39^

### 3-(4-((*tert*-Butyldimethylsilyl)oxy)-3-methoxyphenyl)propan-1-ol (**15b**)

The product was prepared by the general LiAlH_4_ reduction procedure from enoate **11b** (5.01 g, 14.9 mmol) and LiAlH_4_ (1.13 g, 29.8 mmol) in THF (75 mL). After workup, the residue was purified by column chromatography on silica gel (10 → 30% EtOAc/hexanes) to afford compound **15b** as a pale yellow oil (3.13 g, 71% yield). ^1^H NMR (500 MHz, CDCl_3_): δ 6.76 (d, *J* = 8.0 Hz, 1H), 6.69 (d, *J* = 2.1 Hz, 1H), 6.63 (dd, *J* = 8.0, 2.1 Hz, 1H), 3.78 (s, 3H), 3.65 (t, *J* = 6.4 Hz, 2H), 2.63 (t, *J* = 7.4 Hz, 2H), 1.91–1.79 (m, 3H), 0.99 (s, 9H), 0.15 (s, 6H) ppm. ^13^C{^1^H} NMR (125 MHz, CDCl_3_): δ 150.7, 143.1, 135.2, 120.7, 120.4, 112.5, 62.4, 55.5, 34.3, 31.8, 25.7, 18.4, −4.7 ppm. All spectroscopic data were consistent with those previously reported.^40^

### *tert*-Butyl(2,6-dimethoxy-4-(3-((triisopropylsilyl)oxy)propyl)phenoxy) Dimethylsilane (**16a**)

The product was prepared by the general silyl ether protection procedure using **15a** (2.22 g, 6.81 mmol), 2,6-lutidine (0.887 g, 8.28 mmol), and TIPSOTf (2.31 g, 7.54 mmol) in CH_2_Cl_2_ (70 mL). After workup, the residue was purified by column chromatography on silica gel (2% Et_2_O/pentane) to afford compound **16a** as a pale yellow oil (3.19 g, 97% yield). ^1^H NMR (500 MHz, CDCl_3_): δ 6.38 (s, 2H), 3.78 (s, 6H), 3.70 (t, *J* = 6.4 Hz, 2H), 2.64 (t, *J* = 7.3 Hz, 2H), 1.90–1.80 (m, 2H), 1.12–1.05 (m, 21H), 1.02 (s, 9H), 0.13 (s, 5H) ppm. ^13^C{^1^H} NMR (125 MHz, CDCl_3_): δ 151.3, 134.8, 132.2, 105.5, 62.5, 55.7, 34.6, 32.2, 25.8, 18.0, 12.0, −4.7 ppm. HRMS (ESI) (*m*/*z*): [M + H]^+^ calcd for C_26_H_50_O_4_Si_2_, 482.3248; found, 482.3244.

### *tert*-Butyl(2-methoxy-4-(3-((triisopropylsilyl)oxy)propyl)phenoxy) Dimethylsilane (**16b**)

The product was prepared by the general silyl ether protection procedure using **15b** (4.15 g, 14.0 mmol), 2,6-lutidine (1.75 g, 16.4 mmol), and TIPSOTf (4.56 g, 14.9 mmol) in CH_2_Cl_2_ (140 mL). After workup, the residue was purified by column chromatography on silica gel (1% Et_2_O/pentane) to afford compound **16b** as a pale yellow oil (6.17 g, 97% yield). ^1^H NMR (500 MHz, CDCl_3_): δ 6.80–6.62 (m, 3H), 3.80 (s, 3H), 3.70 (t, *J* = 6.4 Hz, 2H), 2.65 (t, *J* = 7.6 Hz, 2H), 1.91–1.79 (m, 2H), 1.18–1.05 (m, 21H), 1.01 (s, 9H), 0.16 (s, 5H) ppm. ^13^C{^1^H} NMR (125 MHz, CDCl_3_): δ 150.6, 142.9, 135.8, 120.6, 120.5, 112.6, 62.5, 55.4, 34.7, 31.7, 31.6, 25.7, 18.0, 12.0, −4.7 ppm. HRMS (ESI) (*m*/*z*): [M + H]^+^ calcd for C_25_H_48_O_3_Si_2_, 452.3142; found, 452.3143.

### General Selective Aryl Silyl Ether Deprotection Procedure

To a stirring solution of aryl silyl ether (1 equiv) in anhydrous DMF and DI water (0.2 M, 50:1) was added LiOAc·2H_2_O (0.1 equiv) and the reaction was heated to 70 °C. Upon complete consumption of the starting material as monitored by TLC (1–2 days), the reaction was cooled, diluted with Et_2_O, and washed with brine (2×). The organic material was then dried over Na_2_SO_4_, filtered over a pad of Celite, and concentrated in vacuo. The crude product was then purified by flash column chromatography on SiO_2_.

### 2,6-Dimethoxy-4-(3-((triisopropylsilyl)oxy)propyl)phenol (**7a**)

The product was prepared following the general selective aryl silyl ether deprotection procedure using **16a** (3.11 g, 6.45 mmol) and LiOAc·2H_2_O (67.8 g, 0.665 mmol) in DMF/H_2_O (31:0.5 mL). After workup, the residue was purified by column chromatography on silica gel (3% EtOAc/hexanes) to afford compound **7a** as a pale yellow oil (1.92 g, 81% yield). ^1^H NMR (500 MHz, CDCl_3_): δ 6.43 (s, 2H), 5.36 (s, 1H), 3.87 (s, 6H), 3.70 (t, *J* = 6.3 Hz, 2H), 2.64 (t, *J* = 7.6 Hz, 2H), 1.86–1.81 (m, 2H), 1.10–1.05 (m, 21H) ppm. ^13^C{^1^H} NMR (125 MHz, CDCl_3_): δ 146.8, 133.4, 132.6, 105.0, 62.4, 56.2, 34.8, 32.2, 18.0, 12.0 ppm. HRMS (ESI) (*m*/*z*): [M + H]^+^ calcd for C_20_H_37_O_4_Si, 369.2456; found, 369.2453.

### 2-Methoxy-4-(3-((triisopropylsilyl)oxy)propyl)phenol (**7b**)

The product was prepared following the general selective aryl silyl ether deprotection procedure using **16b** (6.05 g, 13.4 mmol) and LiOAc·2H_2_O (0.136 g, 1.34 mmol) in DMF/H_2_O (65:1 mL). After workup, the residue was purified by column chromatography on silica gel (3% EtOAc/hexanes) to afford compound **7b** as a clear oil (3.411 g, 75% yield). ^1^H NMR (500 MHz, CDCl_3_): δ 6.83 (d, *J* = 7.9 Hz, 1H), 6.72–6.69 (m, 2H), 5.50 (s, 1H), 3.88 (s, 3H), 3.71 (t, *J* = 6.3 Hz, 2H), 2.65 (t, *J* = 7.4 Hz, 2H), 1.87–1.81 (m, 2H), 1.10–1.07 (m, 21H) ppm. ^13^C{^1^H} NMR (125 MHz, CDCl_3_): δ 146.3, 143.5, 134.3, 121.0, 114.1, 111.1, 62.5, 55.8, 34.9, 31.7, 18.0, 12.0 ppm. HRMS (ESI) (*m*/*z*): [M + H]^+^ calcd for C_19_H_35_O_3_Si, 339.2349; found, 339.2347.

### General DIBAL-H Reduction Procedure

To a stirring solution of α,β-unsaturated ester (1 equiv) in anhydrous solvent (0.5 M) under N_2_ at 0 °C was added DIBAL-H (1 M in CH_2_Cl_2_ 1.5–2.3 equiv) dropwise. The mixture continued stirring at this temperature until full conversion of the starting material as monitored by TLC. The reaction was then quenched by the dropwise addition of a saturated aqueous solution of Rochelle’s salt and stirred overnight. The aqueous layer was then separated and extracted with CH_2_Cl_2_. The combined organic material was then dried over anhydrous Na_2_SO_4_, filtered, and concentrated in vacuo. The crude product was then purified by flash column chromatography on SiO_2_.

### (*E*)-3-(4-((*tert*-butyldimethylsilyl)oxy)-3,5-dimethoxyphenyl)prop-2-en-1-ol (**10a**)

The product was prepared following the general DIBAL-H reduction procedure using **11a** (28.5 g, 77.8 mmol) and DIBAL-H (35 mL, 0.194 mol) in THF (150 mL). After workup, the residue was purified by column chromatography on silica gel (30% EtOAc/hexanes) to afford compound **10a** as a white solid (23.9 g, 95% yield). ^1^H NMR (500 MHz, CDCl_3_): δ 6.57 (s, 2H), 6.49 (d, *J* = 15.8 Hz, 1H), 6.23 (dt, *J* = 15.8, 5.9 Hz, 1H), 4.27 (dd, *J* = 5.9, 1.5 Hz, 2H), 3.78 (s, 3H), 1.89 (br s, 1H), 1.01 (s, 9H), 0.13 (s, 6H) ppm. ^13^C{^1^H} NMR (125 MHz, CDCl_3_): δ 151.7, 134.5, 131.7, 129.3, 126.7, 103.7, 63.8, 55.7, 25.8, 18.7, −4.7 ppm. All spectroscopic data were consistent with those previously reported.^37^

### (*E*)-3-(4-((*tert*-butyldimethylsilyl)oxy)-3-methoxyphenyl)prop-2-en-1-ol (**10b**)

The product was prepared following the general DIBAL-H reduction procedure using **11b** (12.0 g, 35.7 mmol) and DIBAL-H (16 mL, 88.8 mmol) in THF (70 mL). After workup, the residue was purified by column chromatography on silica gel (30% EtOAc/hexanes) to afford compound **10b** as a pale yellow oil (9.45 g, 90% yield). ^1^H NMR (500 MHz, CDCl_3_): δ 6.91–6.77 (m, 3H), 6.52 (d, *J* = 15.9 Hz, 1H), 6.22 (dt, *J* = 15.8, 5.9 Hz, 1H), 4.28 (d, *J* = 4.3 Hz, 2H), 3.81 (s, 3H), 1.76 (br s, 1H), 1.00 (s, 9H), 0.16 (s, 6H) ppm. ^13^C{^1^H} NMR (125 MHz, CDCl_3_): δ 151.0, 145.0, 131.3, 130.6, 126.5, 120.9, 119.6, 109.8, 63.8, 55.4, 25.7, 18.4, −4.7 ppm. All spectroscopic data were consistent with those previously reported.^38^

### Sharpless Asymmetric Dihydroxylation Procedure

To a stirring solution of AD-mix (1.4 g/1 mmol of allylic alcohol) in tBuOH and DI water (0.1 M, 1:1) was added MeSO_2_NH_2_ (95 mg/1 mmol of allylic alcohol) and the mixture stirred at room temperature until all solids dissolved. The solution was then cooled to 0 °C and allylic alcohol (1 equiv) was added in one portion. The reaction was warmed to room temperature and stirred overnight before being quenched with Na_2_SO_3_ (1.5 g/1 mmol allylic alcohol) and stirred an additional 30 min before being extracted with CH_2_Cl_2_ (3×), dried over Na_2_SO_4_, filtered over a pad of Celite, and concentrated in vacuo. The crude product was then purified by flash column chromatography on SiO_2_.

### (1*R*,2*R*)-1-(4-((*tert*-Butyldimethylsilyl)oxy)-3,5-dimethoxyphenyl) Propane-1,2,3-triol [(*R*,*R*)-**9a**]

The product was prepared from **10a** (4.507 g, 13.89 mmol), AD-mix-β (19.42 g), MeSO_2_NH (1.317 g), and Na_2_SO_3_ (20.80 g) in *t*BuOH (70 mL) and DI water (70 mL). After workup and purification by silica gel chromatography (2 → 5% MeOH/CH_2_Cl_2_), (*R*,*R*)-**9a** was delivered as an off-white solid (4.785 g, 96% yield, >99% ee). It was visualized by TLC using a vanillin stain. ^1^H NMR (500 MHz, CDCl_3_): δ 6.53 (s, 2H), 4.53 (d, *J* = 7.2 Hz, 1H), 3.75–3.71 (s + bs, 7H), 3.52–3.38 (m, 3H), 0.98 (s, 9H), 0.09 (s, 6H). ^13^C{^1^H} NMR (125 MHz, CDCl_3_): δ 151.8, 134.2, 133.3, 103.8, 76.3, 75.3, 63.4, 55.9, 25.9, 18.8, −4.50. HRMS (ESI) (*m*/*z*): [M + H]^+^ calcd for C_17_H_31_O_6_Si, 359.1884; not detected. HPLC (RegisReflect C-AmyloseA, isopropanol/hexanes = 5/95, flow rate = 1.5 mL/min, l = 254 nm) *t*_1_ = 9.188 min (minor), *t*_2_ = 12.800 min (major). All spectroscopic data were consistent with those previously reported.^41^

### (1*S*,2*S*)-1-(4-((*tert*-Butyldimethylsilyl)oxy)-3,5-dimethoxyphenyl) Propane-1,2,3-triol [(*S*,*S*)-**9b**]

The product was prepared following the general Sharpless asymmetric dihydroxylation procedure using **10a** (5.50 g, 13.6 mmol), AD-mix-α (23.8 g), MeSO_2_NH_2_ (1.62 g), and Na_2_SO_3_ (25.5 g) in tBuOH (80 mL) and DI water (80 mL). After workup, the residue was purified by column chromatography on silica gel (2 → 5% MeOH/CH_2_Cl_2_) to afford (*S*,*S*)-**9b** as an off-white solid (5.95 g, 98% yield, >99% ee). It was visualized by TLC using a vanillin stain. ^1^H NMR (500 MHz, CDCl_3_): δ 6.52 (s, 2H), 4.53 (d, *J* = 7.2 Hz, 1H), 3.82 (br s, 1H), 3.73 (s, 6H), 3.51–3.39 (m, 2H), 0.98 (s, 9H), 0.09 (s, 6H) ppm. ^13^C{^1^H} NMR (125 MHz, CDCl_3_): δ 151.8, 134.2, 133.3, 103.8, 76.3, 75.3, 63.4, 55.9, 25.9, 18.8, −4.5 ppm. HPLC (RegisReflect C-AmyloseA, isopropanol/hexanes = 5/95, flow rate = 1.5 mL/min, l = 254 nm) *t*_1_ = 9.856 min (major), *t*_2_ = 11.728 min (minor). All spectroscopic data were consistent with those previously reported.^41^

### (1*R*,2*R*)-1-(4-((*tert*-Butyldimethylsilyl)oxy)-3-methoxyphenyl)propane-1,2,3-triol [(*R*,*R*)-**9c**]

The product was prepared following the general Sharpless asymmetric dihydroxylation procedure using **10b** (3.72 g, 12.6 mmol), AD-mix-β (17.6 g), MeSO_2_NH_2_ (1.19 g), and Na_2_SO_3_ (18.9 g) in tBuOH (70 mL) and DI water (70 mL). After workup, the residue was purified by column chromatography on silica gel (2 → 5% MeOH/CH_2_Cl_2_) to afford (*R*,*R*)-**9c** as a pale yellow solid (3.78 g, 91% yield, >99% ee). It was visualized by TLC using a vanillin stain. ^1^H NMR (500 MHz, CDCl_3_): δ 6.86 (d, *J* = 2.0 Hz, 1H), 6.80–6.74 (m, 2H), 4.55 (d, *J* = 7.2 Hz, 1H), 3.77–3.74 (s + bs, 4H), 3.52–3.39 (m, 2H), 0.98 (s, 9H), 0.12 (s, 6H) ppm. ^13^C{^1^H} NMR (125 MHz, CDCl_3_): δ 151.3, 145.1, 134.1, 121.0, 119.3, 110.7, 76.3, 75.0, 63.5, 55.7, 25.9, 18.6, −4.47, −4.49 ppm. HRMS (ESI) (*m*/*z*): [M + H]^+^ calcd for C_16_H_29_O_5_Si, 329.1779; not detected. HPLC (RegisReflect C-AmyloseA, isopropanol/hexanes = 5/95, flow rate = 1.5 mL/min, l = 254 nm) *t*_1_ = 10.748 min (minor), *t*_2_ = 11.836 min (major). All spectroscopic data were consistent with those previously reported.^41^

### Synthesis of (1*S*,2*S*)-1-(4-((*tert*-Butyldimethylsilyl)oxy)-3-methoxyphenyl)propane-1,2,3-triol [(*S*, *S*)-**9d**]

The product was prepared from **10b** (4.016 g, 13.62 mmol), AD-mix-α (19.207 g), MeSO_2_NH (1.292 g), and Na_2_SO_3_ (20.47 g) in *t*BuOH (70 mL) and DI water (70 mL). After workup and purification by silica gel chromatography (2 → 5% MeOH/CH_2_Cl_2_), (*S*,*S***)-9d** was delivered as a pale yellow solid (4.317 g, 96% yield, >99% ee). It was visualized by TLC using a vanillin stain. ^1^H NMR (500 MHz, CDCl_3_): δ 6.87 (s, 1H), 6.78–6.72 (m, 2H), 4.55 (d, *J* = 4.1 Hz, 1H), 3.78 (br s, 1H), 3.72 (s, 3H), 3.49–3.37 (m, 2H), 0.97 (s, 9H), 0.11 (s, 6H). ^13^C{^1^H} NMR (125 MHz, CDCl_3_): δ 151.2, 145.1, 134.1, 121.0, 119.4, 110.9, 76.2, 74.9, 63.3, 55.7, 25.9, 18.6, −4.47, −4.48. HPLC (RegisReflectC-AmyloseA, isopropanol/hexanes = 5/95, flow rate = 1.5 mL/min, l = 254 nm) *t*_1_ = 10.128 min (major), *t*_2_ = 13.392 min (minor). All spectroscopic data were consistent with those previously reported.^41^

### General Procedure for Benzylidene Acetal Formation

A stirring solution of triol (1 equiv) in anhydrous CH_2_Cl_2_ (0.4 M) under N_2_ was cooled to 0 °C. Then, benzaldehyde dimethyl acetal (1.3 equiv) was added followed by TsOH (0.05 equiv) After conversion of the starting material, as monitored by TLC, the reaction was quenched by the addition of NEt_3_ and DI water. The aqueous layer was extracted with CH_2_Cl_2_ (2×) and the organics were dried over Na_2_SO_4_, filtered, and concentrated in vacuo. The crude product was then partially purified by flash column chromatography (5 → 10% EtOAc/hexanes) on SiO_2_ to isolate the dioxane/dioxolane mixture. The crude isomer mixture (1 equiv) was then dissolved in anhydrous CH_2_Cl_2_ (0.3 M) and NEt_3_ (2 equiv) and TsCl (1 equiv) were added. After consumption of the dioxolane byproduct as monitored by TLC, the reaction was quenched by the addition of MeOH followed by DI water. The aqueous layer was extracted with CH_2_Cl_2_ (2×) and the organics were dried over Na_2_SO_4_, filtered, and concentrated in vacuo. The crude product was then purified by flash column chromatography on SiO_2_.

### (4*R*,5*R*)-4-(4-((*tert*-Butyldimethylsilyl)oxy)-3,5-dimethoxyphenyl)-2-phenyl-1,3-dioxan-5-ol [(*R*,*R*)-**8a**]

The product was prepared following the general benzylidene acetal formation procedure using (*R*,*R*)-**9a** (4.67 g, 13.0 mmol), benzaldehyde dimethyl acetal (2.52 g, 16.6 mmol), TsOH (0.135 g, 0.786 mmol), NEt_3_ (1.59 g, 15.7 mmol), and TsCl (1.64 g, 8.76 mmol) in CH_2_Cl_2_ (35 and then 26 mL). After workup, the residue (4.79 g, 2.3:1 dioxane to dioxolane) was purified by column chromatography on silica gel (20% Et_2_O/hexanes) to afford (*R*,*R*)-**8a** and was delivered as an off-white solid (3.34 g, 57% yield). It was visualized by TLC using a vanillin stain. ^1^H NMR (500 MHz, CDCl_3_): δ 7.64–7.58 (m, 2H), 7.45–7.38 (m, 3H), 6.65 (s, 2H), 5.77 (s, 1H), 5.02 (s, 1H), 4.37 (dd, *J* = 11.9, 3.4 Hz, 1H), 4.23 (dd, *J* = 11.9, 3.1 Hz, 1H), 3.82–3.78 (s + m, 7H), 2.40 (d, *J* = 8.3 Hz, 1H), 1.03 (s, 9H), 0.14 (s, 6H) ppm. ^13^C{^1^H} NMR (125 MHz, CDCl_3_): δ 151.7, 137.9, 134.1, 130.2, 129.0, 128.3, 126.1, 103.2, 101.7, 81.1, 72.2, 66.7, 55.9, 25.8, 18.7, −4.6 ppm. HRMS (ESI) (*m*/*z*): [M + NH_4_]^+^ calcd for C_24_H_38_O_6_NSi, 464.2463; found, 464.2458.

### (4*S*,5*S*)-4-(4-((*tert*-Butyldimethylsilyl)oxy)-3,5-dimethoxyphenyl)-2-phenyl-1,3-dioxan-5-ol [(*S*,*S*)-**8b**]

The product was prepared following the general benzylidene acetal formation procedure using (*S*,*S*)-**9b** (4.50 g, 12.5 mmol), benzaldehyde dimethyl acetal (2.52 g, 16.5 mmol), TsOH (0.120 g, 0.697 mmol), NEt_3_ (1.74 g, 17.2 mmol), and TsCl (1.50 g, 7.88 mmol) in CH_2_Cl_2_ (31 and then 21 mL). After workup, the residue (4.43 g, 2.3:1 dioxane to dioxolane) was purified by column chromatography on silica gel (20% Et_2_O/hexanes) to afford (*S*,*S*)-**8b** as an off-white solid (3.09 g, 55% yield). It was visualized by TLC using a vanillin stain. ^1^H NMR (500 MHz, CDCl_3_): δ 7.63–7.58 (m, 2H), 7.45–7.38 (m, 3H), 6.65 (s, 2H), 5.77 (s, 1H), 5.02 (s, 1H), 4.37 (dd, *J* = 11.8, 1.1 Hz, 1H), 4.23 (dd, *J* = 11.9, 1.5 Hz, 1H), 3.82–3.78 (s + m, 7H), 2.40 (d, *J* = 8.6 Hz, 1H), 1.03 (s, 9H), 0.14 (s, 6H) ppm. ^13^C{^1^H} NMR (125 MHz, CDCl_3_): δ 151.7, 137.9, 134.1, 130.2, 129.0, 128.3, 126.1, 103.2, 101.7, 81.1, 72.2, 66.7, 55.9, 25.8, 18.7, −4.6 ppm. HRMS (ESI) (*m*/*z*): [M + NH_4_]^+^ calcd for C_24_H_38_O_6_NSi, 464.2463; found, 464.2458.

### (4*R*,5*R*)-4-(4-((*tert*-Butyldimethylsilyl)oxy)-3-methoxyphenyl)-2-phenyl-1,3-dioxan-5-ol [(*R*,*R*)-**8c**]

The product was prepared following the general benzylidene acetal formation procedure using (*R*,*R*)-**9c** (3.60 g, 11.0 mmol), benzaldehyde dimethyl acetal (2.16 g, 14.2 mmol), TsOH (0.108 g, 0.628 mmol), NEt_3_ (1.56 g, 15.4 mmol), and TsCl (1.47 g, 7.70 mmol) in CH_2_Cl_2_ (27 and then 22 mL). After workup, the residue (3.98 g, 2.4:1 dioxane to dioxolane) was purified by column chromatography on silica gel (15% Et_2_O/hexanes) to afford (*R*,*R*)-**8c** as an off-white solid (2.81 g, 61% yield). It was visualized by TLC using a vanillin stain. ^1^H NMR (500 MHz, CDCl_3_): δ 7.60 (dd, *J* = 7.2, 1.8 Hz, 2H), 7.46–7.37 (m, 3H), 6.98 (d, *J* = 1.9 Hz, 1H), 6.93–6.84 (m, 2H), 5.78 (s, 1H), 5.03 (s, 1H), 4.36 (dd, *J* = 11.8, 1.9 Hz, 1H), 4.23 (dd, *J* = 11.8, 1.4 Hz, 1H), 3.83–3.77 (s + m, 4H), 2.43 (d, *J* = 8.9 Hz, 1H), 1.01 (s, 9H), 0.16 (s, 6H) ppm. ^13^C{^1^H} NMR (125 MHz, CDCl_3_): δ 151.0, 144.8, 137.9, 131.3, 129.0, 128.3, 126.1, 120.8, 118.4, 110.3, 101.7, 80.9, 72.3, 66.7, 55.6, 25.7, 18.4, −4.60, −4.62 ppm. HRMS (ESI) (*m*/*z*): [M + NH_4_]^+^ calcd for C_23_H_36_O_5_NSi, 434.2357; found, 434.2352.

### (4*S*,5*S*)-4-(4-((*tert*-Butyldimethylsilyl)oxy)-3-methoxyphenyl)-2-phenyl-1,3-dioxan-5-ol [(*S*,*S*)-**8d**]

The product was prepared following the general benzylidene acetal formation procedure using (*S*,*S*)-**9d** (2.99 g, 9.11 mmol), benzaldehyde dimethyl acetal (1.85 g, 12.2 mmol), TsOH (0.089 g, 0.517 mmol), NEt_3_ (1.36 g, 13.5 mmol), and TsCl (1.29 g, 6.76 mmol) in CH_2_Cl_2_ (23 mL and then 20 mL). After workup, the residue (3.56 g of 2.3:1 dioxane to dioxolane) was purified by column chromatography on silica gel (15% Et_2_O/hexanes) to afford (*S*,*S*)-**8d** as an off-white solid (2.48 g, 65% yield). It was visualized by TLC using a vanillin stain. ^1^H NMR (500 MHz, CDCl_3_): δ 7.61 (dd, *J* = 7.1, 1.8 Hz, 2H), 7.43–7.38 (m, 3H), 6.98 (d, *J* = 2.0 Hz, 1H), 6.91–6.86 (m, 2H), 5.77 (s, 1H), 5.03 (s, 1H), 4.36 (dd, *J* = 11.8, 1.9 Hz, 1H), 4.23 (dd, *J* = 11.9, 1.5 Hz, 1H), 3.83–3.78 (s + m, 4H), 2.43 (d, *J* = 8.8 Hz, 1H), 1.01 (s, 9H), 0.17 (s, 6H) ppm. ^13^C{^1^H} NMR (125 MHz, CDCl_3_): δ 151.0, 144.8, 137.9, 131.3, 129.0, 128.3, 126.1, 120.8, 118.4, 110.3, 101.7, 80.9, 72.3, 66.7, 55.6, 25.7, 18.4, −4.60, −4.61 ppm. HRMS (ESI) (*m*/*z*): [M + NH_4_]^+^ calcd for C_23_H_36_O_5_NSi, 434.2357; found, 434.2352.

### General Mitsunobu Coupling Procedure

To a stirring solution of PPh_3_ (1.2–2 equiv) in anhydrous THF (0.1 M) under N_2_ was added DIAD (1.2–2 equiv) dropwise. The mixture was stirred for 30 min before being cooled to 0 °C. A mixture of secondary alcohol (1–1.1 equiv) and phenol (1–2 equiv) in THF (0.1 M) was then added, and the reaction was then stirred at the indicated temperature. The reaction was then washed with DI H_2_O (2×), and the organics were dried over anhydrous Na_2_SO_4_, filtered, and concentrated in vacuo. The crude product was then purified by flash column chromatography on SiO_2_.

### *tert*-Butyl (4-((4*R*,5*S*)-5-(2,6-Dimethoxy-4-(3-((triisopropylsilyl)oxy)propyl)phenoxy)-2-phenyl-1,3-dioxan-4-yl)-2,6-dimethoxyphenoxy)dimethylsilane [(*R*,*S*)-**18aa**]

The product was prepared following the general Mitsunobu coupling procedure using (*R*,*R*)-**8a** (0.512 g, 1.15 mmol), **7a** (0.392 g, 1.06 mmol), DIAD (0.257 g, 1.27 mmol), and PPh_3_ (0.340 g, 1.30 mmol) in THF (11 mL) at room temperature. After workup, the residue was purified by column chromatography on silica gel (3 → 7% Et_2_O/pentane) to afford (*R*,*S*)-**18aa** as a colorless gel (0.497 g, 59% yield). It was visualized by TLC using a vanillin stain. ^1^H NMR (500 MHz, CDCl_3_): δ 7.55 (dd, *J* = 8.0, 1.8 Hz, 2H), 7.38–7.33 (m, 3H), 6.75 (s, 2H), 6.29 (s, 2H), 5.73 (s, 1H), 4.83 (d, *J* = 9.0 Hz, 1H), 4.46–4.40 (m, 2H), 4.11–4.05 (m, 1H), 3.75 (s, 6H), 3.69 (t, *J* = 6.2 Hz, 2H), 3.63 (s, 6H), 2.60 (t, *J* = 7.7 Hz, 2H), 1.83–1.77 (m, 2H), 1.07 (d, *J* = 4.7 Hz, 21H), 1.01 (s, 9H), 0.11 (s, 6H) ppm. ^13^C{^1^H} NMR (125 MHz, CDCl_3_): δ 152.6, 150.9, 138.3, 137.8, 134.2, 133.2, 131.0, 128.8, 128.2, 126.3, 105.6, 105.2, 101.4, 83.3, 74.8, 70.1, 62.3, 55.8, 55.7, 34.5, 32.4, 25.8, 18.7, 18.0, 12.0, −4.6, −4.6 ppm. HRMS (ESI) (*m*/*z*): [M + NH_4_]^+^ calcd for C_44_H_72_O_9_NSi_2_, 814.4818; found 814.4759.

### *tert*-Butyl (2,6-Dimethoxy-4-((4*R*,5*S*)-5-(2-methoxy-4-(3-((triisopropylsilyl)oxy)propyl)phenoxy)-2-phenyl-1,3-dioxan-4-yl)phenoxy)dimethylsilane [(*R*,*S*)-**18ab**]

The product was prepared following the general Mitsunobu coupling procedure using (*R*,*R*)-**8a** (0.506 g, 1.13 mmol), **7b** (0.355 g, 1.05 mmol), DIAD (0.273 g, 1.35 mmol), and PPh_3_ (0.325 g, 1.24 mmol) in THF (11 mL) at room temperature. After workup, the residue was purified by column chromatography on silica gel (2 → 5% Et_2_O/pentane) to afford (*R*,*S*)-**18ab** as a colorless gel (0.298 g, 37% yield). It was visualized by TLC using a vanillin stain. ^1^H NMR (500 MHz, CDCl_3_): δ 7.62–7.58 (m, 2H), 7.43–7.37 (m, 3H), 6.79 (s, 2H), 6.68 (s, 1H), 6.54 (dd, *J* = 8.2, 2.1 Hz, 1H), 6.35 (d, *J* = 8.1 Hz, 1H), 5.77 (s, 1H), 4.86 (d, *J* = 9.2 Hz, 1H), 4.61 (dd, *J* = 11.0, 5.0 Hz, 1H), 4.28–4.21 (m, 1H), 4.04 (t, *J* = 10.7 Hz, 1H), 3.80 (s, 3H), 3.77 (s, 6H), 3.71 (t, *J* = 6.2 Hz, 2H), 2.62 (t, *J* = 7.6 Hz, 2H), 1.85–1.78 (m, 2H), 1.13–1.09 (d + m, 21H), 1.04 (s, 9H), 0.14 (s, 6H) ppm. ^13^C{^1^H} NMR (125 MHz, CDCl_3_): δ 151.4, 150.3, 145.4, 137.7, 137.4, 134.5, 131.0, 128.9, 128.2, 126.3, 120.6, 118.0, 112.6, 105.1, 101.5, 82.9, 76.0, 69.9, 62.5, 55.8, 55.7, 34.7, 31.7, 25.8, 18.7, 18.0, 12.1, −4.7 ppm. HRMS (ESI) (*m*/*z*): [M + NH_4_]^+^ calcd for C_43_H_70_O_8_NSi_2_, 784.4634; found, 784.4629.

### *tert*-Butyl (4-((4*S*,5*R*)-5-(2,6-Dimethoxy-4-(3-((triisopropylsilyl)oxy)propyl)phenoxy)-2-phenyl-1,3-dioxan-4-yl)-2,6-dimethoxy phenoxy)dimethylsilane [(*S*,*R*)-**18ba**]

The product was prepared following the general Mitsunobu coupling procedure using (*S*,*S*)-**8b** (0.397 g, 0.889 mmol), **7a** (0.302 g, 0.820 mmol), DIAD (0.199 g, 0.986 mmol), and PPh_3_ (0.263 g, 1.002 mmol) in THF (8 mL) at room temperature. After workup, the residue was purified by column chromatography on silica gel (3 → 7% Et_2_O/pentane) to afford (*S*,*R*)-**18ba** as a colorless gel (0.389 g, 60% yield). It was visualized by TLC using a vanillin stain. ^1^H NMR (500 MHz, CDCl_3_): δ 7.55 (dd, *J* = 8.1, 1.7 Hz, 2H), 7.38–7.32 (m, 3H), 6.77 (s, 2H), 6.30 (s, 2H), 5.73 (s, 1H), 4.83 (d, *J* = 8.7 Hz, 1H), 4.47–4.41 (m, 2H), 4.11–4.05 (m, 1H), 3.76 (s, 6H), 3.71 (t, *J* = 6.3 Hz, 2H), 3.64 (s, 6H), 2.61 (t, *J* = 7.6 Hz, 2H), 1.85–1.78 (m, 2H), 1.12–1.07 (d + m, 21H), 1.03 (s, 9H), 0.13 (s, 6H). ^13^C{^1^H} NMR (125 MHz, CDCl_3_): δ 152.7, 151.0, 138.3, 137.9, 134.4, 133.4, 131.1, 128.8, 128.1, 126.3, 105.8, 105.4, 101.4, 83.4, 74.8, 70.1, 62.4, 55.8, 55.7, 34.5, 32.4, 25.8, 18.7, 18.0, 12.0, −4.6, −4.6 ppm. HRMS (ESI) (*m*/*z*): [M + NH_4_]^+^ calcd for C_44_H_72_O_9_NSi_2_, 814.4818; found, 814.4759.

### *tert*-Butyl(2,6-dimethoxy-4-((4*S*,5*R*)-5-(2-methoxy-4-(3-((triisopropylsilyl)oxy)propyl)phenoxy)-2-phenyl-1,3-dioxan-4-yl) Phenoxy)dimethylsilane [(*S*,*R*)-**18bb**]

The product was prepared following the general Mitsunobu coupling procedure using (*S*,*S*)-**8b** (0.509 g, 1.14 mmol), **7b** (0.350 g, 1.04 mmol), DIAD (0.289 g, 1.43 mmol), and PPh_3_ (0.326 g, 1.24 mmol) in THF (11 mL) at room temperature. After workup, the residue was purified by column chromatography on silica gel (2 → 5% Et_2_O/pentane) to afford (*S*,*R*)-**18bb** as a colorless gel (0.316 g, 40% yield). It was visualized by TLC using a vanillin stain. ^1^H NMR (500 MHz, CDCl_3_): δ 7.59 (dd, *J* = 7.5, 1.8 Hz, 2H), 7.43–7.37 (m, 3H), 6.79 (s, 2H), 6.68 (s, 1H), 6.54 (dd, *J* = 8.2, 2.4 Hz, 1H), 6.35 (d, *J* = 8.1 Hz, 1H), 5.77 (s, 1H), 4.86 (d, *J* = 9.2 Hz, 1H), 4.61 (dd, *J* = 11.1, 5.2 Hz, 1H), 4.25 (td, *J* = 9.6, 5.0 Hz, 1H), 4.04 (t, *J* = 10.6 Hz, 1H), 3.80 (s, 3H), 3.77 (s, 6H), 3.71 (t, *J* = 6.2 Hz, 2H), 2.62 (t, *J* = 7.7 Hz, 2H), 1.85–1.78 (m, 2H), 1.12–1.08 (d + m, 21H), 1.04 (s, 9H), 0.14 (s, 6H) ppm. ^13^C{^1^H} NMR (125 MHz, CDCl_3_): δ 151.4, 150.3, 145.4, 137.7, 137.4, 134.4, 131.0, 128.9, 128.2, 126.3, 120.6, 118.0, 112.6, 105.0, 101.5, 82.8, 76.0, 69.9, 62.5, 55.8, 55.7, 34.6, 31.7, 25.8, 18.7, 18.0, 12.0, −4.7 ppm. HRMS (ESI) (*m*/*z*): [M + NH_4_]^+^ calcd for C_43_H_70_O_8_NSi_2_, 784.4634; found, 784.4629.

### *tert*-Butyl (2-Methoxy-4-((4*R*,5*S*)-5-(2-methoxy-4-(3-((triisopropylsilyl)oxy)propyl)phenoxy)-2-phenyl-1,3-dioxan-4-yl)phenoxy) Dimethylsilane [(*R*,*S*)-**18cb**]

The product was prepared following the general Mitsunobu coupling procedure using (*R*,*R*)-**8c** (0.800 g, 1.92 mmol), **7b** (0.591 g, 1.75 mmol), DIAD (0.431 g, 2.13 mmol), and PPh_3_ (0.558 g, 2.13 mmol) in THF (18 mL) at room temperature. After workup, the residue was purified by column chromatography on silica gel (2 → 5% Et_2_O/pentane) to afford (*R*,*S*)-**18cb** as a colorless gel (0.550 g, 43% yield). It was visualized by TLC using a vanillin stain. ^1^H NMR (500 MHz, CDCl_3_): δ 7.57 (dd, *J* = 7.9, 1.8 Hz, 2H), 7.40–7.35 (m, 3H), 7.06–7.02 (m, 2H), 6.82 (d, *J* = 7.9 Hz, 1H), 6.64 (s, 1H), 6.50 (dd, *J* = 8.2, 2.1 Hz, 1H), 6.30 (d, *J* = 8.2 Hz, 1H), 5.74 (s, 1H), 4.85 (d, *J* = 9.2 Hz, 1H), 4.57 (dd, *J* = 11.0, 5.0 Hz, 1H), 4.21 (td, *J* = 9.9, 5.1 Hz, 1H), 4.01 (t, *J* = 10.5 Hz, 1H), 3.77 (s, 3H), 3.75 (s, 3H), 3.67 (t, *J* = 6.3 Hz, 2H), 2.58 (t, *J* = 7.8 Hz, 2H), 1.81–1.75 (m, 2H), 1.11–1.05 (d + m, 21H), 0.99 (s, 9H), 0.13 (s, 6H) ppm. ^13^C{^1^H} NMR (125 MHz, CDCl_3_): δ 150.7, 150.3, 145.3, 145.0, 137.7, 137.4, 132.1, 129.0, 128.2, 126.3, 120.6, 120.5, 120.1, 118.1, 112.5, 111.7, 101.5, 82.5, 76.2, 69.9, 62.4, 55.7, 55.5, 34.6, 31.7, 25.7, 18.4, 18.0, 12.0, −4.7 ppm. HRMS (ESI) (*m*/*z*): [M + NH_4_]^+^ calcd for C_42_H_68_O_7_NSi_2_, 754.4529; found, 754.4554.

### *tert*-Butyl (2-Methoxy-4-((4*S*,5*R*)-5-(2-methoxy-4-(3-((triisopropylsilyl)oxy)propyl)phenoxy)-2-phenyl-1,3-dioxan-4-yl)phenoxy) Dimethylsilane [(*S*,*R*)-**18db**]

The product was prepared following the general Mitsunobu coupling procedure using (*S*,*S*)-**8d** (0.704 g, 1.69 mmol), **7b** (0.522 g, 1.54 mmol), DIAD (0.372 g, 1.84 mmol), and PPh_3_ (0.483 g, 1.84 mmol) in THF (15 mL) at room temperature. After workup, the residue was purified by column chromatography on silica gel (2 → 5% Et_2_O/Pentane) to afford (*S*,*R*)-**18db** as a colorless gel (0.474 g, 42% yield). It was visualized by TLC using a vanillin stain. ^1^H NMR (500 MHz, CDCl_3_): δ 7.57 (dd, *J* = 8.0, 1.8 Hz, 2H), 7.41–7.35 (m, 3H), 7.08–7.03 (m, 2H), 6.82 (d, *J* = 7.9 Hz, 1H), 6.66 (s, 1H), 6.50 (dd, *J* = 8.2, 2.1 Hz, 1H), 6.30 (d, *J* = 8.2 Hz, 1H), 5.74 (s, 1H), 4.85 (d, *J* = 9.2 Hz, 1H), 4.57 (dd, *J* = 11.0, 5.0 Hz, 1H), 4.21 (td, *J* = 9.9, 5.0 Hz, 1H), 4.01 (t, *J* = 10.6 Hz, 1H), 3.77 (s, 3H), 3.75 (s, 3H), 3.67 (t, *J* = 6.3 Hz, 2H), 2.58 (t, *J* = 7.8 Hz, 2H), 1.83–1.77 (m, 2H), 1.10–1.06 (d + m, 21H), 0.99 (s, 9H), 0.13 (s, 6H) ppm. ^13^C{^1^H} NMR (125 MHz, CDCl_3_): δ 150.7, 150.4, 145.4, 145.1, 137.8, 137.4, 132.1, 128.9, 128.2, 126.3, 120.6, 120.5, 120.2, 118.3, 112.7, 111.9, 101.5, 82.6, 76.2, 69.9, 62.5, 55.7, 55.5, 34.6, 31.7, 25.7, 18.4, 18.0, 12.0, −4.7 ppm. HRMS (ESI) (*m*/*z*): [M + NH_4_]^+^ calcd for C_42_H_68_O_7_NSi_2_, 754.4529; found, 754.4553.

### General Global Benzylidene Acetal and Silyl Ether Deprotection Procedure

To a stirring solution of benzylidene acetal (1 equiv) and AcOH (1 drop) in 1,4-dioxane (0.1 M) under a N_2_ atmosphere was added 10% Pd/C (0.1 equiv). The N_2_ atmosphere was then exchanged for H_2_ and the reaction stirred under these conditions until complete consumption of the starting material, as monitored by TLC. The catalyst was then removed by filtration and the solvent removed under reduced pressure. The crude product was dissolved in anhydrous THF (0.1 M) and cooled to 0 °C under a N_2_ atmosphere. TBAF (1 M in THF, 2.2 equiv) was then added dropwise and the reaction was slowly allowed to warm to room temperature and stirred for 2 h. The reaction was then quenched by the addition of 1 M HCl and was extracted with CH_2_Cl_2_ (3×) and the organics were dried over Na_2_SO_4_, filtered, and concentrated in vacuo. The crude product was then purified by preparative TLC.

### (1*R*,2*S*)-1-(4-Hydroxy-3,5-dimethoxyphenyl)-2-(4-(3-hydroxypropyl)-2,6-dimethoxyphenoxy)propane-1,3-diol [(7*R*,8*S*)-**1**]

The product was prepared following the general global benzylidene acetal and silyl ether deprotection procedure using (7*R*,8*S*)-**18aa** (0.0421 g, 0.0528 mmol), AcOH (1 drop), and 10% Pd/C (4 mg) in 1,4-dioxane (1 mL) and then TBAF (0.12 mL) in THF (1 mL). After workup, the residue was purified by preparative TLC (7% MeOH/CH_2_Cl_2_) to afford (7*R*,8*S*)-**1** as a colorless gel (0.0198 g, 86% yield). ^1^H NMR (500 MHz, CDCl_3_): δ 6.58 (s, 2H), 6.48 (s, 2H), 4.98 (d, *J* = 3.8 Hz, 1H), 4.11–4.06 (m, 1H), 3.89–3.84 (s + m, 13H), 3.69 (t, *J* = 6.3 Hz, 2H), 3.45 (d, *J* = 12.1 Hz, 1H), 2.69 (t, *J* = 7.7 Hz, 2H), 1.93–1.87 (m, 2H) ppm. ^13^C{^1^H} NMR (125 MHz, CDCl_3_): δ 153.1, 147.0, 138.7, 133.8, 132.9, 130.4, 105.3, 102.5, 87.0, 72.55, 62.0, 60.6, 56.3, 56.1, 34.2, 32.6 ppm. HRMS (ESI) (*m*/*z*): [M – H]^+^ calcd for C_22_H_29_O_9_, 437.1817; found, 437.1815. All spectroscopic data were consistent with those previously reported.^7^

### (1*S*,2*R*)-1-(4-Hydroxy-3,5-dimethoxyphenyl)-2-(4-(3-hydroxypropyl)-2,6-dimethoxyphenoxy)propane-1,3-diol [(7*S*,8*R*)-**1**]

The product was prepared following the general global benzylidene acetal and silyl ether deprotection procedure using (7*S*,8*R*)-**18ba** (0.0425 g, 0.0533 mmol), AcOH (1 drop), and 10% Pd/C (4 mg) in 1,4-dioxane (1 mL) and then TBAF (0.12 mL) in THF (1 mL). After workup, the residue was purified by preparative TLC (7% MeOH/CH_2_Cl_2_) to afford (7*S*,8*R*)-**1** as a colorless gel (0.0202 g, 86% yield). ^1^H NMR (500 MHz, CDCl_3_): δ 6.58 (s, 2H), 6.48 (s, 2H), 4.98 (d, *J* = 3.8 Hz, 1H), 4.11–4.06 (m, 1H), 3.90–3.85 (s + m, 13H), 3.69 (t, *J* = 7.4 Hz, 2H), 3.46 (d, *J* = 12.7 Hz, 1H), 2.69 (t, *J* = 7.7 Hz, 2H), 1.93–1.86 (m, 2H) ppm. ^13^C{^1^H} NMR (125 MHz, CDCl_3_): δ 153.1, 147.0, 138.7, 133.8, 132.9, 130.4, 105.3, 102.5, 87.0, 72.5, 62.0, 60.6, 56.3, 56.1, 34.2, 32.6 ppm. HRMS (ESI) (*m*/*z*): [M – H]^+^ calcd for C_22_H_29_O_9_, 437.1817; found, 437.1817. All spectroscopic data were consistent with those previously reported.^7^

### (1*R*,2*S*)-1-(4-Hydroxy-3,5-dimethoxyphenyl)-2-(4-(3-hydroxypropyl)-2-methoxyphenoxy)propane-1,3-diol [(7*R*,8*S*)-**2**]

The product was prepared following the general global benzylidene acetal and silyl ether deprotection procedure using (7*R*,8*S*)-**18ab** (0.0473 g, 0.0617 mmol), AcOH (1 drop), and 10% Pd/C (5 mg) in 1,4-dioxane (1 mL) and then TBAF (0.13 mL) in THF (1 mL). After workup, the residue was purified by preparative TLC (7% MeOH/CH_2_Cl_2_) to afford (7*R*,8*S*)-**2** as a colorless gel (0.0201 g, 80% yield). ^1^H NMR (700 MHz, CDCl_3_): δ 6.86 (d, *J* = 8.1 Hz, 1H), 6.77 (d, *J* = 2.3 Hz, 1H), 6.74 (dd, *J* = 8.1, 2.3 Hz, 1H), 6.62 (s, 2H), 4.94 (d, *J* = 4.8 Hz, 1H), 4.10 (dt, *J* = 5.5, 3.0 Hz, 1H), 3.92–3.84 (dd + s + s, 10H), 3.68 (t, *J* = 6.4 Hz, 2H), 3.64 (dd, *J* = 12.2, 3.4 Hz, 1H), 2.68 (t, *J* = 7.7 Hz, 2H), 1.92–1.83 (m, 2H) ppm. ^13^C{^1^H} NMR (175 MHz, CDCl_3_): δ 151.3, 147.1, 144.8, 138.2, 134.1, 131.0, 121.3, 120.9, 112.3, 102.7, 87.5, 72.8, 62.1, 60.7, 56.3, 55.9, 34.2, 31.9 ppm. HRMS (ESI) (*m*/*z*): [M – H]^+^ calcd for C_21_H_27_O_8_, 407.1711; found, 407.1712. All spectroscopic data were consistent with those previously reported.^9^

### (1*S*,2*R*)-1-(4-Hydroxy-3,5-dimethoxyphenyl)-2-(4-(3-hydroxypropyl)-2-methoxyphenoxy)propane-1,3-diol [(7*S*,8*R*)-**2**]

The product was prepared following the general global benzylidene acetal and silyl ether deprotection procedure using (7*S*,8*R*)-**18bb** (0.0567 g, 0.0739 mmol), AcOH (1 drop), and 10% Pd/C (6 mg) in 1,4-dioxane (1 mL) and then TBAF (0.16 mL) in THF (1 mL). After workup, the residue was purified by preparative TLC (7% MeOH/CH_2_Cl_2_) to afford (7*S*,8*R*)-**2** as a colorless gel (0.0233 g, 77% yield). ^1^H NMR (700 MHz, CDCl_3_): δ 6.87 (d, *J* = 8.1 Hz, 1H), 6.77 (d, *J* = 2.3 Hz, 1H), 6.74 (dd, *J* = 8.1, 2.3 Hz, 1H), 6.62 (s, 2H), 4.94 (d, *J* = 4.8 Hz, 1H), 4.10 (dt, *J* = 5.7, 3.0 Hz, 1H), 3.91–3.87 (dd + s + s, 10H), 3.68 (t, *J* = 6.4 Hz, 2H), 3.64 (dd, *J* = 12.2, 3.5 Hz, 1H), 2.68 (t, *J* = 7.7 Hz, 2H), 1.91–1.86 (m, 2H) ppm. ^13^C{^1^H} NMR (175 MHz, CDCl_3_): δ 151.4, 147.1, 144.8, 138.3, 134.1, 130.9, 121.3, 121.0, 112.3, 102.7, 87.5, 72.8, 62.1, 60.7, 56.3, 55.9, 34.2, 31.9 ppm. HRMS (ESI) (*m*/*z*): [M – H]^+^ calcd for C_21_H_27_O_8_, 407.1711; found, 407.1712. All spectroscopic data were consistent with those previously reported.^7^

### (1*R*,2*S*)-1-(4-Hydroxy-3-methoxyphenyl)-2-(4-(3-hydroxypropyl)-2-methoxyphenoxy)propane-1,3-diol [(7*R*,8*S*)-**3**]

The product was prepared following the general global benzylidene acetal and silyl ether deprotection procedure using (7*R*,8*S*)-**18cb** (0.0392 g, 0.0532 mmol), AcOH (1 drop), and 10% Pd/C (4 mg) in 1,4-dioxane (1 mL) and then TBAF (0.12 mL) in THF (1 mL). After workup, the residue was purified by preparative TLC (7% MeOH/CH_2_Cl_2_) to afford (7*R*,8*S*)-**3** as a colorless gel (0.0157 g, 78% yield). ^1^H NMR (500 MHz, CDCl_3_): δ 6.97–6.95 (m, 1H), 6.88–6.80 (m, 3H), 6.76 (t, *J* = 2.4 Hz, 1H), 6.74–6.71 (m, 1H), 4.95 (d, *J* = 4.7 Hz, 1H), 4.12–4.09 (m, 1H), 3.90–3.86 (m, 7H), 3.69–3.64 (m, 2H), 2.68–2.64 (m, 2H), 1.95–1.79 (m, 2H) ppm. ^13^C{^1^H} NMR (125 MHz, CDCl_3_): δ 151.3, 146.6, 145.1, 144.9, 138.1, 131.9, 121.2, 120.7, 119.0, 114.3, 112.4, 108.8, 87.3, 72.7, 62.1, 60.8, 56.0, 55.9, 34.2, 31.8 ppm. HRMS (ESI) (*m*/*z*): [M – H]^+^ calcd for C_20_H_25_O_7_, 377.1606; found, 377.1606. All spectroscopic data were consistent with those previously reported.^6b^

### (1*S*,2*R*)-1-(4-Hydroxy-3-methoxyphenyl)-2-(4-(3-hydroxypropyl)-2-methoxyphenoxy)propane-1,3-diol [(7*S*,8*R*)-**3**]

The product was prepared following the general global benzylidene acetal and silyl ether deprotection procedure using (7*S*,8*R*)-**18db** (0.0367 g, 0.050 mmol), AcOH (1 drop), and 10% Pd/C (4 mg) in 1,4-dioxane (1 mL) and then TBAF (0.11 mL) in THF (1 mL). After workup, the residue was purified by preparative TLC (7% MeOH/CH_2_Cl_2_) to afford (7*S*,8*R*)-**3** as a colorless gel (0.0149 g, 79% yield). ^1^H NMR (500 MHz, CDCl_3_): δ 6.97–6.95 (m, 1H), 6.88–6.80 (m, 3H), 6.76 (t, *J* = 2.4 Hz, 1H), 6.74–6.71 (m, 1H), 4.95 (d, *J* = 4.7 Hz, 1H), 4.12–4.09 (m, 1H), 3.90–3.86 (m, 7H), 3.69–3.64 (m, 2H), 2.68–2.64 (m, 2H), 1.95–1.79 (m, 2H) ppm. ^13^C{^1^H} NMR (125 MHz, CDCl_3_): δ 151.3, 146.6, 145.1, 144.9, 138.1, 131.9, 121.3, 120.9, 119.0, 114.2, 112.4, 108.7, 87.4, 72.7, 62.1, 60.8, 56.0, 55.9, 34.2, 31.8 ppm. HRMS (ESI) (*m*/*z*): [M – H]^+^ calcd for C_20_H_25_O_7_, 377.1606; found, 377.1607. All spectroscopic data were consistent with those previously reported.^6b^

### General Benzylic Oxidation Procedure

To a stirring solution of benzylic alcohol (1 equiv) in anhydrous CH_2_Cl_2_ (0.02 M) under a N_2_ atmosphere at 0 °C was added activated MnO_2_ (5 equiv), and the reaction was allowed to slowly warm to room temperature. Upon complete consumption of starting material as monitored by TLC, the catalyst was removed by filtration, and the solution was concentrated in vacuo. The crude product was then purified by preparative TLC.

### (*S*)-3-Hydroxy-1-(4-hydroxy-3,5-dimethoxyphenyl)-2-(4-(3-hydroxypropyl)-2,6-dimethoxyphenoxy)propan-1-one (Asprenol B, **4**)

The product was prepared following the general benzylic oxidation procedure using (7*R*,8*S*)-**1** (0.0127 g, 0.0290 mmol) and MnO_2_ (0.0132 g, 0.1518 mmol) in CH_2_Cl_2_ (3 mL). After workup, the residue was purified by preparative TLC (7% MeOH/CH_2_Cl_2_) to afford Asprenol B [(*S*)-**4**], which was delivered as an off-white solid (0.0100 g, 78% yield, 98.7% ee). ^1^H NMR (500 MHz, CDCl_3_): δ 7.42 (s, 2H), 6.42 (s, 2H), 5.05 (dd, *J* = 7.4, 4.2 Hz, 1H), 4.00 (dd, *J* = 12.4, 7.1 Hz, 1H), 3.92 (s, 6H), 3.82 (dd, *J* = 12.1, 3.8 Hz, 1H), 3.72 (s, 6H), 3.68 (t, *J* = 5.7 Hz, 2H), 2.66 (t, *J* = 7.8 Hz, 2H), 1.92–1.81 (m, 2H). ^13^C{^1^H} NMR (125 MHz, CDCl_3_): δ 195.0, 152.4, 146.7, 139.9, 138.5, 134.5, 127.1, 106.4, 105.3, 87.3, 63.5, 62.1, 56.5, 55.9, 34.2, 32.5. HRMS (ESI) (*m*/*z*): [M + H]^+^ calcd for C_22_H_29_O_9_, 437.1806; found, 437.1801. HPLC (RegisReflectC-AmyloseA, EtOH/hexanes = 50/50, flow rate = 1.5 mL/min, l = 254 nm) *t*_1_ = 3.608 min (minor), *t*_2_ = 4.916 min (major). All spectroscopic data were consistent with those previously reported.^12^

### (*R*)-3-Hydroxy-1-(4-hydroxy-3,5-dimethoxyphenyl)-2-(4-(3-hydroxypropyl)-2,6-dimethoxyphenoxy)propan-1-one (*ent*-Asprenol B, **4′**)

The product was prepared following the general benzylic oxidation procedure using (7*S*,8*R*)-**1** (0.0153 g, 0.0349 mmol) and MnO_2_ (0.0155 g, 0.1783 mmol) in CH_2_Cl_2_ (3 mL). After workup, the residue was purified by preparative TLC (7% MeOH/CH_2_Cl_2_) to afford *ent*-Asprenol B (**4′**) as an off-white solid (0.0109 g, 72% yield, 99% ee). ^1^H NMR (500 MHz, CDCl_3_): δ 7.42 (s, 2H), 6.42 (s, 2H), 5.04 (dd, *J* = 7.5, 3.1 Hz, 1H), 4.00 (dd, *J* = 12.0, 7.6 Hz, 1H), 3.92 (s, 6H), 3.84–3.80 (m, 1H), 3.72 (s, 6H), 3.68 (t, *J* = 6.4 Hz, 2H), 2.66 (t, *J* = 7.8 Hz, 2H), 1.91–1.85 (m, 2H) ppm. ^13^C{^1^H} NMR (125 MHz, CDCl_3_): δ 195.0, 152.4, 146.7, 139.9, 138.5, 134.5, 127.1, 106.4, 105.3, 87.3, 63.5, 62.1, 56.5, 55.9, 34.2, 32.5 ppm. HRMS (ESI) (*m*/*z*): [M + H]^+^ calcd for C_22_H_29_O_9_, 437.1806; found, 437.1804. HPLC (RegisReflect C-AmyloseA, EtOH/hexanes = 50/50, flow rate = 1.5 mL/min, l = 254 nm) *t*_1_ = 3.600 min (major), *t*_2_ = 5.004 min (minor). All spectroscopic data were consistent with those previously reported.^12^

### (*S*)-3-Hydroxy-1-(4-hydroxy-3,5-dimethoxyphenyl)-2-(4-(3-hydroxypropyl)-2-methoxyphenoxy)propan-1-one [(*S*)-Icariol A_1_, (*S*)-**19**]

The product was prepared following the general benzylic oxidation procedure using (7*R*,8*S*)-**2** (0.0080 g, 0.0197 mmol) and MnO_2_ (0.0085 g, 0.0978 mmol) in CH_2_Cl_2_ (2 mL). After workup, the residue was purified by preparative TLC (7% MeOH/CH_2_Cl_2_) to afford (*S*)-icariol A_1_ [(*S*)-**19**] as an off-white solid (0.0050 g, 63% yield, 99.5% ee). ^1^H NMR (700 MHz, CDCl_3_): δ 7.41 (s, 2H), 6.81 (d, *J* = 8.2 Hz, 1H), 6.75 (d, *J* = 2.0 Hz, 1H), 6.65 (dd, *J* = 8.2, 2.0 Hz, 1H), 5.30 (dd, *J* = 6.6, 4.1 Hz, 1H), 4.12–4.00 (m, 2H), 3.92 (s, 6H), 3.85 (s, 3H), 3.66 (t, *J* = 6.4 Hz, 2H), 2.64 (t, *J* = 7.7 Hz, 2H), 1.88–1.82 (m, 2H) ppm. ^13^C NMR (175 MHz, CDCl_3_): δ 195.2, 150.2, 146.8, 144.9, 140.4, 137.6, 126.4, 120.7, 118.3, 112.5, 106.5, 84.8, 63.6, 62.2, 56.4, 55.8, 34.2, 31.8. HRMS (ESI) (*m*/*z*): [M + H]^+^ calcd for C_21_H_27_O_8_, 407.1700; found, 407.1697. HPLC (RegisReflect C-AmyloseA, EtOH/hexanes = 12.5/87.5, flow rate = 1.5 mL/min, l = 254 nm) *t*_1_ = 69.204 min (minor), *t*_2_ = 73.948 min (major). All spectroscopic data were consistent with those previously reported.^31^

### (*R*)-3-Hydroxy-1-(4-hydroxy-3,5-dimethoxyphenyl)-2-(4-(3-hydroxypropyl)-2-methoxyphenoxy)propan-1-one [(*R*)-Icariol A_1_, (*R*)-**19**]

The product was prepared following the general benzylic oxidation procedure using (*S*,*R*)-**2** (0.0050 g, 0.0211 mmol) and MnO_2_ (0.0053 g, 0.0610 mmol) in CH_2_Cl_2_ (1.2 mL). After workup, the residue was purified by preparative TLC (7% MeOH/CH_2_Cl_2_) to afford (*R*)-icariol A_1_ [(*R*)-**19**] as an off-white solid (0.0030 g, 60% yield, 98.1% ee). ^1^H NMR (700 MHz, CDCl_3_): δ 7.41 (s, 2H), 6.81 (d, *J* = 8.1 Hz, 1H), 6.75 (d, *J* = 2.0 Hz, 1H), 6.66 (dd, *J* = 8.2, 2.0 Hz, 1H), 5.29 (dd, *J* = 6.6, 4.1 Hz, 1H), 4.10–4.04 (m, 2H), 3.92 (s, 6H), 3.85 (s, 3H), 3.66 (t, *J* = 6.3 Hz, 2H), 2.64 (t, *J* = 7.7 Hz, 2H), 1.89–1.81 (m, 2H) ppm. ^13^C NMR (175 MHz, CDCl_3_): δ 195.2, 150.3, 146.8, 144.9, 140.4, 137.6, 126.4, 120.7, 118.4, 112.5, 106.5, 84.8, 63.6, 62.2, 56.4, 55.7, 34.2, 31.8 ppm. HRMS (ESI) (*m*/*z*): [M + H]^+^ calcd for C_21_H_27_O_8_, 407.1700; found, 407.1697. HPLC (RegisReflect C-AmyloseA, EtOH/hexanes = 12.5/87.5, flow rate = 1.5 mL/min, l = 254 nm) *t*_1_ = 67.328 min (major), *t*_2_ = 73.296 min (minor). All spectroscopic data were consistent with those previously reported.^31^

## Data Availability

The data underlying this study are available in the published article and its Supporting Information.

## Supporting Information Available

^1^H NMR spectra for known compounds, NMR (^1^H and ^13^C) spectra for all new compounds, NOESY spectra for compound **8b** and **18ab**, ^1^H spectra for (S)- and (R)-Mosher esters for **8b** and stereochemical analysis, and HPLC traces for enantioenriched triols and 7-keto-8,4′-oxyneolignan derivatives (PDF)
