# Supplementary material for: Modular Enantioselective Total Syntheses of the erythro-7,9-Dihydroxy- and 9-Hydroxy-7-Keto-8,4′-Oxyneolignans
Source: J Org Chem. 2024 Jul 3;89(14):9910–22. doi: 10.1021/acs.joc.4c00710 (PMC11267612; doi:10.1021/acs.joc.4c00710)
Supplement: Supplementary file 1 — jo4c00710_si_001.pdf [file jo4c00710_si_001.pdf]

Supporting Information for

Modular Enantioselective Total Syntheses of the *erythro*-7,9-Dihydroxy- and 9-Hydroxy-7-keto-8,4'-Oxyneolignans

Meghan C. Benda,[a] Caria Evans,[a,c,d] Shaoren Yuan,[a] Ian M. McClish,[b] William J. Berkey,[a,c,d] Hailey E. Areheart,[a] Emily S. Arnold,[a] Michelle L. Tang,[a] and Stefan France[a,c,d,*]

[a] School of Chemistry and Biochemistry, Georgia Institute of Technology, Atlanta, Georgia 30332, United States

[b] School of Biological Sciences, Georgia Institute of Technology, Atlanta, Georgia, 30332, United States

[c] Renewable Bioproducts Institute, Georgia Institute of Technology, Atlanta, Georgia 30332, United States

[d] Center for a Renewables-based Economy from WOOD (ReWOOD), Georgia Institute of Technology, Atlanta, Georgia, 30332, United States

stefan.france@chemistry.gatech.edu

Table of Contents

1. **Mosher Ester Analysis of Compound 8b**……S-4
2. **Attempted Approaches to 7,9-Dihydroxy- and 9-Hydroxy-7-keto-8,4'-oxyneolignans**
   Attempted Mitsunobu Reactions on 1-Aryl-2,3-dihydroxypropanone **I**……S-4
   Attempted Benzylic Oxidation of C-7 in Compound **VI**……S-5
   Attempted Mitsunobu Reactions on 7,9-Diprotected-7,8,9-trihydroxyarylpropanes……S-5

3. **Copies of NMR Spectra**
   ^{1}H NMR of Compound **14a**……S-6
   ^{13}C NMR of Compound **14a**……S-7
   ^{1}H NMR of Compound **14b**……S-8
   ^{13}C NMR of Compound **14b**……S-9
   ^{1}H NMR of Compound **11a**……S-10
   ^{13}C NMR of Compound **11a**……S-11
   ^{1}H NMR of Compound **11b**……S-12
   ^{13}C NMR of Compound **11b**……S-13
   ^{1}H NMR of Compound **15a**……S-14
   ^{13}C NMR of Compound **15a**……S-15
   ^{1}H NMR of Compound **15b**……S-16
   ^{13}C NMR of Compound **15b**……S-17
   ^{1}H NMR of Compound **16a**……S-18
   ^{13}C NMR of Compound **16a**……S-19

^{1}H NMR of Compound **16b**……S-20
^{13}C NMR of Compound **16b**……S-21
^{1}H NMR of Compound **7a**……S-22
^{13}C NMR of Compound **7a**……S-23
^{1}H NMR of Compound **7b**……S-24
^{13}C NMR of Compound **7b**……S-25
^{1}H NMR of Compound **10a**……S-26
^{13}C NMR of Compound **10a**……S-27
^{1}H NMR of Compound **10b**……S-28
^{13}C NMR of Compound **10b**……S-29
^{1}H NMR of Compound **9a**……S-30
^{13}C NMR of Compound **9a**……S-31
^{1}H NMR of Compound **9b**……S-32
^{13}C NMR of Compound **9b**……S-33
^{1}H NMR of Compound **9c**……S-34
^{13}C NMR of Compound **9c**……S-35
^{1}H NMR of Compound **9d**……S-36
^{13}C NMR of Compound **9d**……S-37
^{1}H NMR of Compound **8a**……S-38
^{13}C NMR of Compound **8a**……S-39
^{1}H NMR of Compound **8b**……S-40
^{13}C NMR of Compound **8b**……S-41
^{1}H NMR of (S)-Mosher ester of Compound **8b**……S-42
^{1}H NMR of (S)-Mosher ester of Compound **8b**……S-43
^{1}H NMR of (R)-Mosher ester of Compound **8b**……S-44
^{1}H NMR of (R)-Mosher ester of Compound **8b**……S-45
^{1}H NMR Overlay of (S)-Mosher ester (red) and (R)-Mosher ester (blue) of Compound **8b**……S-46
^{1}H NMR Overlay of (S)-Mosher ester (red) and (R)-Mosher ester (blue) of Compound **8b**……S-47
^{1}H NMR Overlay of (S)-Mosher ester (red) and (R)-Mosher ester (blue) of Compound **8b**……S-48
^{1}H NMR Overlay of (S)-Mosher ester (red) and (R)-Mosher ester (blue) of Compound **8b**……S-49
NOESY NMR of Compound **8b**……S-50
NOESY NMR of Compound **8b**……S-51
^{1}H NMR of Compound **8c**……S-52
^{13}C NMR of Compound **8c**……S-53
^{1}H NMR of Compound **8d**……S-54
^{13}C NMR of Compound **8d**……S-55
^{1}H NMR of Compound **18aa**……S-56
^{13}C NMR of Compound **18aa**……S-57
^{1}H NMR of Compound **18ab**……S-58
^{13}C NMR of Compound **18ab**……S-59

NOESY NMR of Compound **18ab**……S-60
NOESY NMR of Compound **18ab**……S-61
^{1}H NMR of Compound **18ba**……S-62
^{13}C NMR of Compound **18ba**……S-63
^{1}H NMR of Compound **18bb**……S-64
^{13}C NMR of Compound **18bb**……S-65
^{1}H NMR of Compound **18cb**……S-66
^{13}C NMR of Compound **18cb**……S-67
^{1}H NMR of Compound **18db**……S-68
^{13}C NMR of Compound **18db**……S-69
^{1}H NMR of Compound (*7S,8R*)**-1**……S-70
^{13}C NMR of Compound (*7S,8R*)**-1**……S-71
^{1}H NMR of Compound (*7R,8S*)**-1**……S-72
^{13}C NMR of Compound (*7R,8S*)**-1**……S-73
^{1}H NMR of Compound (*7S,8R*)**-2**……S-74
^{13}C NMR of Compound (*7S,8R*)**-2**……S-75
^{1}H NMR of Compound (*7R,8S*)**-2**……S-76
^{13}C NMR of Compound (*7R,8S*)**-2**……S-77
^{1}H NMR of Compound (*7S,8R*)**-3**……S-78
^{13}C NMR of Compound (*7S,8R*)**-3**……S-79
^{1}H NMR of Compound (*7R,8S*)**-3**……S-80
^{13}C NMR of Compound (*7R,8S*)**-3**……S-81
^{1}H NMR of Compound Asprenol B (**4**)……S-82
^{13}C NMR of Compound Asprenol B (**4**)……S-83
^{1}H NMR of Compound *ent*-Asprenol B (**4'**)……S-84
^{13}C NMR of Compound *ent*-Asprenol B (**4'**)……S-85
^{1}H NMR of Compound *(S)*-Icariol A_1 [(*S*)-**19**]……S-86
^{13}C NMR of Compound *(S)*-Icariol A_1 [(*S*)-**19**]……S-87
^{1}H NMR of Compound *(R)*-Icariol A_1 [(*R*)-**19**]……S-88
^{13}C NMR of Compound *(R)*-Icariol A_1 [*R*)-**19**]……S-89

4. HPLC data

A. Compounds **9a** and **9b**……S-90
B. Compounds **9c** and **9d**……S-93
C. Asprenol B (**4**) and *ent*-Asprenol B (**4'**)……S-96
D. (*S*) and (*R*)-Icariol A_1 (**19**)……S-99

1. Mosher Ester Analysis of Compound 8b

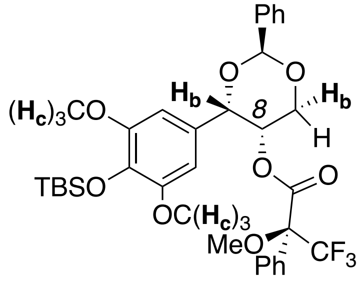

(S)-Mosher ester of **8b**

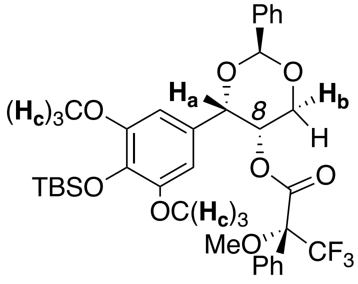

(R)-Mosher ester of **8b**

^{1}H Signal	(S)-ester	(R)-ester	Δδ (δS-δR)
**H_a**	4.86	4.85	+0.01
	4.84	4.84	0.00
**H_b**	4.53	4.58	-0.05
	4.52	4.57	-0.05
	4.51	4.56	-0.05
	4.50	4.55	-0.05
**H_c**	3.755	3.750	+0.05

Based on shifts, the configuration of at C(8) = *S.* This outcome is in agreement with the expected outcome from the Sharpless asymmetric dihydroxylation with AD-mix-α.

2. Attempted Approaches to 7,9-Dihydroxy- and 9-Hydroxy-7-keto-8,4'-oxyneolignans

See Benda, M. C. A Study into the Design, Synthesis, and Application of Asymmetric Catalyst Systems. Ph.D. Dissertation, Georgia Institute of Technology, Atlanta, GA, 2023.

Scheme S1. Attempted Mitsunobu reactions on 1-aryl-2,3-dihydroxypropanones **I**

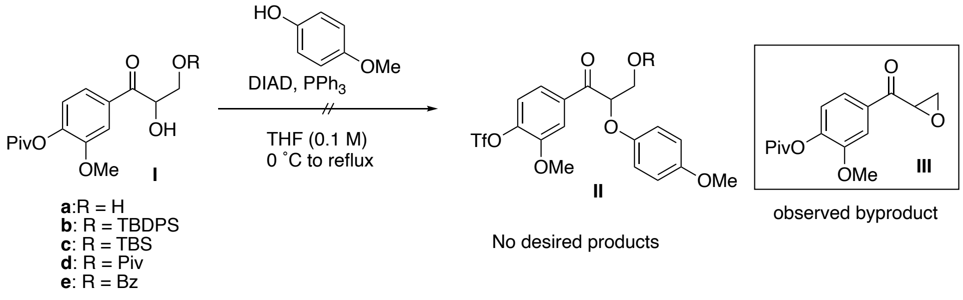

Scheme S2. Attempted Benzylic Oxidation of C-7 in Compound **VI**

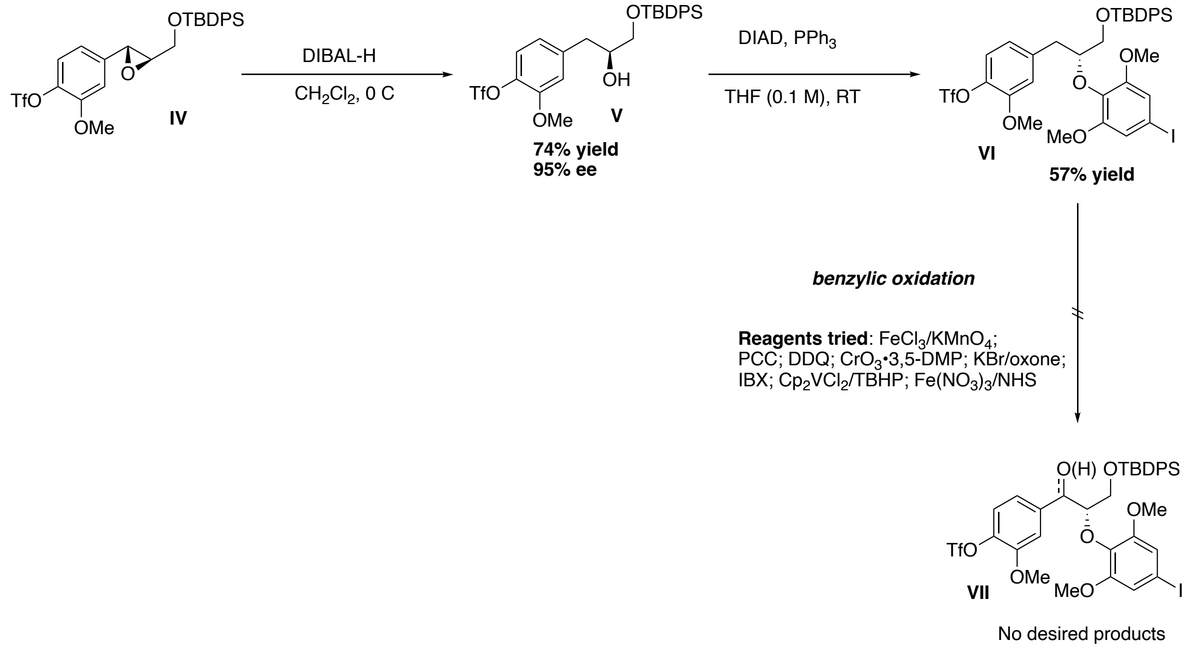

Scheme S3. Attempted Mitsunobu Reactions on 7,9-Diprotected-7,8,9-trihydroxyarylpropanes

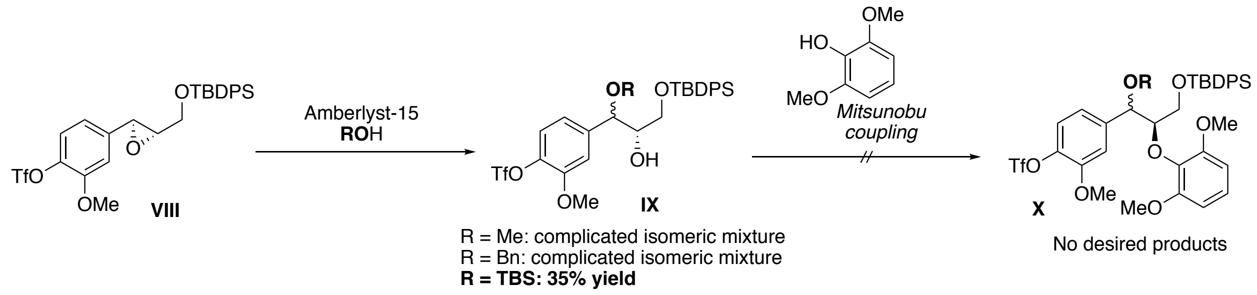

3. Copies of NMR Spectra

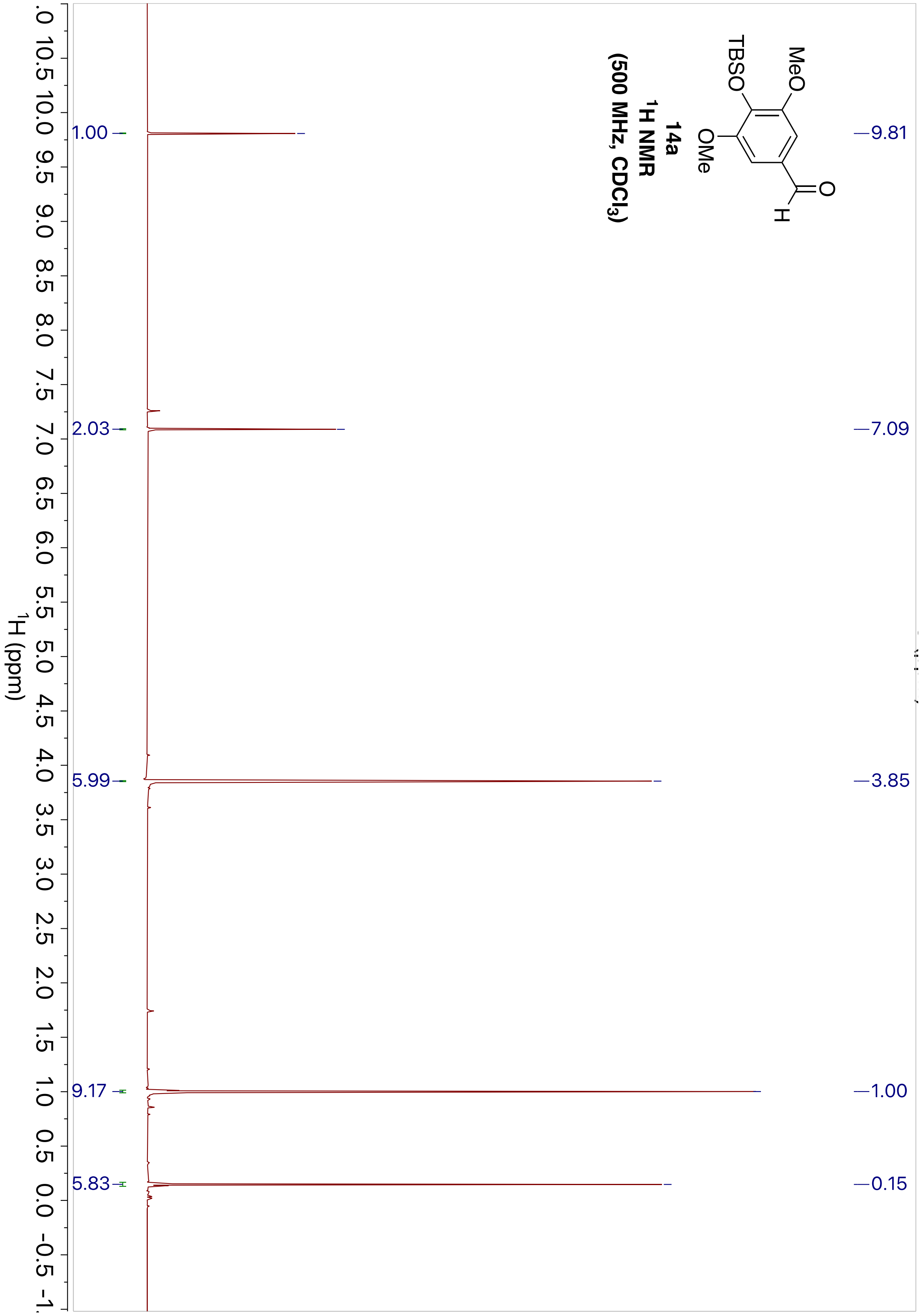
MeO
TBSO
OMe
O
H
14a
^{1}H NMR
(500 MHz, $CDCl_3$)
9.81
7.09
3.85
1.00
0.15
1.00
2.03
5.99
9.17
5.83
^{1}H (ppm)

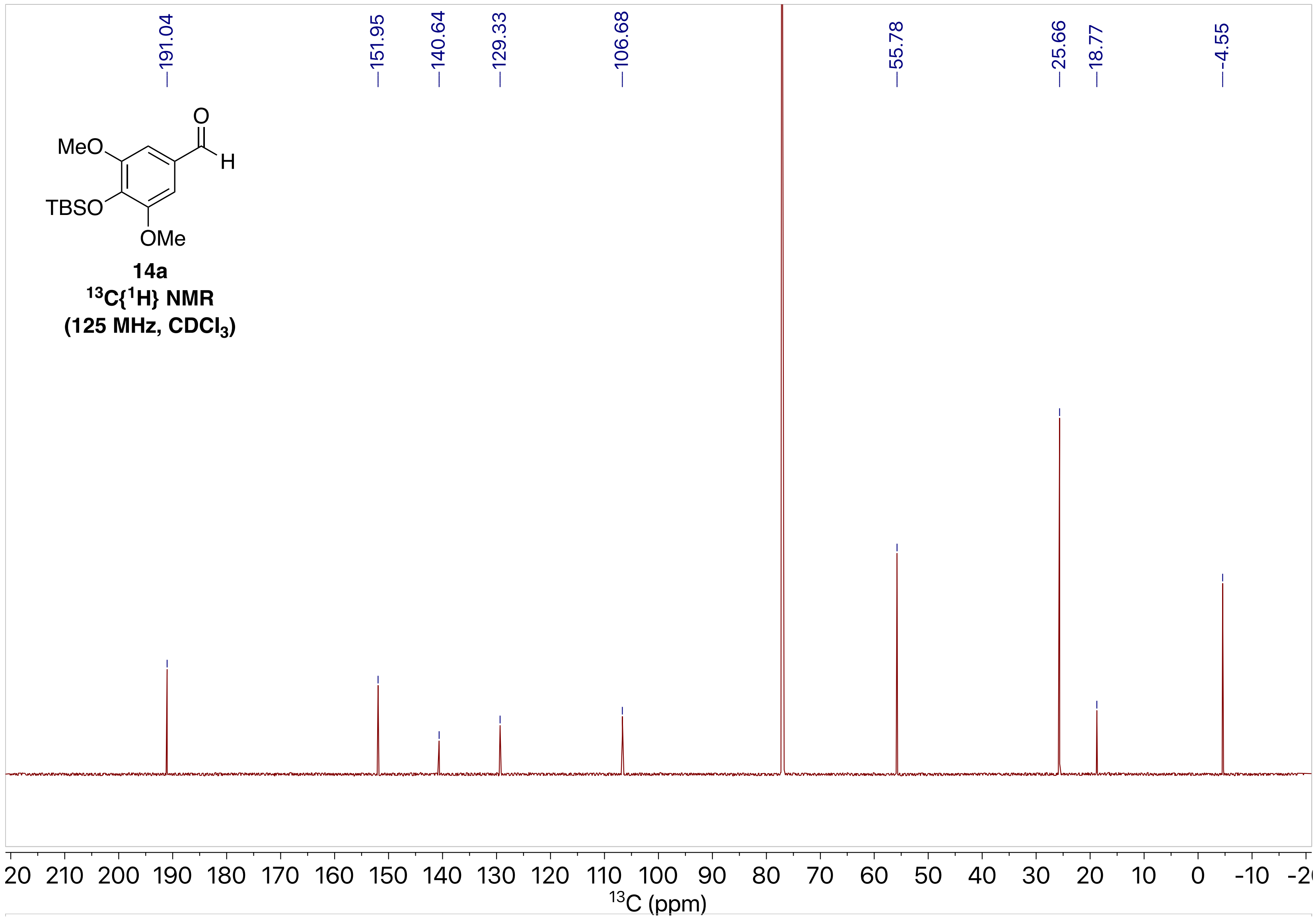

MeO
TBSO
OMe
O
H
14a
$^{13}C\{^1H\}$ NMR
(125 MHz, $CDCl_3$)
191.04
151.95
140.64
129.33
106.68
55.78
25.66
18.77
-4.55
20 210 200 190 180 170 160 150 140 130 120 110 100 90 80 70 60 50 40 30 20 10 0 -10 -2
^{13}C (ppm)

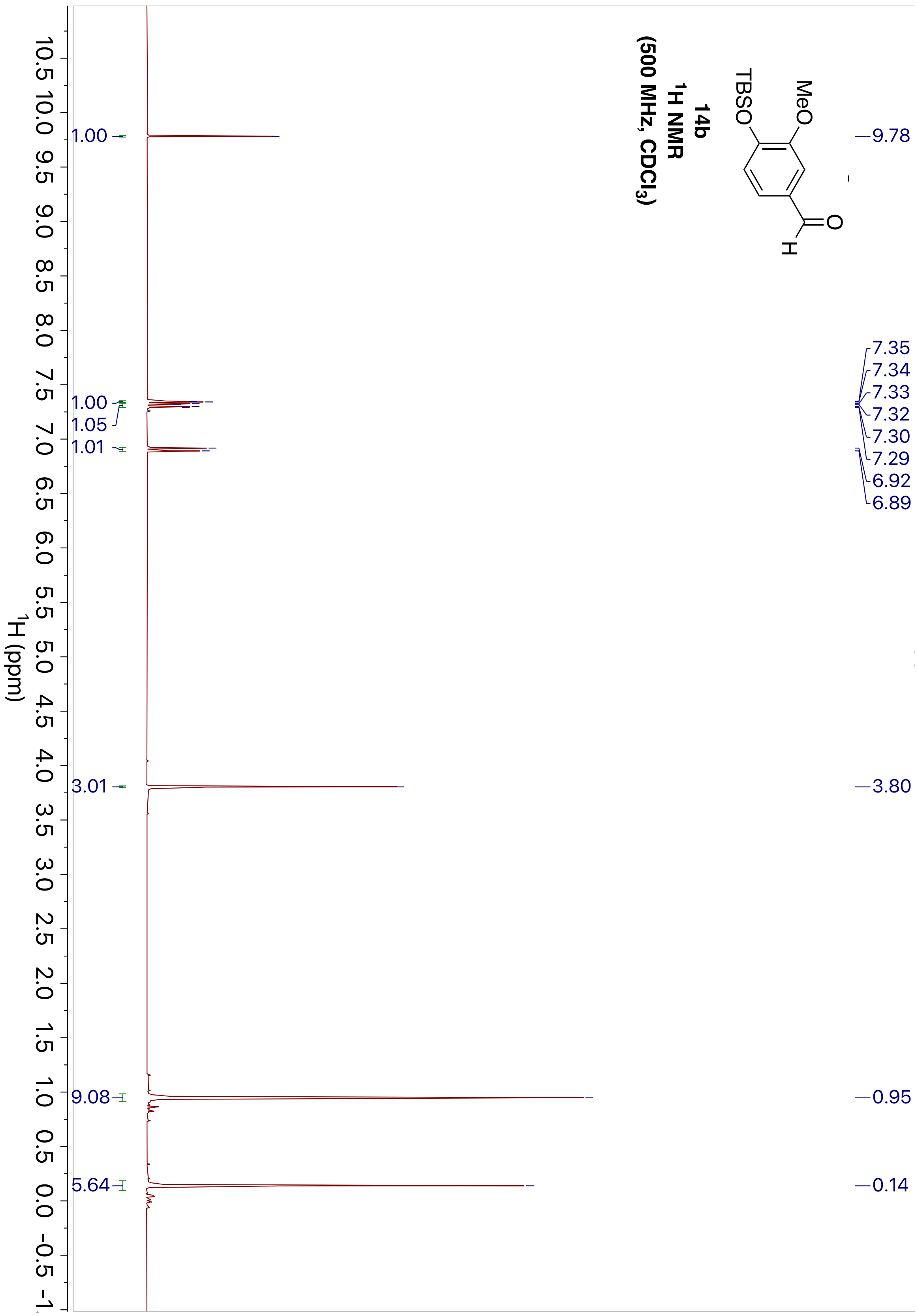
MeO
TBSO
O
H
14b
1H NMR
(500 MHz, CDCl3)
9.78
7.35
7.34
7.33
7.32
7.30
7.29
6.92
6.89
3.80
0.95
0.14
1.00
1.00
1.05
1.01
3.01
9.08
5.64
1H (ppm)

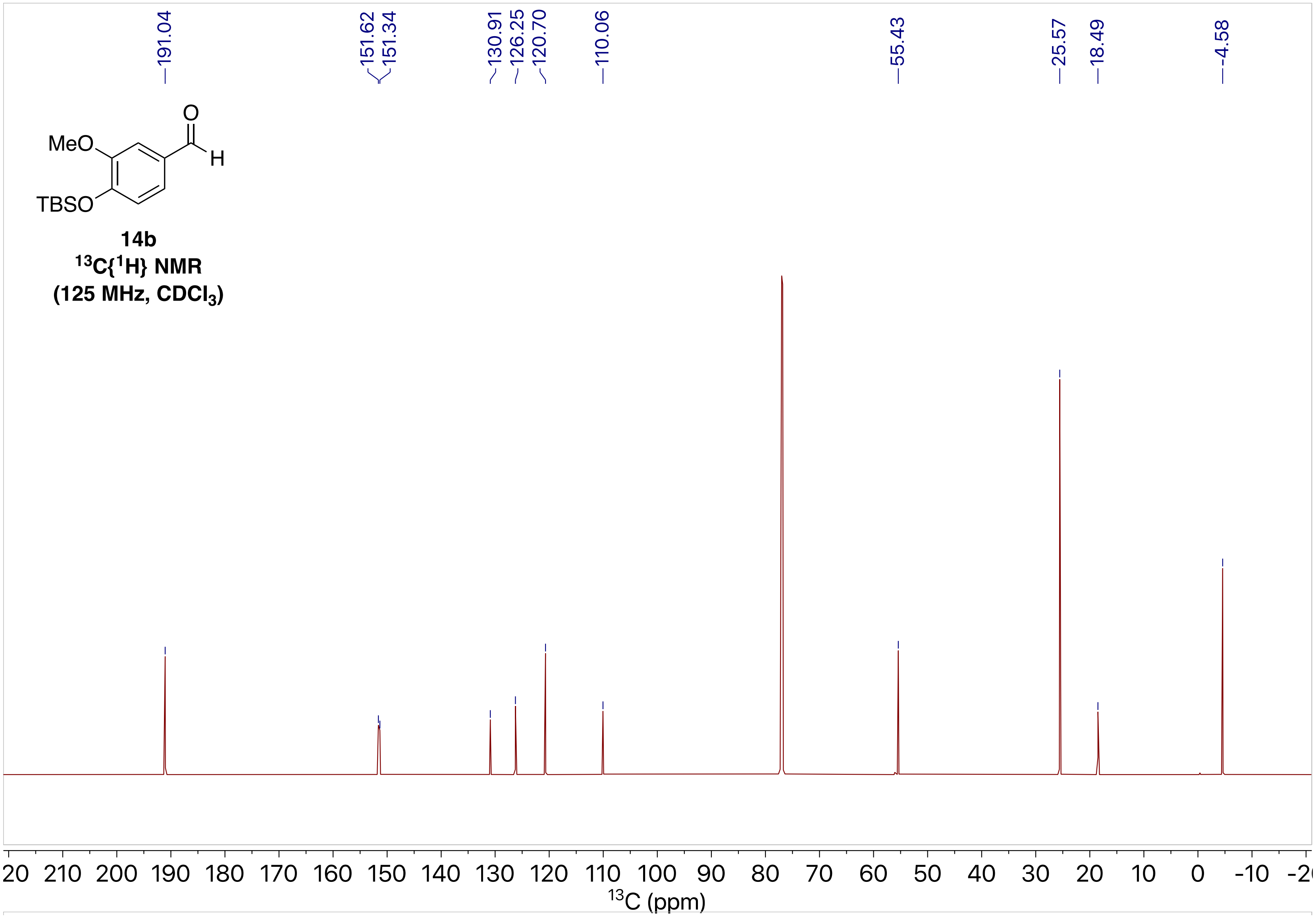
191.04
151.62
151.34
130.91
126.25
120.70
110.06
55.43
25.57
18.49
-4.58
MeO
TBSO
O
H
14b
13C{1H} NMR
(125 MHz, CDCl3)
20 210 200 190 180 170 160 150 140 130 120 110 100 90 80 70 60 50 40 30 20 10 0 -10
13C (ppm)

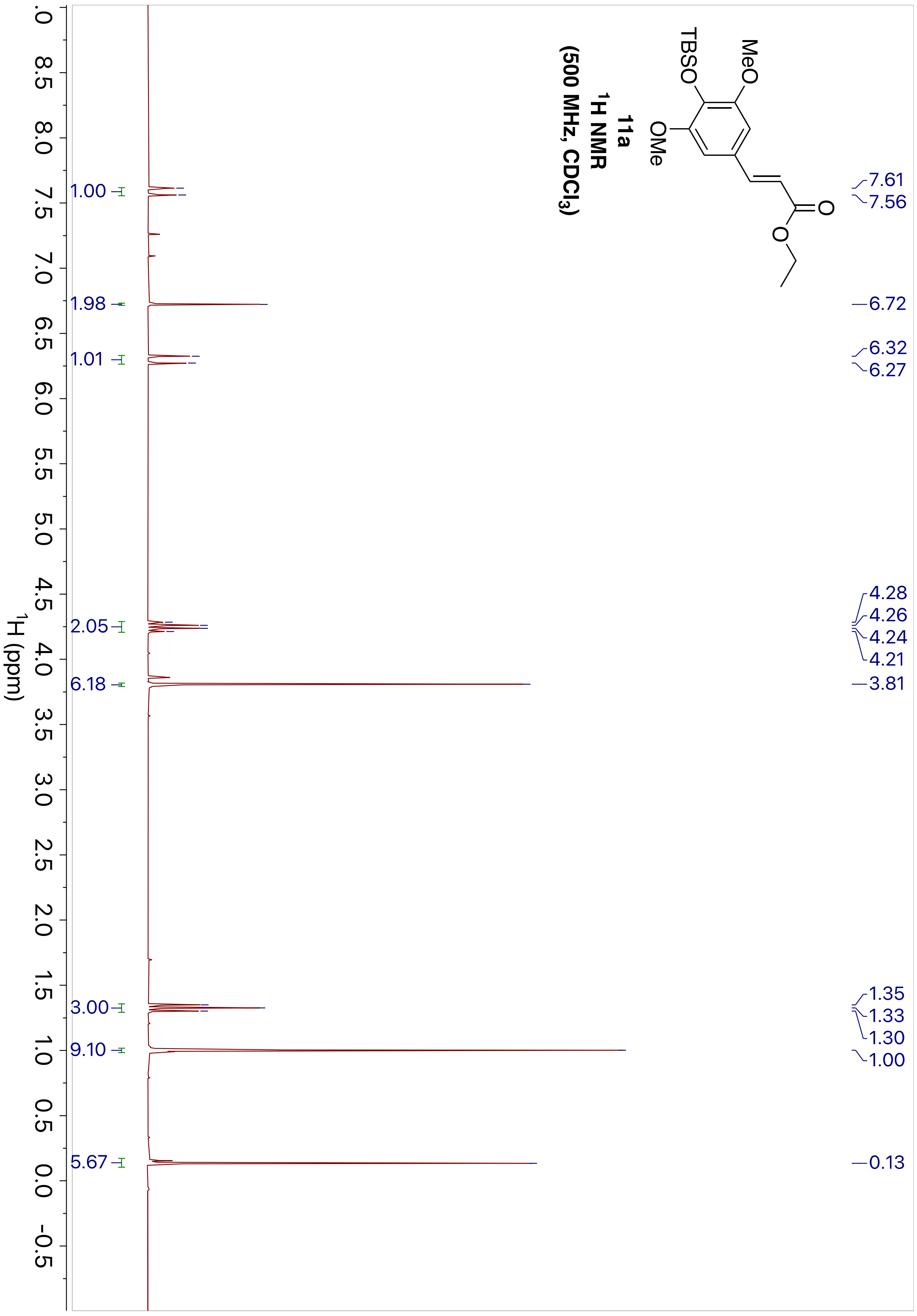

MeO
TBSO
OMe
O
O
11a
1H NMR
(500 MHz, CDCl3)
7.61
7.56
6.72
6.32
6.27
4.28
4.26
4.24
4.21
3.81
1.35
1.33
1.30
1.00
0.13
1.00
1.98
1.01
2.05
6.18
3.00
9.10
5.67
1H (ppm)

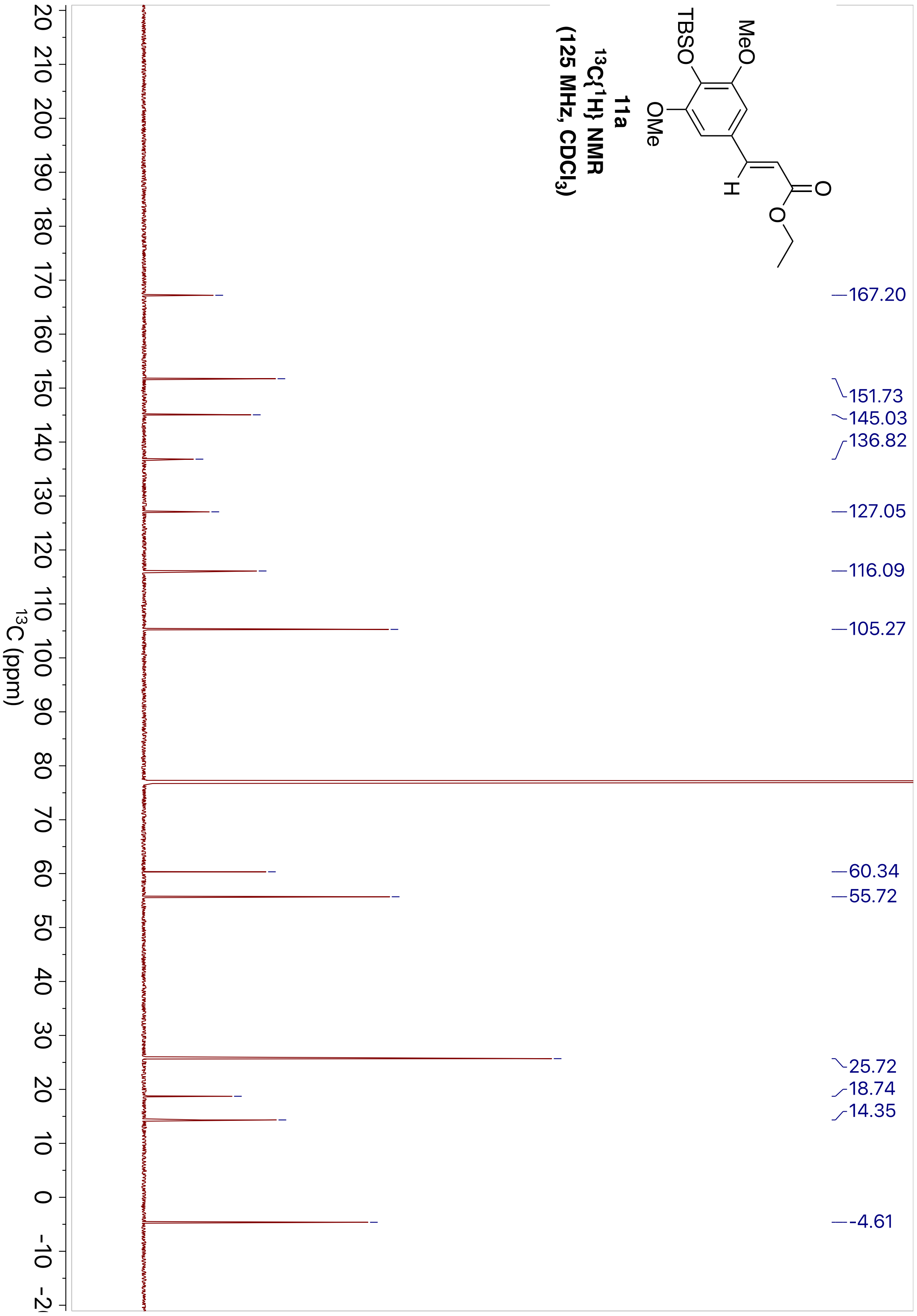
MeO
TBSO
OMe
H
O
O
11a
$^{13}C\{^{1}H\}$ NMR
(125 MHz, $CDCl_3$)
167.20
151.73
145.03
136.82
127.05
116.09
105.27
60.34
55.72
25.72
18.74
14.35
-4.61
^{13}C (ppm)

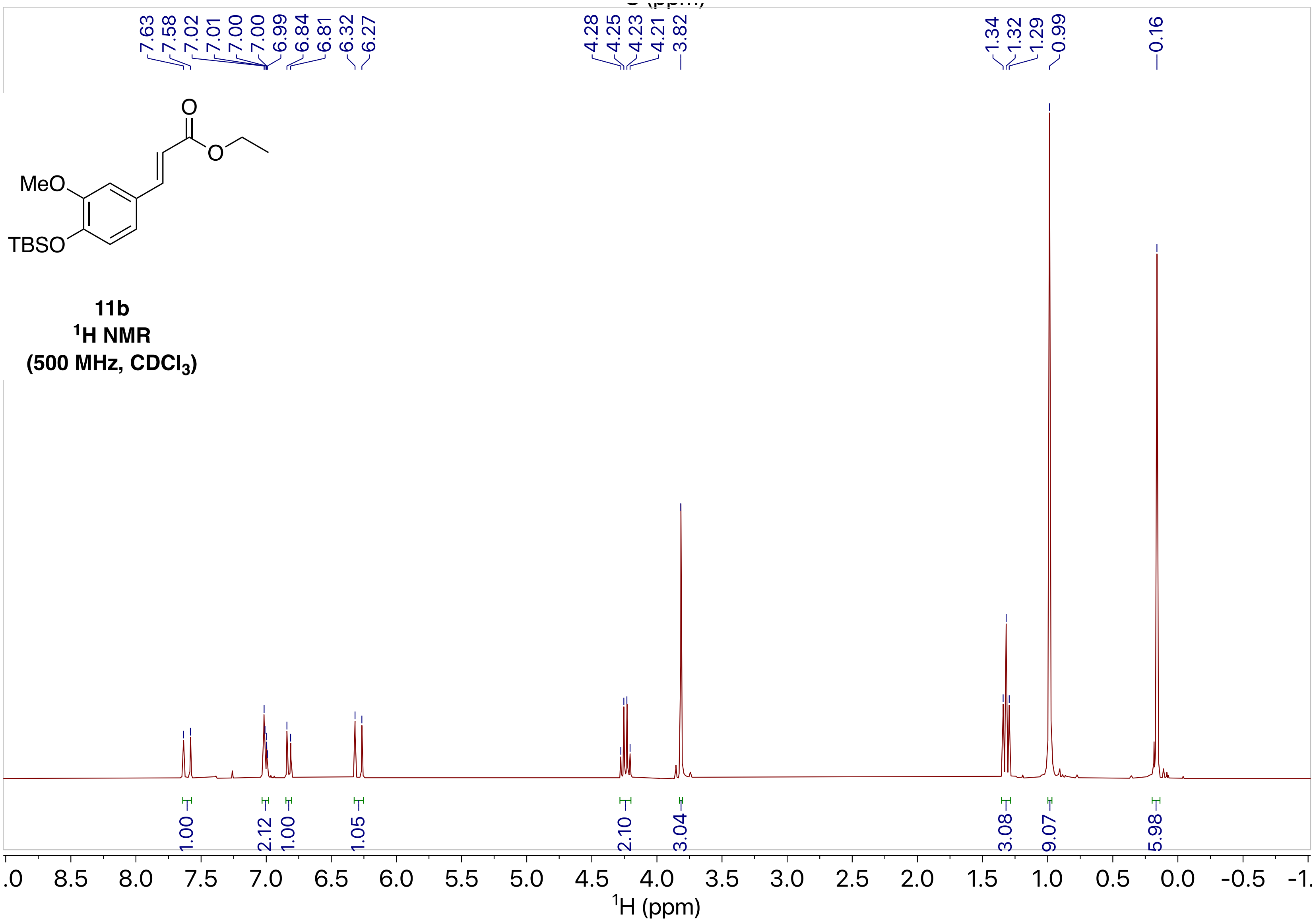

11b
^{1}H NMR
(500 MHz, $CDCl_3$)

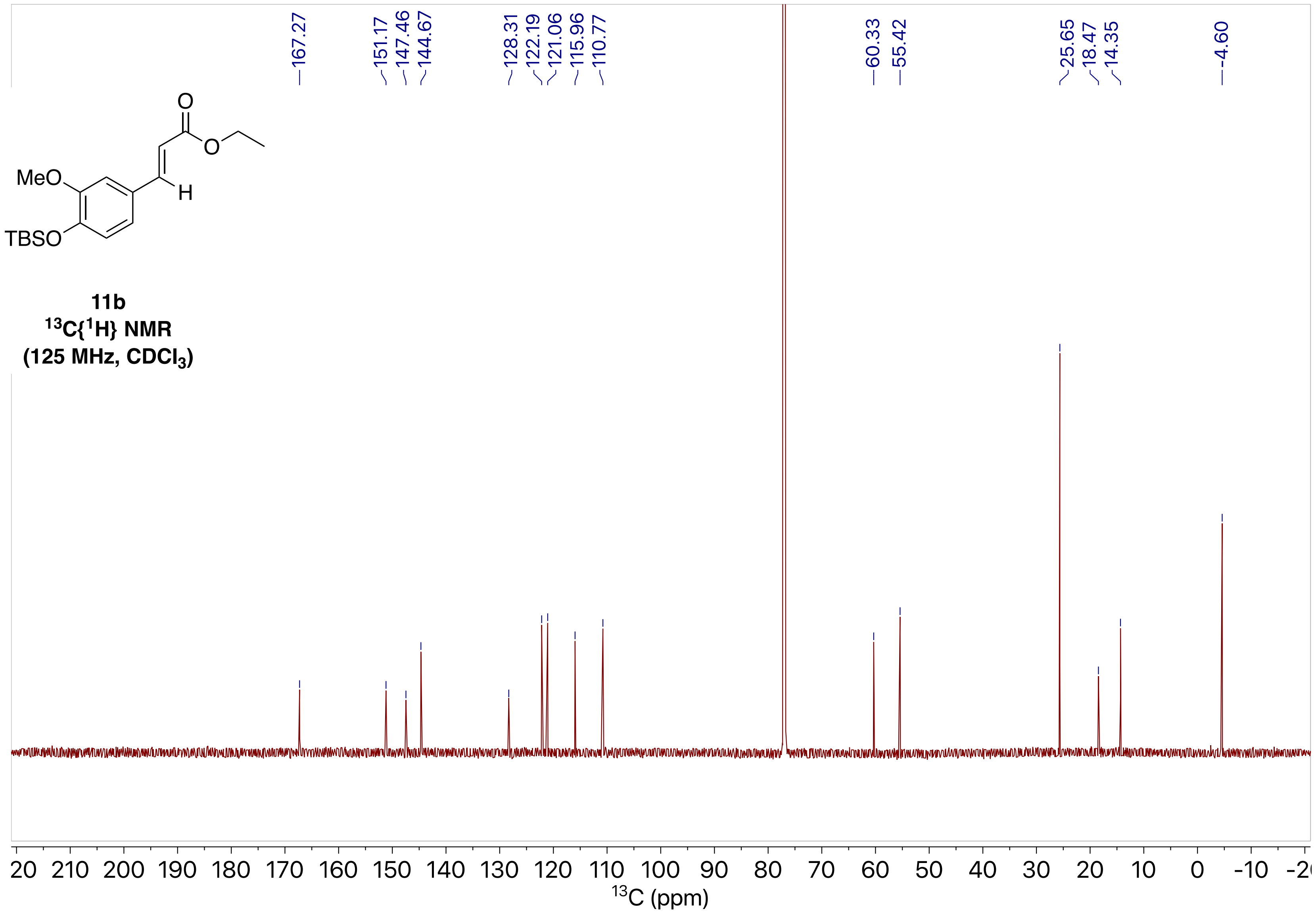

11b
$^{13}C\{^{1}H\}$ NMR
(125 MHz, $CDCl_3$)

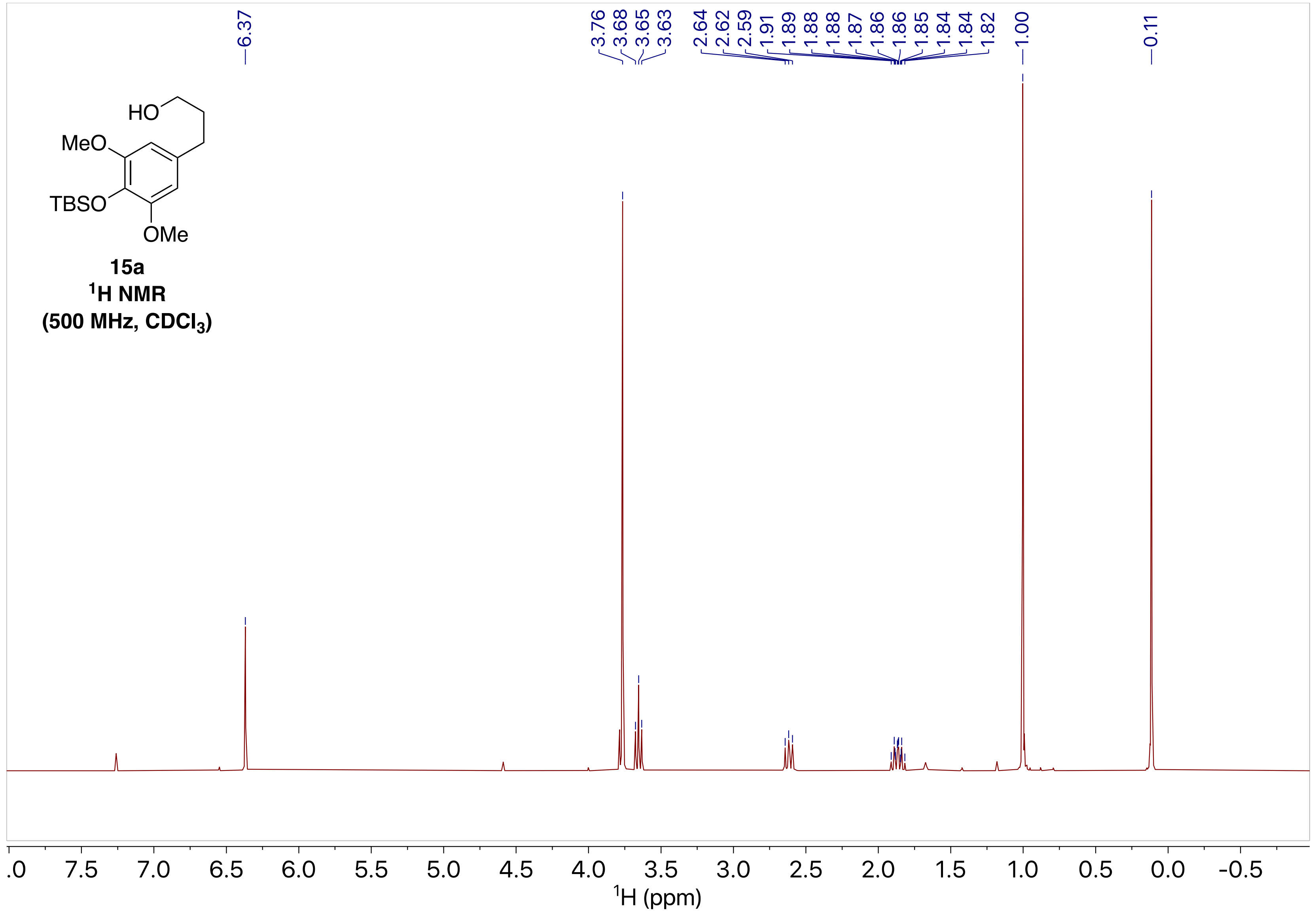

HO
MeO
TBSO
OMe
15a
1H NMR
(500 MHz, CDCl3)
6.37
3.76
3.68
3.65
3.63
2.64
2.62
2.59
1.91
1.89
1.88
1.88
1.87
1.86
1.86
1.85
1.84
1.84
1.82
1.00
0.11
.0 7.5 7.0 6.5 6.0 5.5 5.0 4.5 4.0 3.5 3.0 2.5 2.0 1.5 1.0 0.5 0.0 -0.5
1H (ppm)

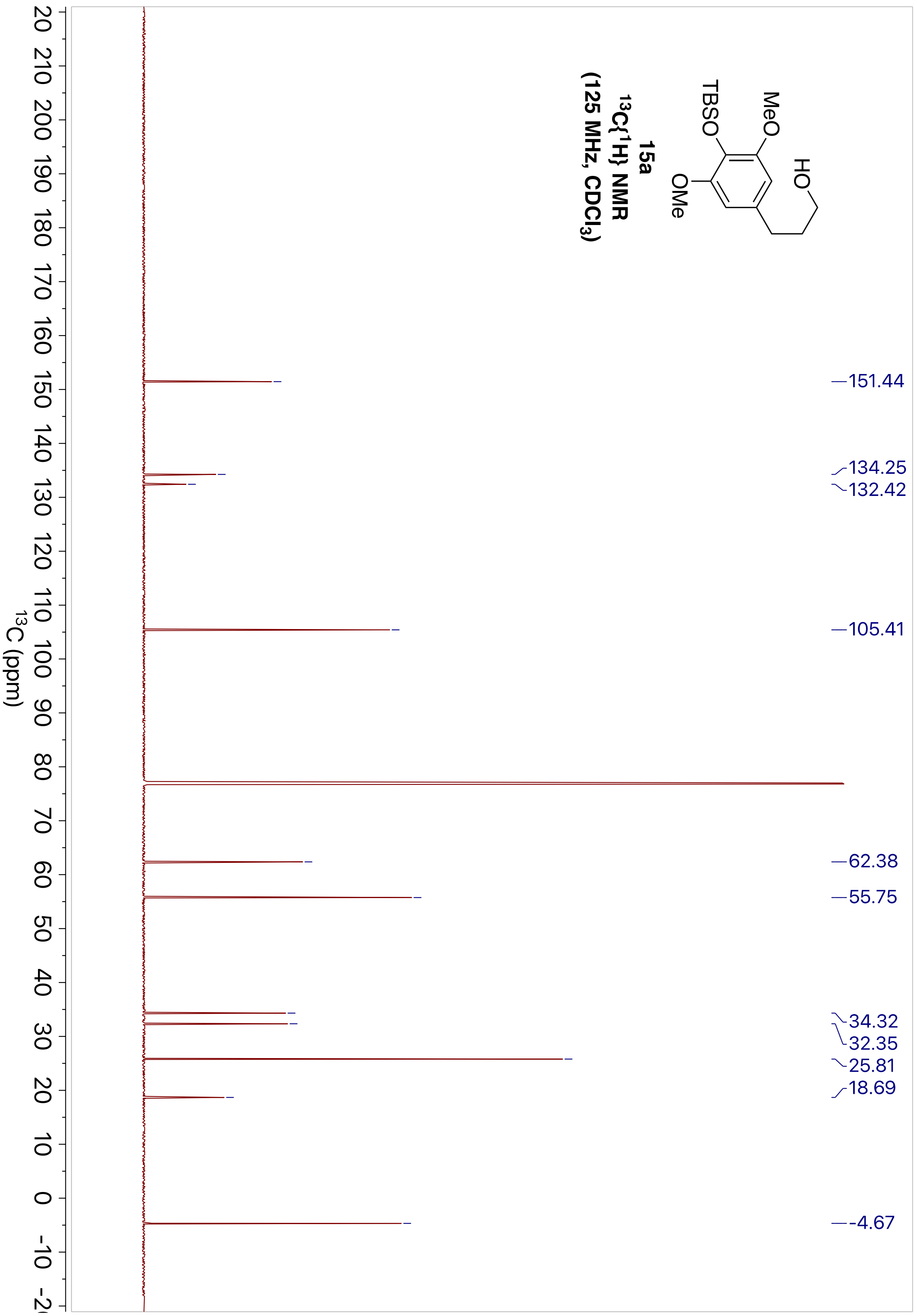

15a
$^{13}C\{^1H\}$ NMR
(125 MHz, $CDCl_3$)
MeO
HO
TBSO
OMe
151.44
134.25
132.42
105.41
62.38
55.75
34.32
32.35
25.81
18.69
-4.67
^{13}C (ppm)

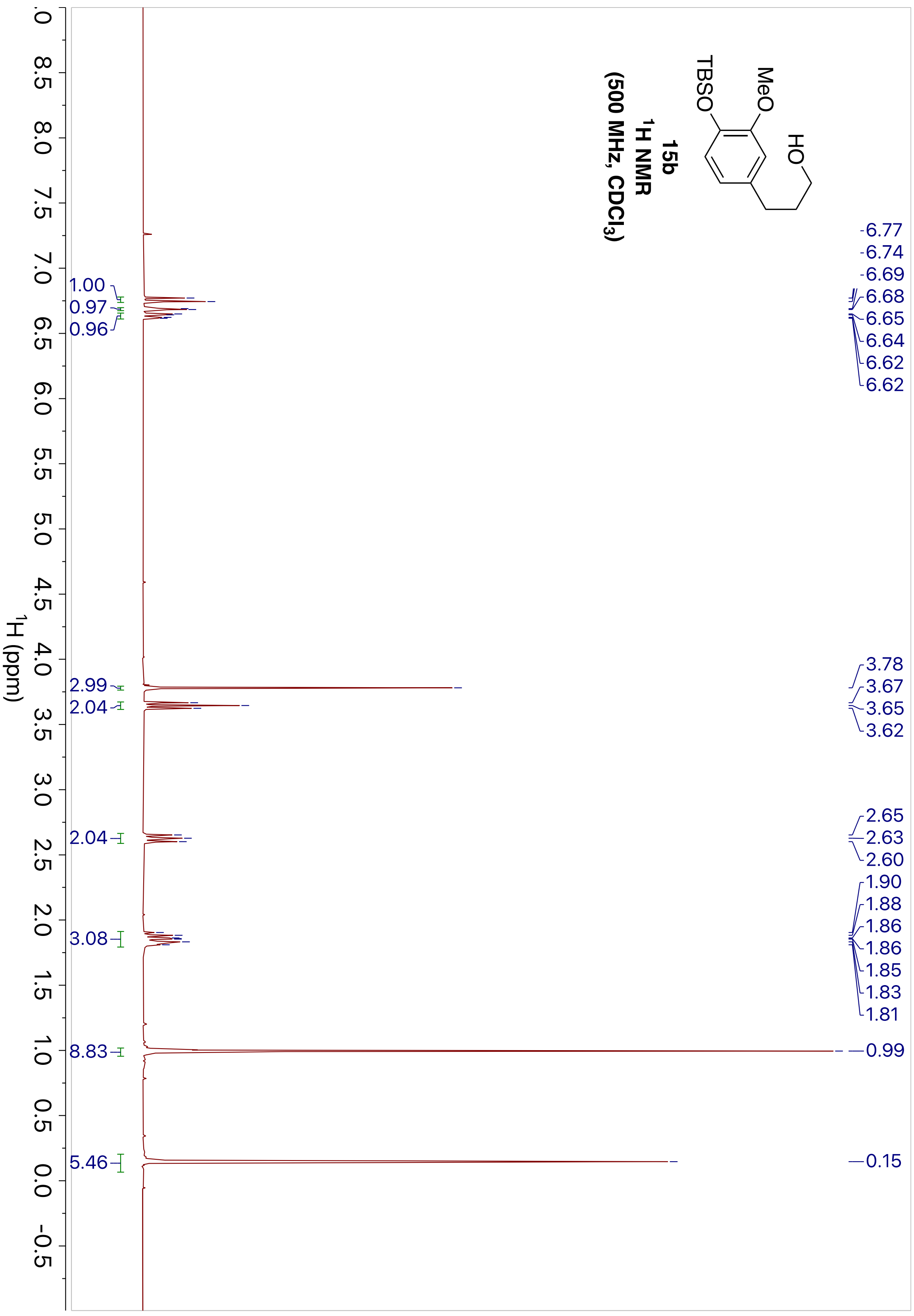
MeO
HO
TBSO
15b
1H NMR
(500 MHz, CDCl3)
1H (ppm)

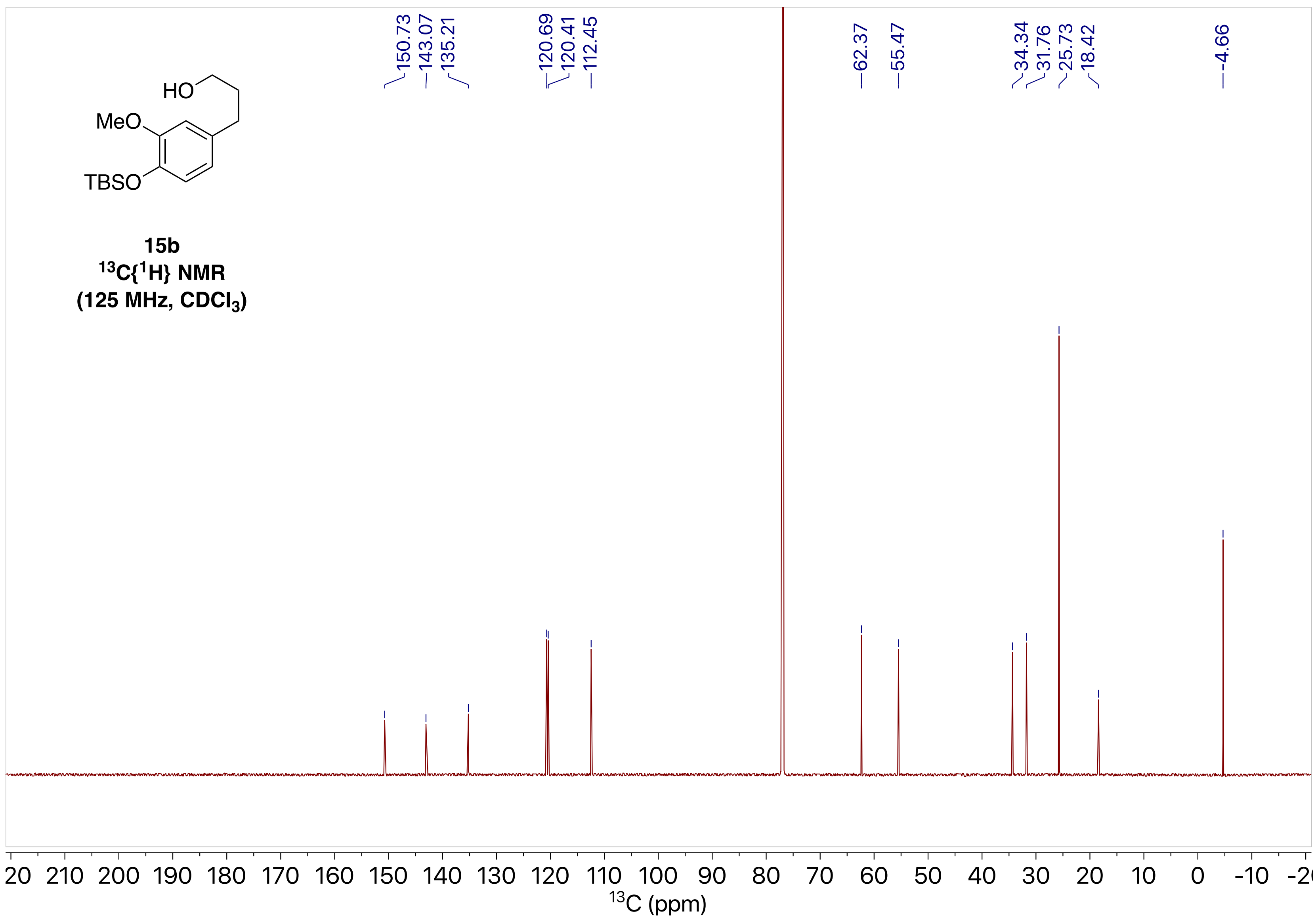
HO
MeO
TBSO
15b
13C{1H} NMR
(125 MHz, CDCl3)
150.73
143.07
135.21
120.69
120.41
112.45
62.37
55.47
34.34
31.76
25.73
18.42
-4.66
20 210 200 190 180 170 160 150 140 130 120 110 100 90 80 70 60 50 40 30 20 10 0 -10
13C (ppm)

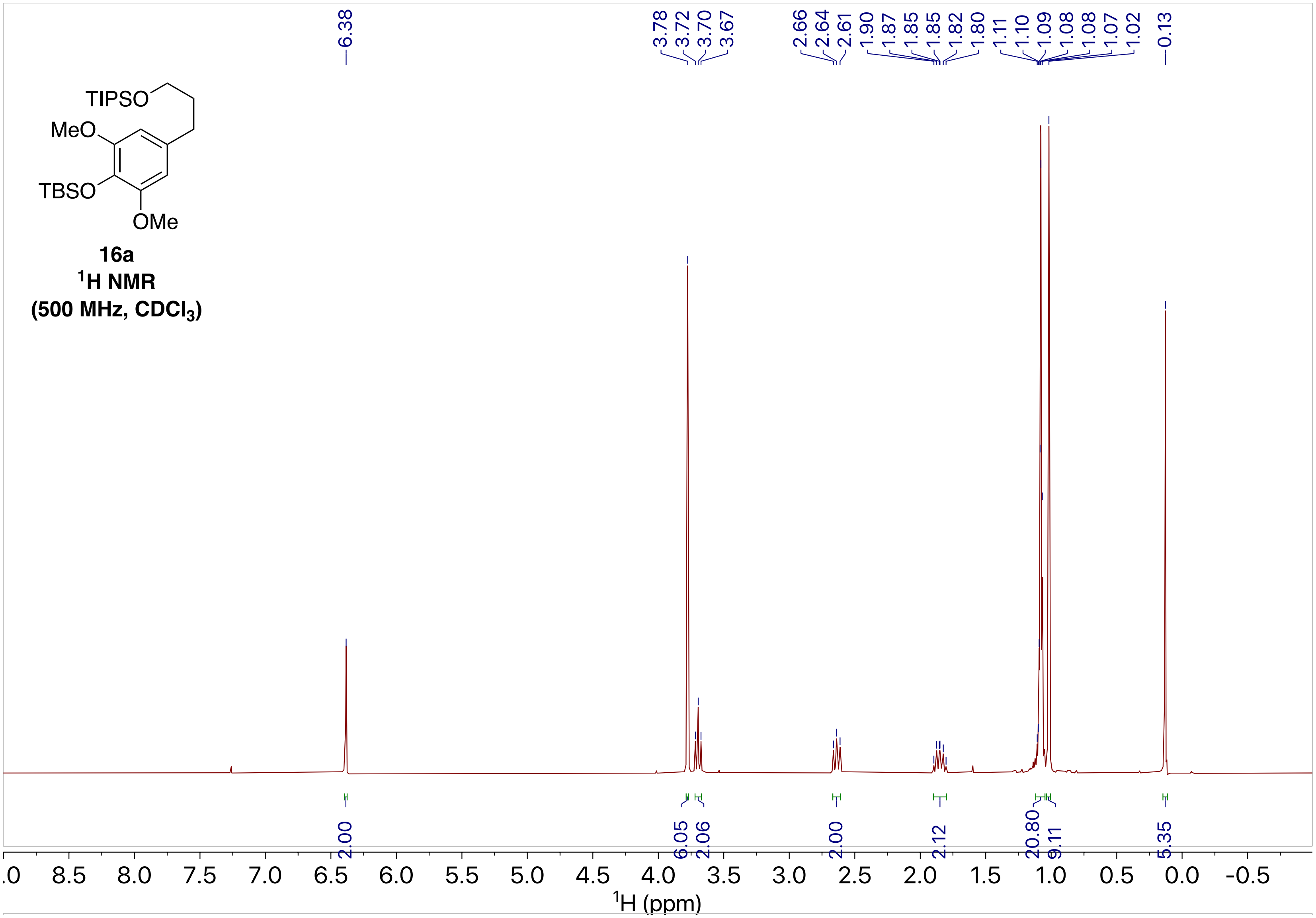
TIPSO
MeO
TBSO
OMe
16a
1H NMR
(500 MHz, CDCl3)
6.38
3.78
3.72
3.70
3.67
2.66
2.64
2.61
1.90
1.87
1.85
1.85
1.82
1.80
1.11
1.10
1.09
1.08
1.08
1.07
1.02
0.13
2.00
6.05
2.06
2.00
2.12
20.80
9.11
5.35
.0 8.5 8.0 7.5 7.0 6.5 6.0 5.5 5.0 4.5 4.0 3.5 3.0 2.5 2.0 1.5 1.0 0.5 0.0 -0.5
1H (ppm)

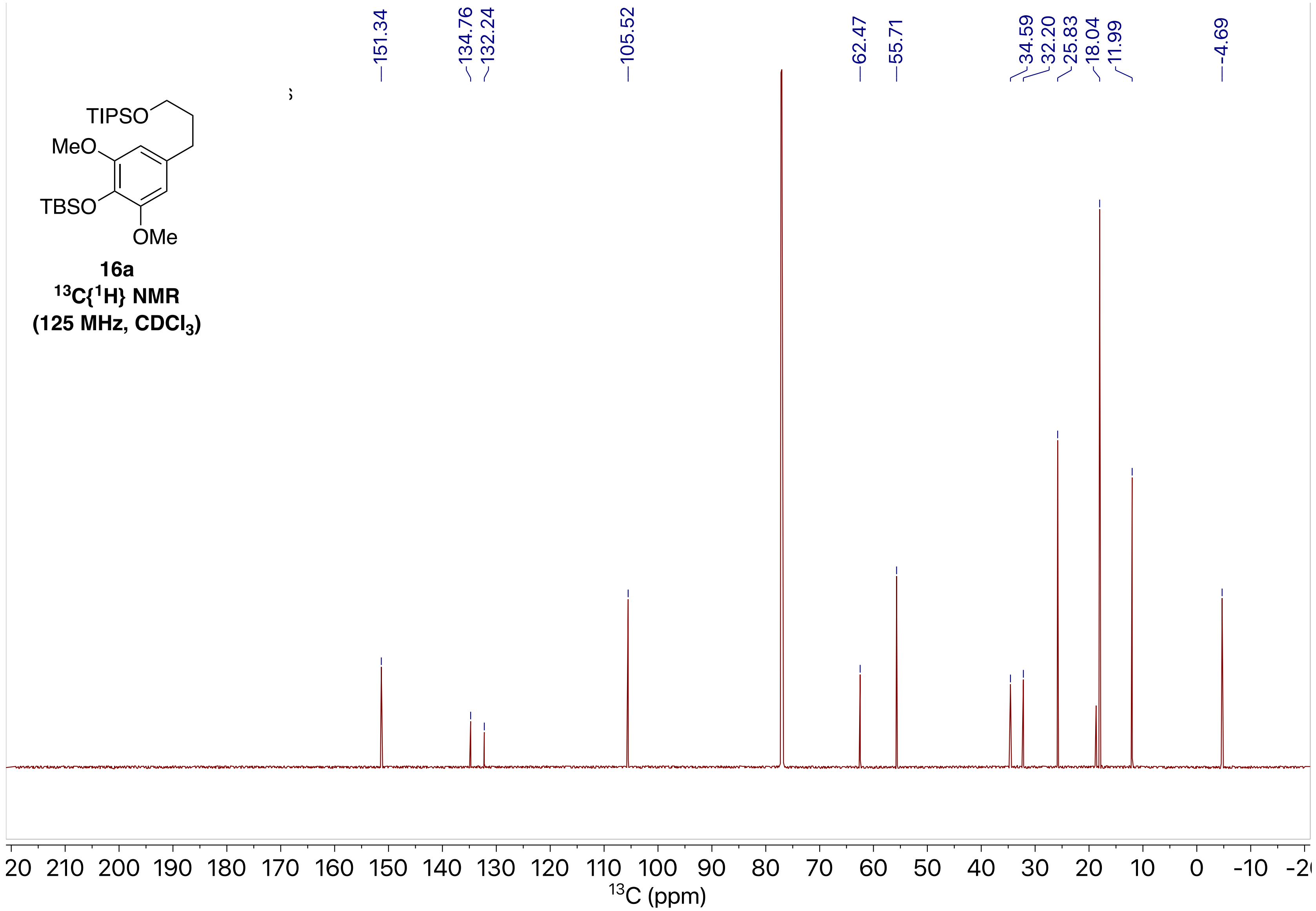
TIPSO
MeO
TBSO
OMe
16a
$^{13}C\{^{1}H\}$ NMR
(125 MHz, $CDCl_3$)
151.34
134.76
132.24
105.52
62.47
55.71
34.59
32.20
25.83
18.04
11.99
-4.69
20 210 200 190 180 170 160 150 140 130 120 110 100 90 80 70 60 50 40 30 20 10 0 -10
^{13}C (ppm)

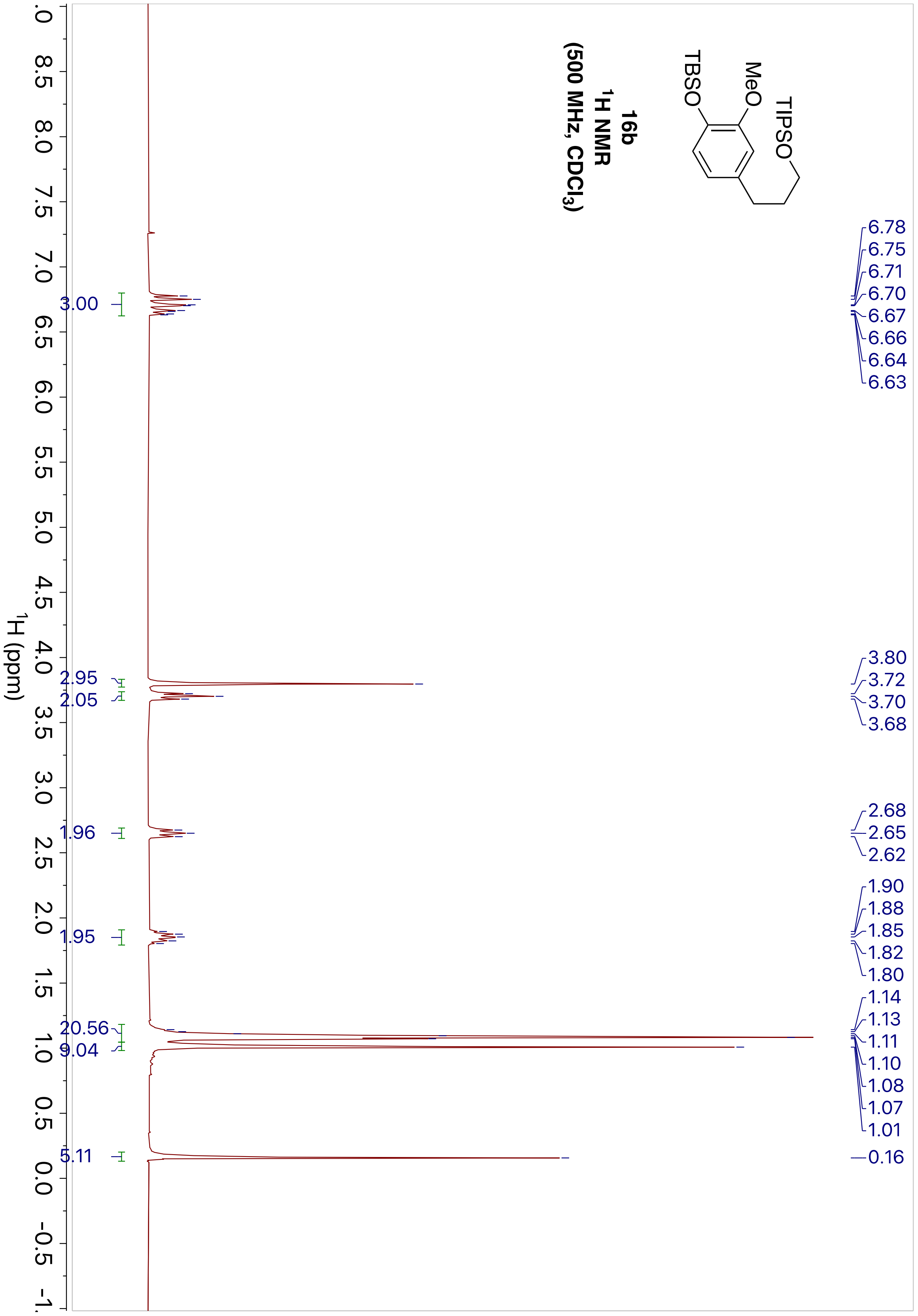
TIPSO
MeO
TBSO
16b
1H NMR
(500 MHz, CDCl3)
6.78
6.75
6.71
6.70
6.67
6.66
6.64
6.63
3.80
3.72
3.70
3.68
2.68
2.65
2.62
1.90
1.88
1.85
1.82
1.80
1.14
1.13
1.11
1.10
1.08
1.07
1.01
0.16
3.00
2.95
2.05
1.96
1.95
20.56
9.04
5.11
1H (ppm)

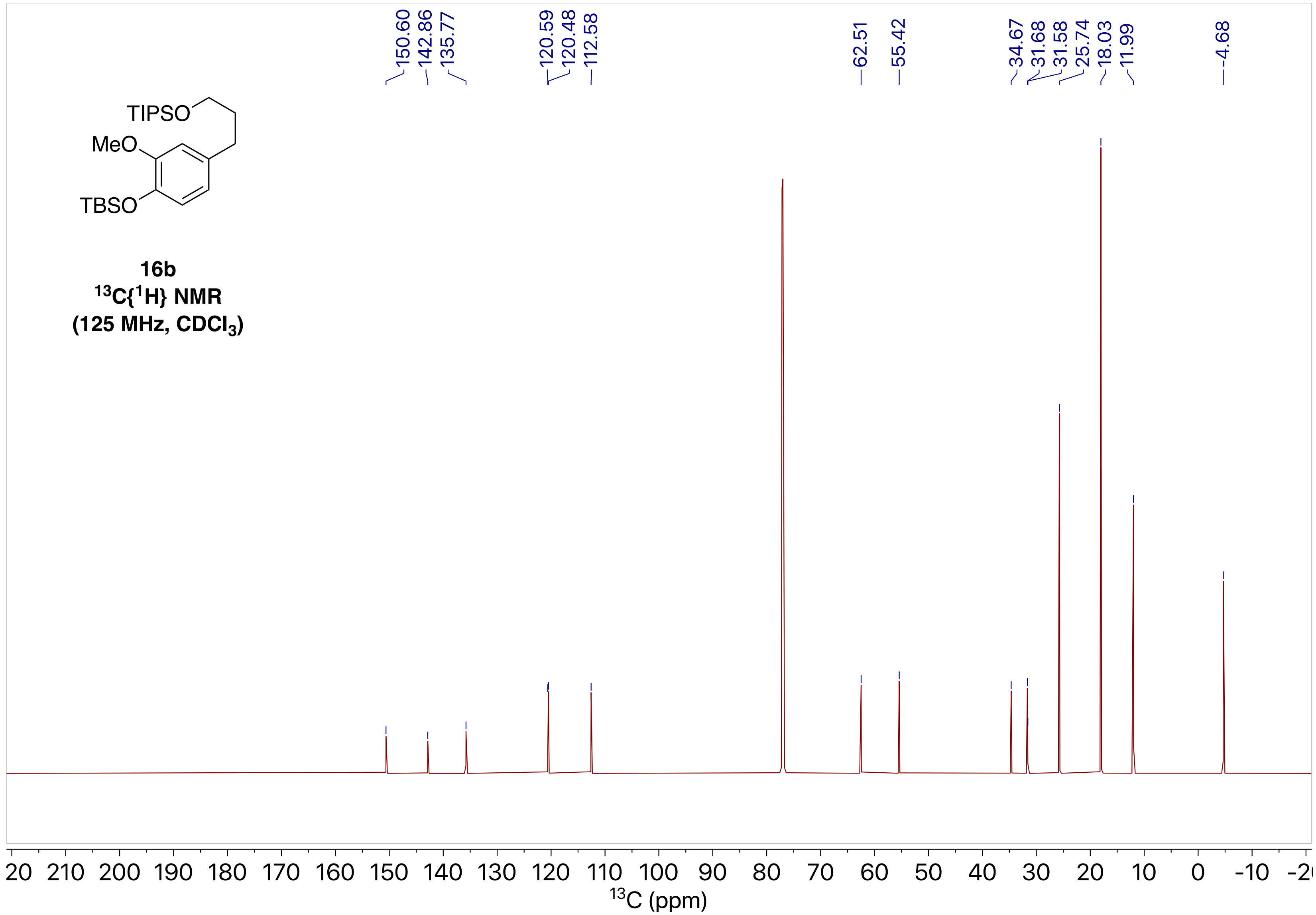

16b

$^{13}C\{^1H\}$ NMR

(125 MHz, $CDCl_3$)

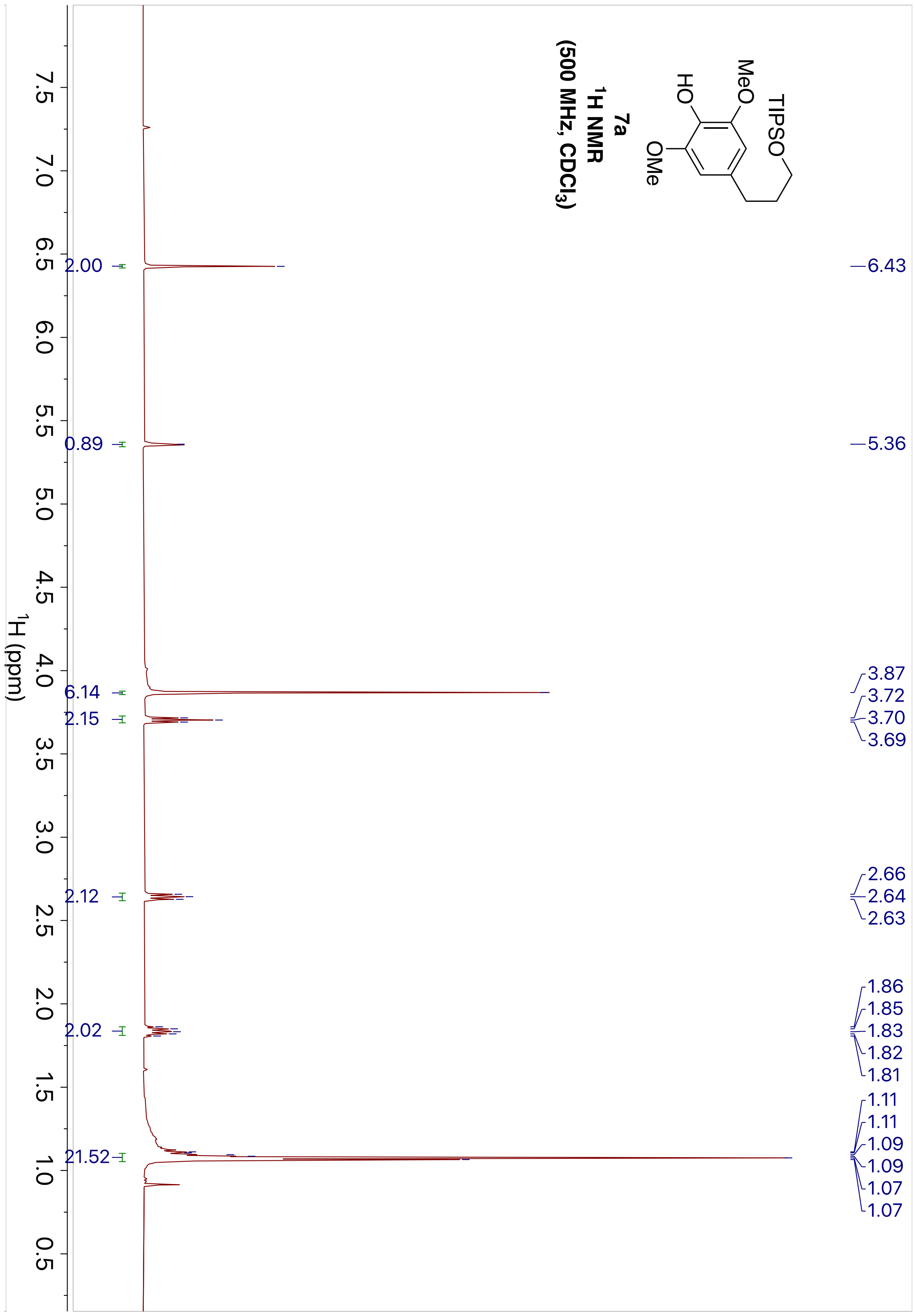

TIPSO
MeO
HO
OMe
7a
1H NMR
(500 MHz, CDCl3)
6.43
5.36
3.87
3.72
3.70
3.69
2.66
2.64
2.63
1.86
1.85
1.83
1.82
1.81
1.11
1.11
1.09
1.09
1.07
1.07
2.00
0.89
6.14
2.15
2.12
2.02
21.52
7.5
7.0
6.5
6.0
5.5
5.0
4.5
4.0
3.5
3.0
2.5
2.0
1.5
1.0
0.5
1H (ppm)

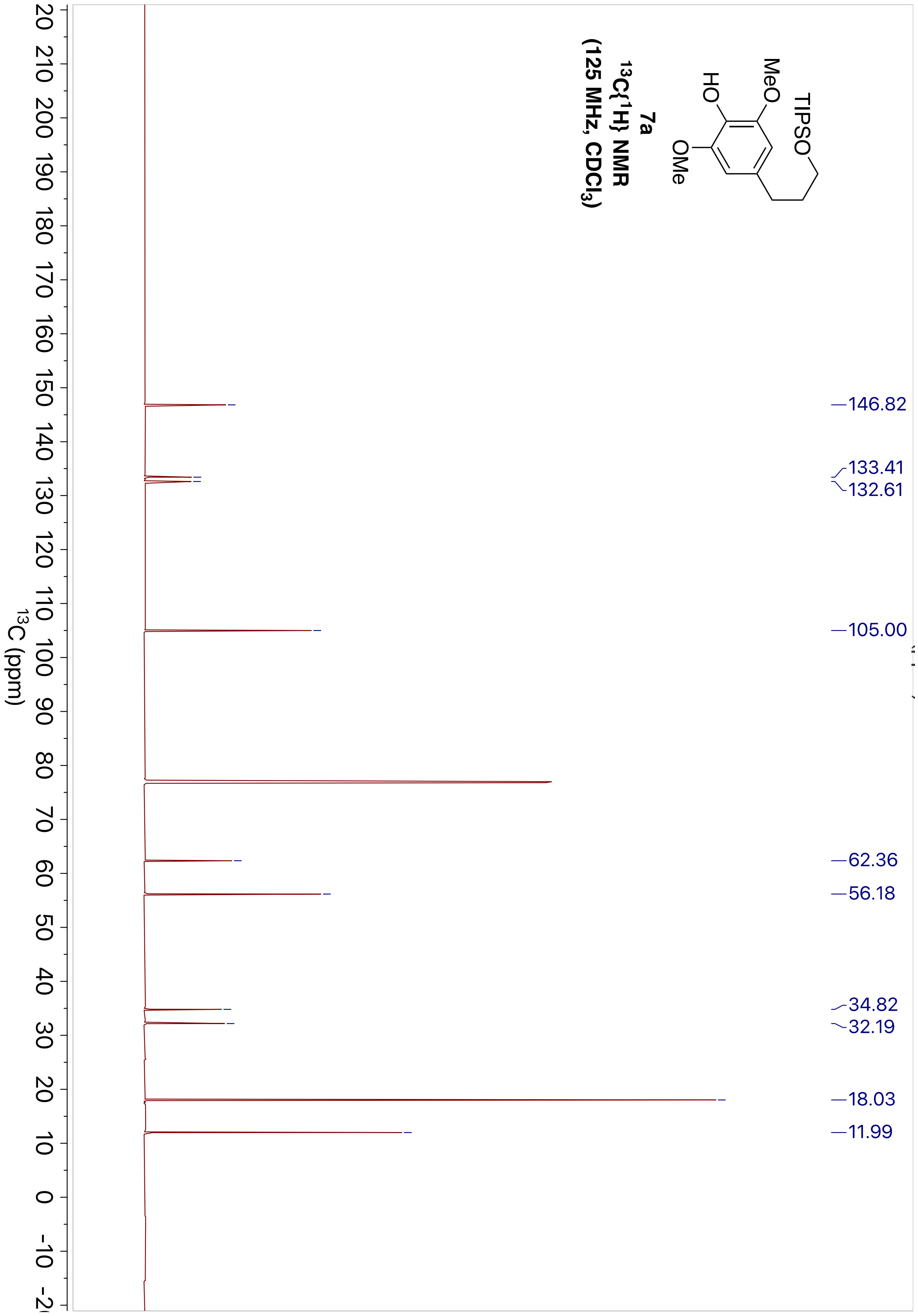
TIPSO
MeO
HO
OMe
7a
13C{1H} NMR
(125 MHz, CDCl3)
146.82
133.41
132.61
105.00
62.36
56.18
34.82
32.19
18.03
11.99
13C (ppm)

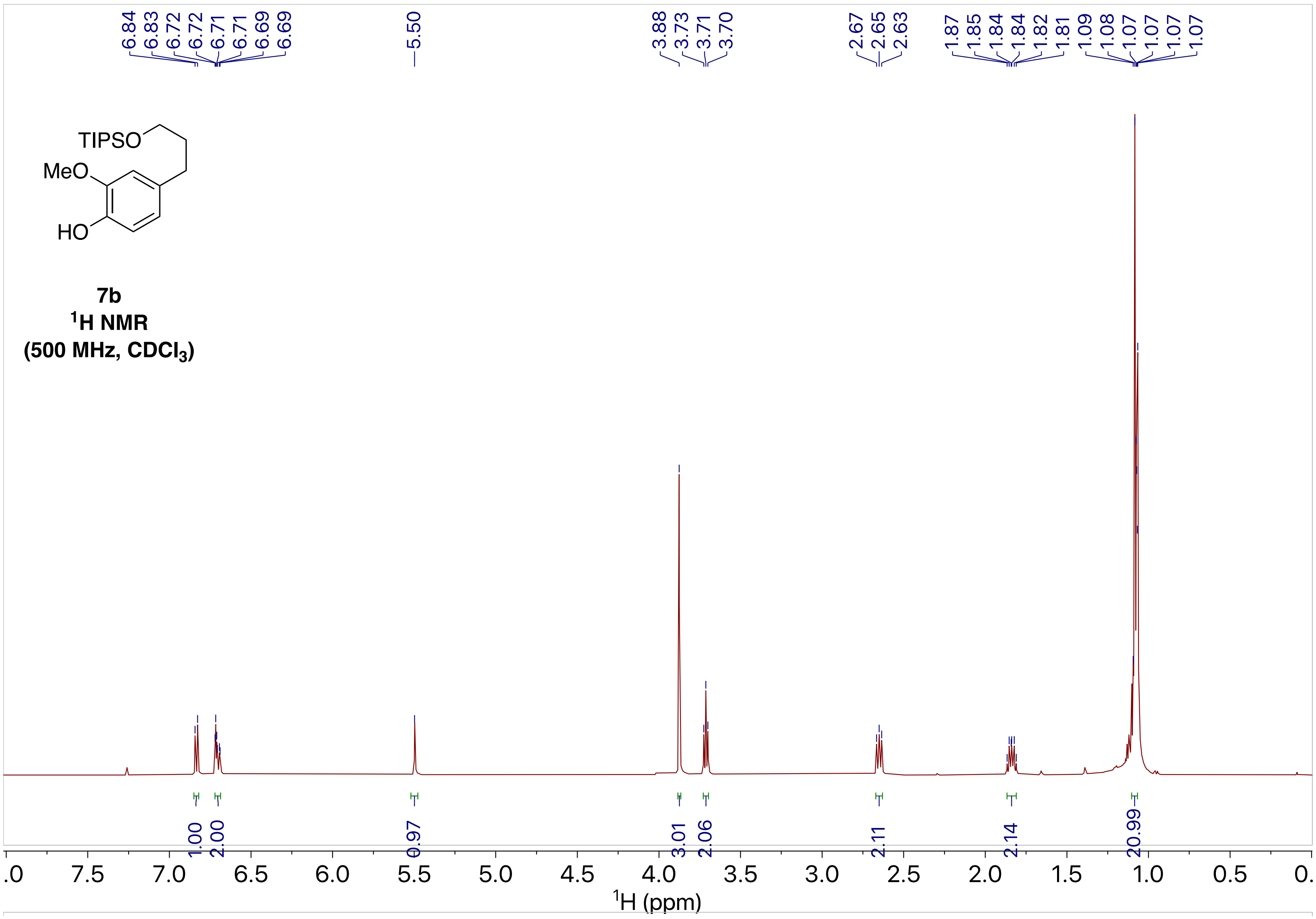
6.84
6.83
6.72
6.72
6.71
6.71
6.69
6.69
5.50
3.88
3.73
3.71
3.70
2.67
2.65
2.63
1.87
1.85
1.84
1.84
1.82
1.81
1.09
1.08
1.07
1.07
1.07
1.07
TIPSO
MeO
HO
7b
1H NMR
(500 MHz, CDCl3)
1.00
2.00
0.97
3.01
2.06
2.11
2.14
20.99
.0
7.5
7.0
6.5
6.0
5.5
5.0
4.5
4.0
3.5
3.0
2.5
2.0
1.5
1.0
0.5
0.
1H (ppm)

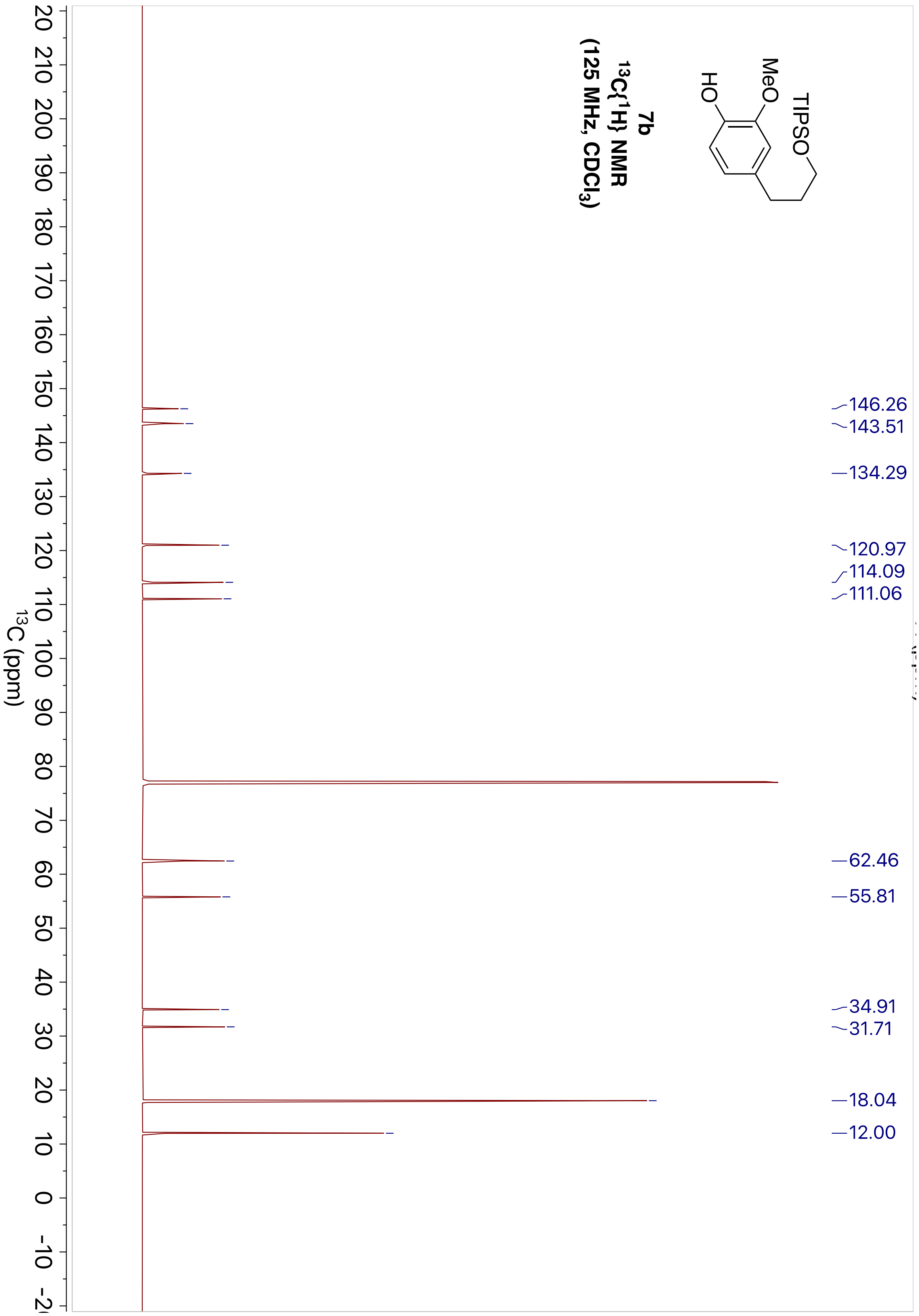
TIPSO
MeO
HO
7b
13C{1H} NMR
(125 MHz, CDCl3)
146.26
143.51
134.29
120.97
114.09
111.06
62.46
55.81
34.91
31.71
18.04
12.00
13C (ppm)

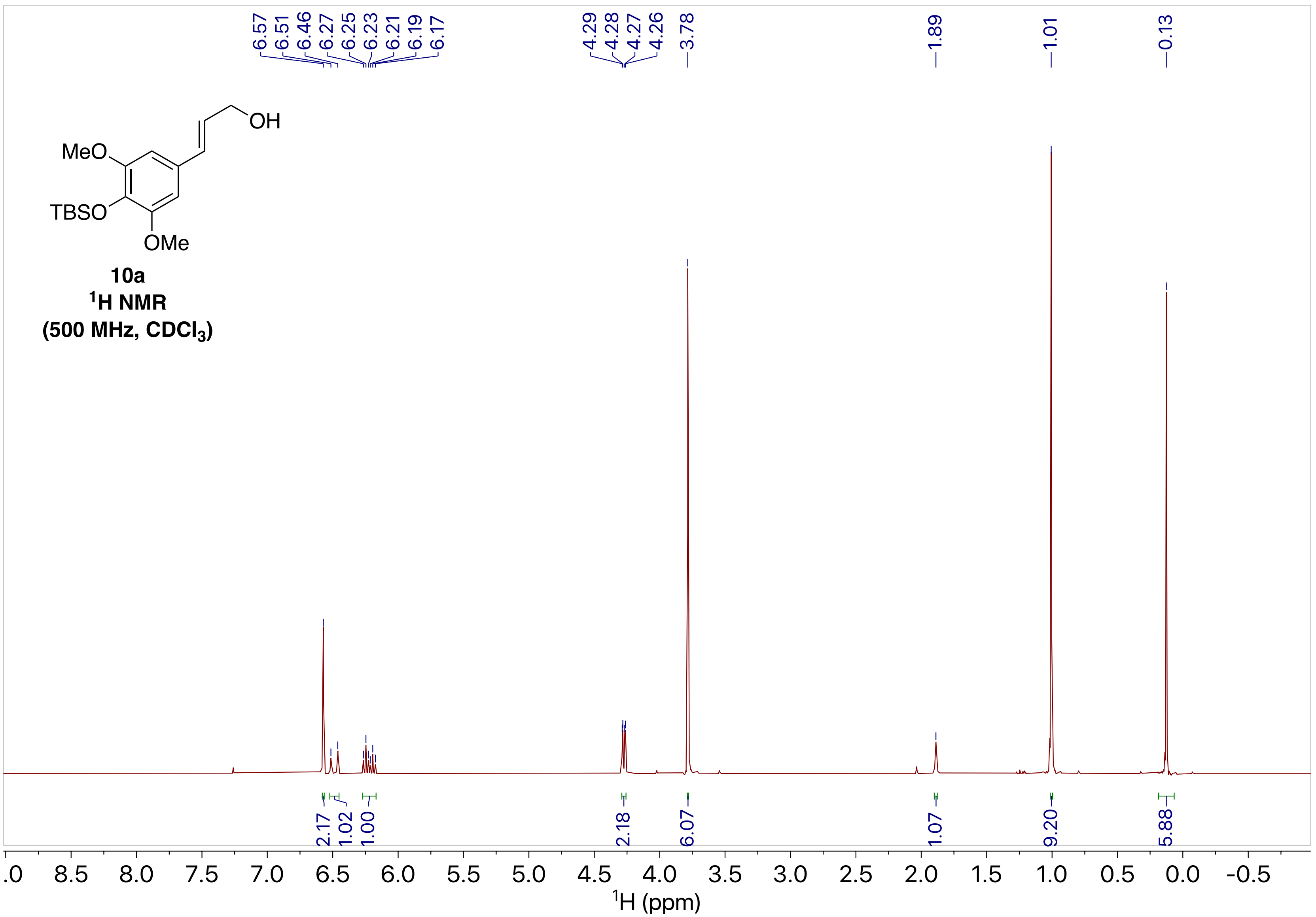
MeO
TBSO
OMe
OH
10a
^{1}H NMR
(500 MHz, $CDCl_3$)
6.57
6.51
6.46
6.27
6.25
6.23
6.21
6.19
6.17
4.29
4.28
4.27
4.26
3.78
1.89
1.01
0.13
2.17
1.02
1.00
2.18
6.07
1.07
9.20
5.88
.0
8.5
8.0
7.5
7.0
6.5
6.0
5.5
5.0
4.5
4.0
3.5
3.0
2.5
2.0
1.5
1.0
0.5
0.0
-0.5
^{1}H (ppm)

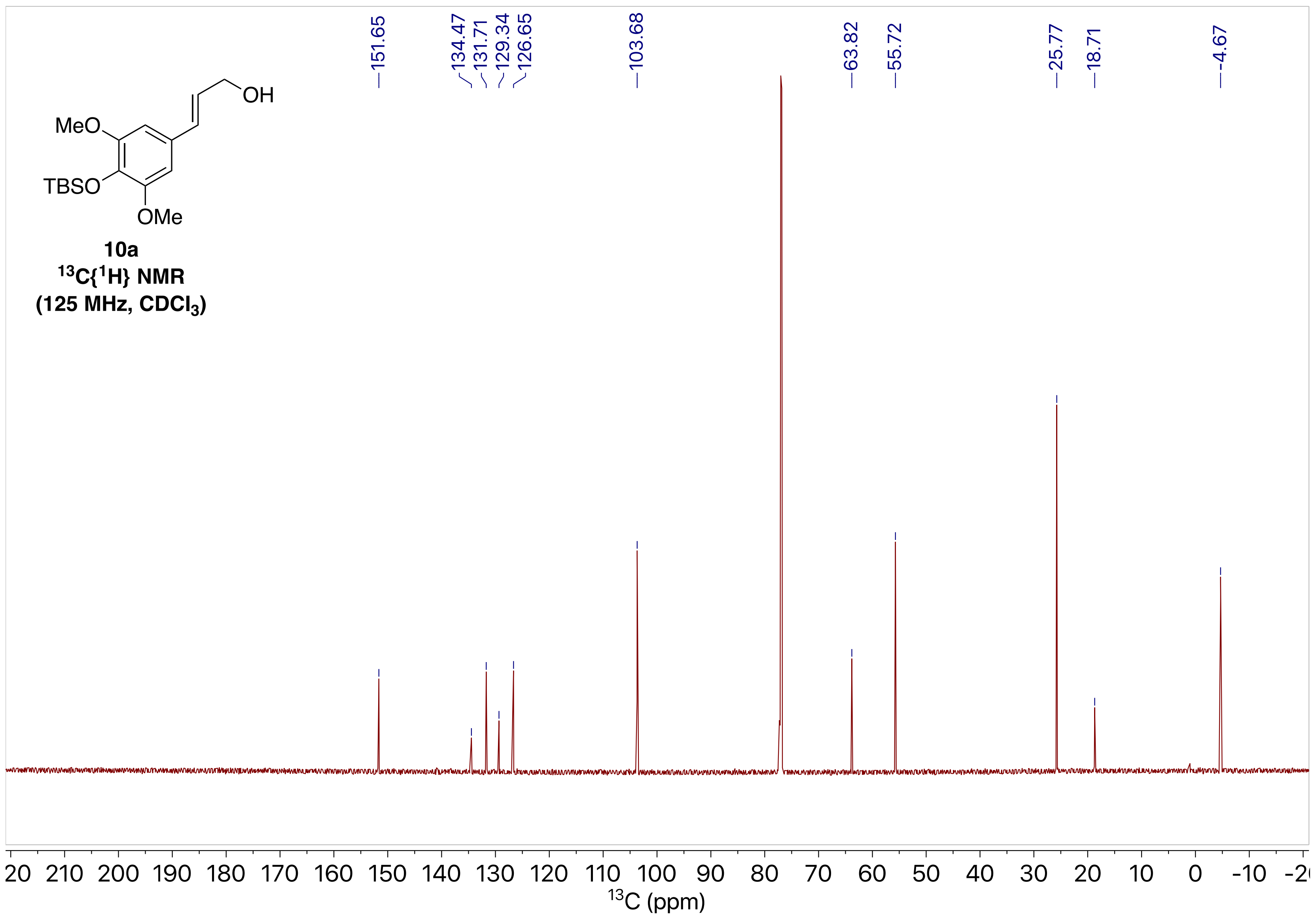
MeO
TBSO
OMe
OH
10a
$^{13}C\{^{1}H\}$ NMR
(125 MHz, $CDCl_3$)
151.65
134.47
131.71
129.34
126.65
103.68
63.82
55.72
25.77
18.71
-4.67
20 210 200 190 180 170 160 150 140 130 120 110 100 90 80 70 60 50 40 30 20 10 0 -10 -2
^{13}C (ppm)

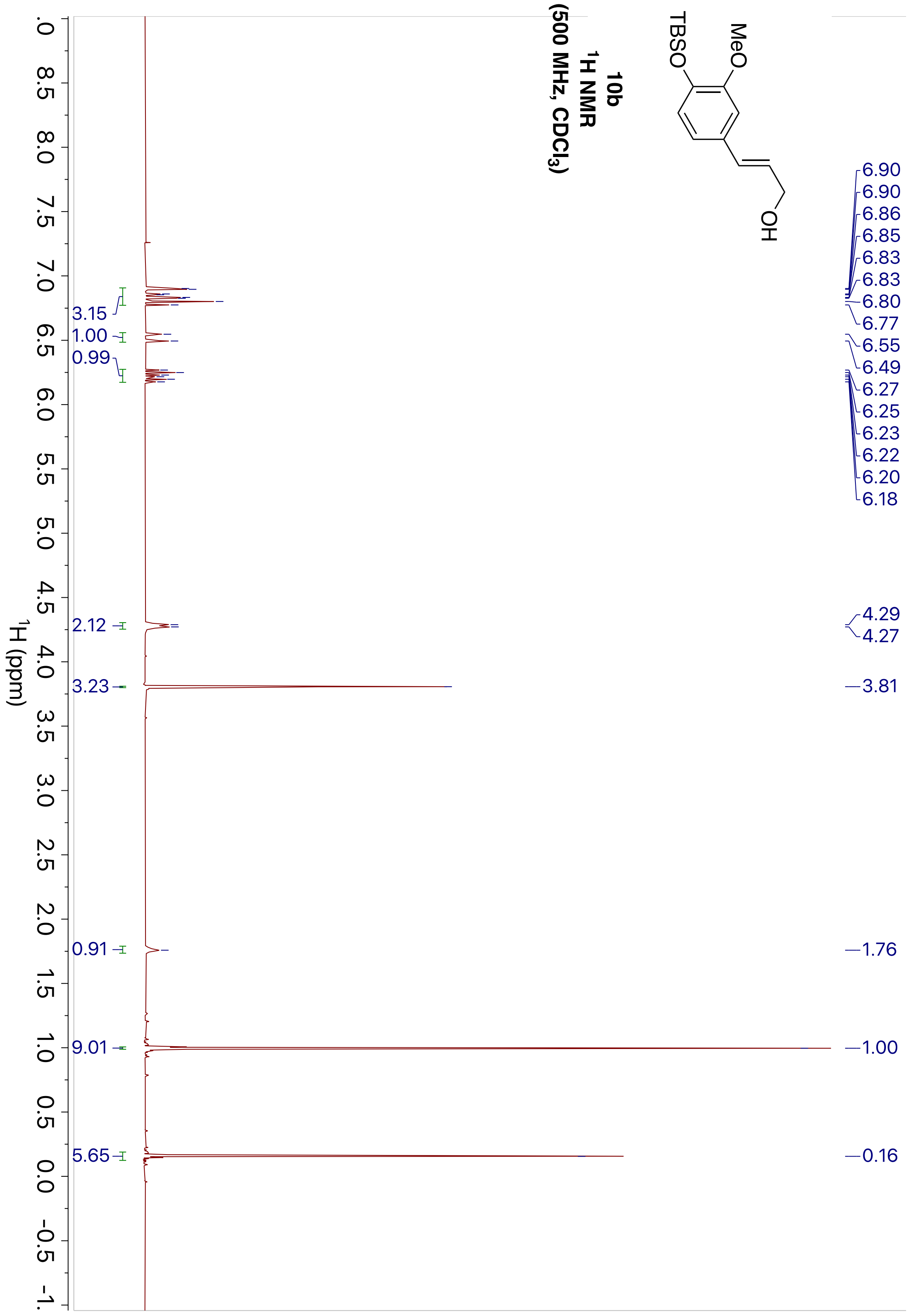
MeO
TBSO
OH
10b
1H NMR
(500 MHz, CDCl3)
6.90
6.90
6.86
6.85
6.83
6.83
6.80
6.77
6.55
6.49
6.27
6.25
6.23
6.22
6.20
6.18
4.29
4.27
3.81
1.76
1.00
0.16
3.15
1.00
0.99
2.12
3.23
0.91
9.01
5.65
1H (ppm)

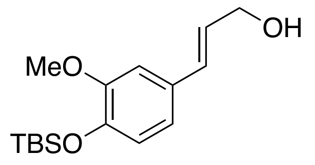

10b
$^{13}C\{^1H\}$ NMR
(125 MHz, $CDCl_3$)

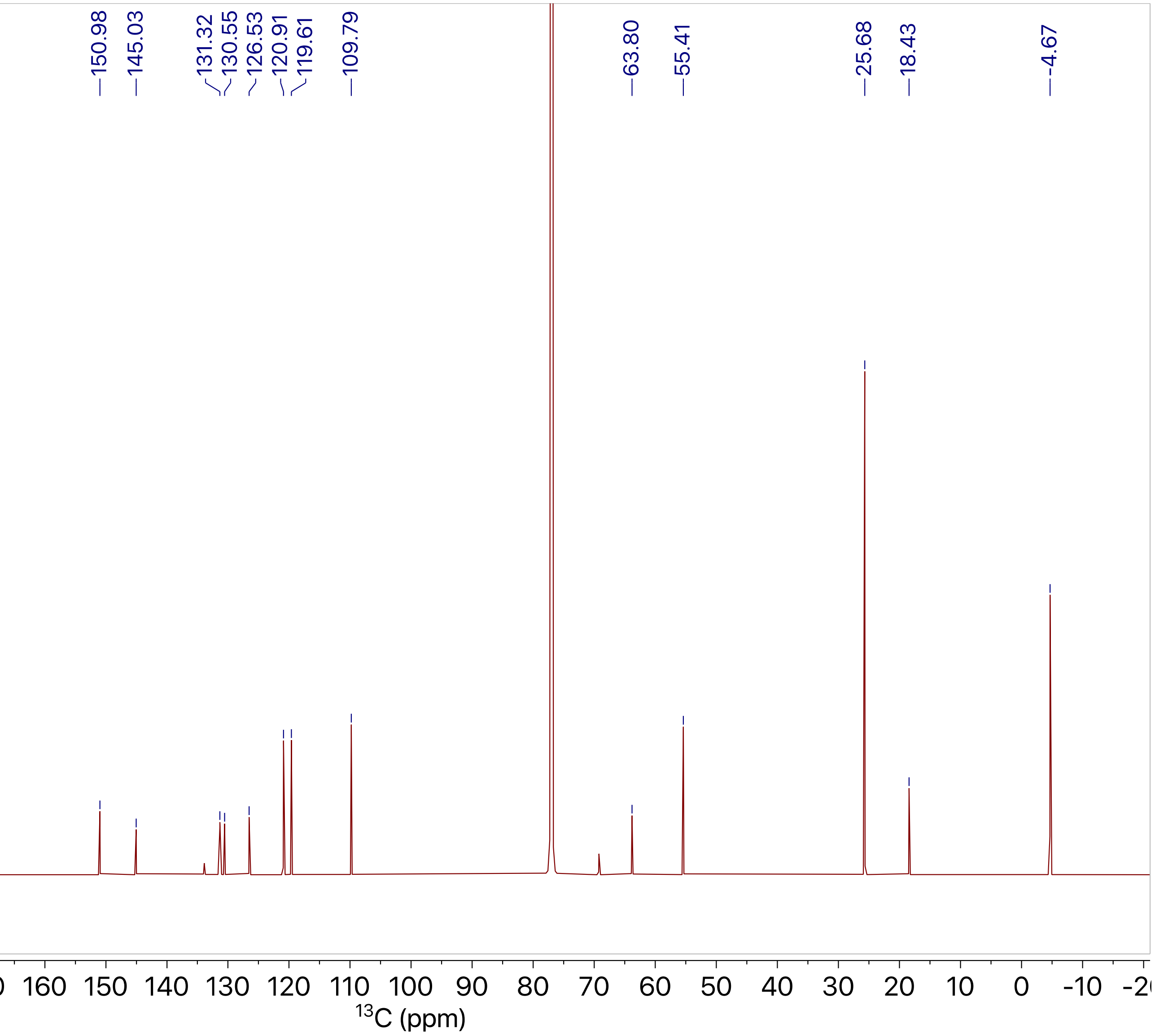

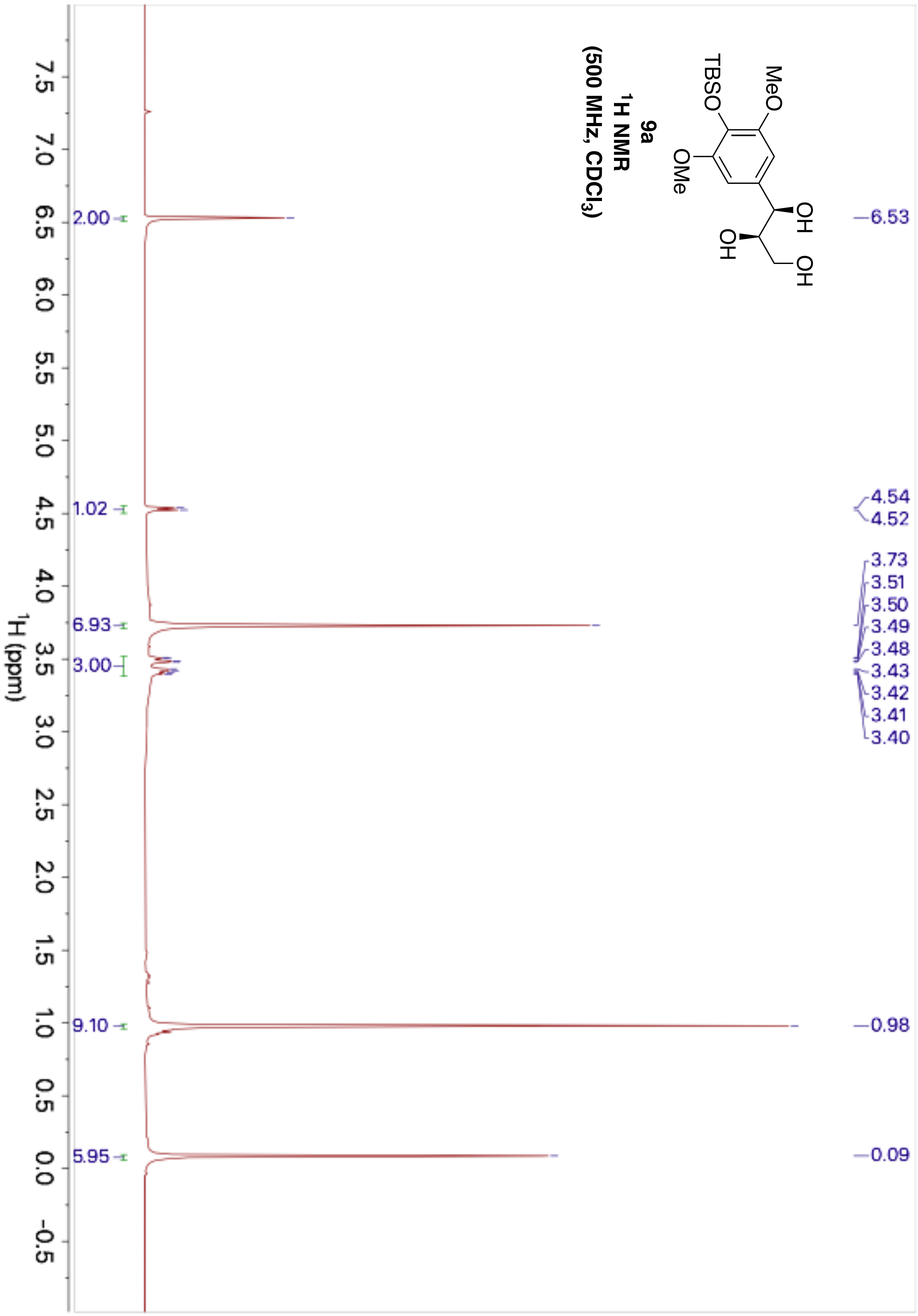

MeO
TBSO
OMe
OH
OH
OH
9a
1H NMR
(500 MHz, CDCl3)
6.53
4.54
4.52
3.73
3.51
3.50
3.49
3.48
3.43
3.42
3.41
3.40
0.98
0.09
2.00
1.02
6.93
3.00
9.10
5.95
7.5
7.0
6.5
6.0
5.5
5.0
4.5
4.0
3.5
3.0
2.5
2.0
1.5
1.0
0.5
0.0
-0.5
1H (ppm)

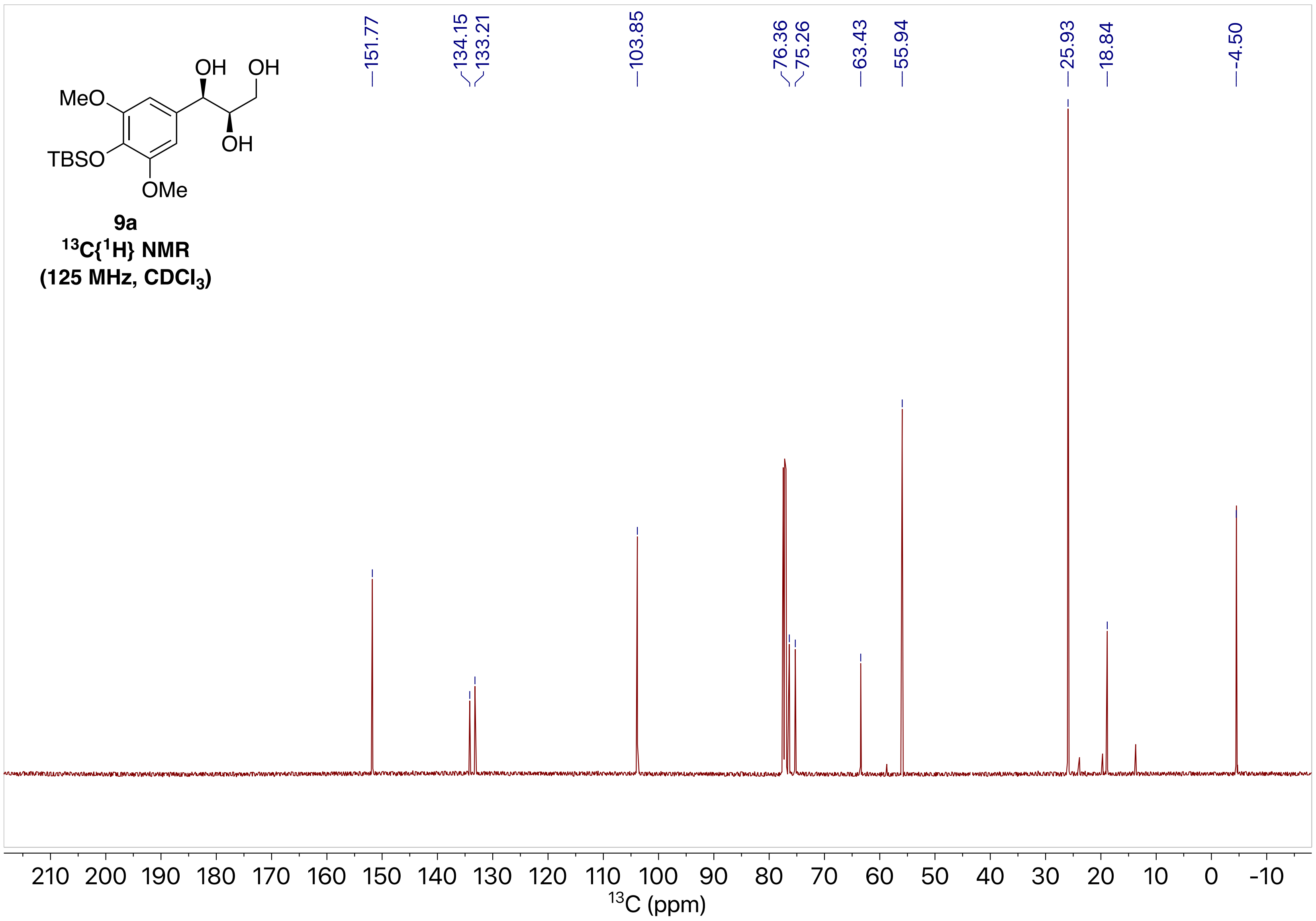
MeO
TBSO
OMe
OH
OH
OH
9a
13C{1H} NMR
(125 MHz, CDCl3)
151.77
134.15
133.21
103.85
76.36
75.26
63.43
55.94
25.93
18.84
-4.50
210 200 190 180 170 160 150 140 130 120 110 100 90 80 70 60 50 40 30 20 10 0 -10
13C (ppm)

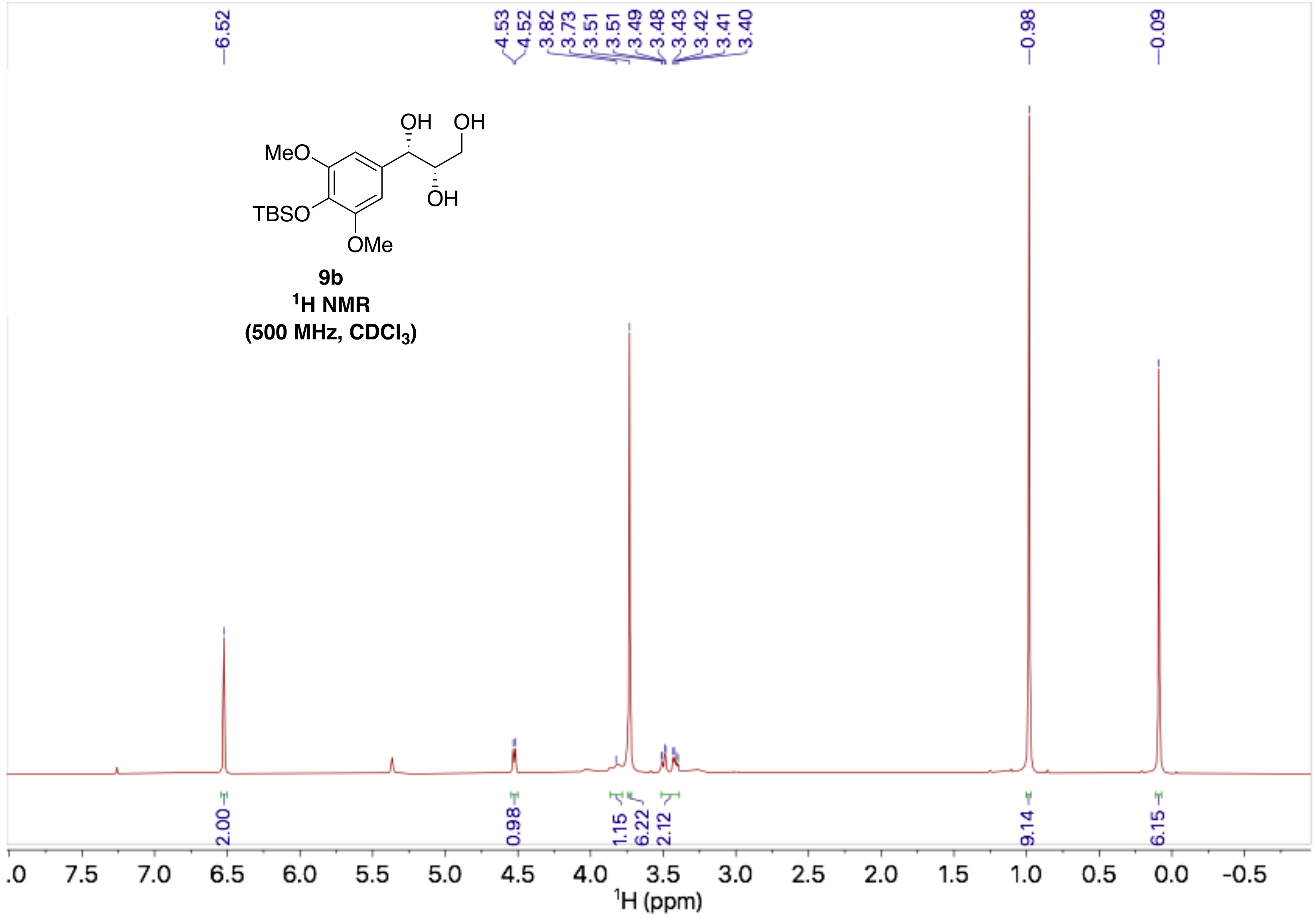
9b
1H NMR
(500 MHz, CDCl3)
MeO
TBSO
OMe
OH
OH
OH
6.52
4.53
4.52
3.82
3.73
3.51
3.51
3.49
3.48
3.43
3.42
3.41
3.40
0.98
0.09
2.00
0.98
1.15
6.22
2.12
9.14
6.15
1H (ppm)

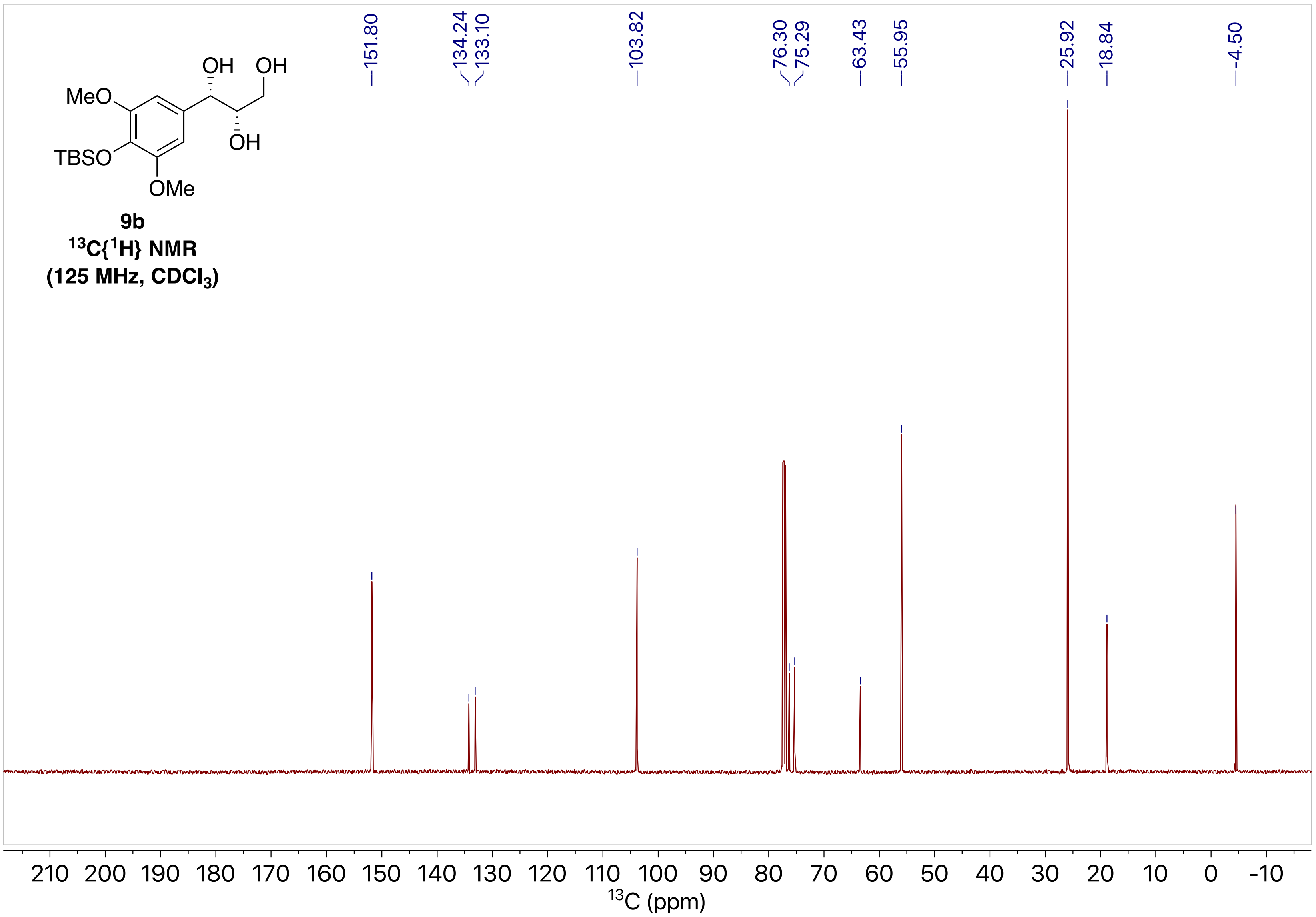

MeO
TBSO
OMe
OH
OH
OH
9b
¹³C{¹H} NMR
(125 MHz, CDCl₃)
151.80
134.24
133.10
103.82
76.30
75.29
63.43
55.95
25.92
18.84
-4.50
210 200 190 180 170 160 150 140 130 120 110 100 90 80 70 60 50 40 30 20 10 0 -10
¹³C (ppm)

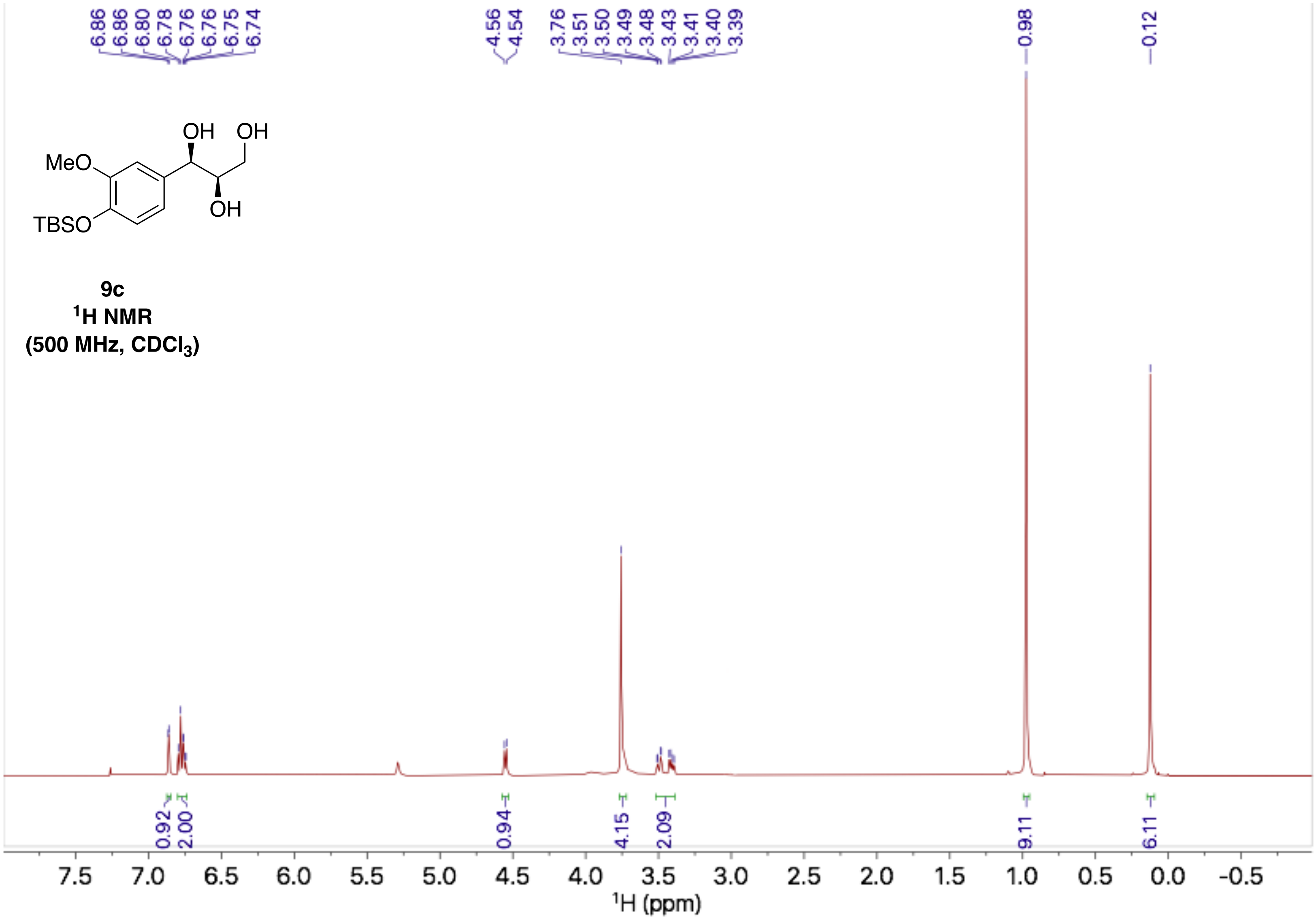

6.86
6.86
6.80
6.78
6.76
6.76
6.75
6.74
4.56
4.54
3.76
3.51
3.50
3.49
3.48
3.43
3.41
3.40
3.39
0.98
0.12
MeO
TBSO
OH
OH
OH
9c
1H NMR
(500 MHz, CDCl3)
0.92
2.00
0.94
4.15
2.09
9.11
6.11
7.5
7.0
6.5
6.0
5.5
5.0
4.5
4.0
3.5
3.0
2.5
2.0
1.5
1.0
0.5
0.0
-0.5
1H (ppm)

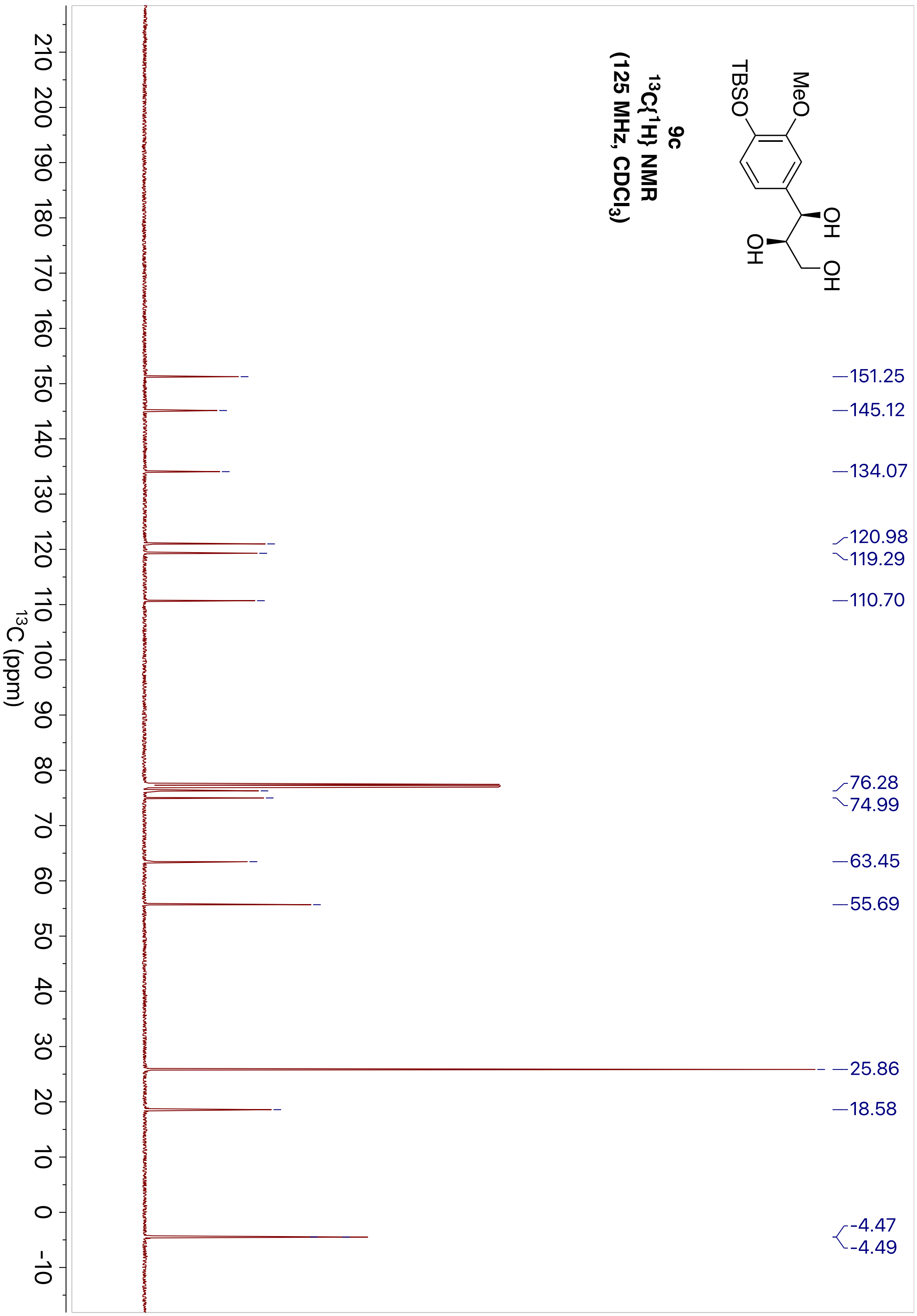
9c
$^{13}C\{^1H\}$ NMR
(125 MHz, $CDCl_3$)
MeO
TBSO
OH
OH
OH
151.25
145.12
134.07
120.98
119.29
110.70
76.28
74.99
63.45
55.69
25.86
18.58
-4.47
-4.49
210 200 190 180 170 160 150 140 130 120 110 100 90 80 70 60 50 40 30 20 10 0 -10
^{13}C (ppm)

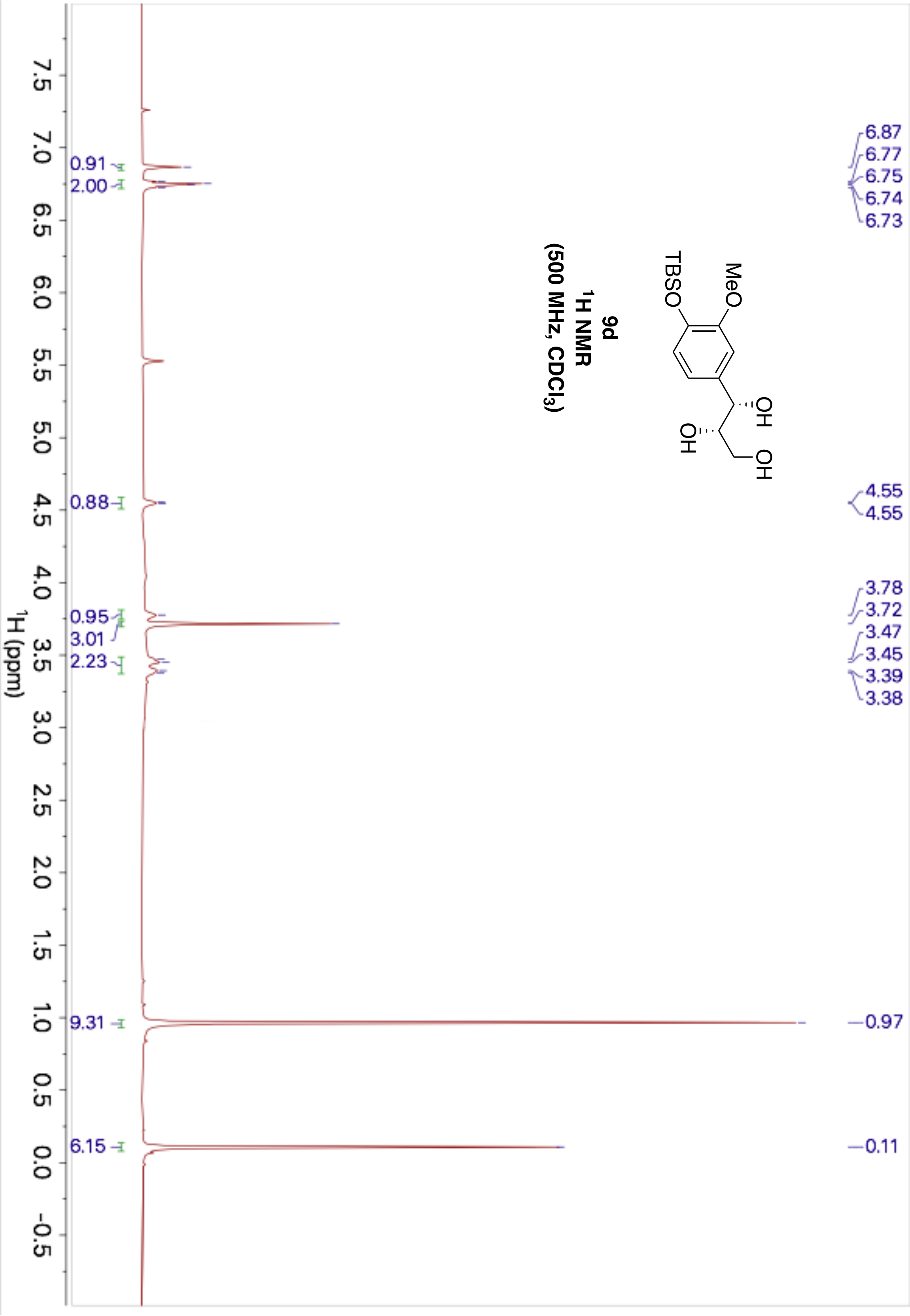
9d
1H NMR
(500 MHz, CDCl3)
MeO
TBSO
OH
OH
OH
6.87
6.77
6.75
6.74
6.73
4.55
4.55
3.78
3.72
3.47
3.45
3.39
3.38
0.97
0.11
0.91
2.00
0.88
0.95
3.01
2.23
9.31
6.15
1H (ppm)
7.5
7.0
6.5
6.0
5.5
5.0
4.5
4.0
3.5
3.0
2.5
2.0
1.5
1.0
0.5
0.0
-0.5

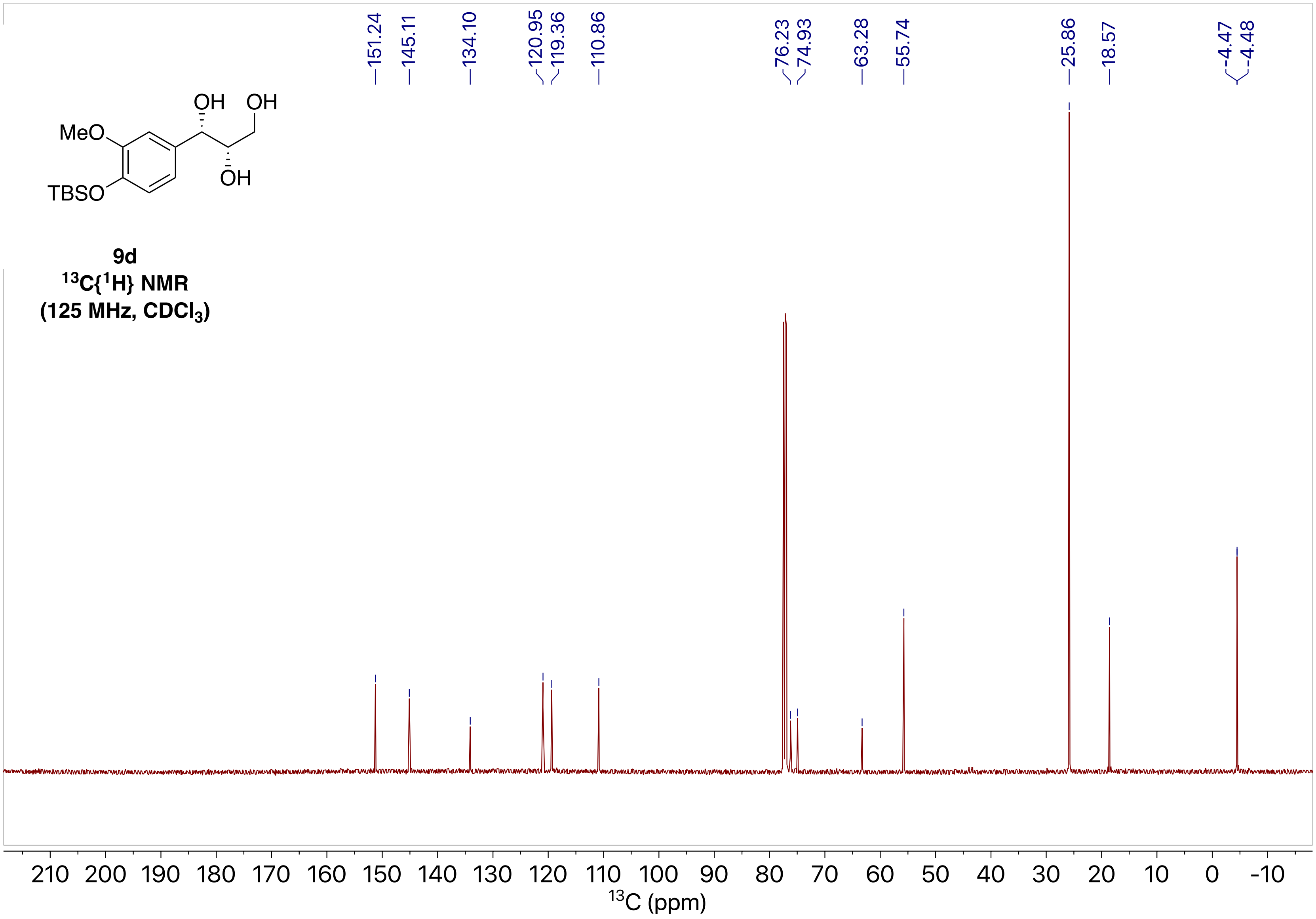
MeO
TBSO
OH
OH
OH
9d
$^{13}C\{^{1}H\}$ NMR
(125 MHz, $CDCl_3$)
151.24
145.11
134.10
120.95
119.36
110.86
76.23
74.93
63.28
55.74
25.86
18.57
-4.47
-4.48
210 200 190 180 170 160 150 140 130 120 110 100 90 80 70 60 50 40 30 20 10 0 -10
^{13}C (ppm)

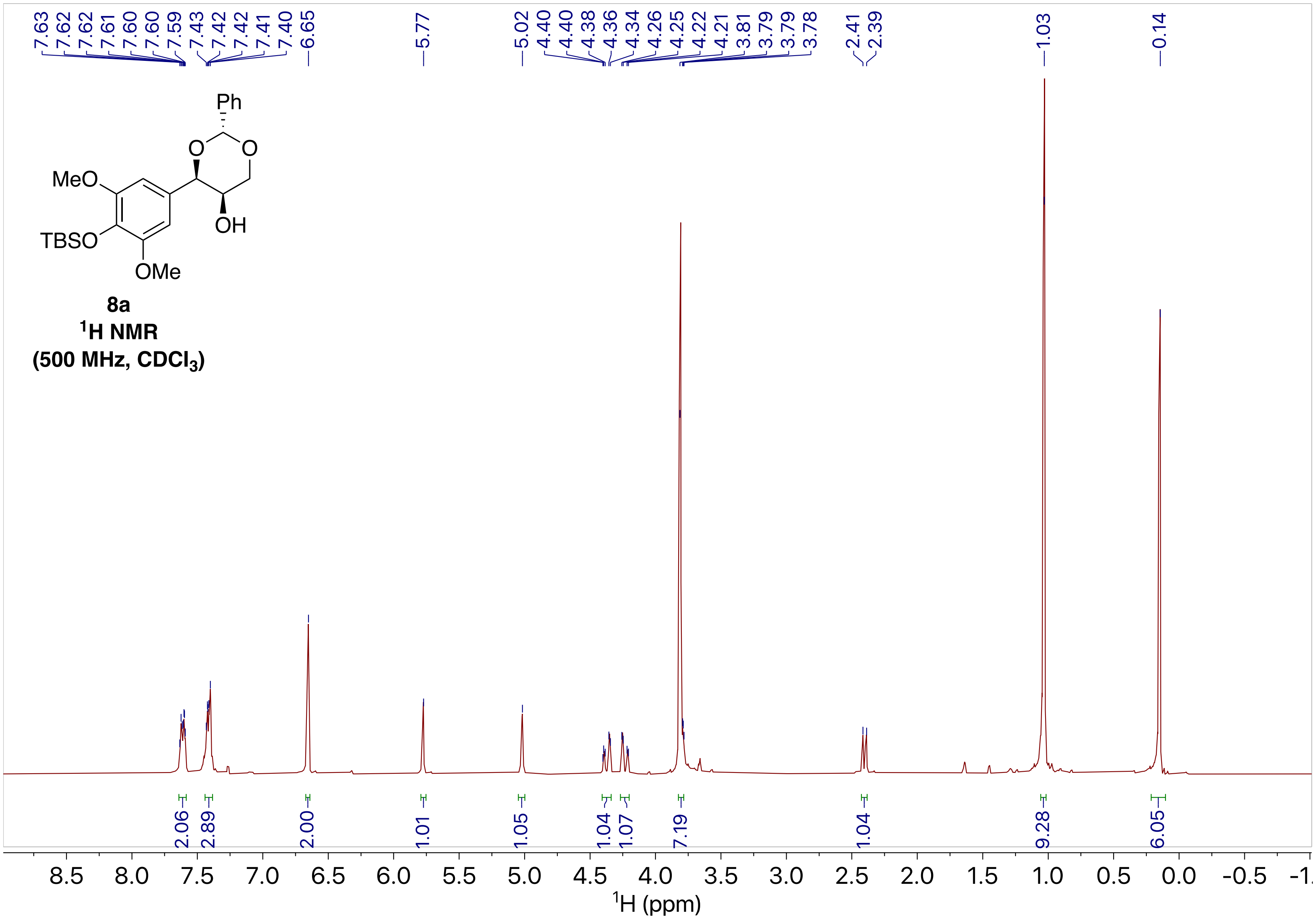
7.63 7.62 7.62 7.61 7.60 7.60 7.59 7.43 7.42 7.42 7.41 7.40 6.65 5.77 5.02 4.40 4.40 4.38 4.36 4.34 4.26 4.25 4.22 4.21 3.81 3.79 3.79 3.78 2.41 2.39 1.03 0.14
Ph
O
O
MeO
TBSO
OMe
OH
8a
^{1}H NMR
(500 MHz, $CDCl_3$)
2.06 2.89 2.00 1.01 1.05 1.04 1.07 7.19 1.04 9.28 6.05
8.5 8.0 7.5 7.0 6.5 6.0 5.5 5.0 4.5 4.0 3.5 3.0 2.5 2.0 1.5 1.0 0.5 0.0 -0.5
^{1}H (ppm)

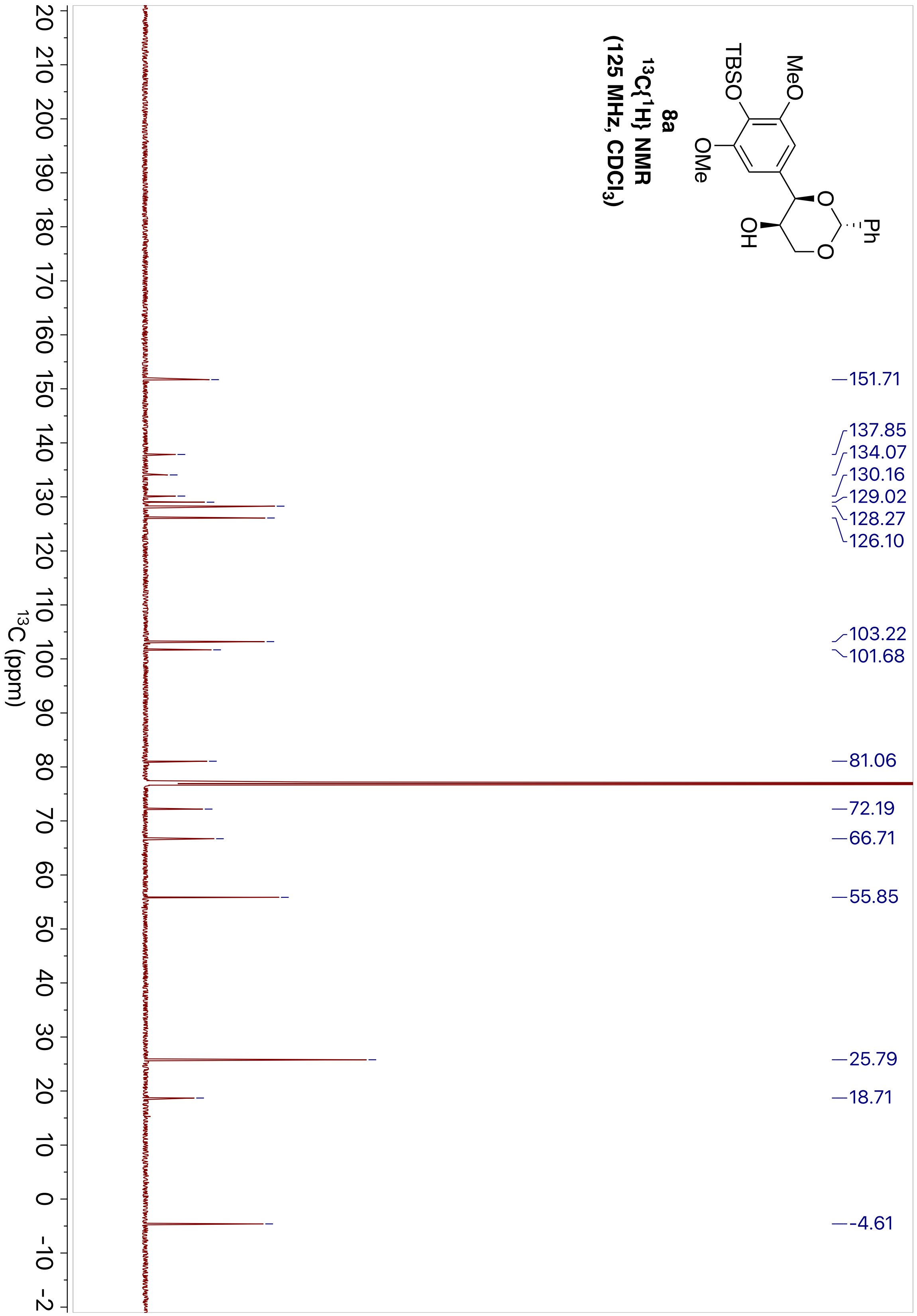

MeO
TBSO
OMe
OH
O
O
Ph
8a
13C{1H} NMR
(125 MHz, CDCl3)
151.71
137.85
134.07
130.16
129.02
128.27
126.10
103.22
101.68
81.06
72.19
66.71
55.85
25.79
18.71
-4.61
13C (ppm)
220 210 200 190 180 170 160 150 140 130 120 110 100 90 80 70 60 50 40 30 20 10 0 -10 -20

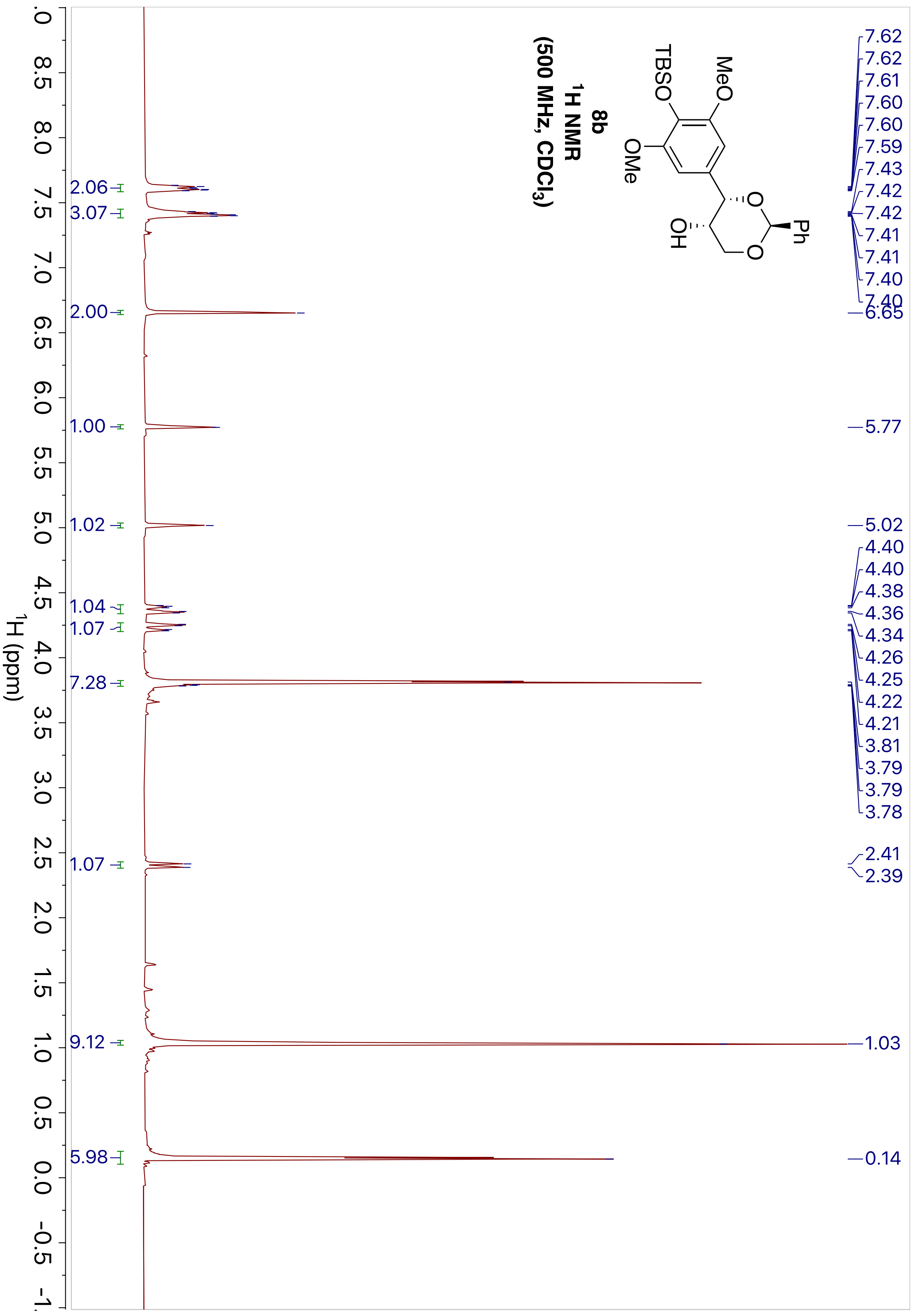
MeO
TBSO
OMe
Ph
OH
8b
1H NMR
(500 MHz, CDCl3)
7.62
7.62
7.61
7.60
7.60
7.59
7.43
7.42
7.42
7.41
7.41
7.40
7.40
6.65
5.77
5.02
4.40
4.40
4.38
4.36
4.34
4.26
4.25
4.22
4.21
3.81
3.79
3.79
3.78
2.41
2.39
1.03
0.14
2.06
3.07
2.00
1.00
1.02
1.04
1.07
7.28
1.07
9.12
5.98
1H (ppm)

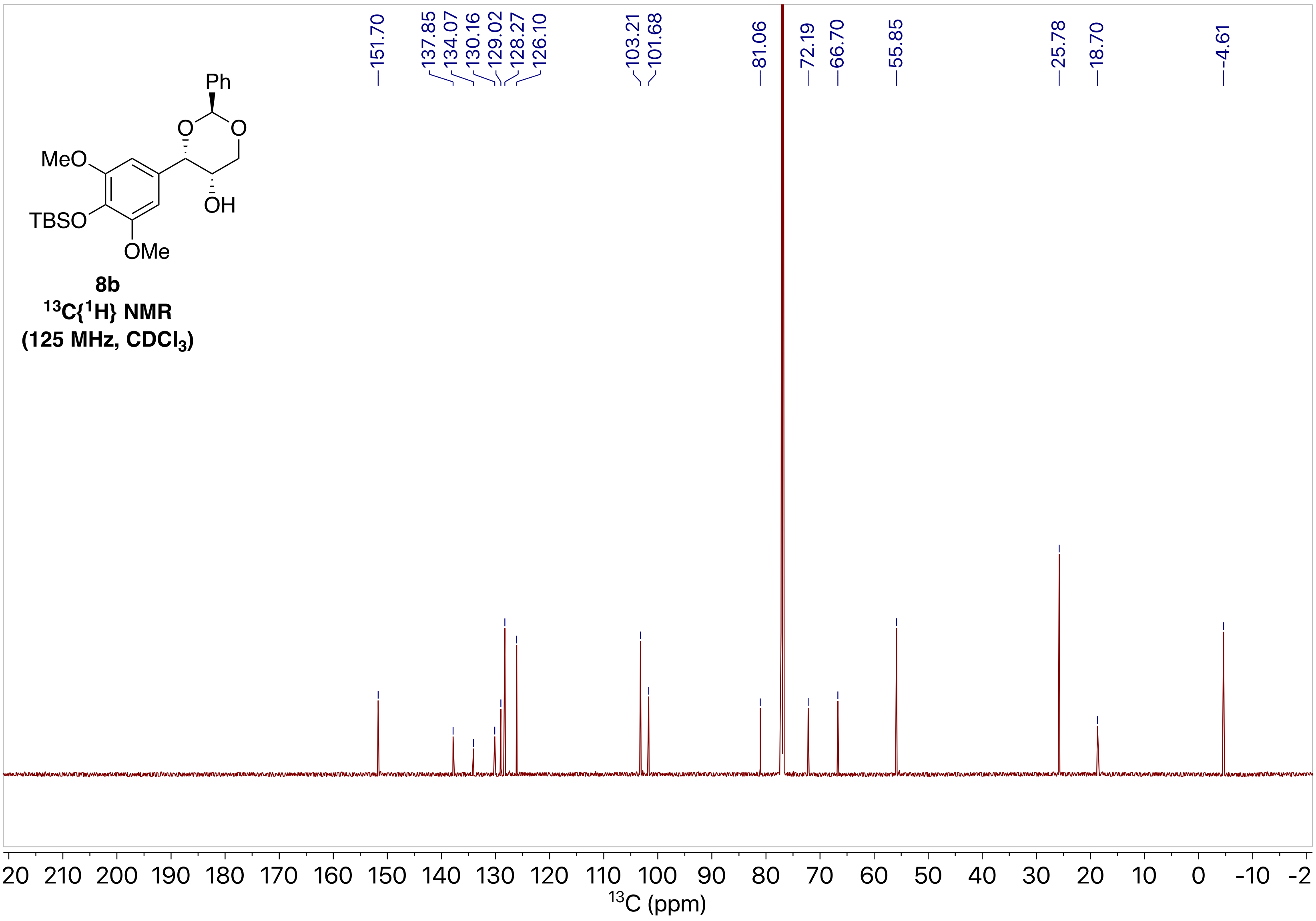

8b
$^{13}C\{^1H\}$ NMR
(125 MHz, $CDCl_3$)

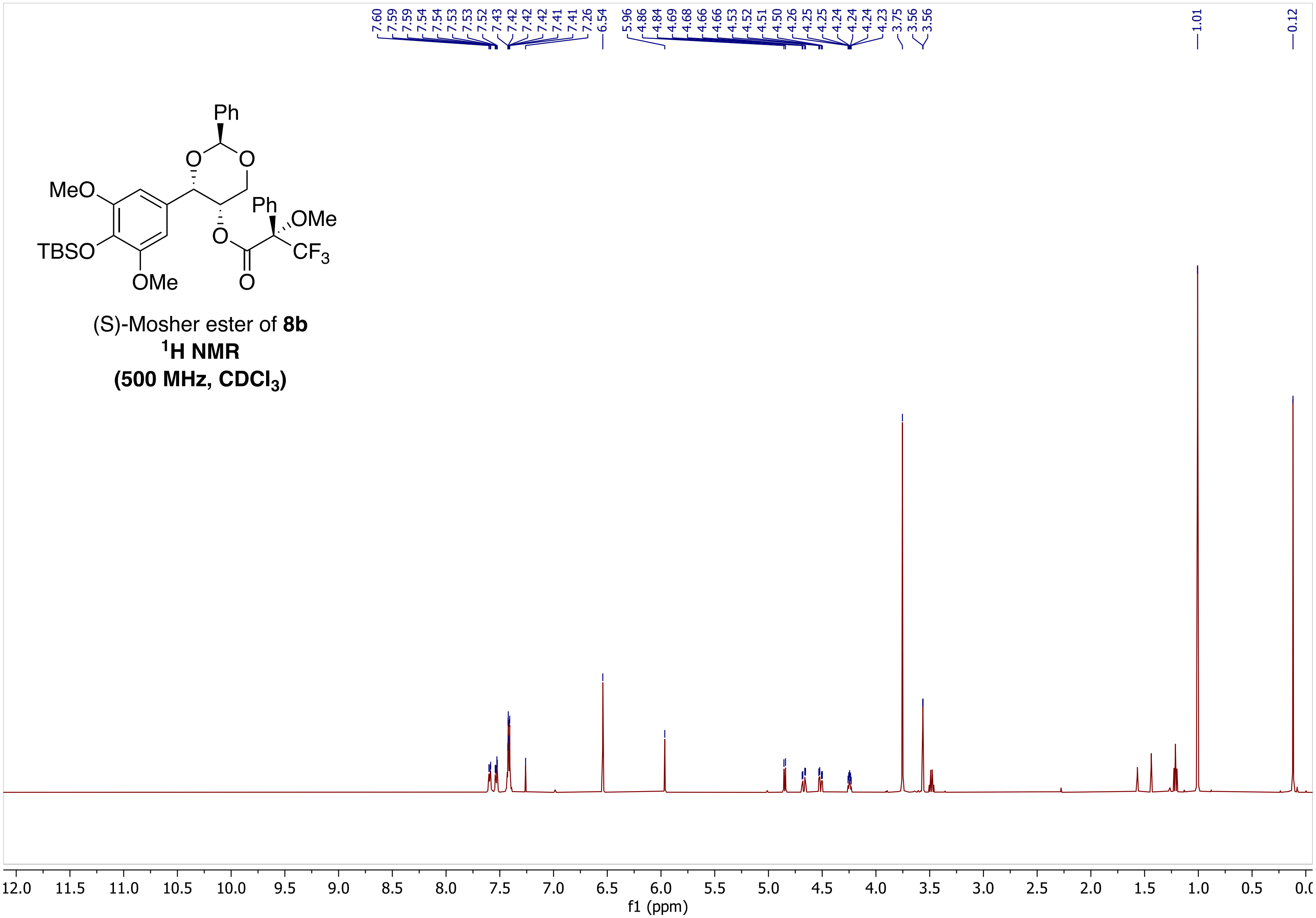
(S)-Mosher ester of 8b
1H NMR
(500 MHz, CDCl3)
MeO
TBSO
OMe
Ph
O
O
Ph
OMe
CF3
7.60
7.59
7.59
7.54
7.54
7.53
7.53
7.52
7.43
7.42
7.42
7.42
7.41
7.41
7.26
6.54
5.96
4.86
4.84
4.69
4.68
4.66
4.66
4.53
4.52
4.51
4.50
4.26
4.25
4.25
4.24
4.24
4.24
4.23
3.75
3.56
3.56
1.01
0.12
f1 (ppm)

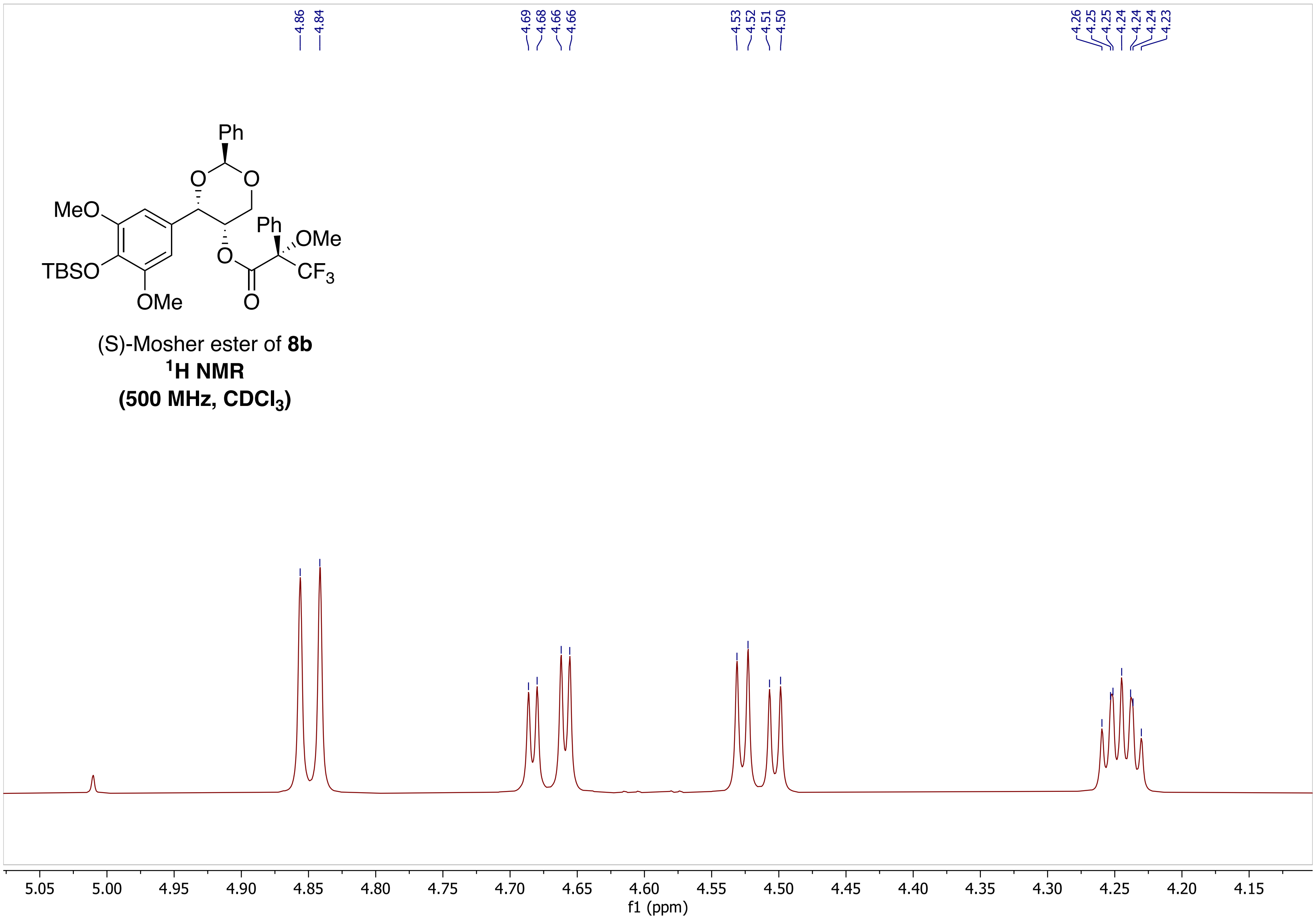
4.86
4.84
4.69
4.68
4.66
4.66
4.53
4.52
4.51
4.50
4.26
4.25
4.25
4.24
4.24
4.24
4.23
Ph
O
O
MeO
Ph
OMe
TBSO
O
CF3
OMe
O
(S)-Mosher ester of 8b
1H NMR
(500 MHz, CDCl3)
5.05
5.00
4.95
4.90
4.85
4.80
4.75
4.70
4.65
4.60
4.55
4.50
4.45
4.40
4.35
4.30
4.25
4.20
4.15
f1 (ppm)

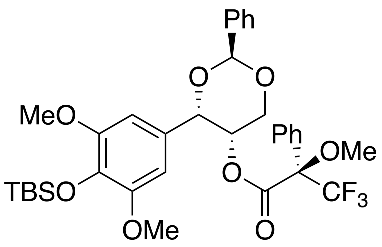

(R)-Mosher ester of **8b**
^{1}H NMR
(500 MHz, $CDCl_3$)

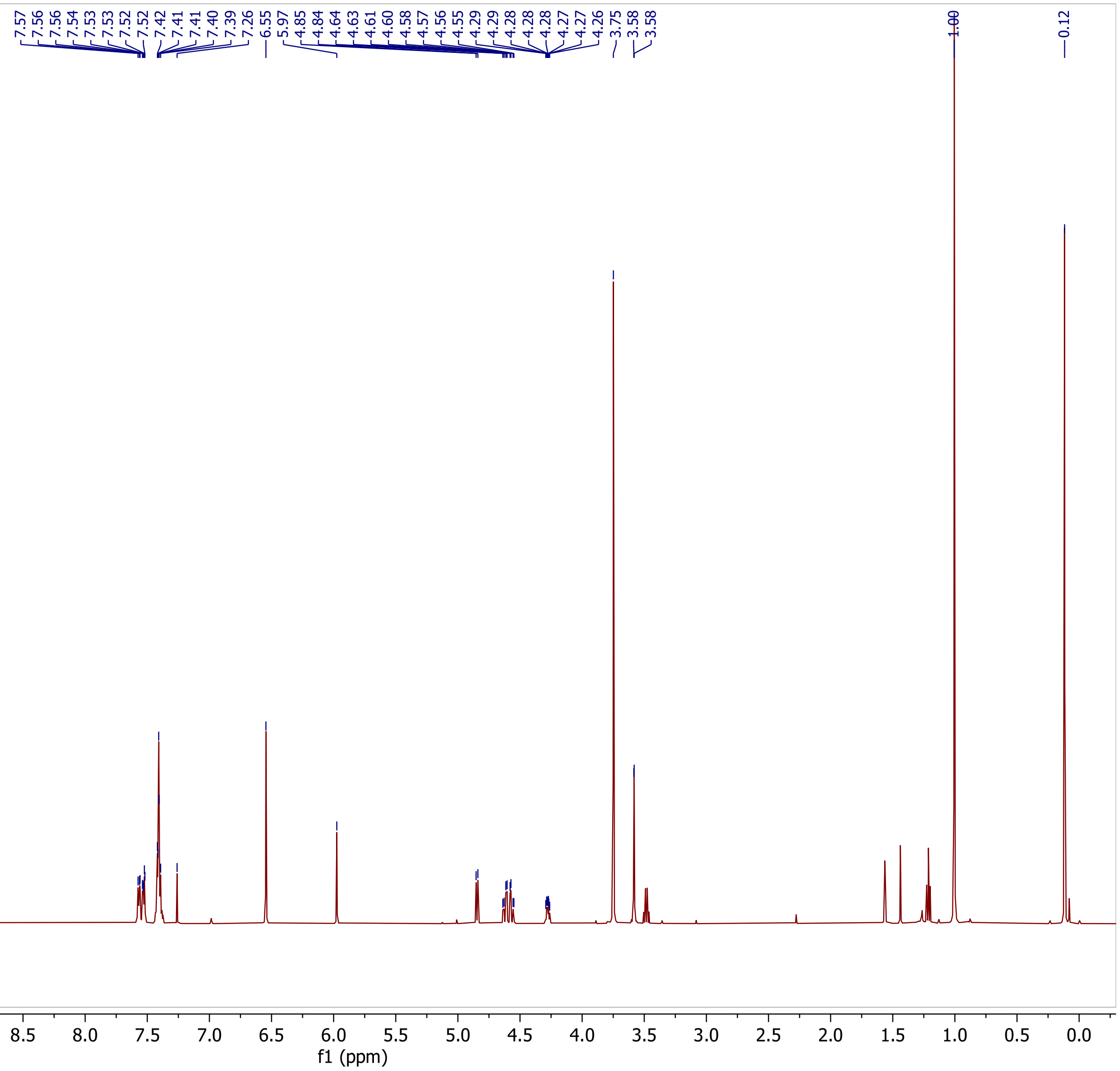

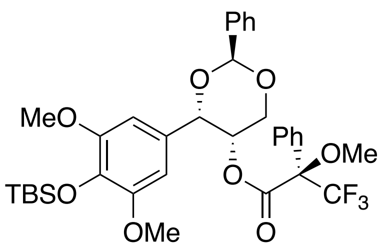

(R)-Mosher ester of **8b**
^{1}H NMR
(500 MHz, $CDCl_3$)

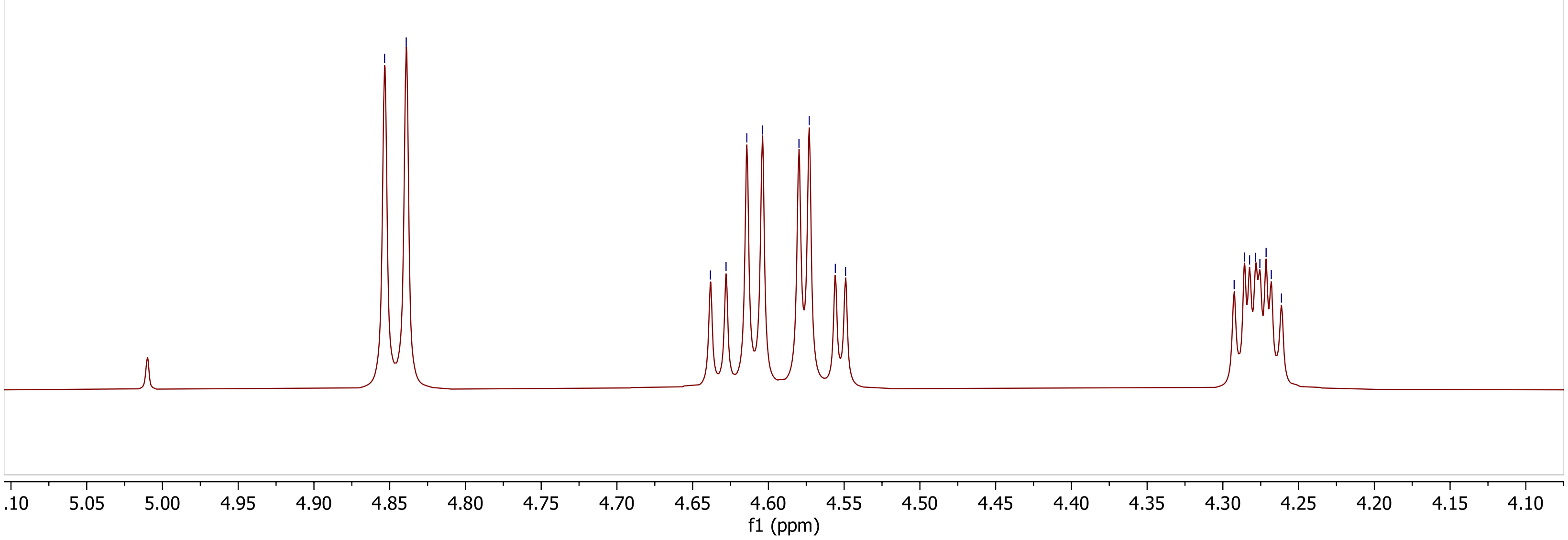

Overlay of (S)-Mosher ester (red) and (R)-Mosher ester (blue) of **8b**
^{1}H NMR (500 MHz, $CDCl_3$)

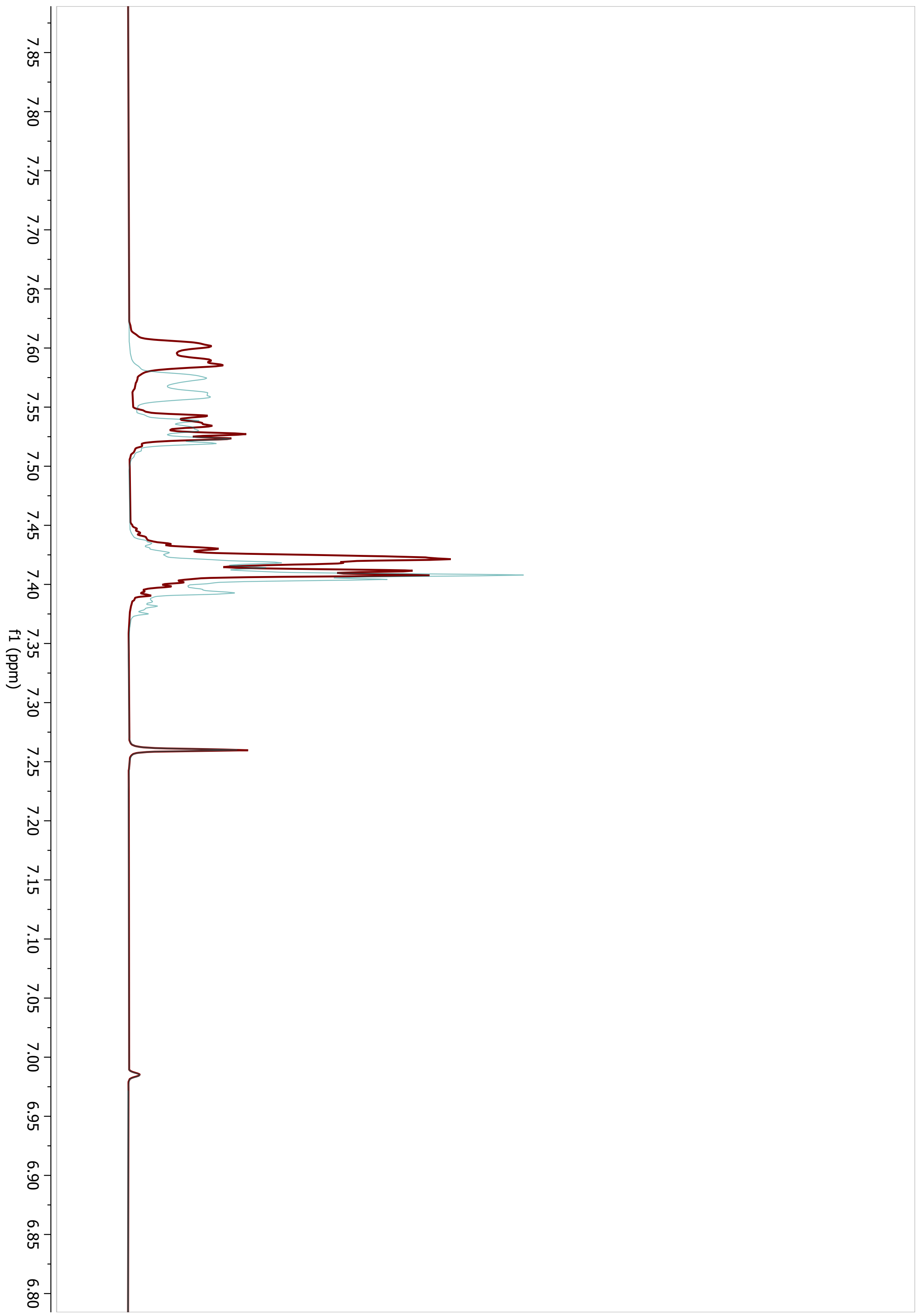

Overlay of (S)-Mosher ester (red) and (R)-Mosher ester (blue) of **8b**
^{1}H NMR (500 MHz, $CDCl_3$)

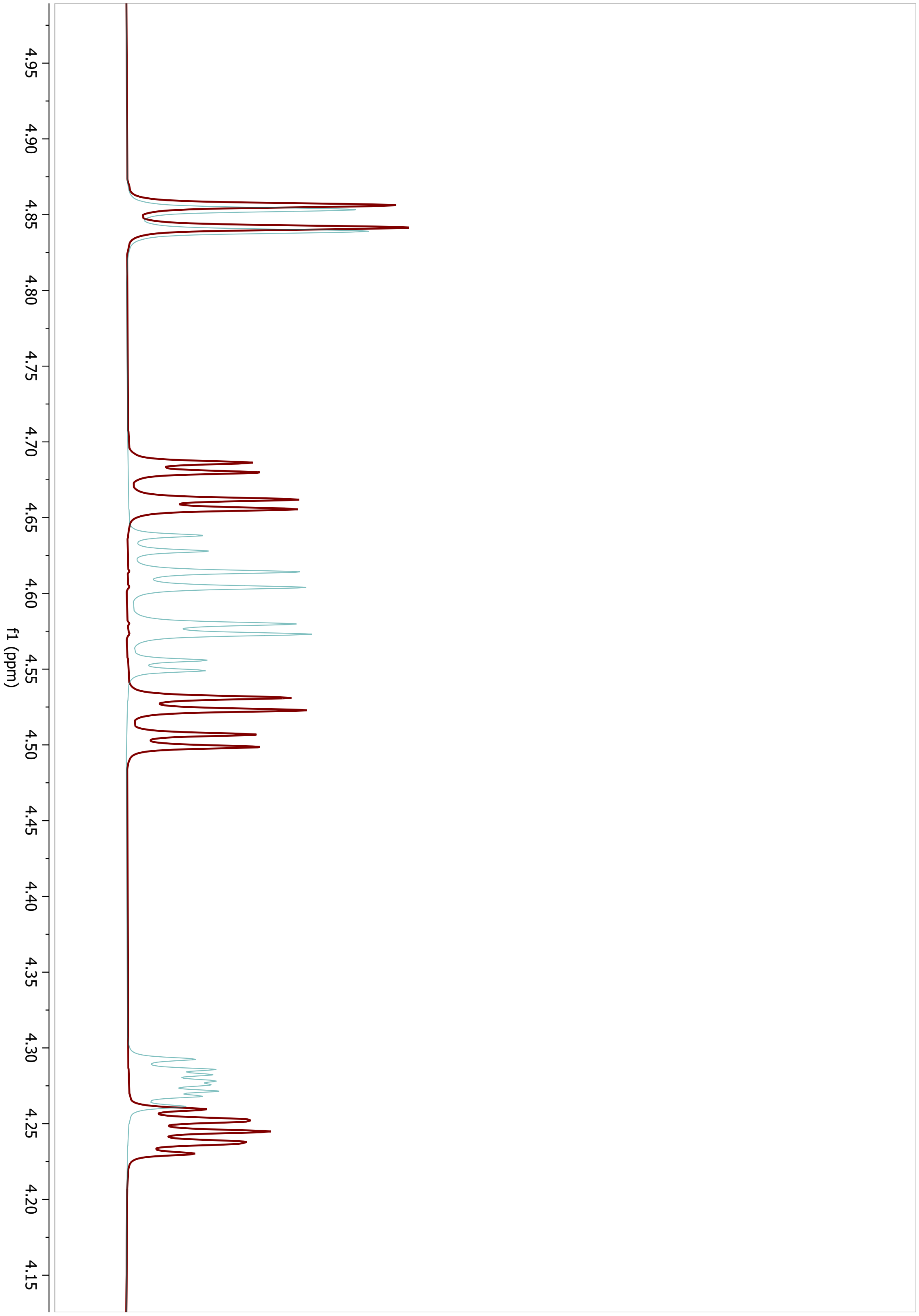

Overlay of (S)-Mosher ester (red) and (R)-Mosher ester (blue) of **8b**
^{1}H NMR (500 MHz, $CDCl_3$)

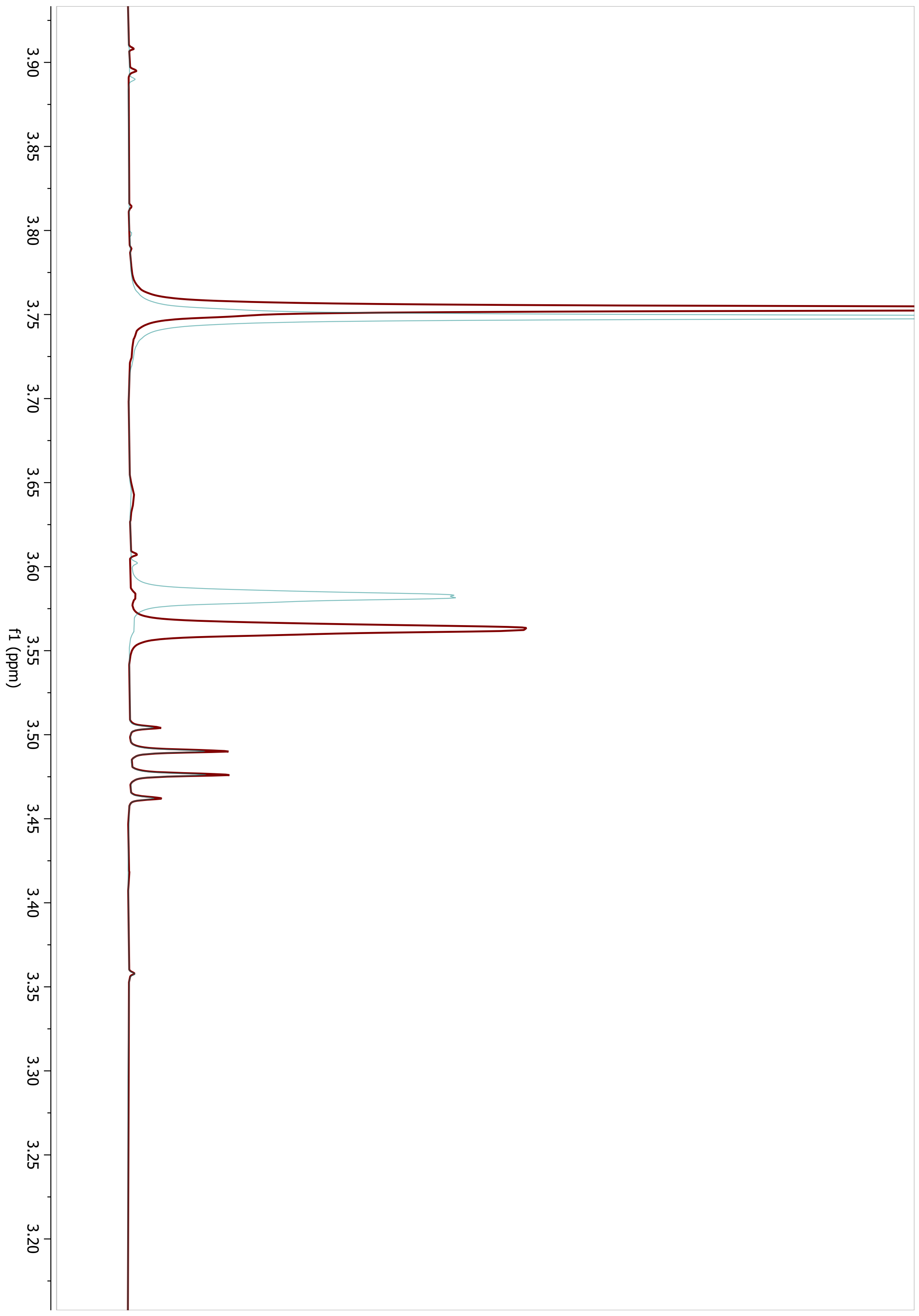

Overlay of (S)-Mosher ester (red) and (R)-Mosher ester (blue) of **8b**
^{1}H NMR (500 MHz, $CDCl_3$)

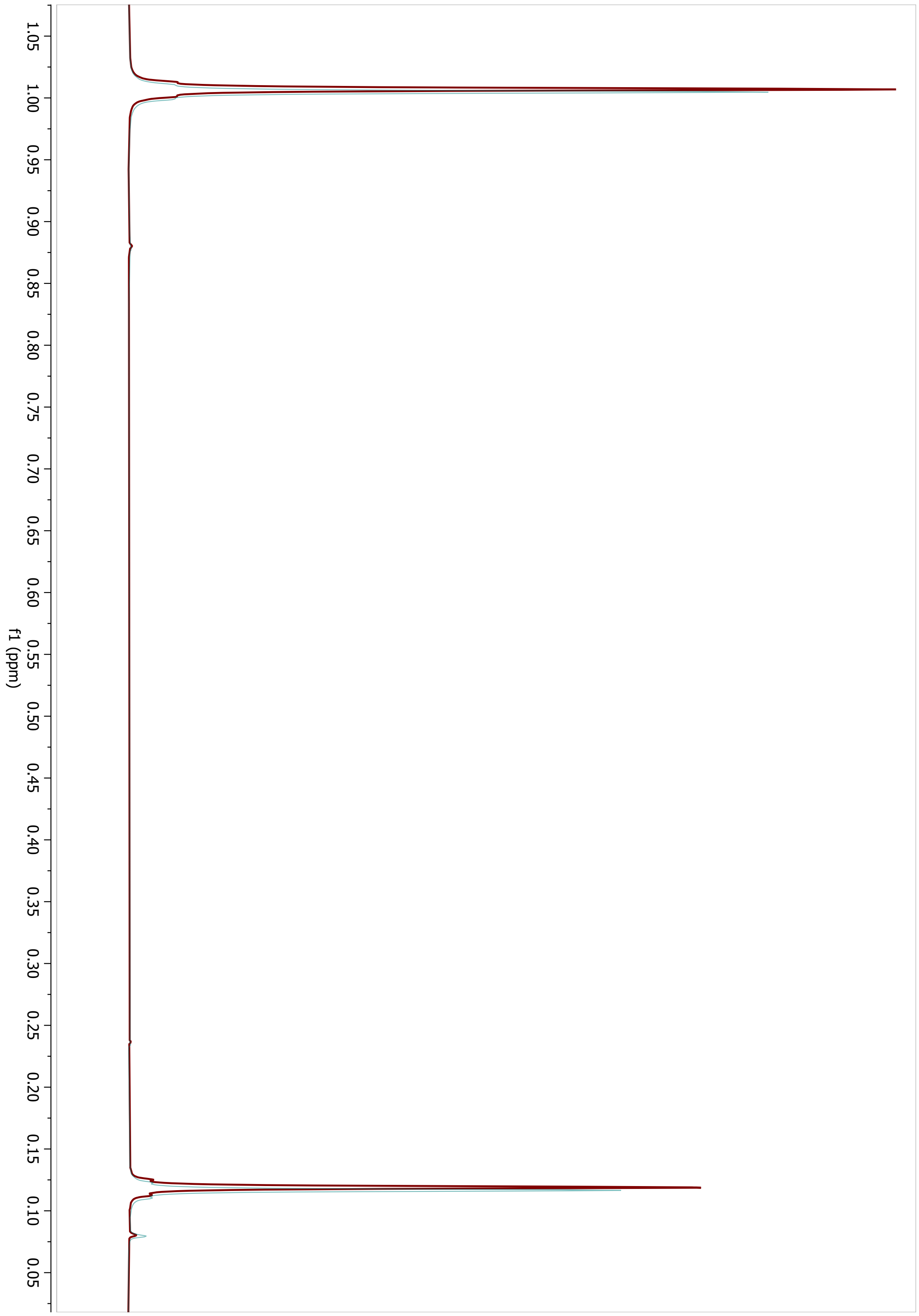

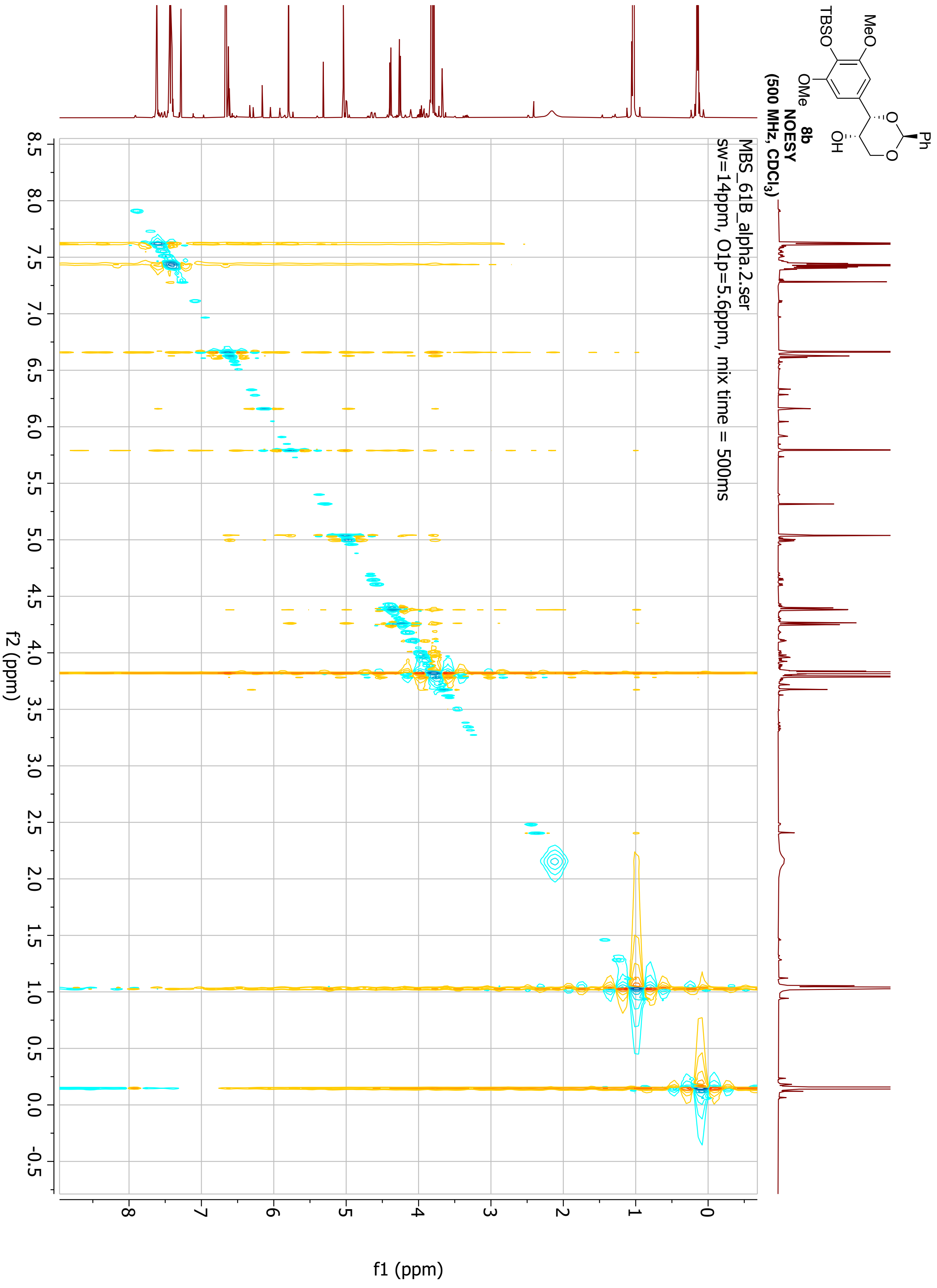
MeO
TBSO
OMe
OH
O
O
Ph
8b
NOESY
(500 MHz, $CDCl_3$)
MBS_61B_alpha.2.ser
sw=14ppm, O1p=5.6ppm, mix time = 500ms
f2 (ppm)
f1 (ppm)

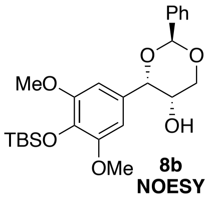

8b
NOESY
(500 MHz, $CDCl_3$)

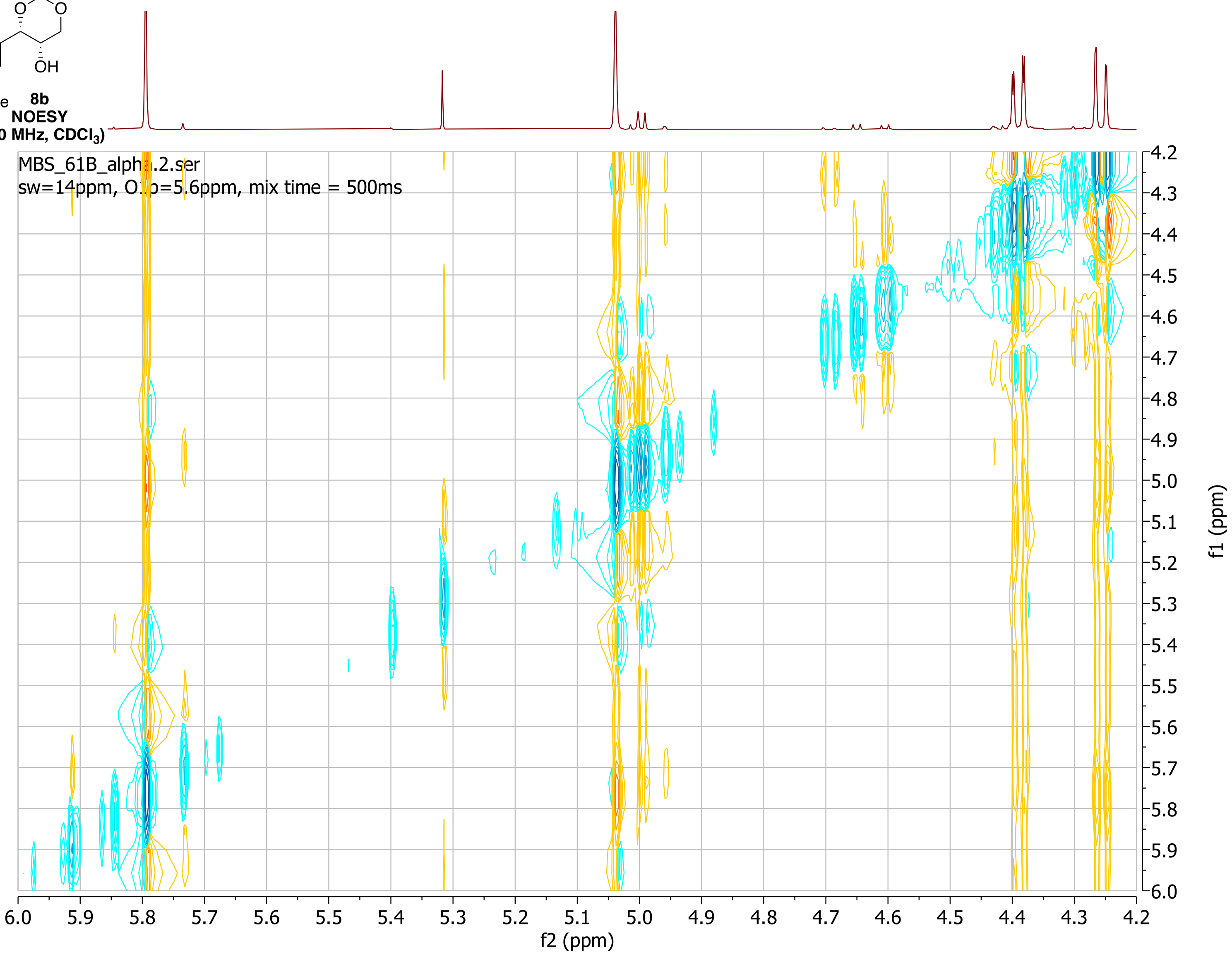

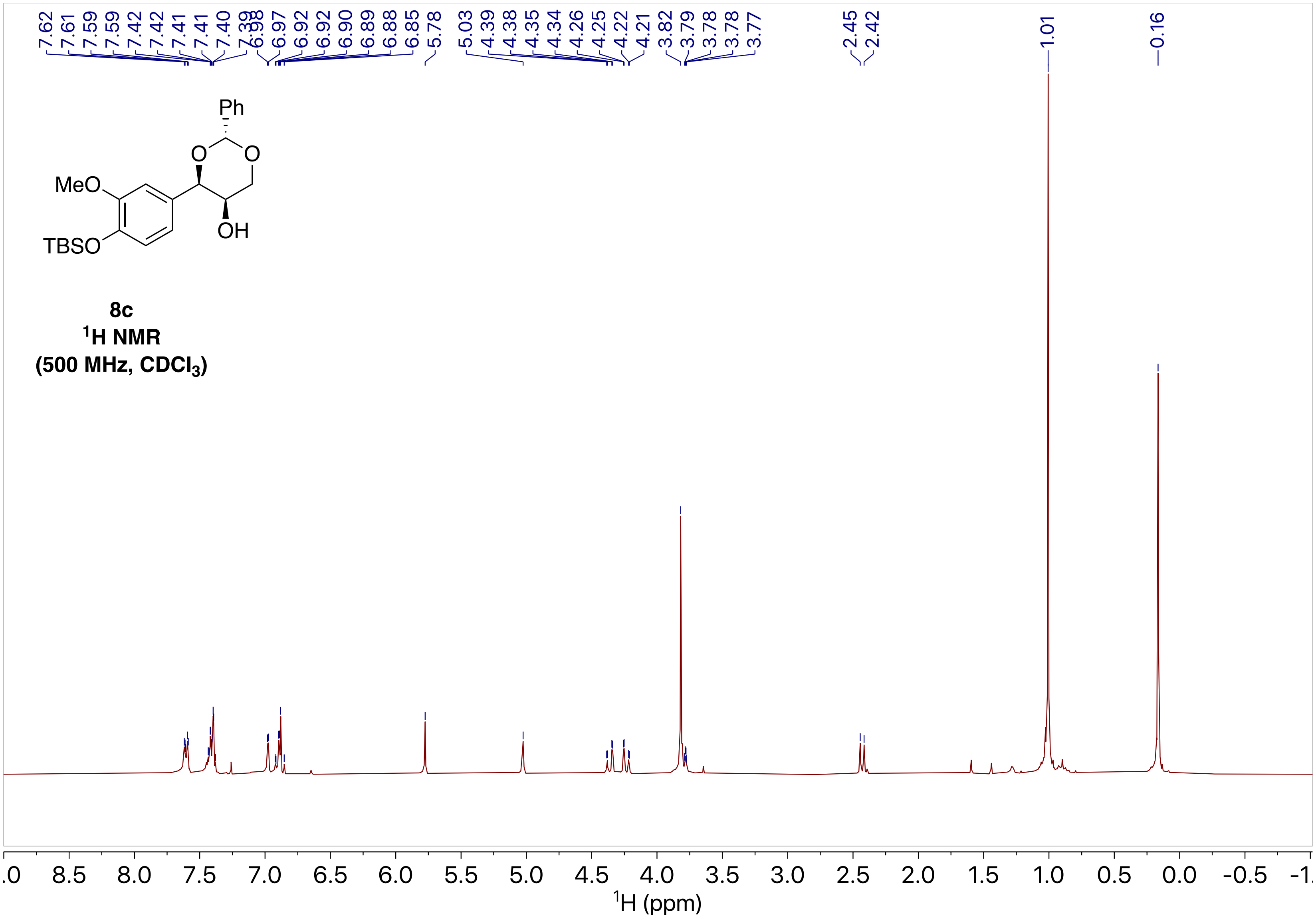
7.62
7.61
7.59
7.59
7.42
7.42
7.41
7.41
7.40
7.39
6.98
6.97
6.92
6.92
6.90
6.89
6.88
6.85
5.78
5.03
4.39
4.38
4.35
4.34
4.26
4.25
4.22
4.21
3.82
3.79
3.78
3.78
3.77
2.45
2.42
1.01
0.16
Ph
O
O
MeO
TBSO
OH
8c
^{1}H NMR
(500 MHz, $CDCl_3$)
.0 8.5 8.0 7.5 7.0 6.5 6.0 5.5 5.0 4.5 4.0 3.5 3.0 2.5 2.0 1.5 1.0 0.5 0.0 -0.5 -1.
^{1}H (ppm)

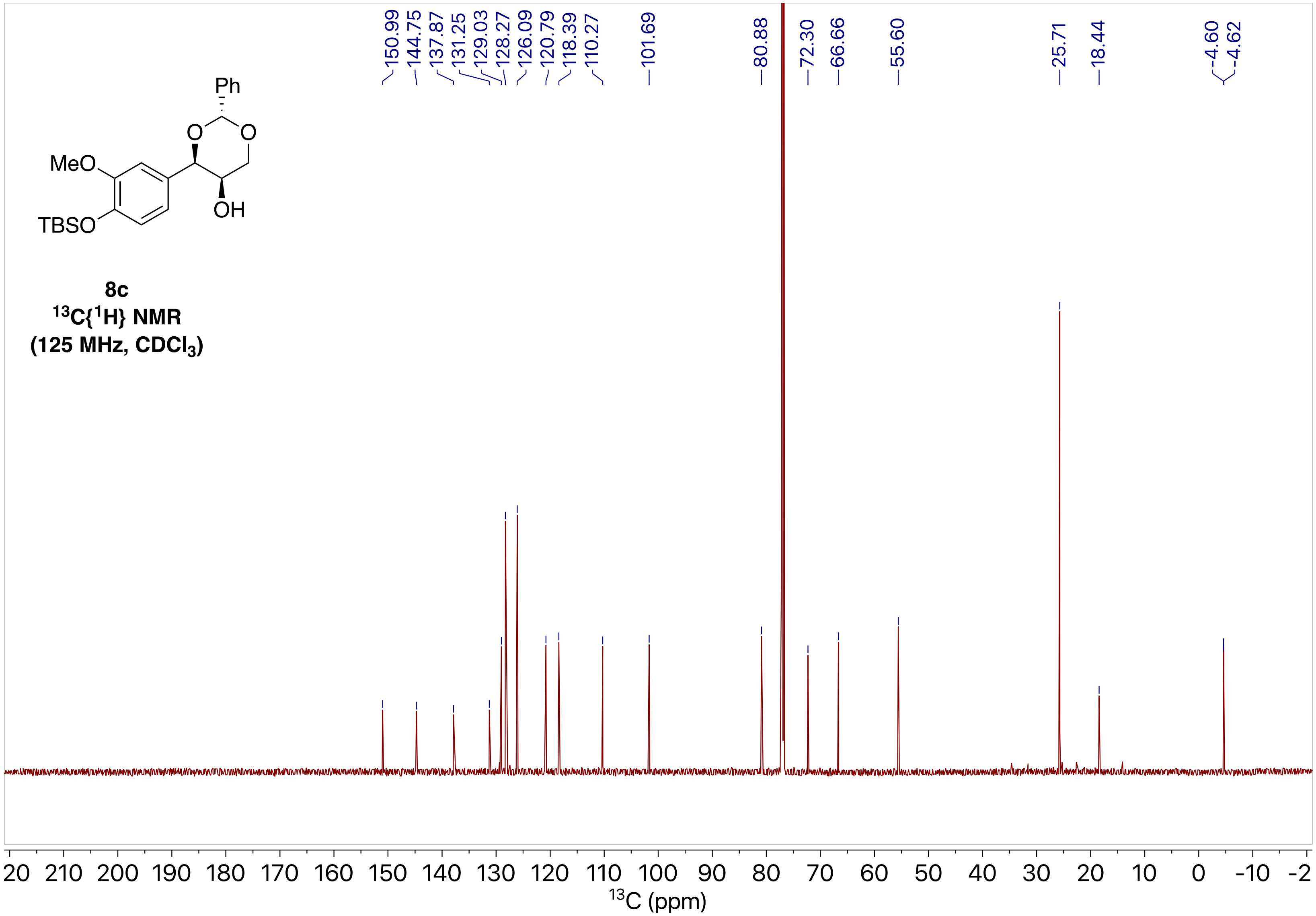

Ph
O
O
MeO
TBSO
OH
8c
13C{1H} NMR
(125 MHz, CDCl3)
150.99
144.75
137.87
131.25
129.03
128.27
126.09
120.79
118.39
110.27
101.69
80.88
72.30
66.66
55.60
25.71
18.44
-4.60
-4.62
20 210 200 190 180 170 160 150 140 130 120 110 100 90 80 70 60 50 40 30 20 10 0 -10 -2
13C (ppm)

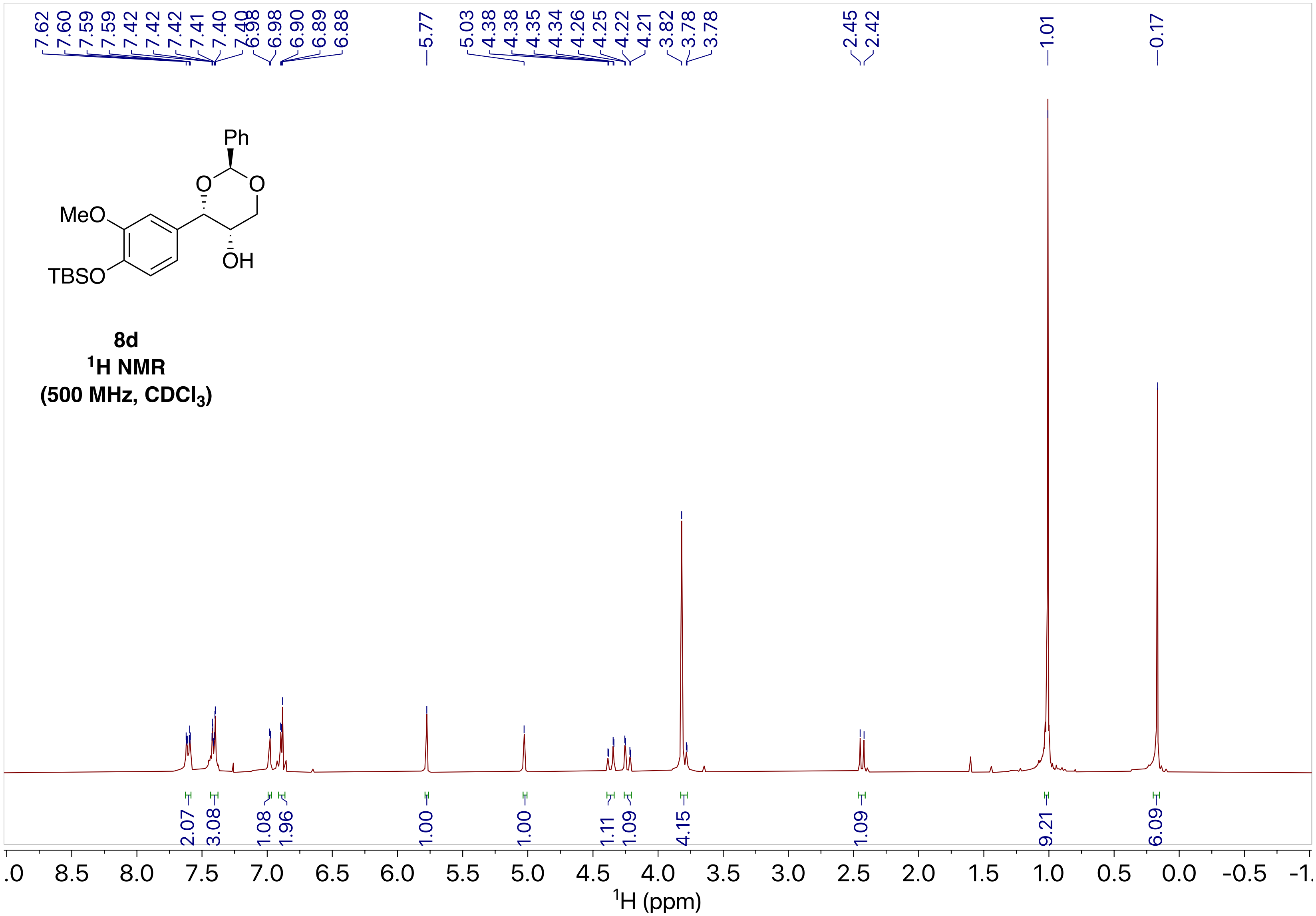

8d
^{1}H NMR
(500 MHz, $CDCl_3$)

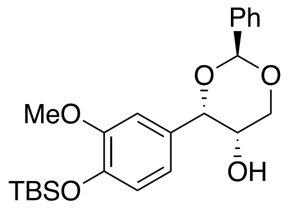

8d
$^{13}C\{^1H\}$ NMR
(125 MHz, $CDCl_3$)

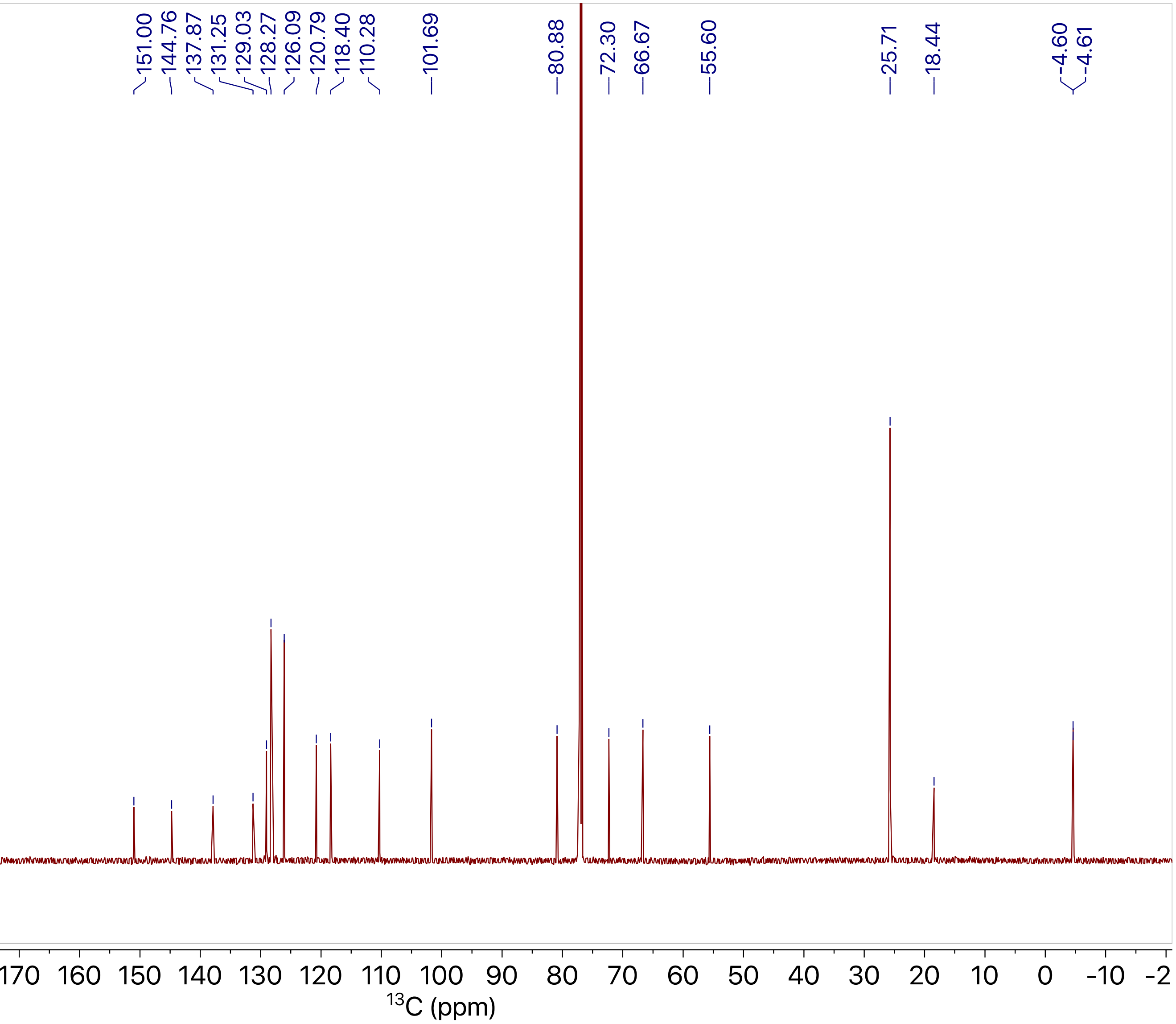

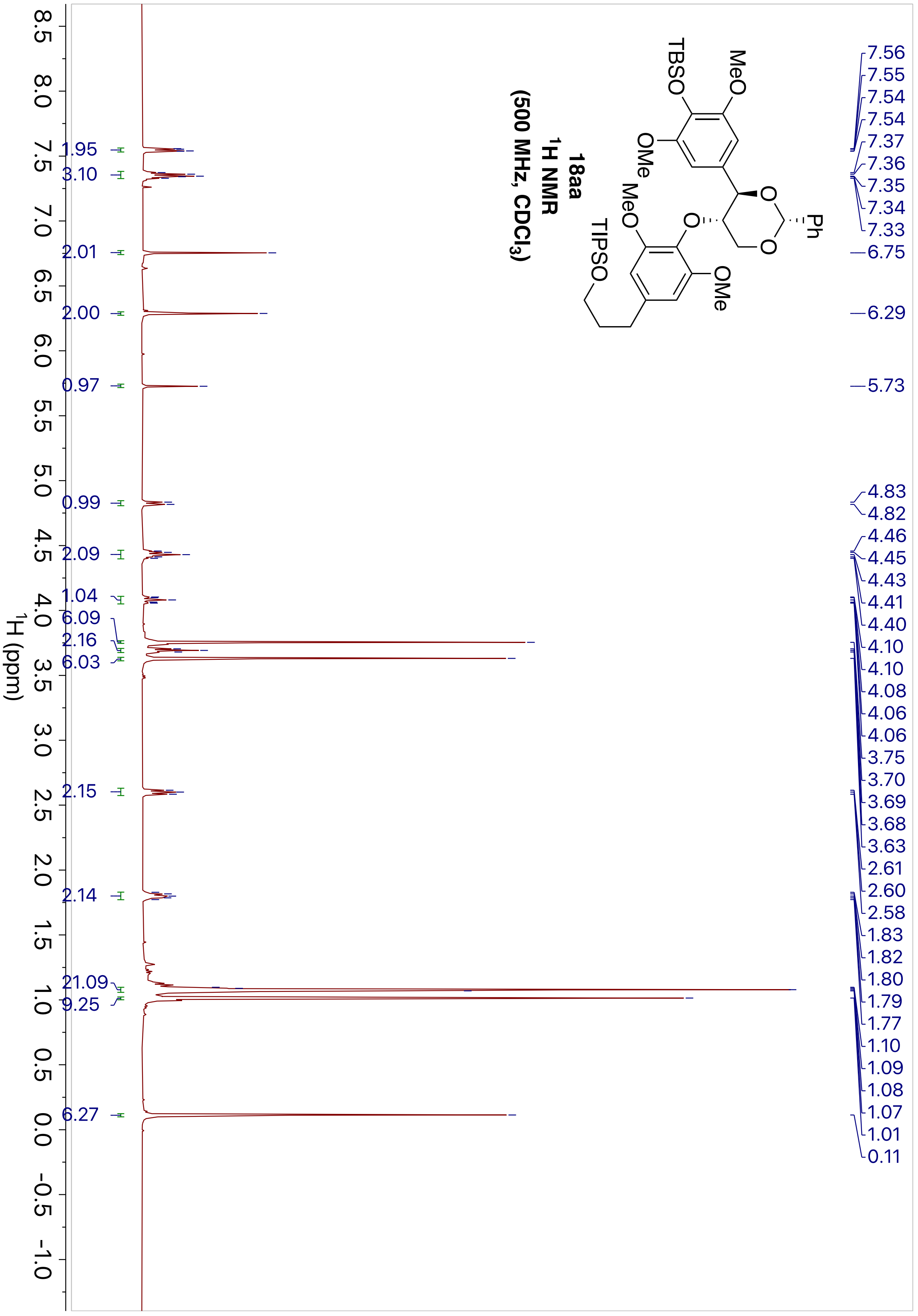
18aa
1H NMR
(500 MHz, CDCl3)
1H (ppm)

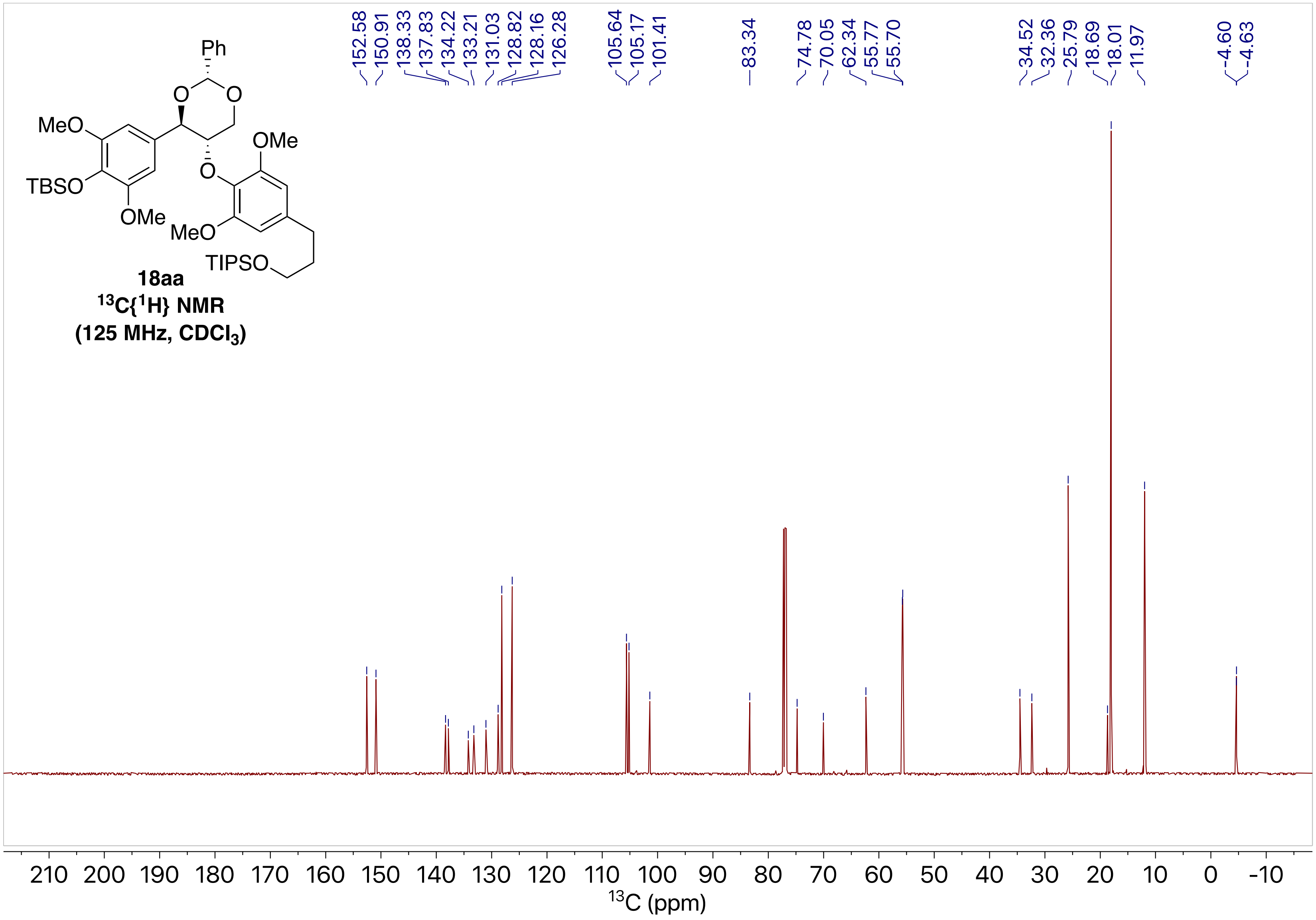

18aa
$^{13}C\{^{1}H\}$ NMR
(125 MHz, $CDCl_3$)

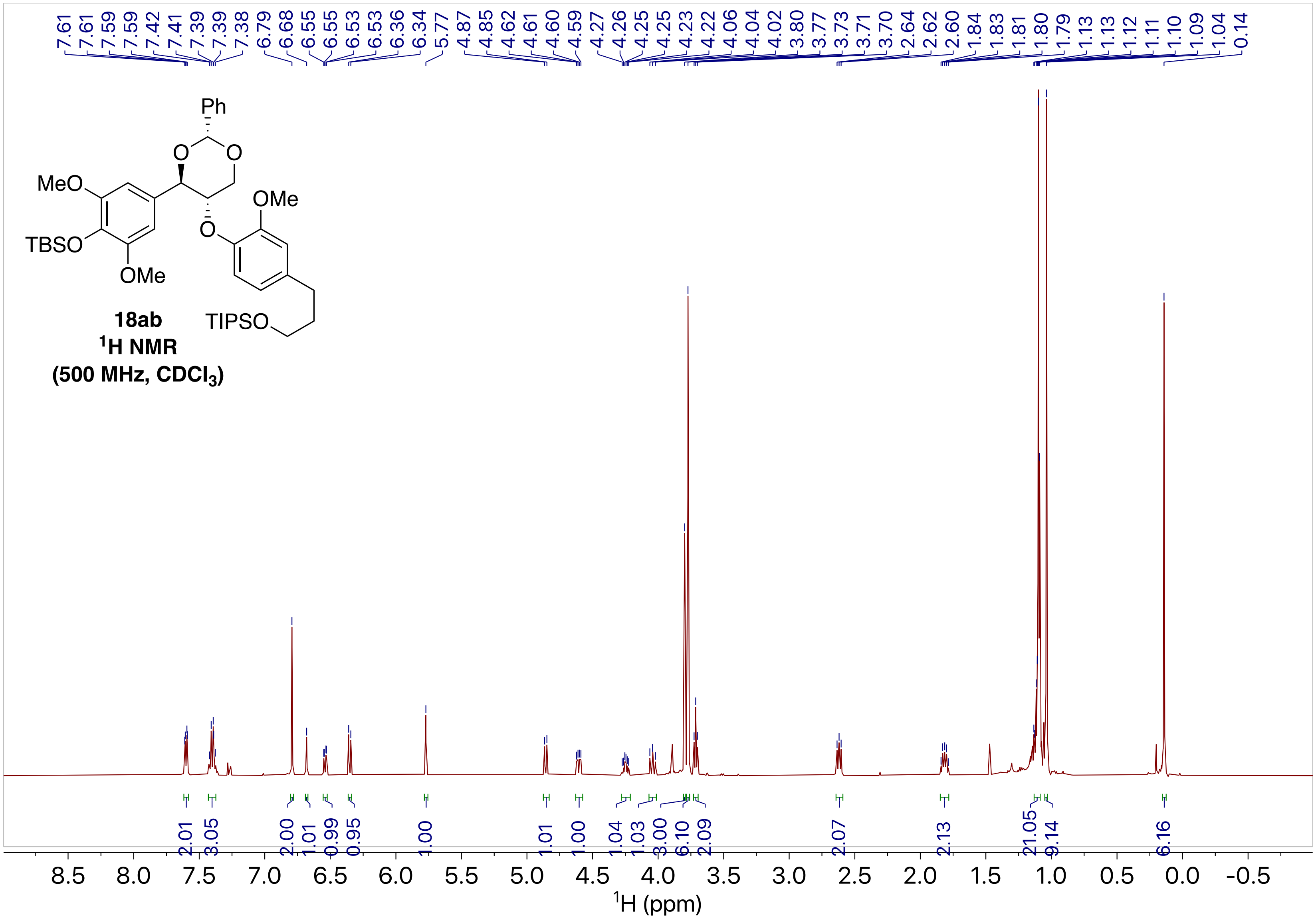
7.61
7.61
7.59
7.59
7.42
7.41
7.39
7.39
7.38
6.79
6.68
6.55
6.55
6.53
6.53
6.36
6.34
5.77
4.87
4.85
4.62
4.61
4.60
4.59
4.27
4.26
4.25
4.25
4.23
4.22
4.06
4.04
4.02
3.80
3.77
3.73
3.71
3.70
2.64
2.62
2.60
1.84
1.83
1.81
1.80
1.79
1.13
1.13
1.12
1.11
1.10
1.09
1.04
0.14
Ph
O
O
MeO
TBSO
OMe
O
OMe
TIPSO
18ab
^{1}H NMR
(500 MHz, $CDCl_3$)
2.01
3.05
2.00
1.01
0.99
0.95
1.00
1.01
1.00
1.04
1.03
3.00
6.10
2.09
2.07
2.13
21.05
9.14
6.16
8.5
8.0
7.5
7.0
6.5
6.0
5.5
5.0
4.5
4.0
3.5
3.0
2.5
2.0
1.5
1.0
0.5
0.0
-0.5
^{1}H (ppm)

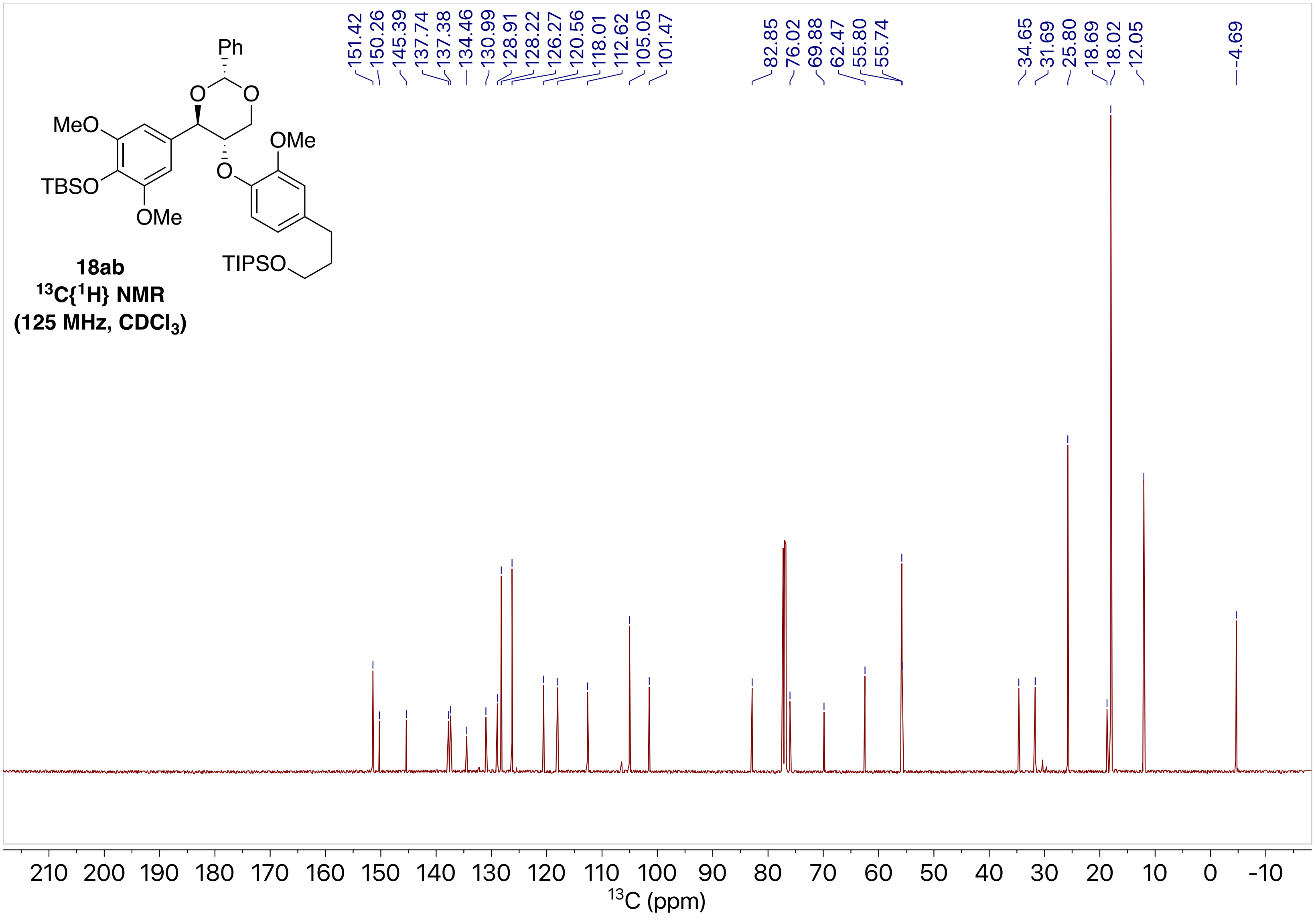
MeO
TBSO
OMe
Ph
O
O
OMe
TIPSO
18ab
$^{13}C\{^{1}H\}$ NMR
(125 MHz, $CDCl_3$)
151.42
150.26
145.39
137.74
137.38
134.46
130.99
128.91
128.22
126.27
120.56
118.01
112.62
105.05
101.47
82.85
76.02
69.88
62.47
55.80
55.74
34.65
31.69
25.80
18.69
18.02
12.05
-4.69
210 200 190 180 170 160 150 140 130 120 110 100 90 80 70 60 50 40 30 20 10 0 -10
^{13}C (ppm)

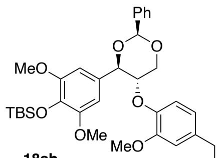

18ab
NOESY
(500 MHz, $CDCl_3$)

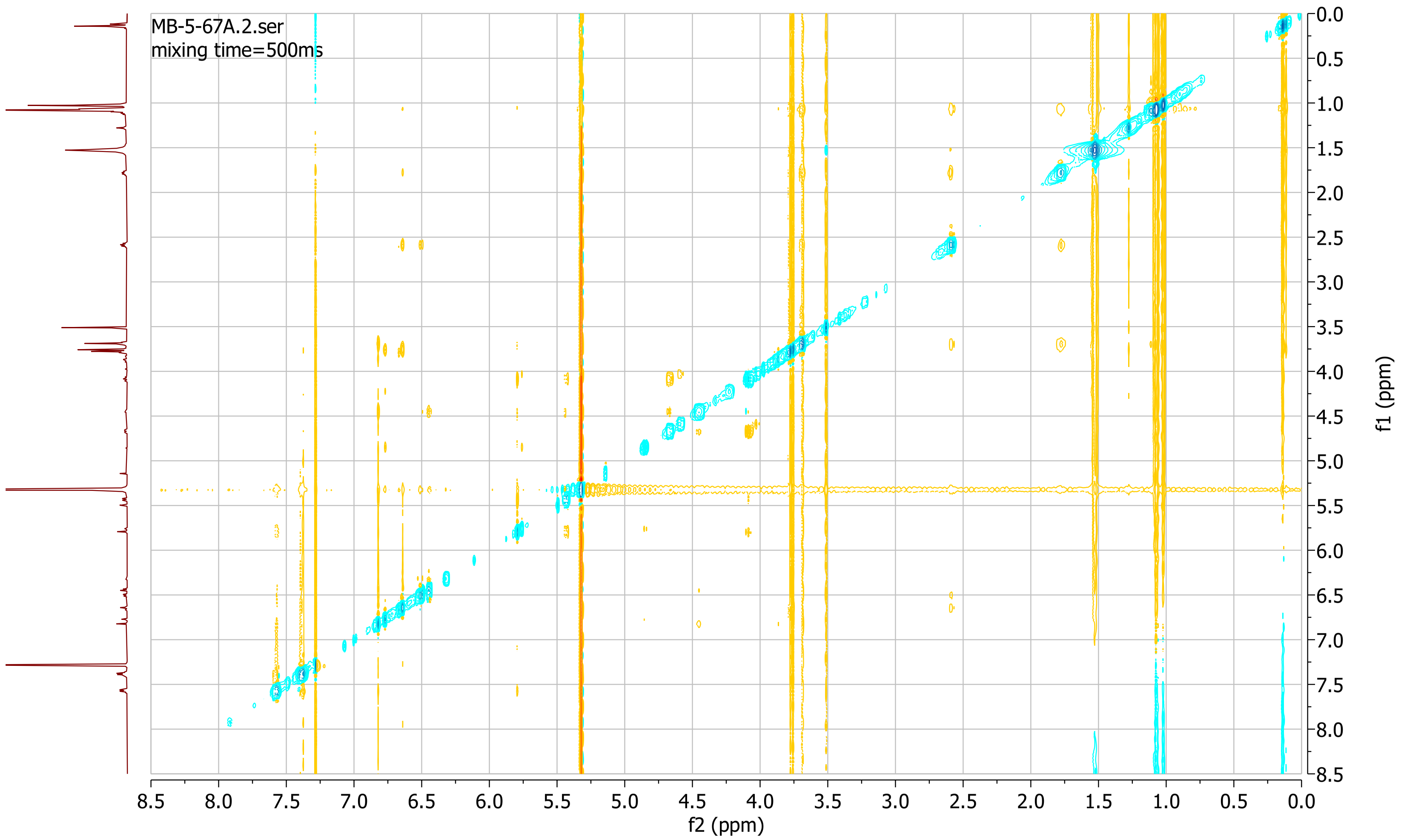

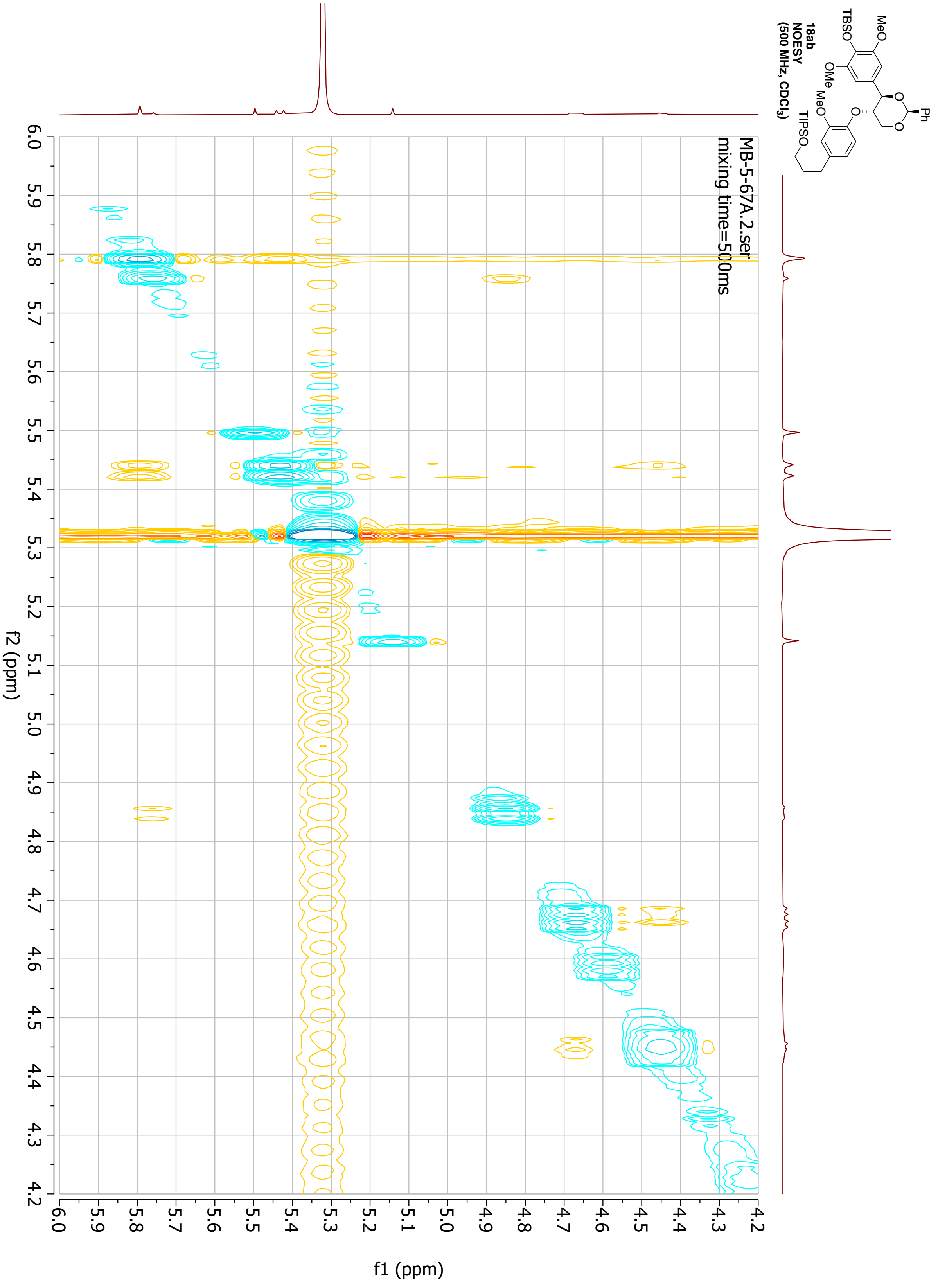
18ab
NOESY
(500 MHz, CDCl3)
MB-5-67A.2.ser
mixing time=500ms
f2 (ppm)
f1 (ppm)

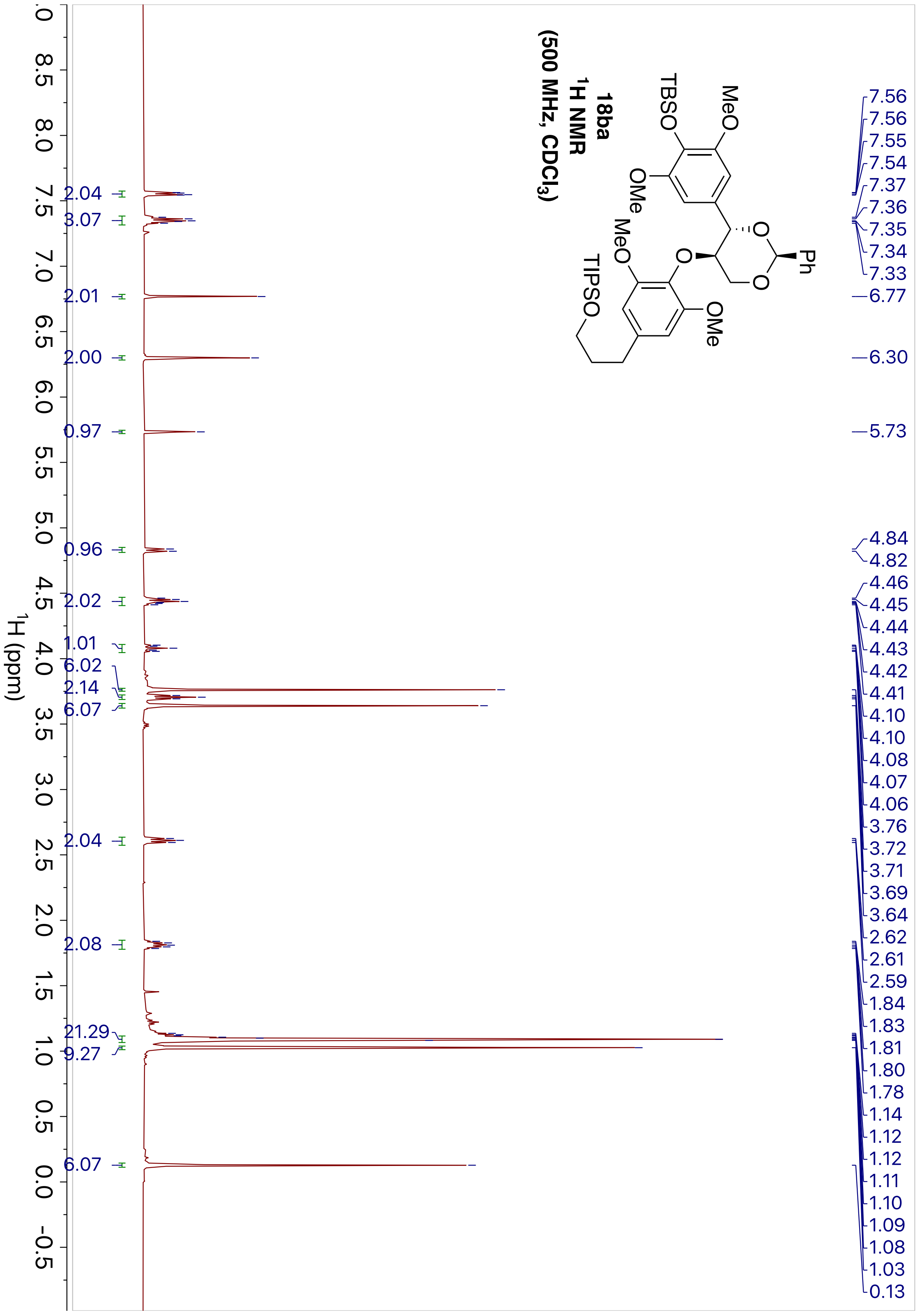
18ba
1H NMR
(500 MHz, CDCl3)
MeO
TBSO
OMe
MeO
TIPSO
OMe
Ph
O
O
O
1H (ppm)
7.56
7.56
7.55
7.54
7.37
7.36
7.35
7.34
7.33
6.77
6.30
5.73
4.84
4.82
4.46
4.45
4.44
4.43
4.42
4.41
4.10
4.10
4.08
4.07
4.06
3.76
3.72
3.71
3.69
3.64
2.62
2.61
2.59
1.84
1.83
1.81
1.80
1.78
1.14
1.12
1.12
1.11
1.10
1.09
1.08
1.03
0.13
2.04
3.07
2.01
2.00
0.97
0.96
2.02
1.01
6.02
2.14
6.07
2.04
2.08
21.29
9.27
6.07

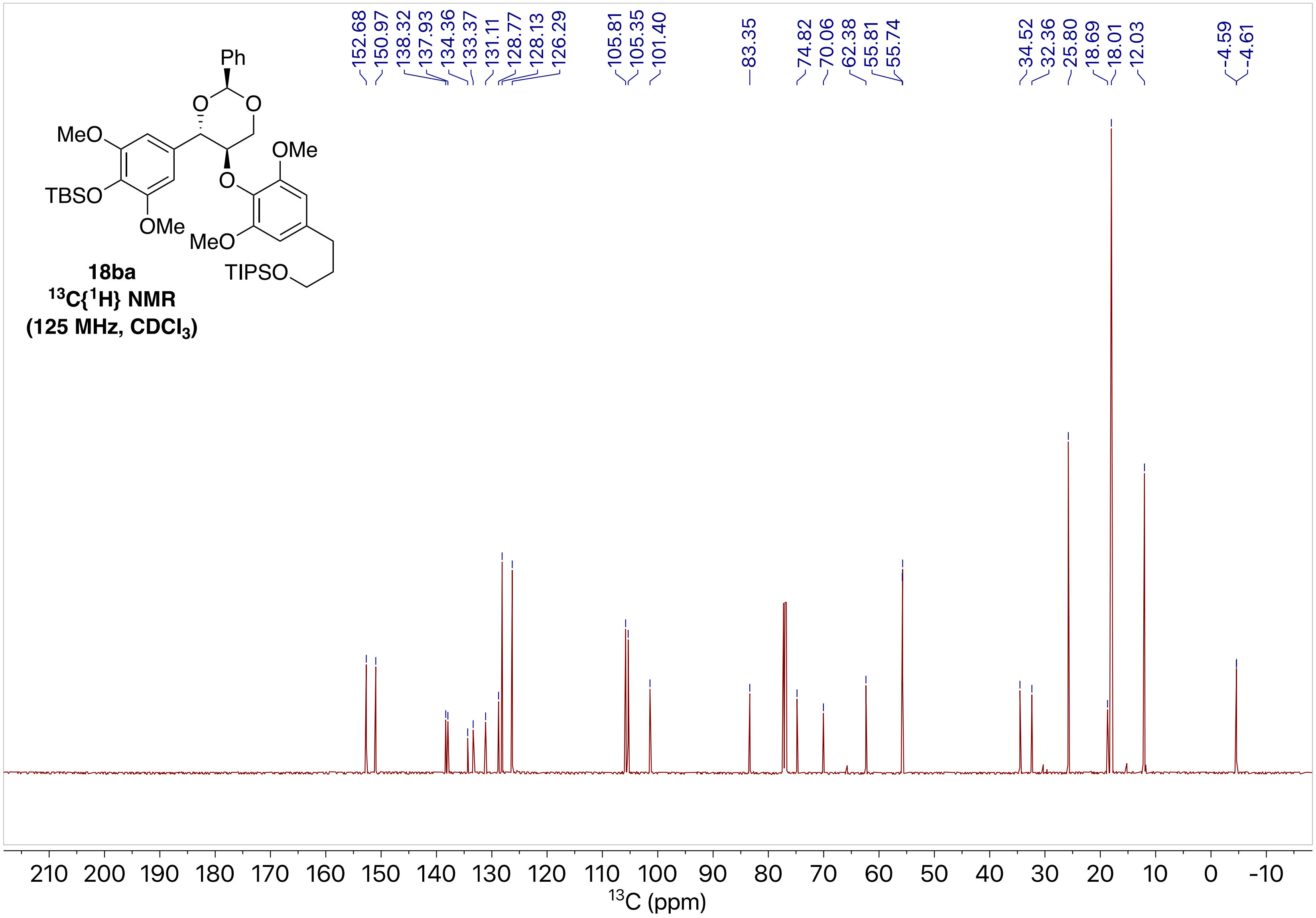
Ph
MeO
TBSO
OMe
OMe
MeO
TIPSO
18ba
$^{13}C\{^{1}H\}$ NMR
(125 MHz, $CDCl_3$)
152.68
150.97
138.32
137.93
134.36
133.37
131.11
128.77
128.13
126.29
105.81
105.35
101.40
83.35
74.82
70.06
62.38
55.81
55.74
34.52
32.36
25.80
18.69
18.01
12.03
-4.59
-4.61
210 200 190 180 170 160 150 140 130 120 110 100 90 80 70 60 50 40 30 20 10 0 -10
^{13}C (ppm)

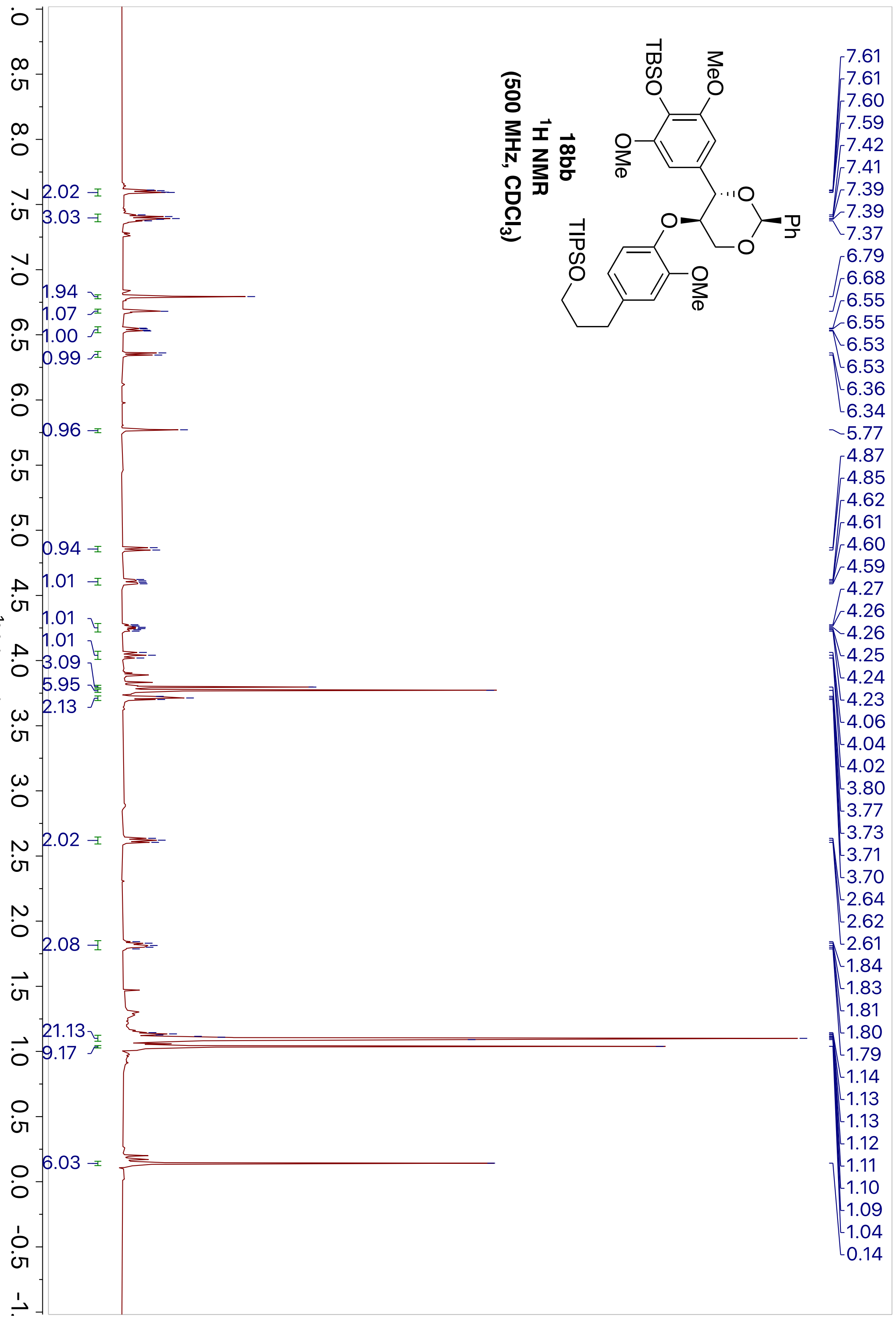
18bb
1H NMR
(500 MHz, CDCl3)
MeO
TBSO
OMe
Ph
TIPSO
OMe
1H (ppm)

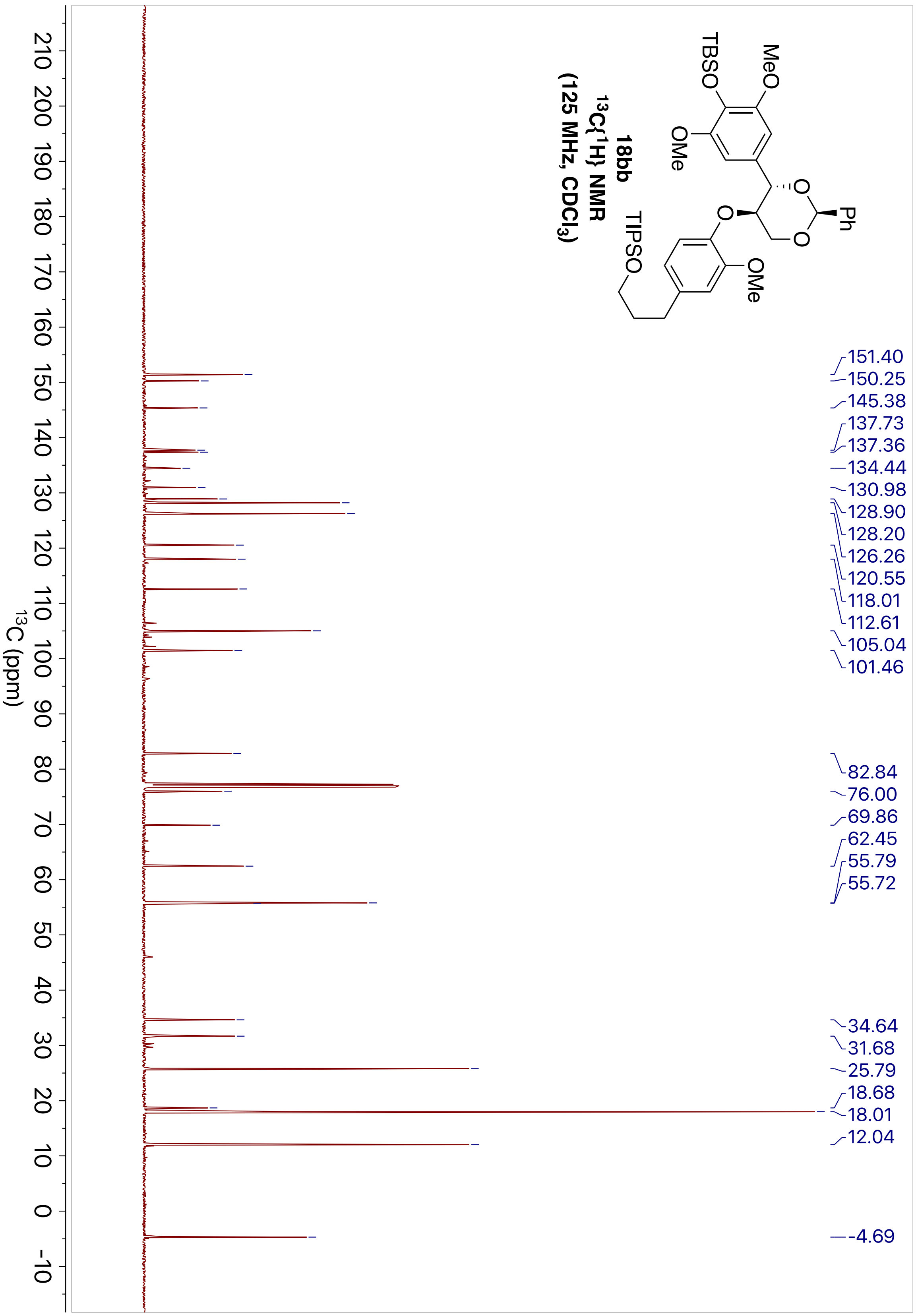
18bb
$^{13}C\{^1H\}$ NMR
(125 MHz, $CDCl_3$)
MeO
TBSO
OMe
O
Ph
OMe
TIPSO
151.40
150.25
145.38
137.73
137.36
134.44
130.98
128.90
128.20
126.26
120.55
118.01
112.61
105.04
101.46
82.84
76.00
69.86
62.45
55.79
55.72
34.64
31.68
25.79
18.68
18.01
12.04
-4.69
210 200 190 180 170 160 150 140 130 120 110 100 90 80 70 60 50 40 30 20 10 0 -10
^{13}C (ppm)

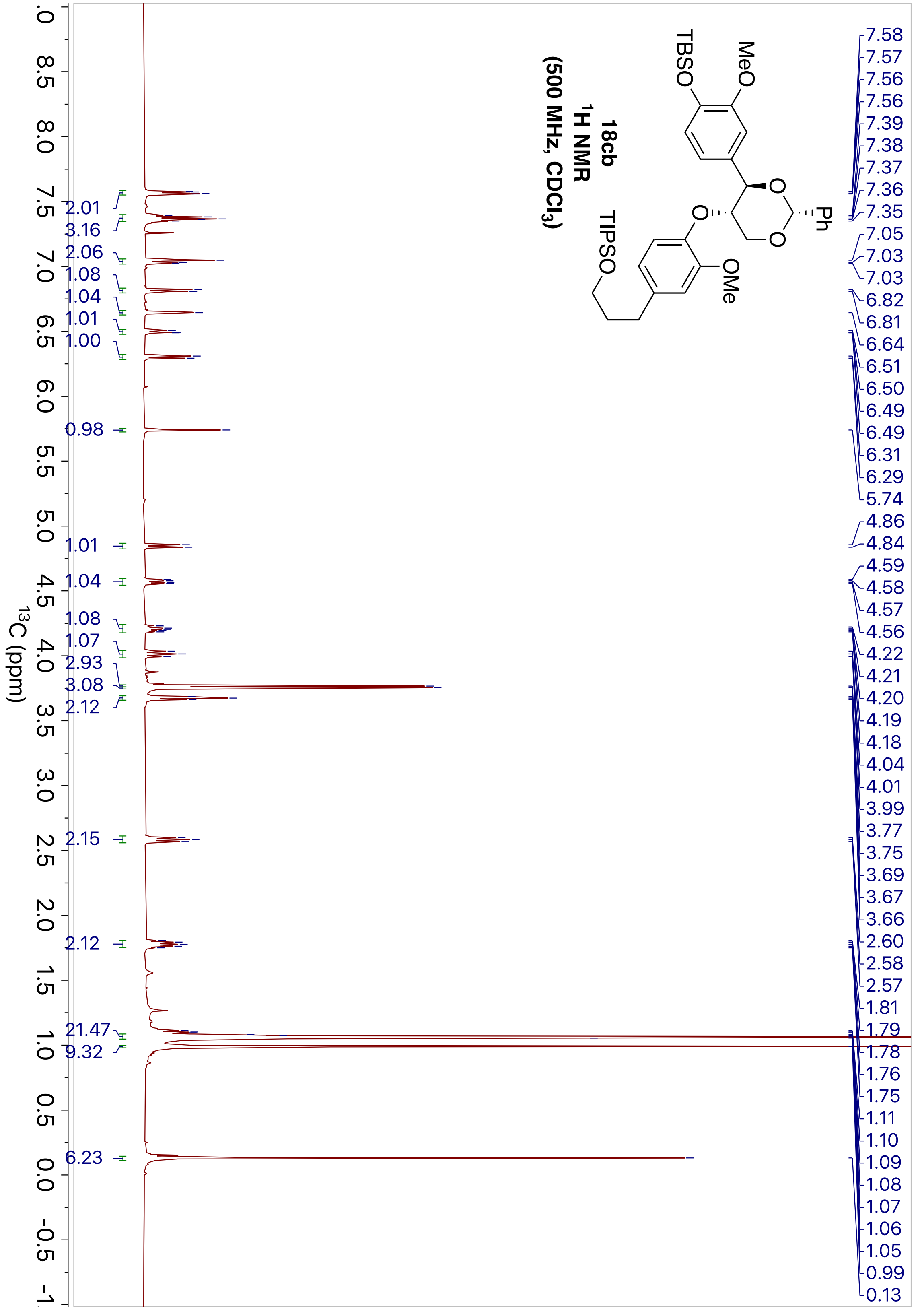

18cb
1H NMR
(500 MHz, CDCl3)
MeO
TBSO
Ph
O
OMe
TIPSO
7.58
7.57
7.56
7.56
7.39
7.38
7.37
7.36
7.35
7.05
7.03
7.03
6.82
6.81
6.64
6.51
6.50
6.49
6.49
6.31
6.29
5.74
4.86
4.84
4.59
4.58
4.57
4.56
4.22
4.21
4.20
4.19
4.18
4.04
4.01
3.99
3.77
3.75
3.69
3.67
3.66
2.60
2.58
2.57
1.81
1.79
1.78
1.76
1.75
1.11
1.10
1.09
1.08
1.07
1.06
1.05
0.99
0.13
2.01
3.16
2.06
1.08
1.04
1.01
1.00
0.98
1.01
1.04
1.08
1.07
2.93
3.08
2.12
2.15
2.12
21.47
9.32
6.23
13C (ppm)

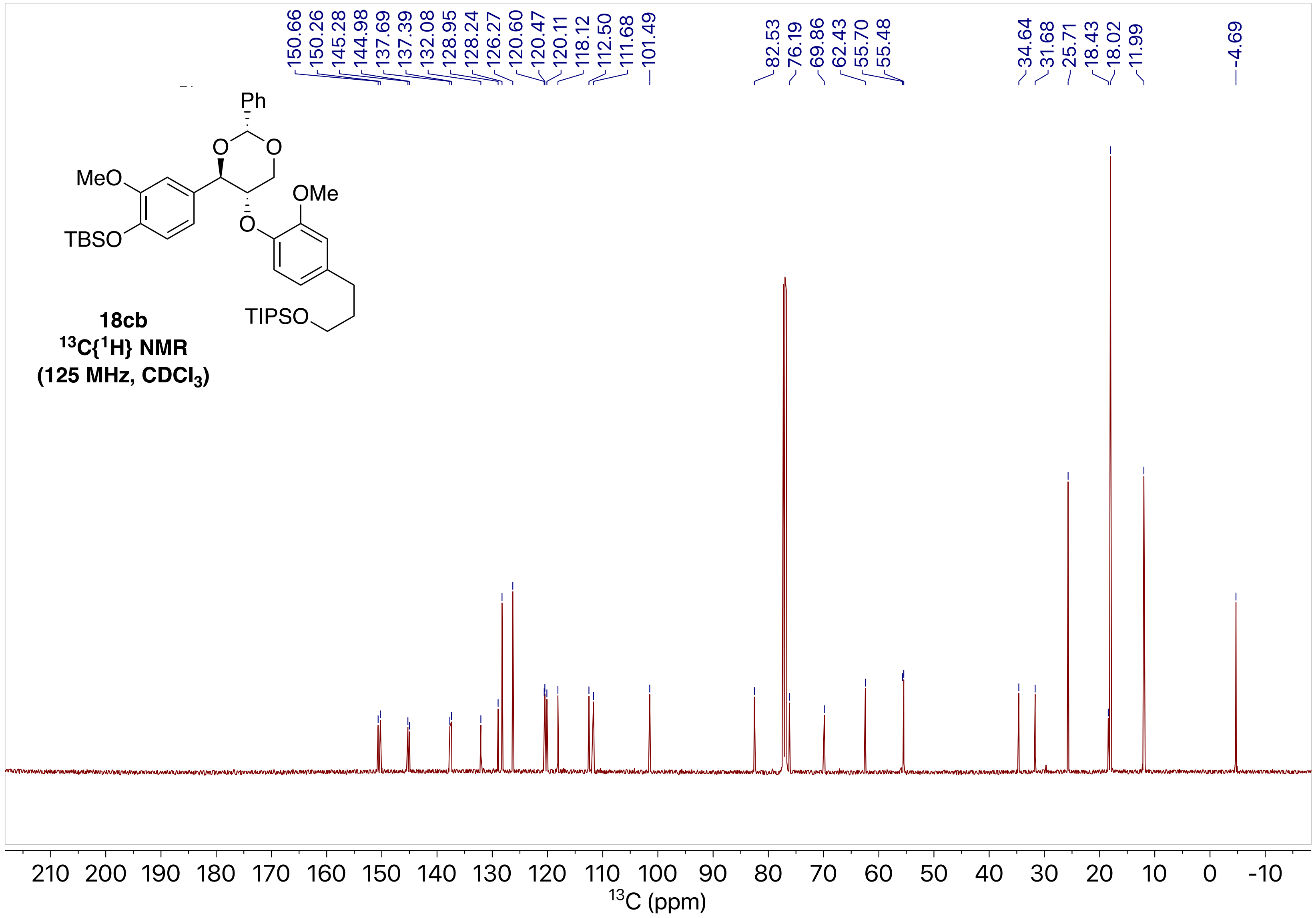
Ph
O
O
MeO
TBSO
OMe
O
TIPSO
18cb
$^{13}C\{^{1}H\}$ NMR
(125 MHz, $CDCl_3$)
150.66
150.26
145.28
144.98
137.69
137.39
132.08
128.95
128.24
126.27
120.60
120.47
120.11
118.12
112.50
111.68
101.49
82.53
76.19
69.86
62.43
55.70
55.48
34.64
31.68
25.71
18.43
18.02
11.99
-4.69
210 200 190 180 170 160 150 140 130 120 110 100 90 80 70 60 50 40 30 20 10 0 -10
^{13}C (ppm)

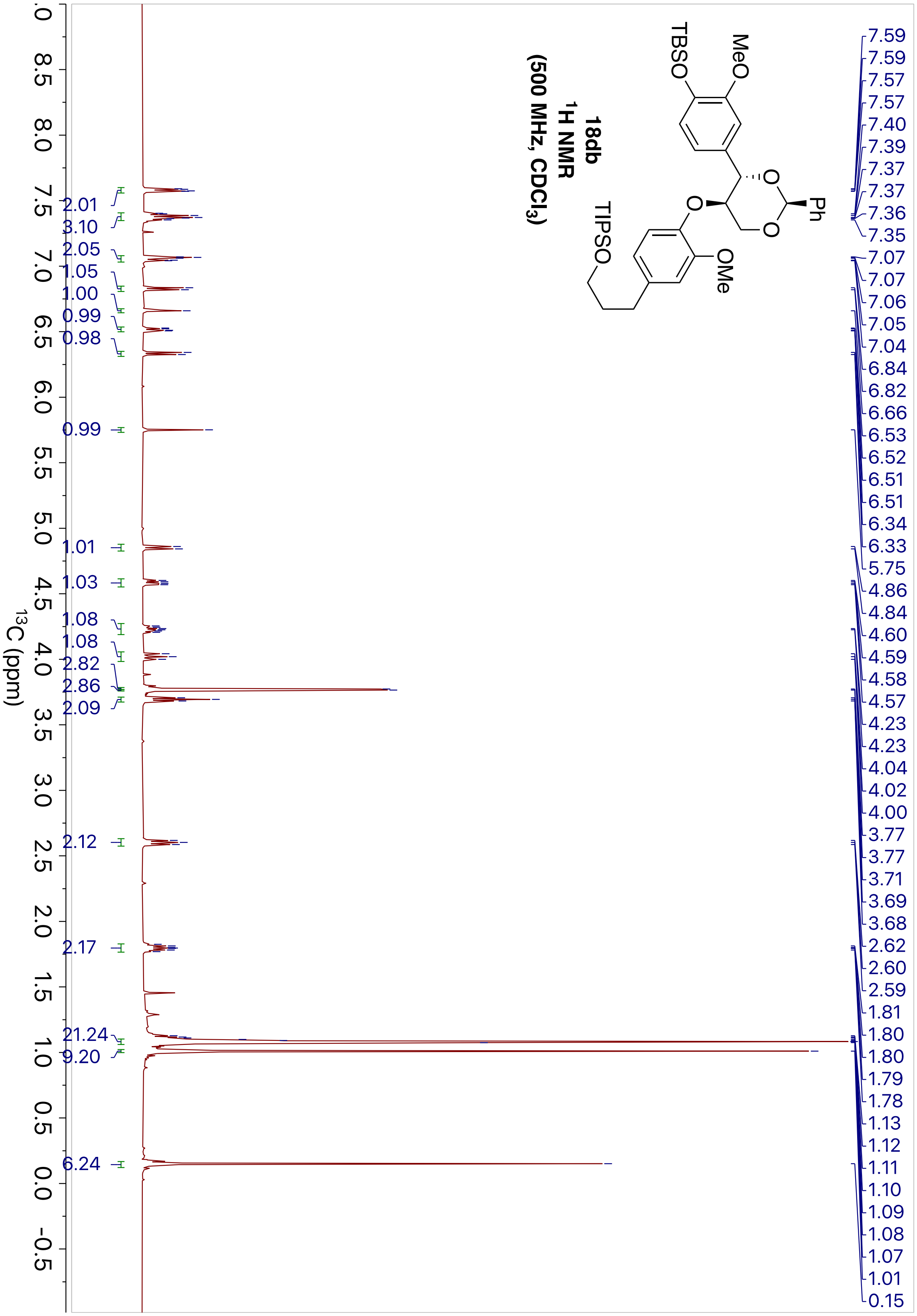
18db
^{1}H NMR
(500 MHz, $CDCl_3$)
MeO
TBSO
Ph
O
O
O
OMe
TIPSO
7.59
7.59
7.57
7.57
7.40
7.39
7.37
7.37
7.36
7.35
7.07
7.07
7.06
7.05
7.04
6.84
6.82
6.66
6.53
6.52
6.51
6.51
6.34
6.33
5.75
4.86
4.84
4.60
4.59
4.58
4.57
4.23
4.23
4.04
4.02
4.00
3.77
3.77
3.71
3.69
3.68
2.62
2.60
2.59
1.81
1.80
1.80
1.79
1.78
1.13
1.12
1.11
1.10
1.09
1.08
1.07
1.01
0.15
2.01
3.10
2.05
1.05
1.00
0.99
0.98
0.99
1.01
1.03
1.08
1.08
2.82
2.86
2.09
2.12
2.17
21.24
9.20
6.24
.0
8.5
8.0
7.5
7.0
6.5
6.0
5.5
5.0
4.5
4.0
3.5
3.0
2.5
2.0
1.5
1.0
0.5
0.0
-0.5
^{13}C (ppm)

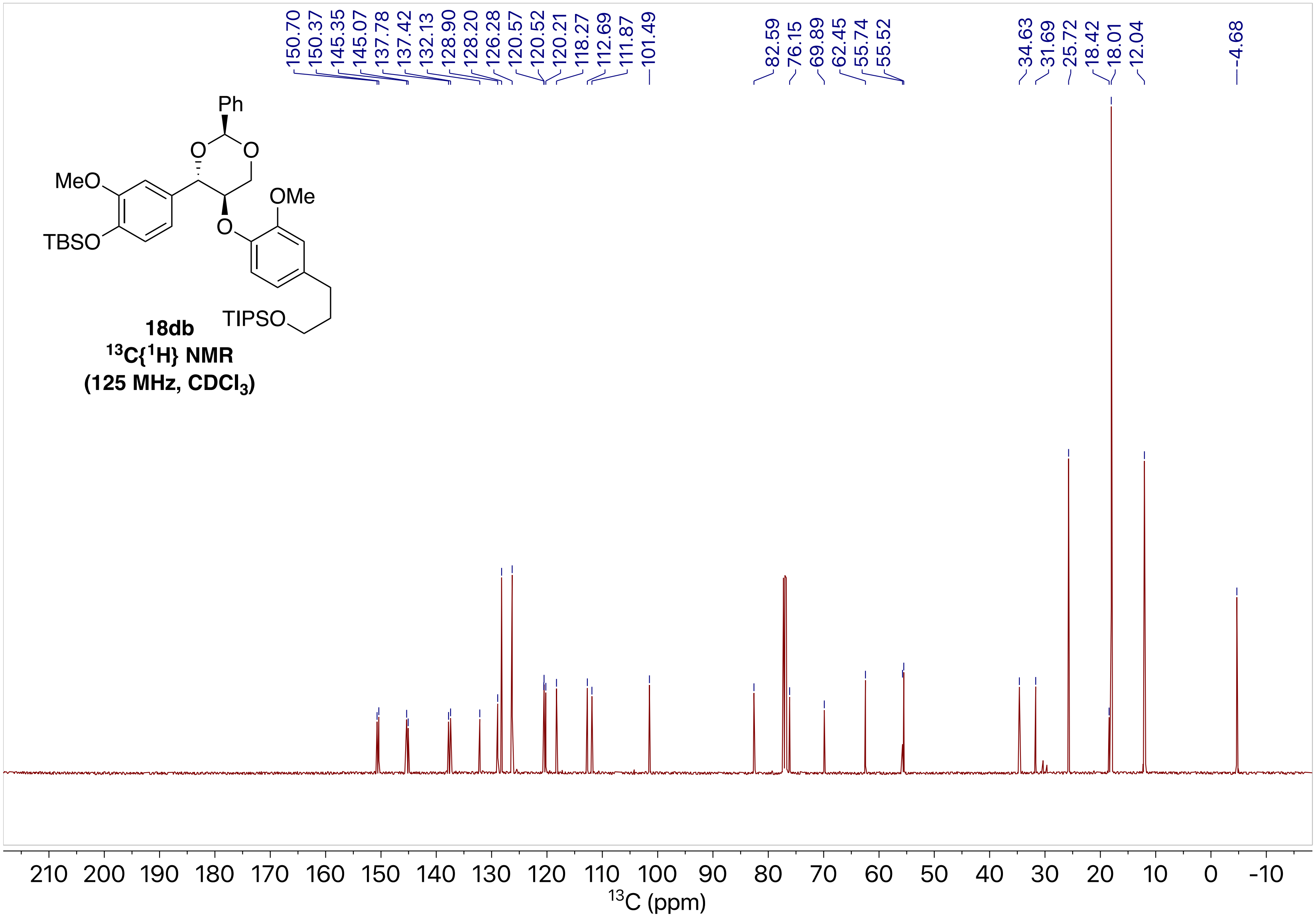
Ph
MeO
TBSO
OMe
TIPSO
18db
$^{13}C\{^{1}H\}$ NMR
(125 MHz, $CDCl_3$)
150.70
150.37
145.35
145.07
137.78
137.42
132.13
128.90
128.20
126.28
120.57
120.52
120.21
118.27
112.69
111.87
101.49
82.59
76.15
69.89
62.45
55.74
55.52
34.63
31.69
25.72
18.42
18.01
12.04
-4.68
210 200 190 180 170 160 150 140 130 120 110 100 90 80 70 60 50 40 30 20 10 0 -10
^{13}C (ppm)

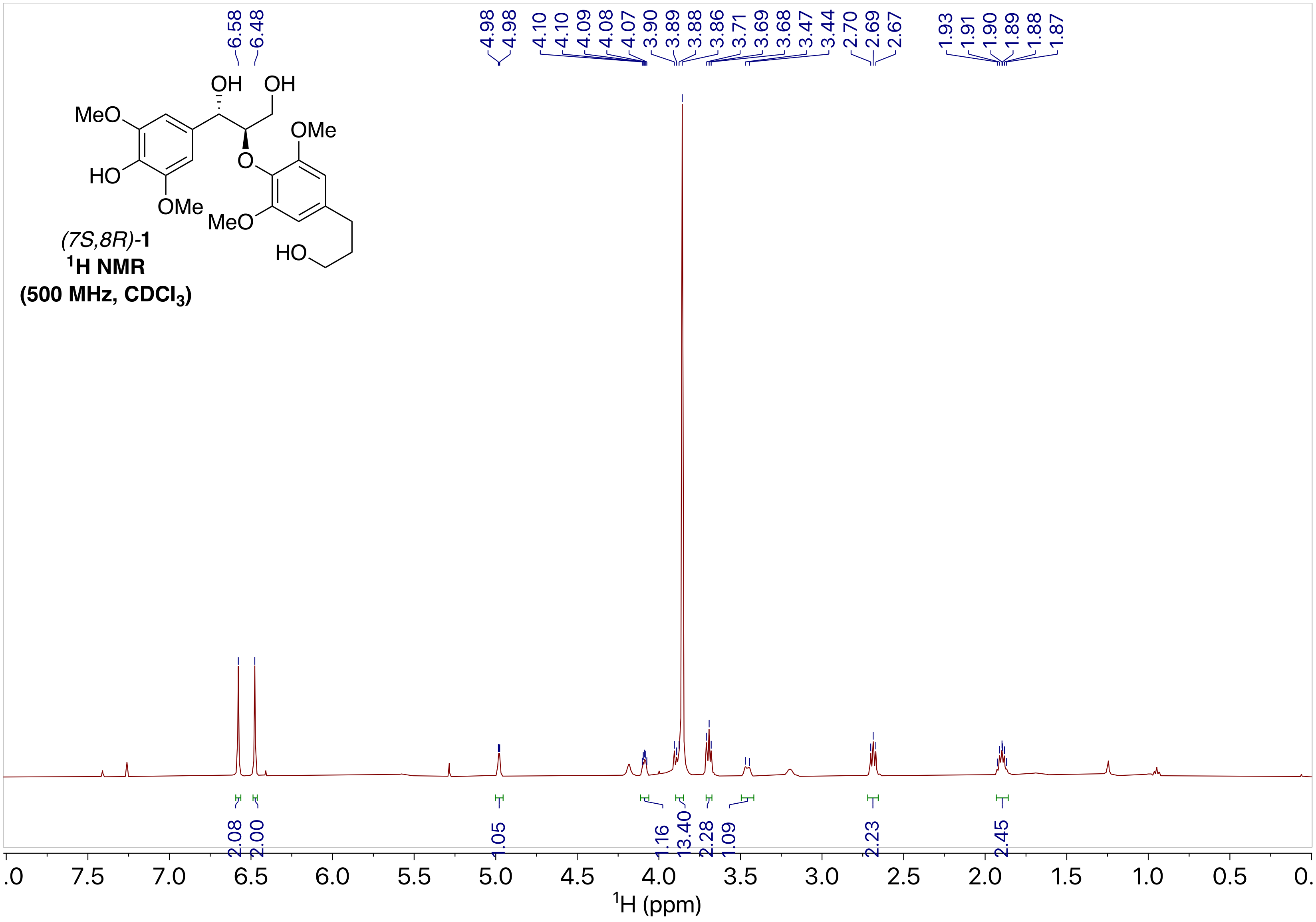
(7S,8R)-1
¹H NMR
(500 MHz, CDCl₃)
MeO
HO
OMe
OH
OH
O
OMe
MeO
HO
6.58
6.48
4.98
4.98
4.10
4.10
4.09
4.08
4.07
3.90
3.89
3.88
3.86
3.71
3.69
3.68
3.47
3.44
2.70
2.69
2.67
1.93
1.91
1.90
1.89
1.88
1.87
2.08
2.00
1.05
1.16
13.40
2.28
1.09
2.23
2.45
¹H (ppm)

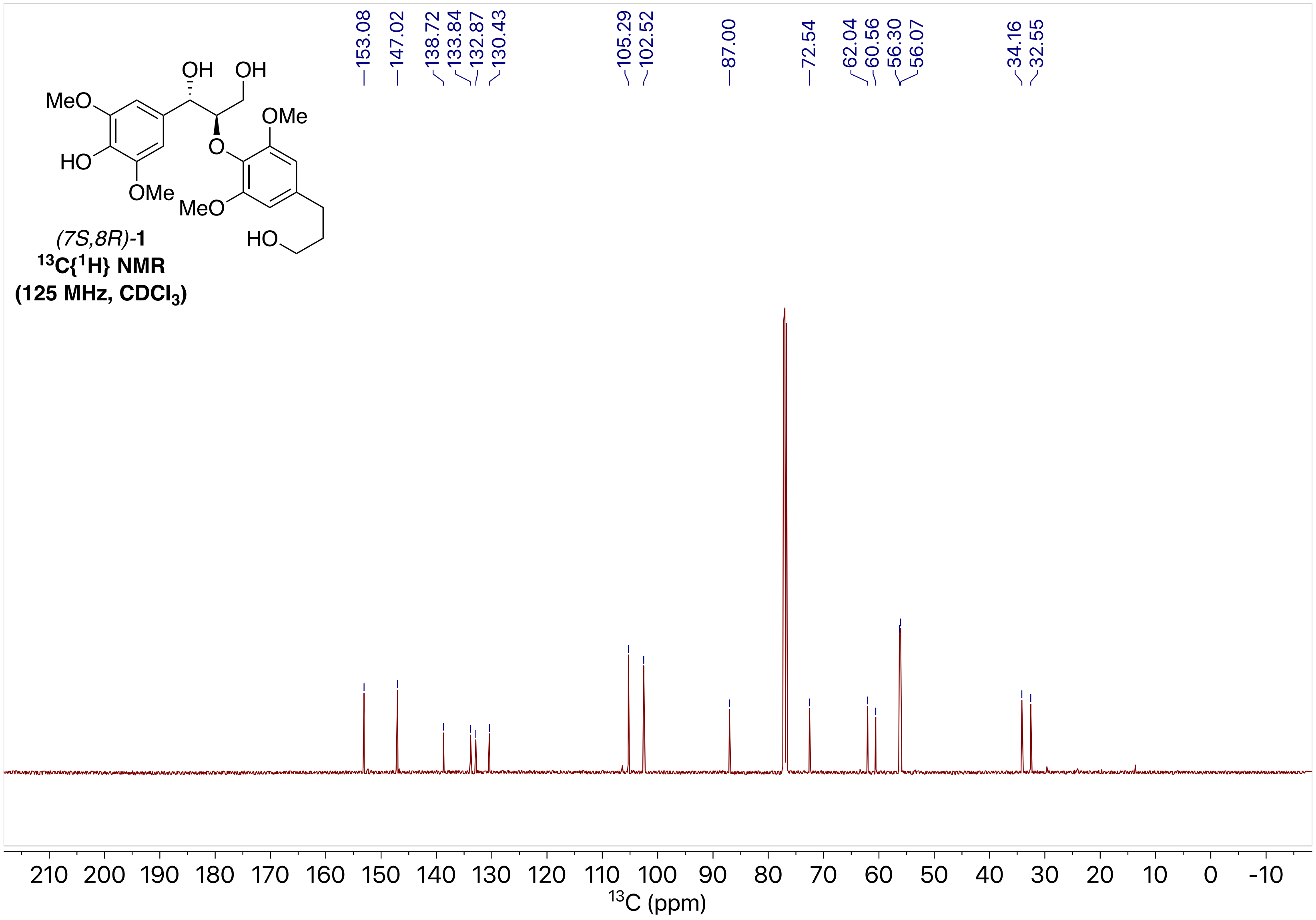
(7S,8R)-1
$^{13}C\{^{1}H\}$ NMR
(125 MHz, $CDCl_3$)
MeO
HO
OMe
OH
OH
O
OMe
MeO
HO
153.08
147.02
138.72
133.84
132.87
130.43
105.29
102.52
87.00
72.54
62.04
60.56
56.30
56.07
34.16
32.55
210 200 190 180 170 160 150 140 130 120 110 100 90 80 70 60 50 40 30 20 10 0 -10
^{13}C (ppm)

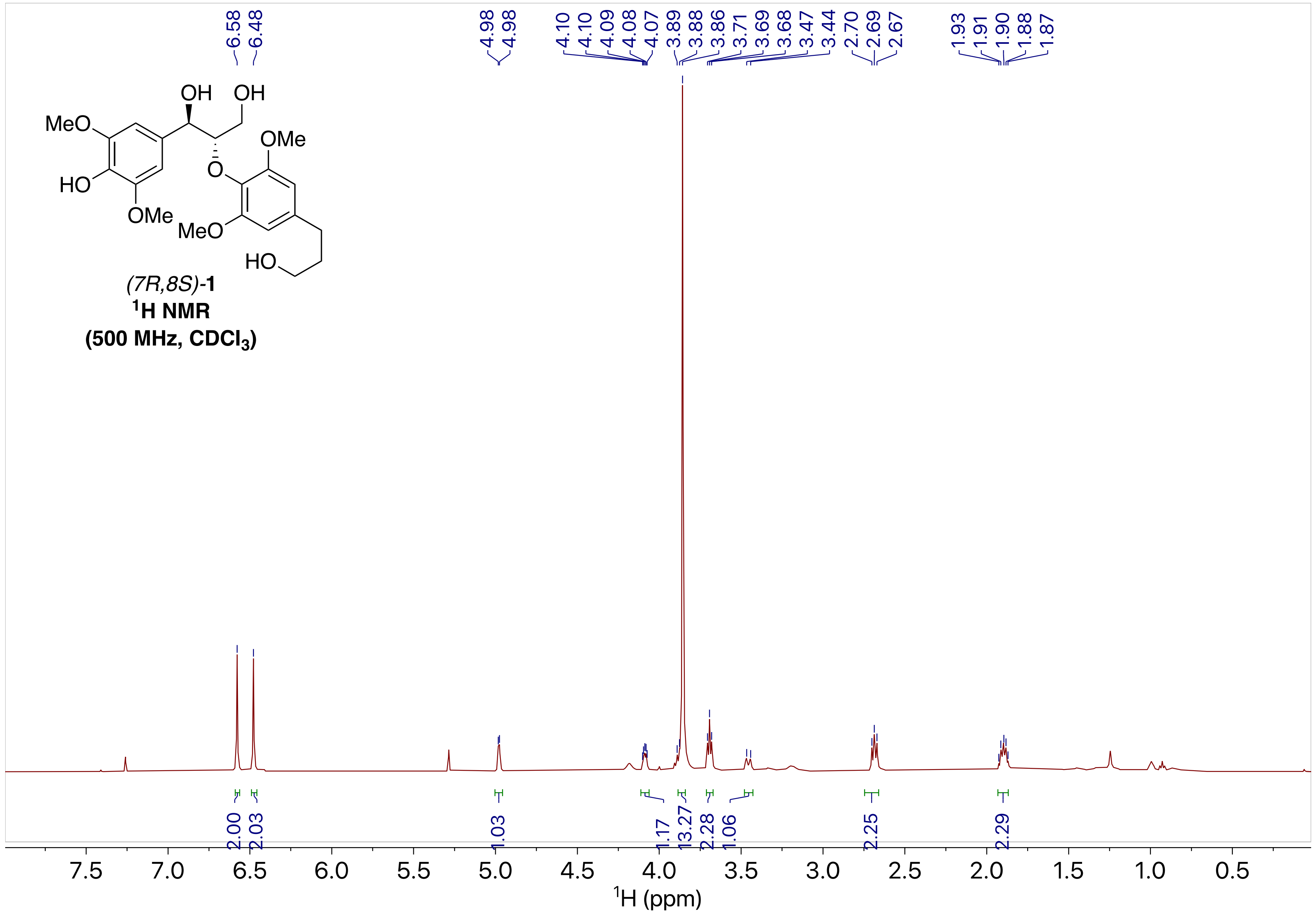

(7R,8S)-1
1H NMR
(500 MHz, CDCl3)
OH
OH
MeO
OMe
HO
O
OMe
MeO
HO
6.58
6.48
4.98
4.98
4.10
4.10
4.09
4.08
4.07
3.89
3.88
3.86
3.71
3.69
3.68
3.47
3.44
2.70
2.69
2.67
1.93
1.91
1.90
1.88
1.87
2.00
2.03
1.03
1.17
13.27
2.28
1.06
2.25
2.29
7.5
7.0
6.5
6.0
5.5
5.0
4.5
4.0
3.5
3.0
2.5
2.0
1.5
1.0
0.5
1H (ppm)

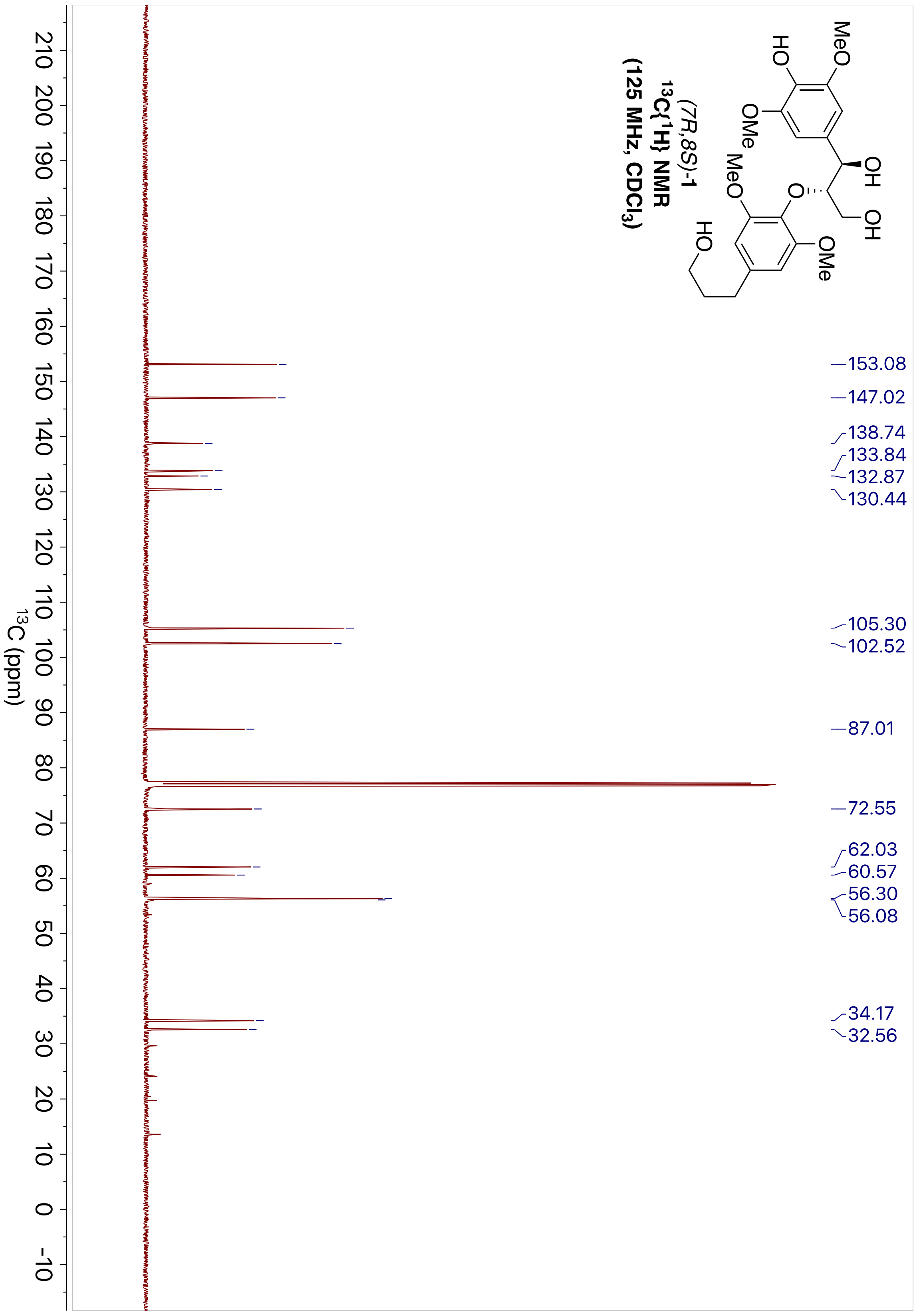
(7R,8S)-1
13C{1H} NMR
(125 MHz, CDCl3)
153.08
147.02
138.74
133.84
132.87
130.44
105.30
102.52
87.01
72.55
62.03
60.57
56.30
56.08
34.17
32.56
13C (ppm)

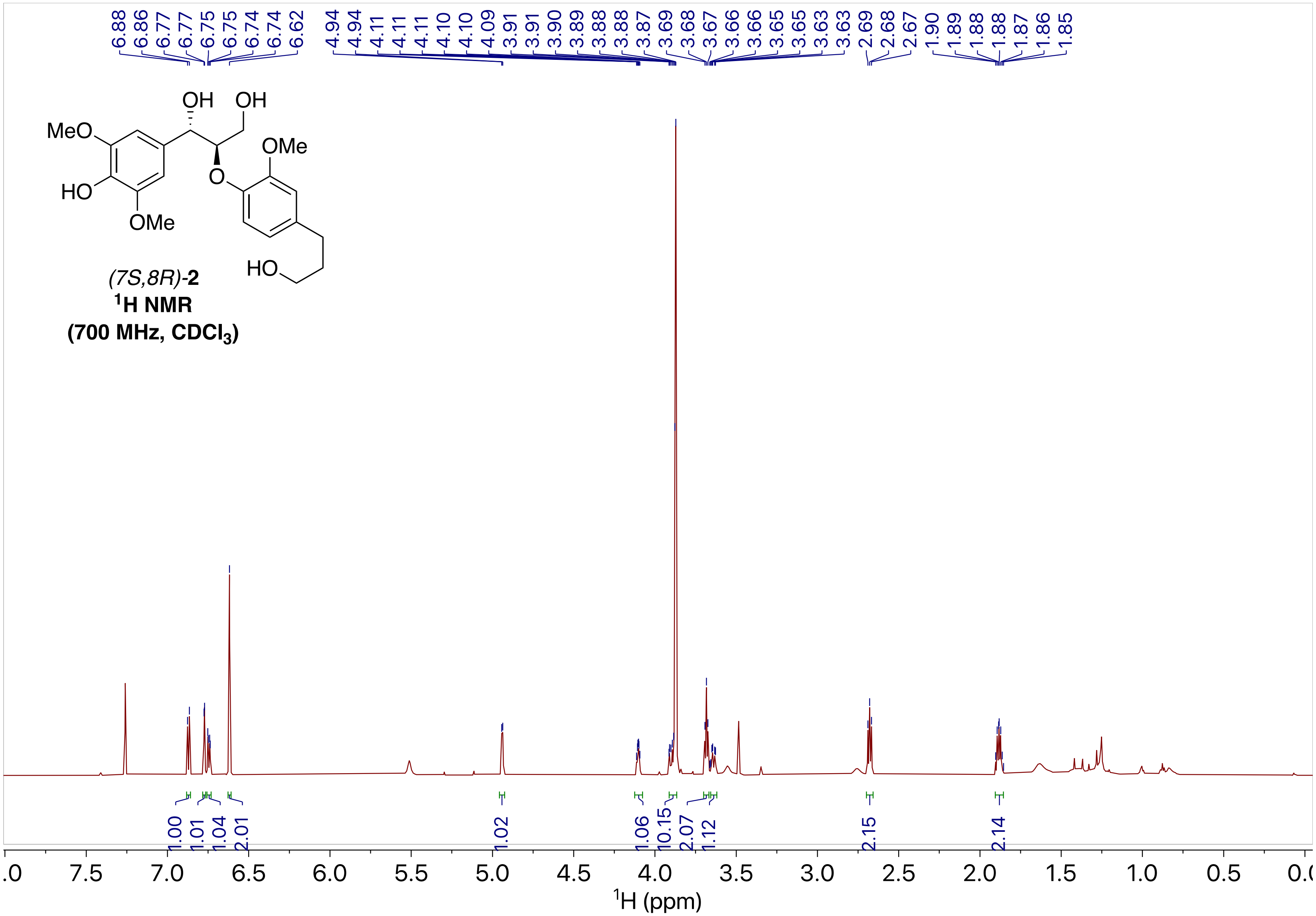
(7S,8R)-2
1H NMR
(700 MHz, CDCl3)
MeO
HO
OMe
OH
OH
O
OMe
HO
6.88 6.86 6.77 6.77 6.75 6.75 6.74 6.74 6.62
4.94 4.94 4.11 4.11 4.11 4.10 4.10 4.09 3.91 3.91 3.90 3.89 3.88 3.88 3.87 3.69 3.68 3.67 3.66 3.66 3.65 3.65 3.63 3.63
2.69 2.68 2.67
1.90 1.89 1.88 1.88 1.87 1.86 1.85
1.00 1.01 1.04 2.01 1.02 1.06 10.15 2.07 1.12 2.15 2.14
7.5 7.0 6.5 6.0 5.5 5.0 4.5 4.0 3.5 3.0 2.5 2.0 1.5 1.0 0.5
1H (ppm)

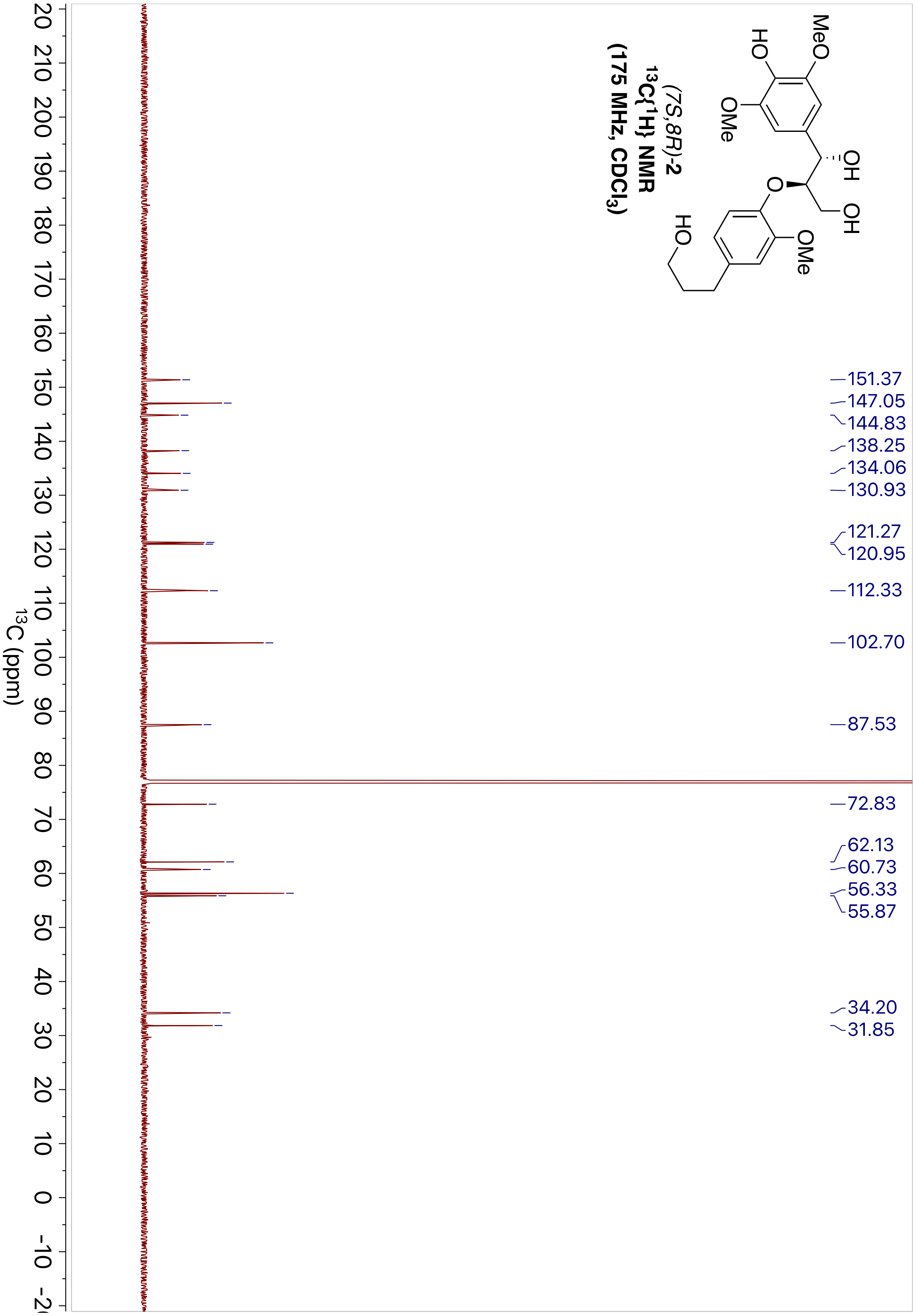

(7S,8R)-2
13C{1H} NMR
(175 MHz, CDCl3)
MeO
HO
OMe
OH
OH
O
OMe
HO
151.37
147.05
144.83
138.25
134.06
130.93
121.27
120.95
112.33
102.70
87.53
72.83
62.13
60.73
56.33
55.87
34.20
31.85
220 210 200 190 180 170 160 150 140 130 120 110 100 90 80 70 60 50 40 30 20 10 0 -10 -20
13C (ppm)

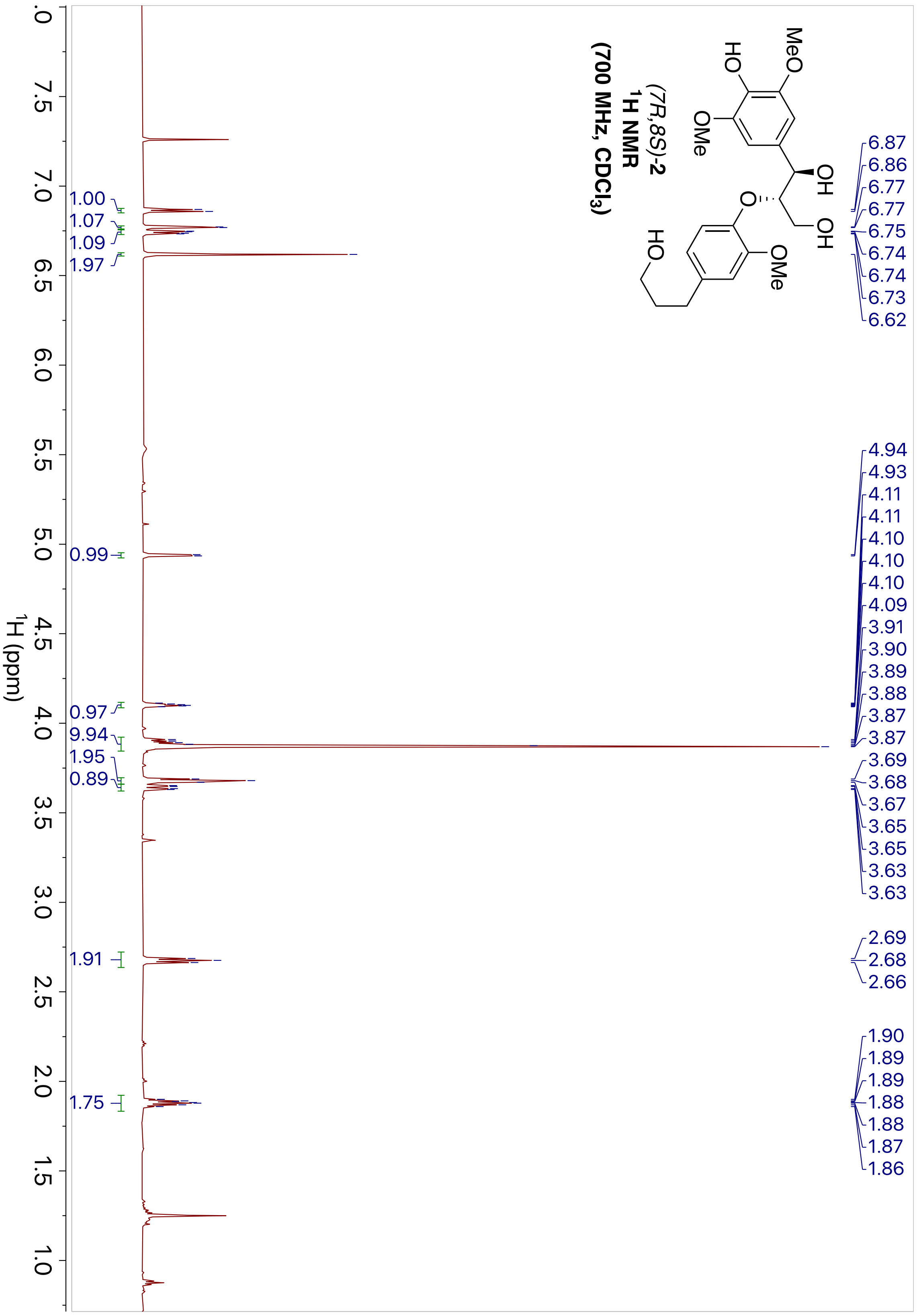

(7R,8S)-2
1H NMR
(700 MHz, CDCl3)
MeO
HO
OMe
OH
OH
O
OMe
HO
1H (ppm)
6.87
6.86
6.77
6.77
6.75
6.74
6.74
6.73
6.62
4.94
4.93
4.11
4.11
4.10
4.10
4.10
4.09
3.91
3.90
3.89
3.88
3.87
3.87
3.69
3.68
3.67
3.65
3.65
3.63
3.63
2.69
2.68
2.66
1.90
1.89
1.89
1.88
1.88
1.87
1.86
1.00
1.07
1.09
1.97
0.99
0.97
9.94
1.95
0.89
1.91
1.75

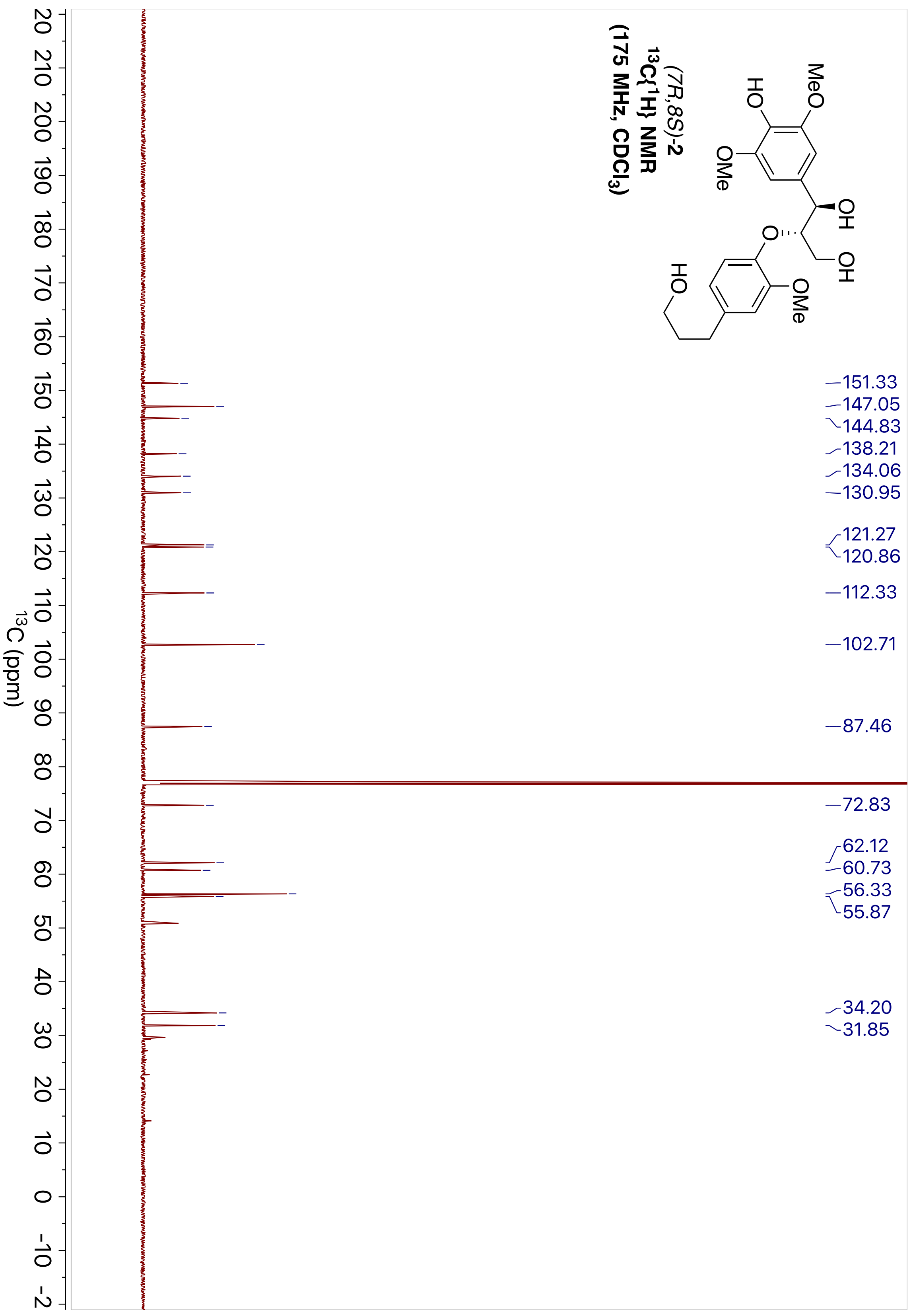
(7R,8S)-2
$^{13}C\{^{1}H\}$ NMR
(175 MHz, $CDCl_3$)
MeO
HO
OMe
OH
OH
O
OMe
HO
151.33
147.05
144.83
138.21
134.06
130.95
121.27
120.86
112.33
102.71
87.46
72.83
62.12
60.73
56.33
55.87
34.20
31.85
^{13}C (ppm)

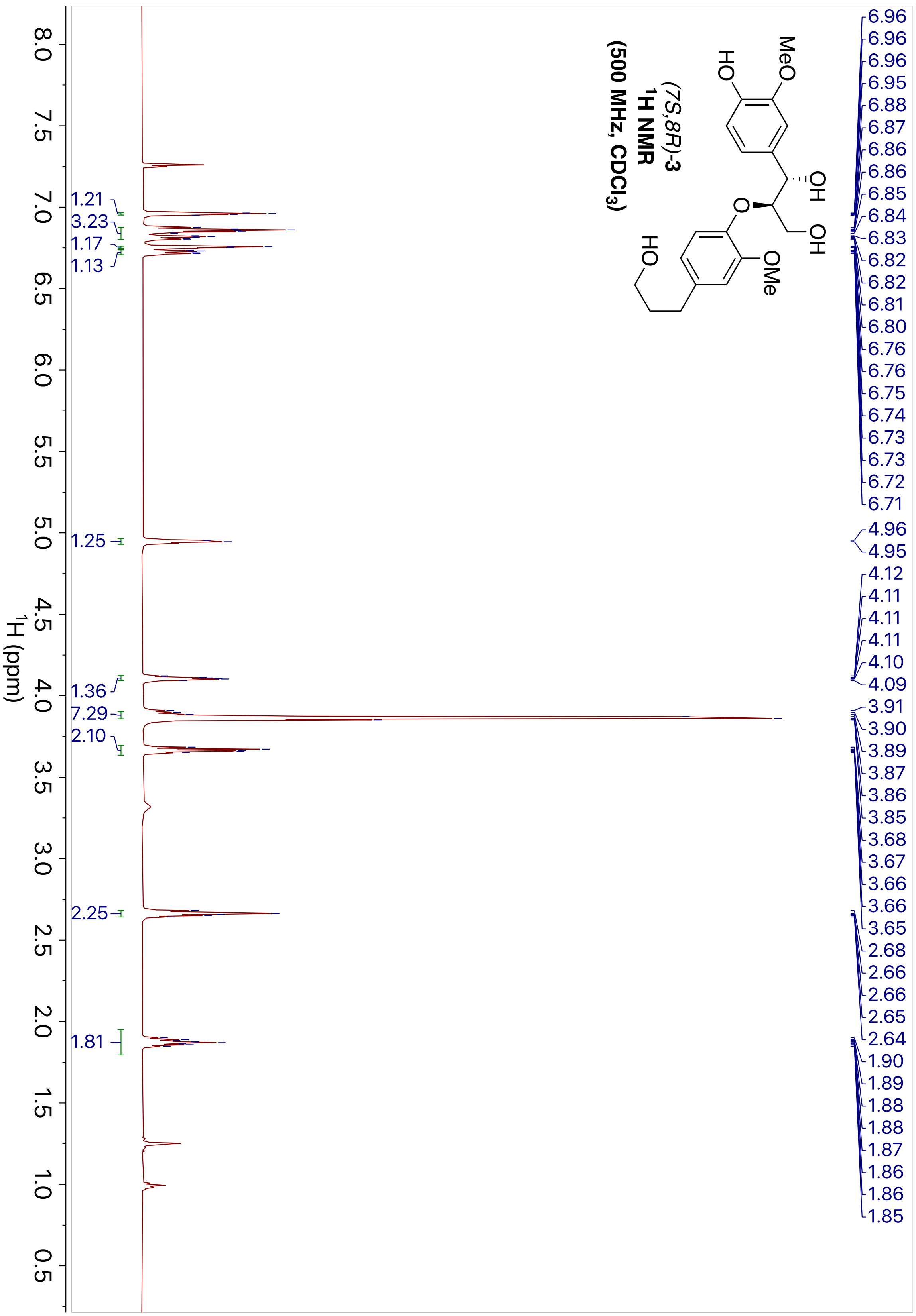
(7S,8R)-3
^{1}H NMR
(500 MHz, CDCl$_3$)
MeO
HO
OH
OH
O
OMe
HO
6.96
6.96
6.96
6.95
6.88
6.87
6.86
6.86
6.85
6.84
6.83
6.82
6.82
6.81
6.80
6.76
6.76
6.75
6.74
6.73
6.73
6.72
6.71
4.96
4.95
4.12
4.11
4.11
4.11
4.10
4.09
3.91
3.90
3.89
3.87
3.86
3.85
3.68
3.67
3.66
3.66
3.65
2.68
2.66
2.66
2.65
2.64
1.90
1.89
1.88
1.88
1.87
1.86
1.86
1.85
1.21
3.23
1.17
1.13
1.25
1.36
7.29
2.10
2.25
1.81
^{1}H (ppm)
8.0
7.5
7.0
6.5
6.0
5.5
5.0
4.5
4.0
3.5
3.0
2.5
2.0
1.5
1.0
0.5

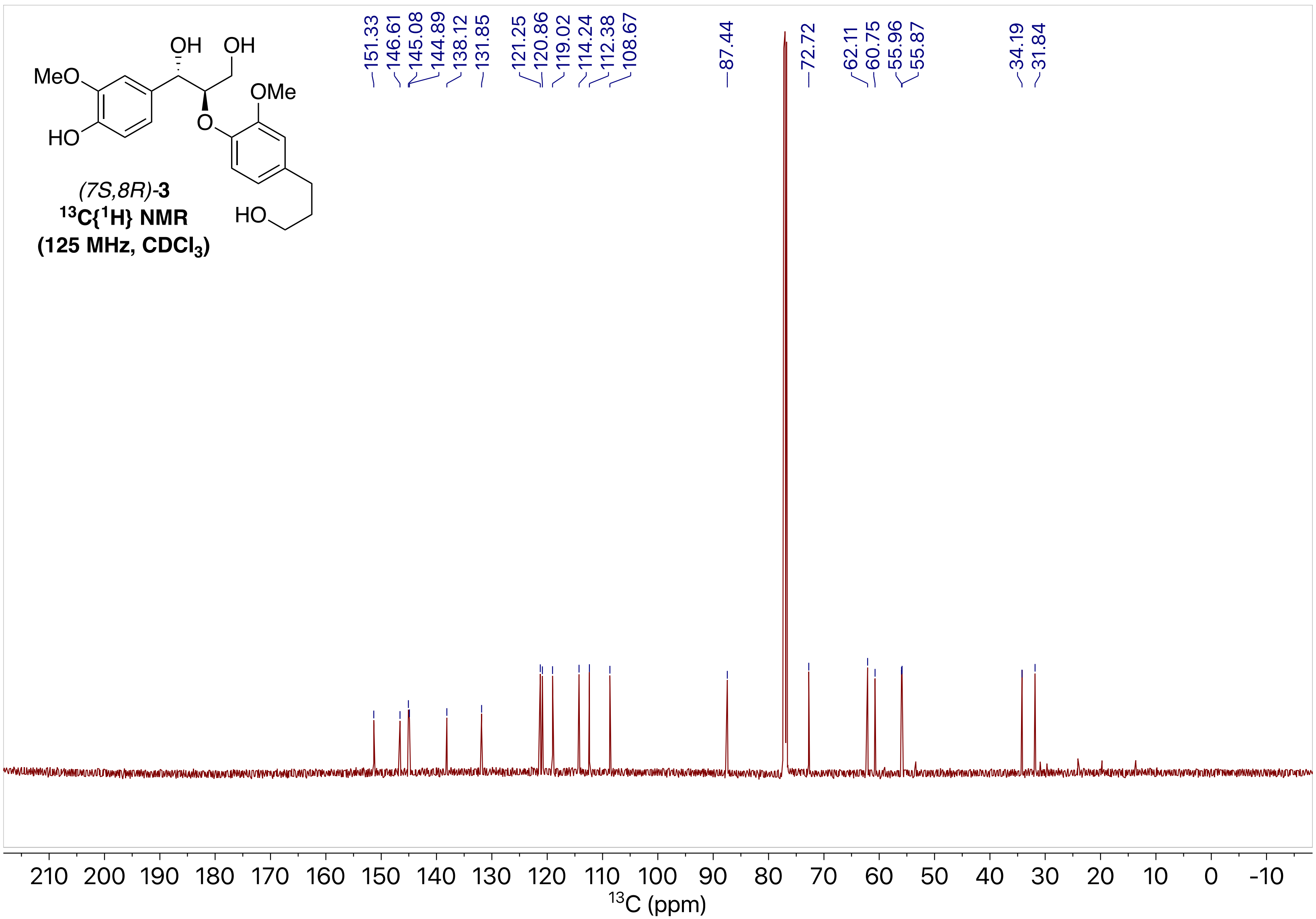
(7S,8R)-3
$^{13}C\{^{1}H\}$ NMR
(125 MHz, $CDCl_3$)
MeO
HO
OH
OH
O
OMe
HO
151.33
146.61
145.08
144.89
138.12
131.85
121.25
120.86
119.02
114.24
112.38
108.67
87.44
72.72
62.11
60.75
55.96
55.87
34.19
31.84
210 200 190 180 170 160 150 140 130 120 110 100 90 80 70 60 50 40 30 20 10 0 -10
^{13}C (ppm)

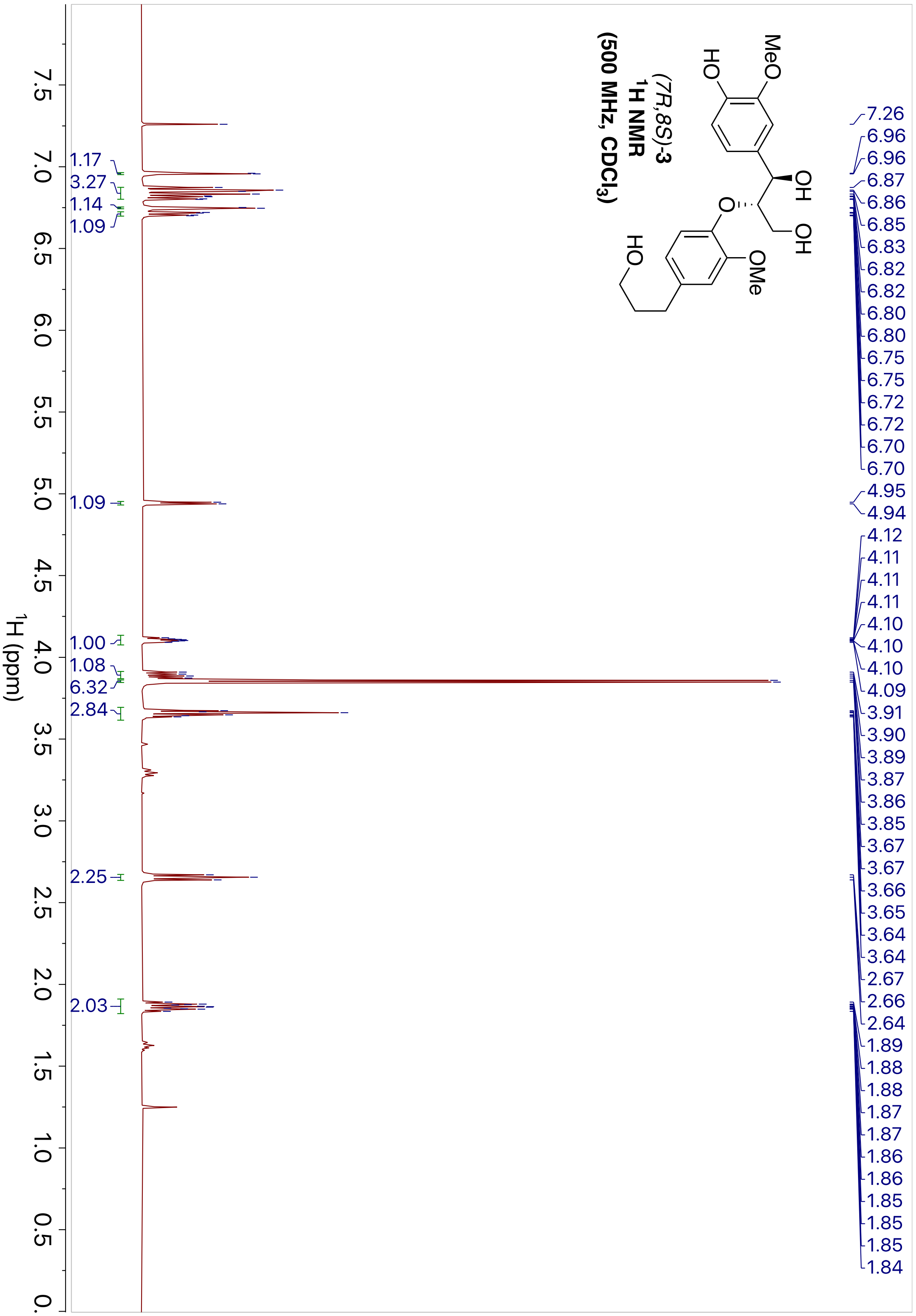
(7R,8S)-3
1H NMR
(500 MHz, CDCl3)
MeO
HO
OH
OH
O
OMe
HO
7.26
6.96
6.96
6.87
6.86
6.85
6.83
6.82
6.82
6.80
6.80
6.75
6.75
6.72
6.72
6.70
6.70
4.95
4.94
4.12
4.11
4.11
4.11
4.10
4.10
4.10
4.09
3.91
3.90
3.89
3.87
3.86
3.85
3.67
3.67
3.66
3.65
3.64
3.64
2.67
2.66
2.64
1.89
1.88
1.88
1.87
1.87
1.86
1.86
1.85
1.85
1.85
1.84
1.17
3.27
1.14
1.09
1.09
1.00
1.08
6.32
2.84
2.25
2.03
1H (ppm)

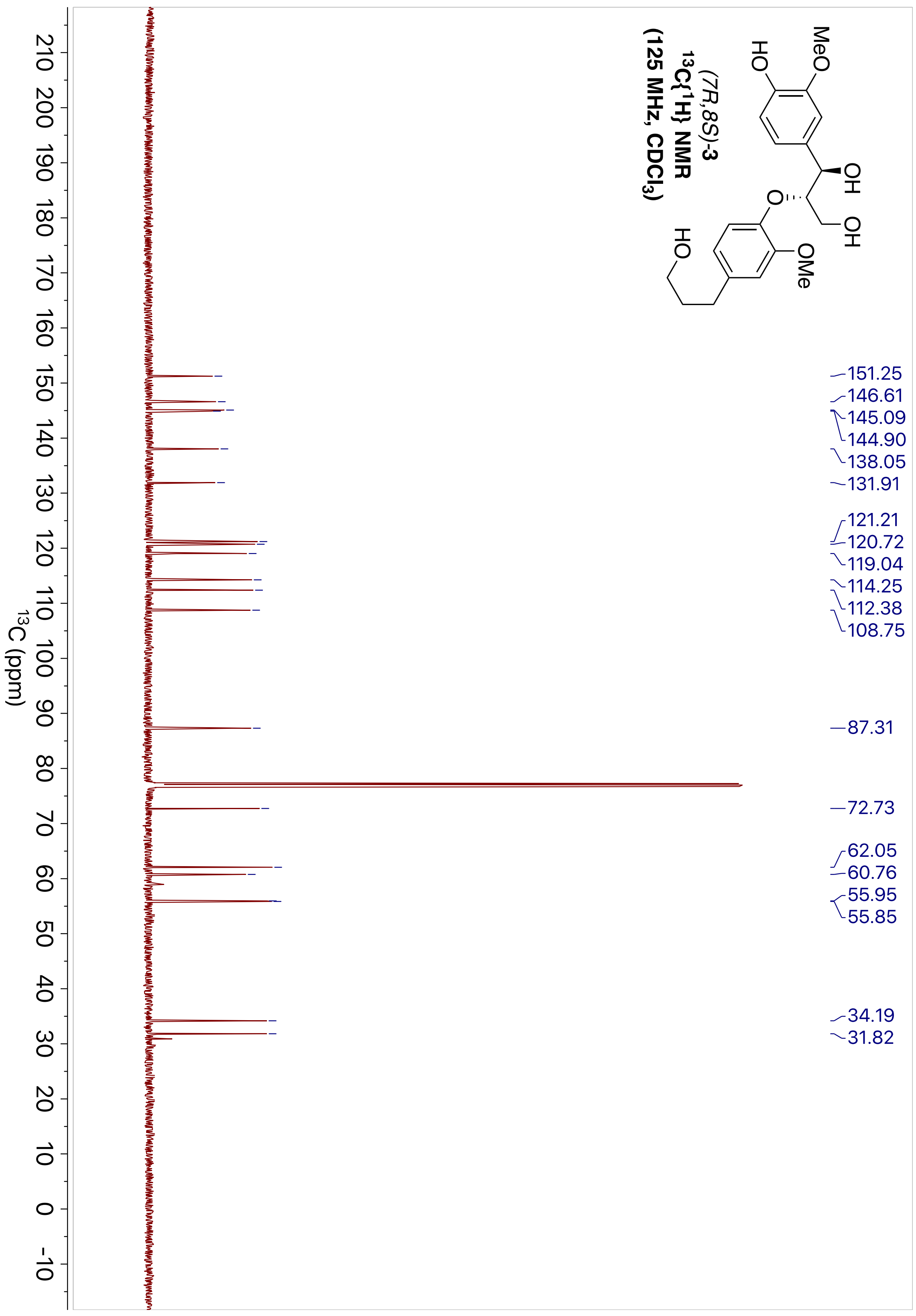
(7R,8S)-3
13C{1H} NMR
(125 MHz, CDCl3)
MeO
HO
OH
OH
O
OMe
HO
151.25
146.61
145.09
144.90
138.05
131.91
121.21
120.72
119.04
114.25
112.38
108.75
87.31
72.73
62.05
60.76
55.95
55.85
34.19
31.82
210 200 190 180 170 160 150 140 130 120 110 100 90 80 70 60 50 40 30 20 10 0 -10
13C (ppm)

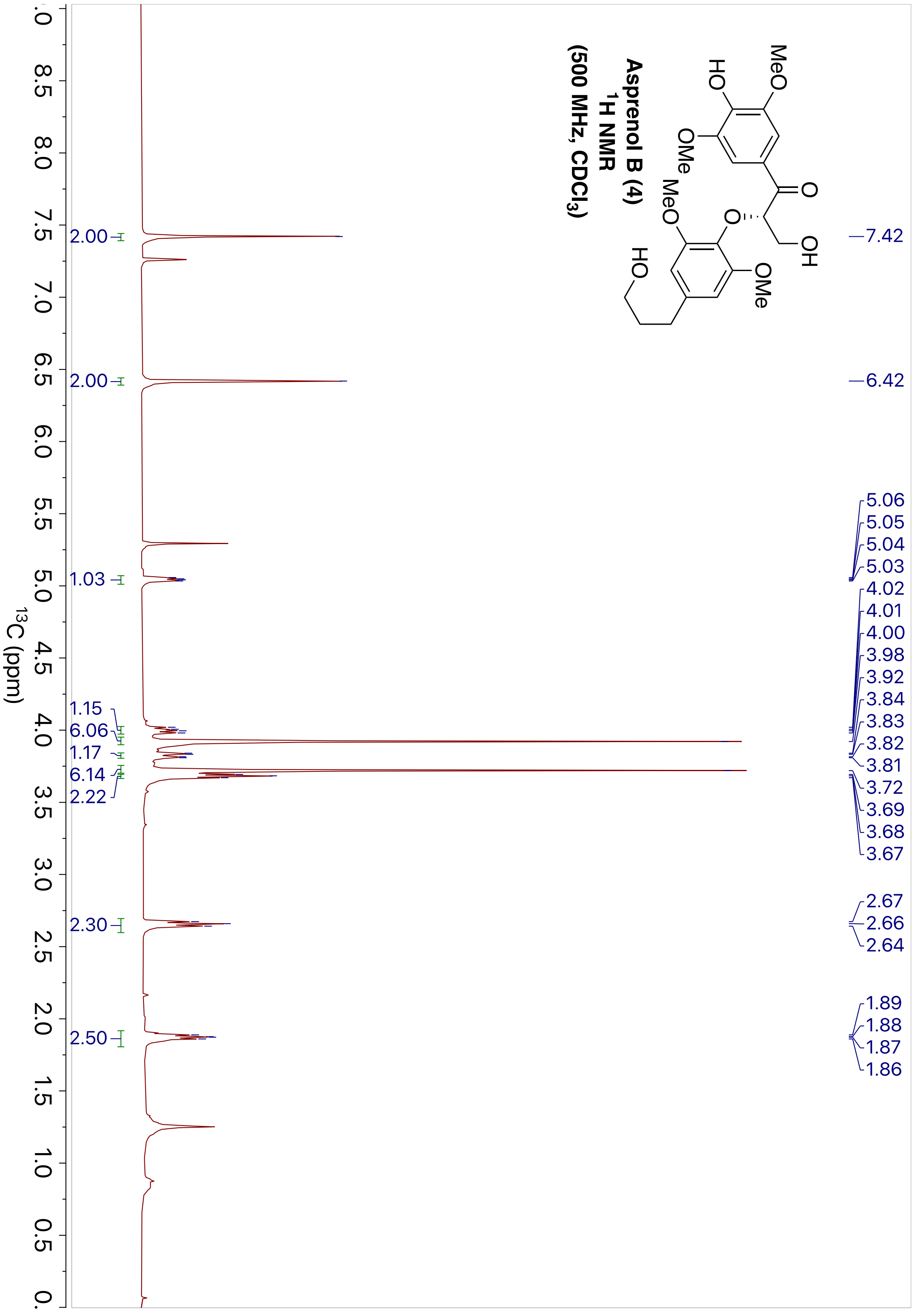
Asprenol B (4)
^{1}H NMR
(500 MHz, CDCl$_3$)
MeO
HO
OMe
O
OH
O
OMe
MeO
HO
7.42
6.42
5.06
5.05
5.04
5.03
4.02
4.01
4.00
3.98
3.92
3.84
3.83
3.82
3.81
3.72
3.69
3.68
3.67
2.67
2.66
2.64
1.89
1.88
1.87
1.86
2.00
2.00
1.03
1.15
6.06
1.17
6.14
2.22
2.30
2.50
.0
8.5
8.0
7.5
7.0
6.5
6.0
5.5
5.0
4.5
4.0
3.5
3.0
2.5
2.0
1.5
1.0
0.5
0.
^{13}C (ppm)

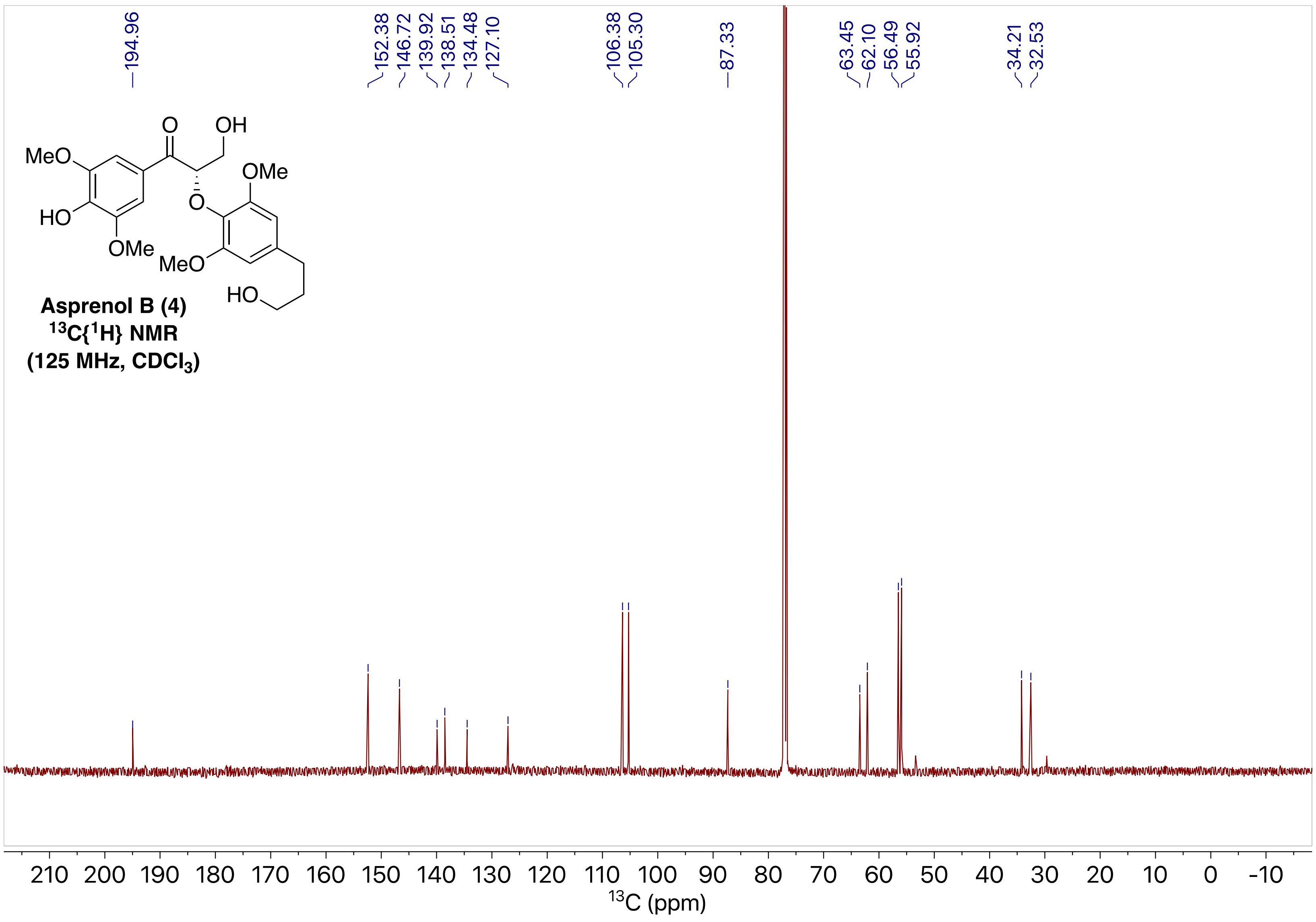

194.96
152.38
146.72
139.92
138.51
134.48
127.10
106.38
105.30
87.33
63.45
62.10
56.49
55.92
34.21
32.53
MeO
HO
OMe
O
OH
OMe
MeO
HO
Asprenol B (4)
$^{13}C\{^1H\}$ NMR
(125 MHz, $CDCl_3$)
210 200 190 180 170 160 150 140 130 120 110 100 90 80 70 60 50 40 30 20 10 0 -10
^{13}C (ppm)

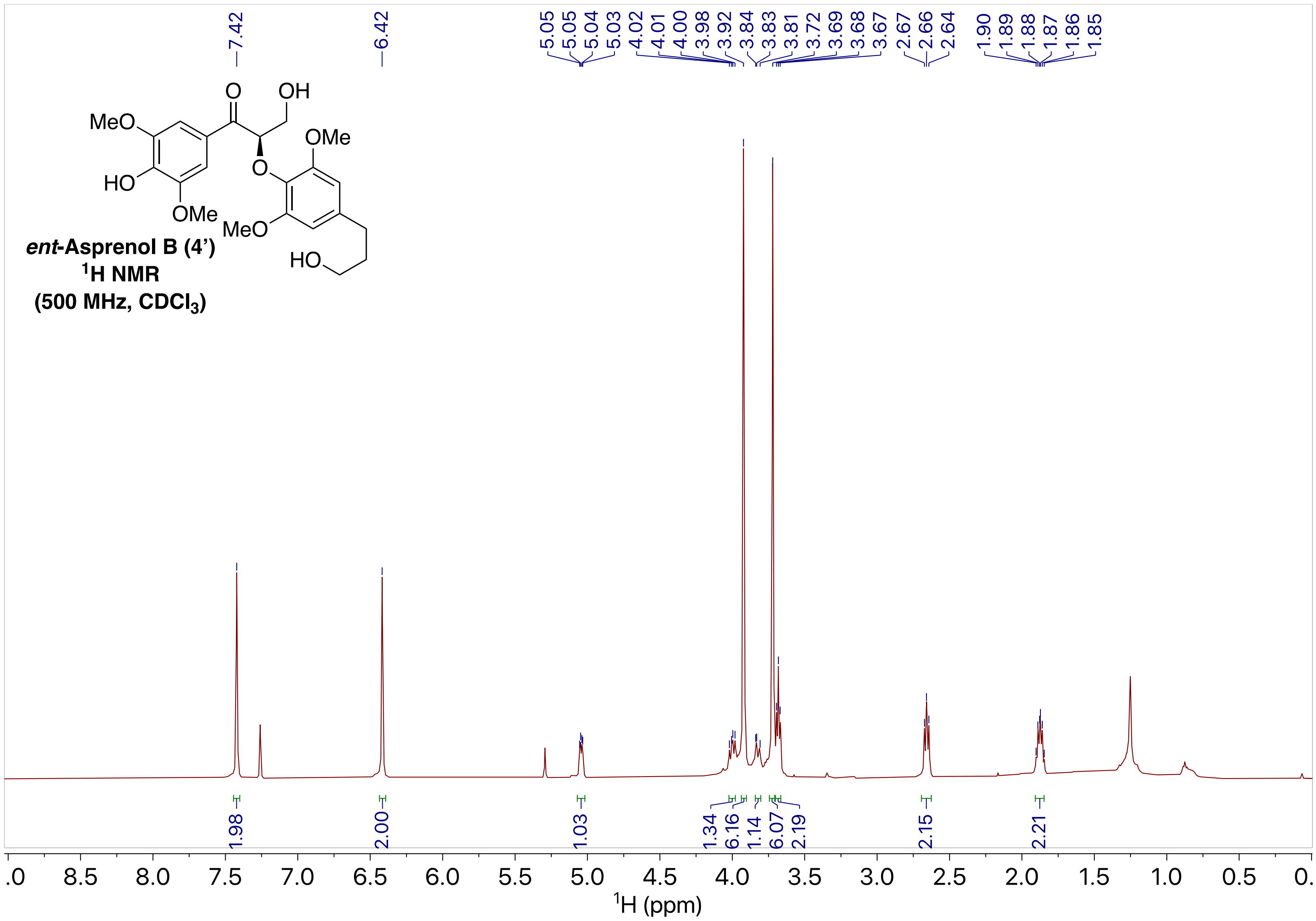
ent-Asprenol B (4')
1H NMR
(500 MHz, CDCl3)
MeO
HO
OMe
O
OH
OMe
MeO
HO
7.42
6.42
5.05
5.05
5.04
5.03
4.02
4.01
4.00
3.98
3.92
3.84
3.83
3.81
3.72
3.69
3.68
3.67
2.67
2.66
2.64
1.90
1.89
1.88
1.87
1.86
1.85
1.98
2.00
1.03
1.34
6.16
1.14
6.07
2.19
2.15
2.21
.0
8.5
8.0
7.5
7.0
6.5
6.0
5.5
5.0
4.5
4.0
3.5
3.0
2.5
2.0
1.5
1.0
0.5
0.
1H (ppm)

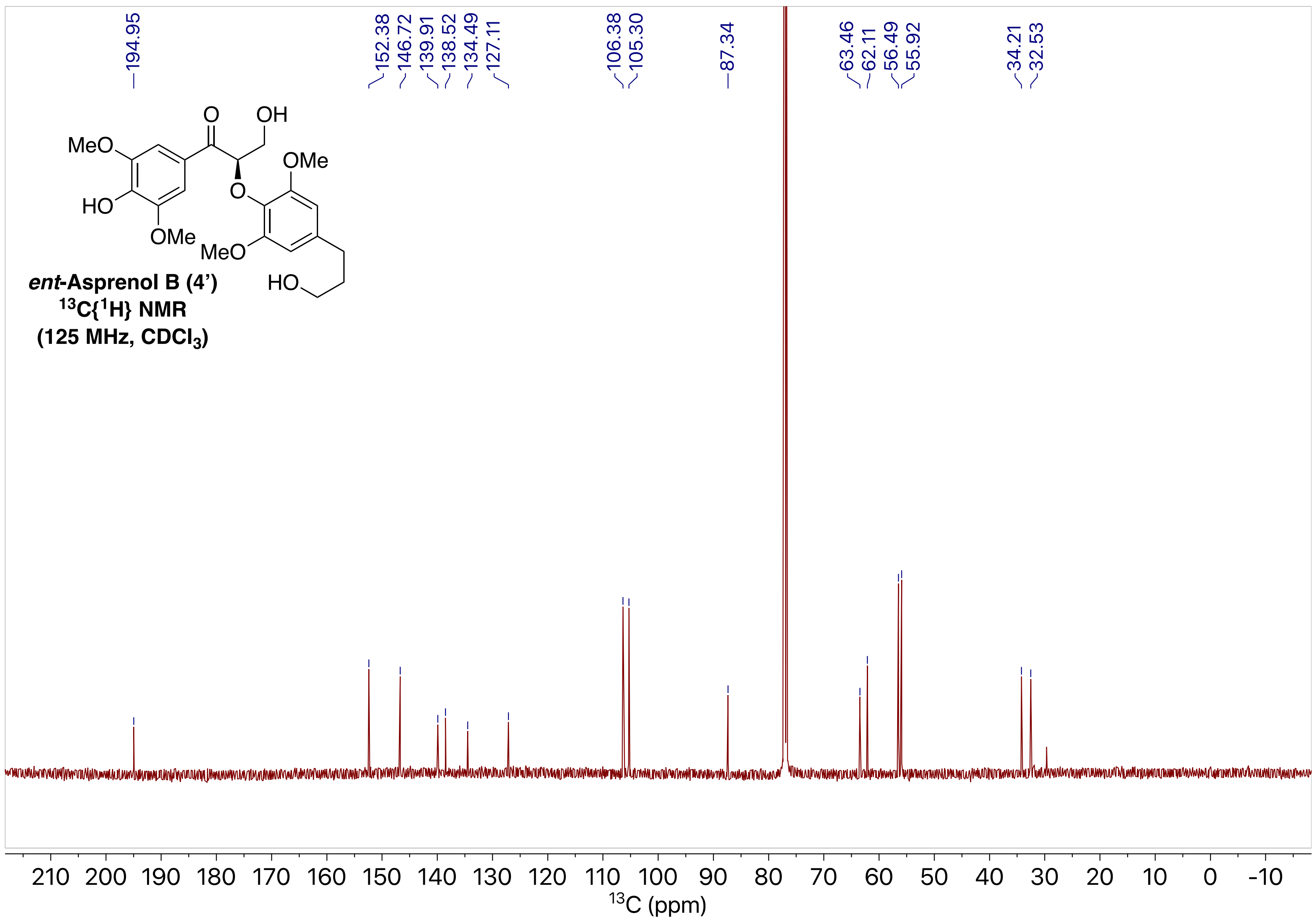
ent-Asprenol B (4')
$^{13}C\{^{1}H\}$ NMR
(125 MHz, $CDCl_3$)
MeO
HO
OMe
O
OH
OMe
MeO
HO
194.95
152.38
146.72
139.91
138.52
134.49
127.11
106.38
105.30
87.34
63.46
62.11
56.49
55.92
34.21
32.53
210 200 190 180 170 160 150 140 130 120 110 100 90 80 70 60 50 40 30 20 10 0 -10
^{13}C (ppm)

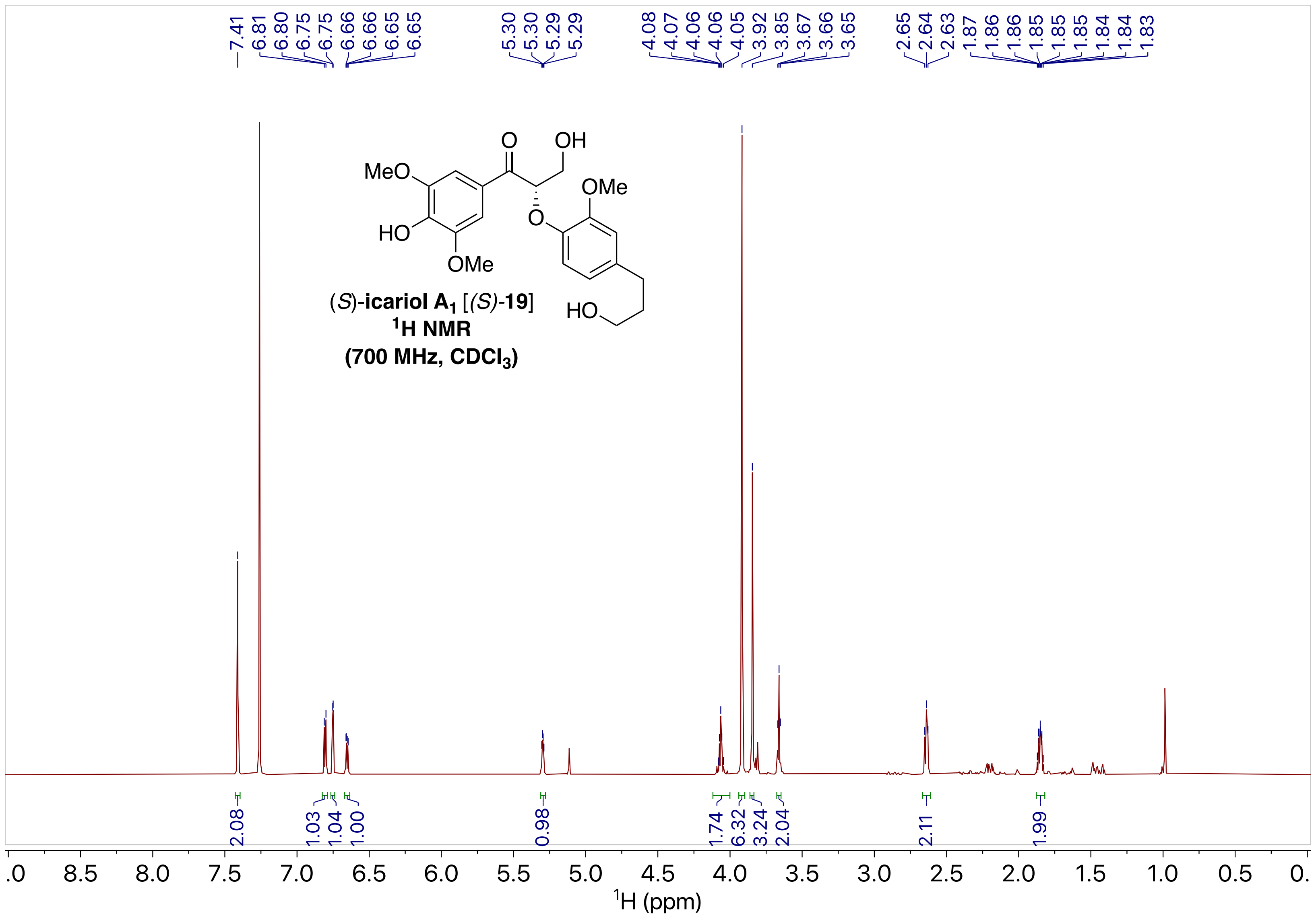
(S)-icariol A$_1$ [(S)-19]
^{1}H NMR
(700 MHz, $CDCl_3$)
MeO
HO
OMe
O
OH
OMe
O
HO
7.41
6.81
6.80
6.75
6.75
6.66
6.66
6.65
6.65
5.30
5.30
5.29
5.29
4.08
4.07
4.06
4.06
4.05
3.92
3.85
3.67
3.66
3.65
2.65
2.64
2.63
1.87
1.86
1.86
1.85
1.85
1.85
1.84
1.84
1.83
2.08
1.03
1.04
1.00
0.98
1.74
6.32
3.24
2.04
2.11
1.99
^{1}H (ppm)

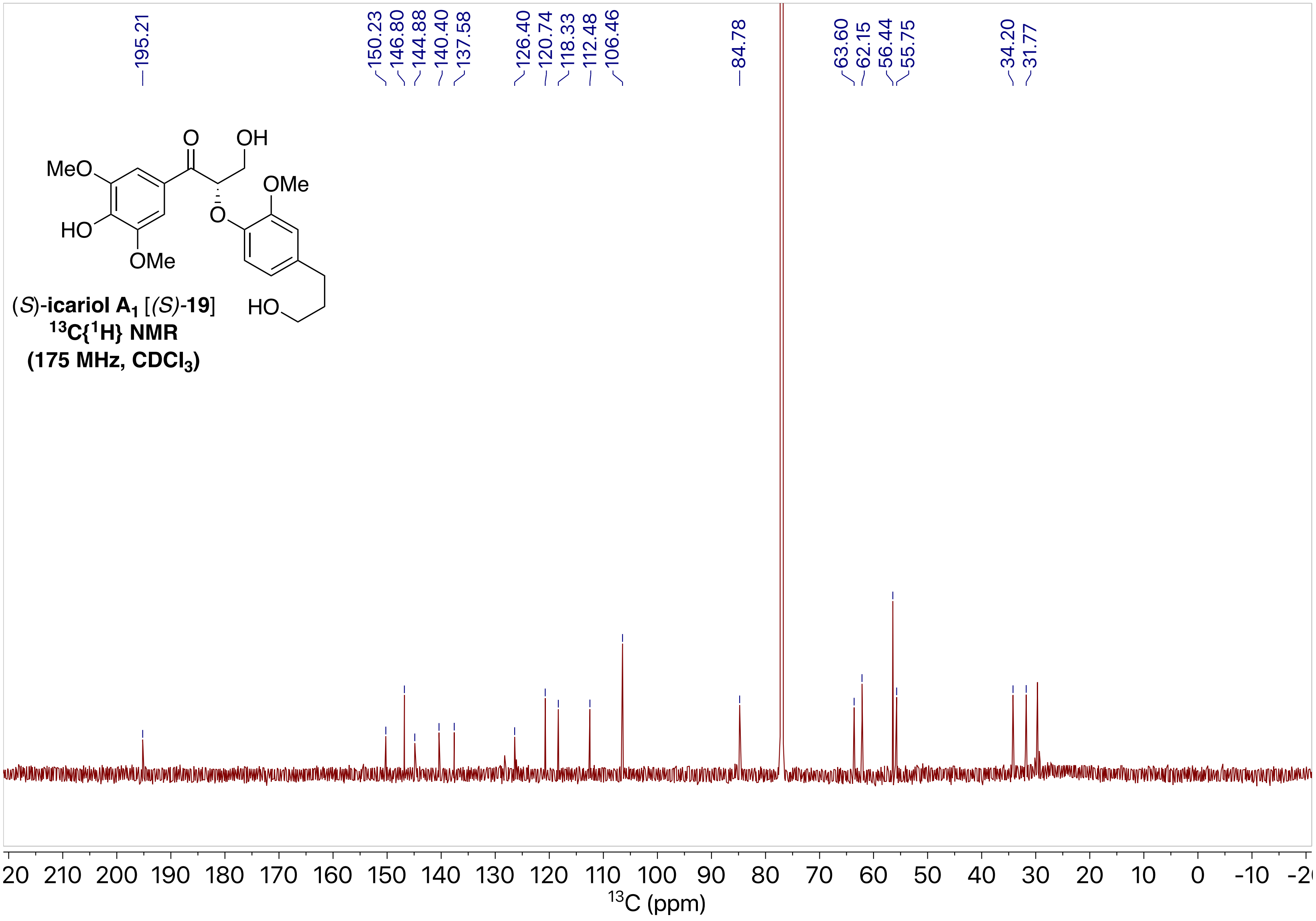
195.21
150.23
146.80
144.88
140.40
137.58
126.40
120.74
118.33
112.48
106.46
84.78
63.60
62.15
56.44
55.75
34.20
31.77
MeO
HO
OMe
O
OH
OMe
O
HO
(*S*)-**icariol A$_1$** [(*S*)-**19**]
$^{13}C\{^1H\}$ NMR
(175 MHz, $CDCl_3$)
^{13}C (ppm)

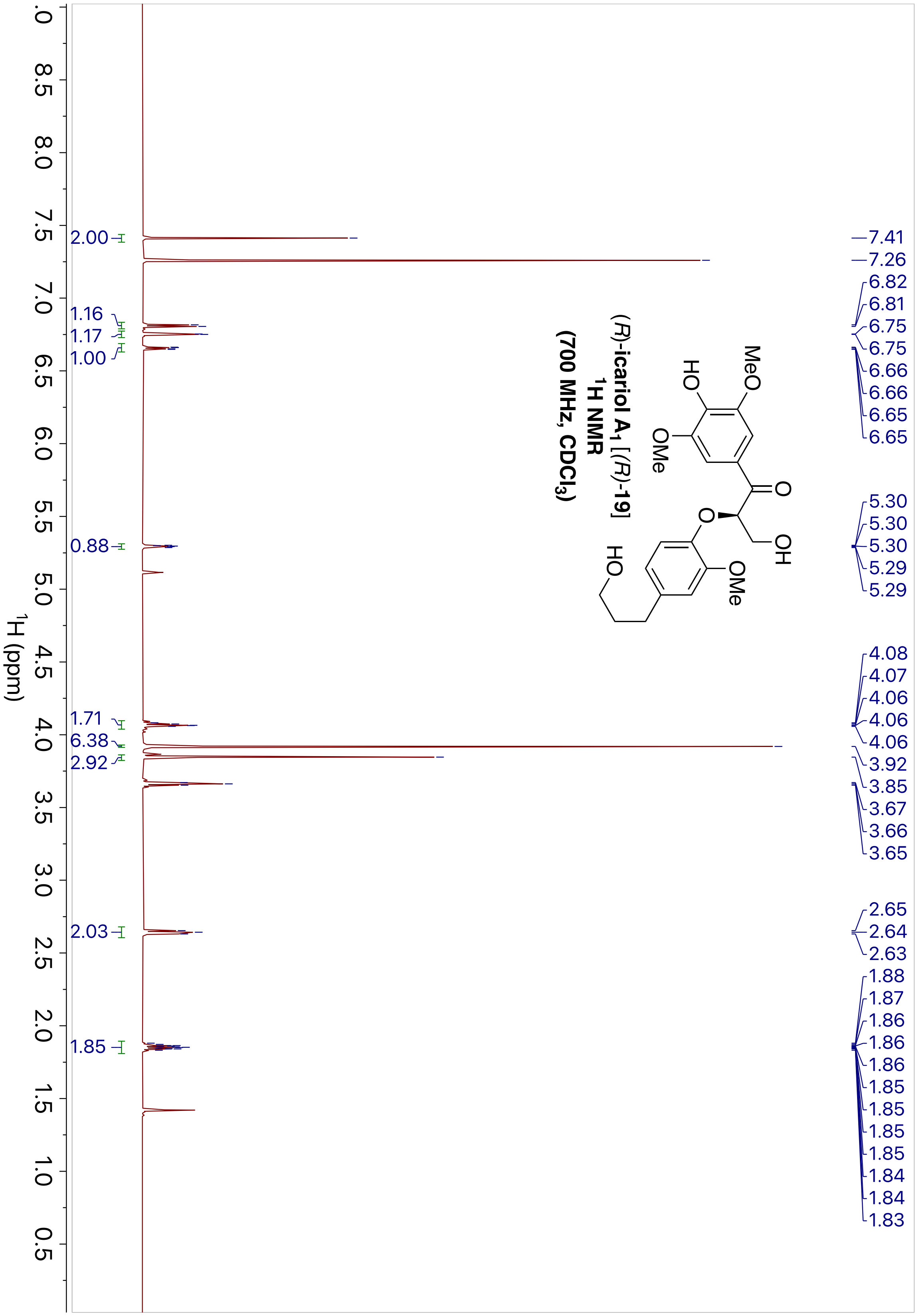
(R)-icariol A1 [(R)-19]
1H NMR
(700 MHz, CDCl3)
1H (ppm)

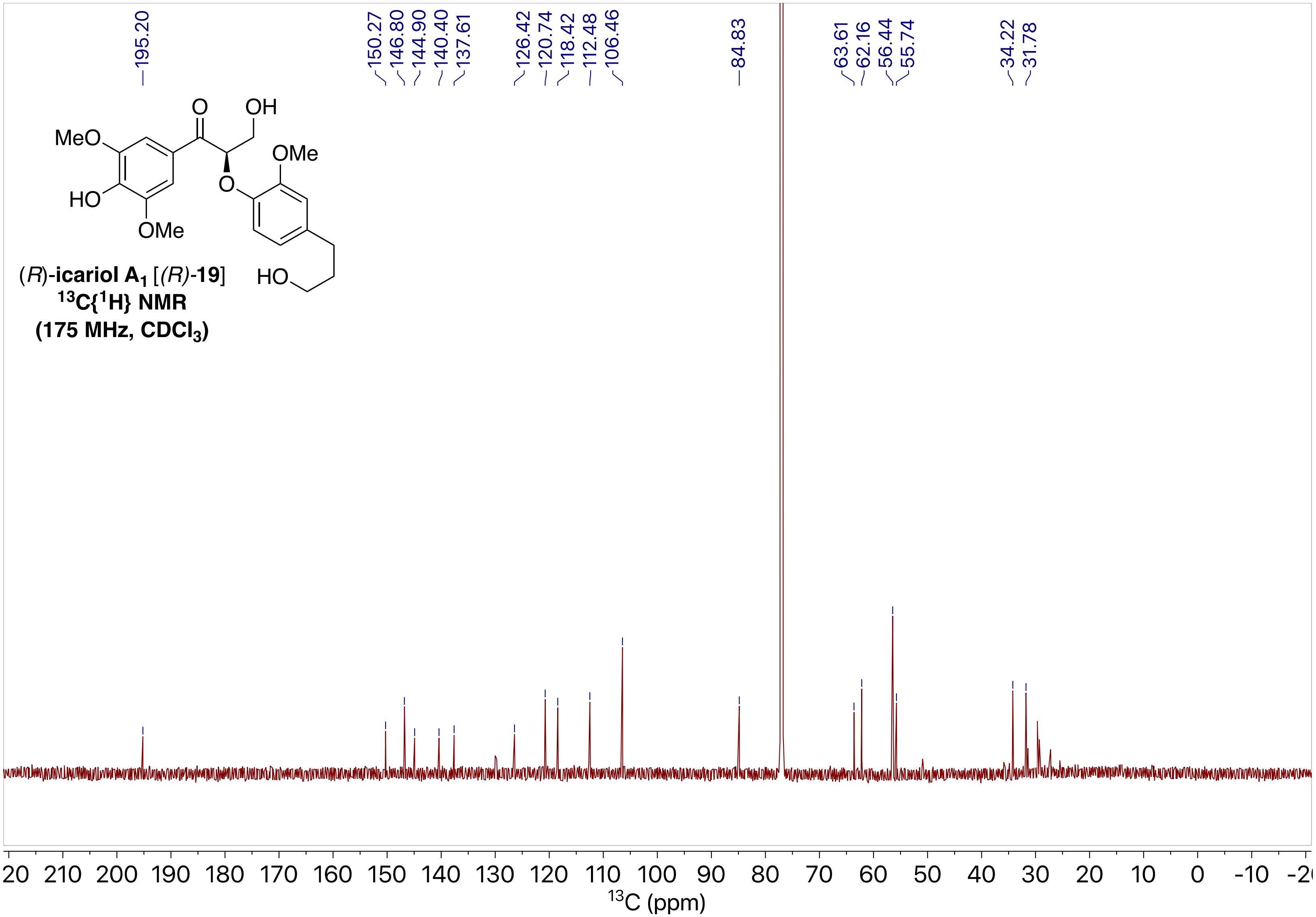

(*R*)-**icariol A$_1$** [(*R*)-**19**]
^{13}C{^{1}H} NMR
(175 MHz, $CDCl_3$)

4. HPLC data

A. Compounds **9a** and **9b**

Column Info: RegisReflect C-Amylose A, 5μm, 250mm*4.6mm
Mobile Phase: 5% IPA/Hexanes
Flow Rate: 1.5 ml/min

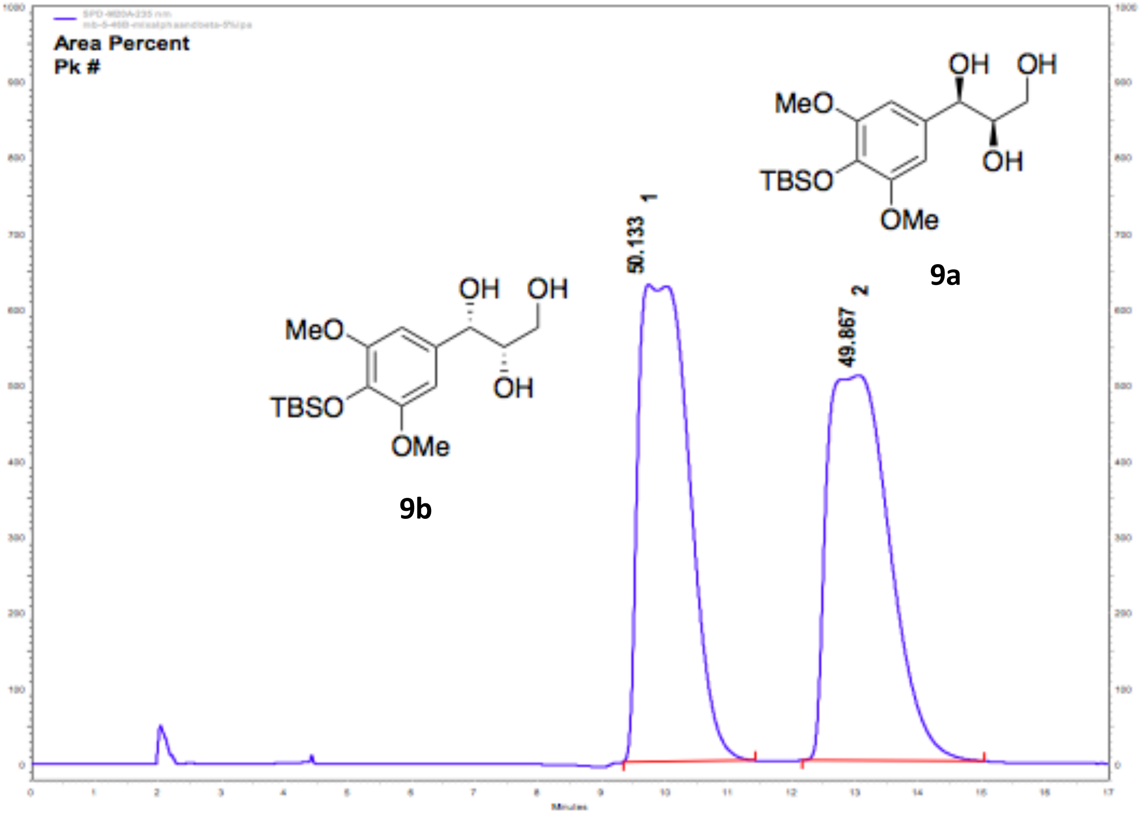

Retention Time	Area	Area %	Height	Height %
9.752	34913034	50.13	629092	55.33
13.068	34727396	49.87	507948	44.67
Totals				
	69640430	100.00	1137040	100.00

Column Info: RegisReflect C-Amylose A, 5μm, 250mm*4.6mm
Mobile Phase: 5% IPA/Hexanes
Flow Rate: 1.5 ml/min

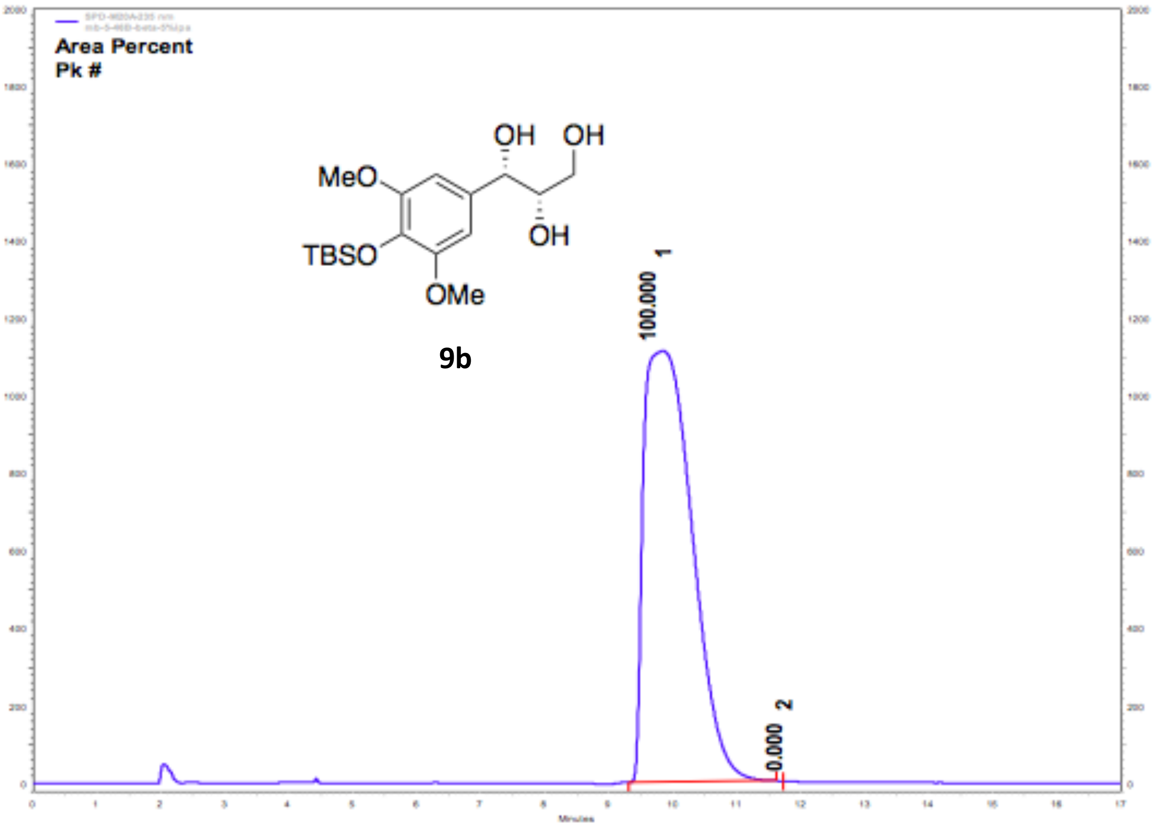

Retention Time	Area	Area %	Height	Height %
9.856	58520920	100.00	1112423	100.00
11.728	0	0.00	0	0.00
Totals				
	58520920	100.00	1112423	100.00

Column Info: RegisReflect C-Amylose A, 5µm, 250mm*4.6mm
Mobile Phase: 5% IPA/Hexanes
Flow Rate: 1.5 ml/min

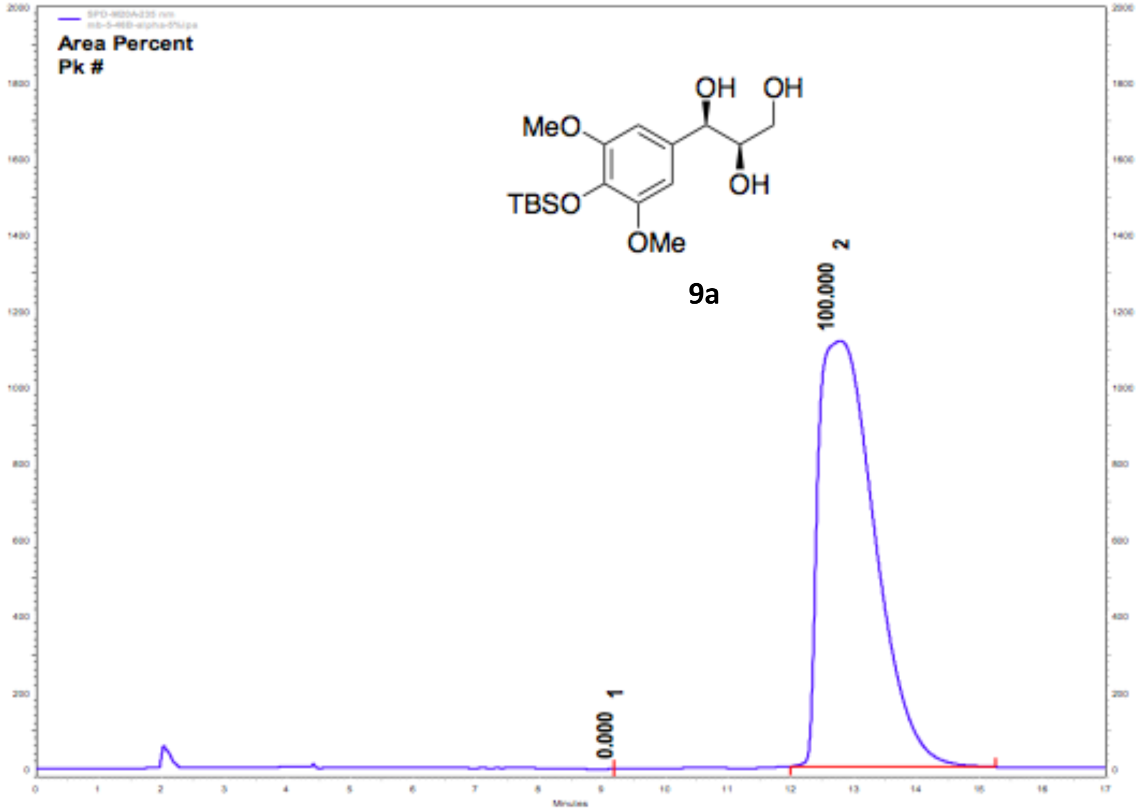

Retention Time	Area	Area %	Height	Height %
9.188	0	0.00	0	0.00
12.800	69320259	100.00	1116720	100.00
Totals				
	69320259	100.00	1116720	100.00

B. Compounds **9c** and **9d**

Column Info: RegisReflect C-Amylose A, 5µm, 250mm*4.6mm
Mobile Phase: 5% IPA/Hexanes
Flow Rate: 1.5 ml/min

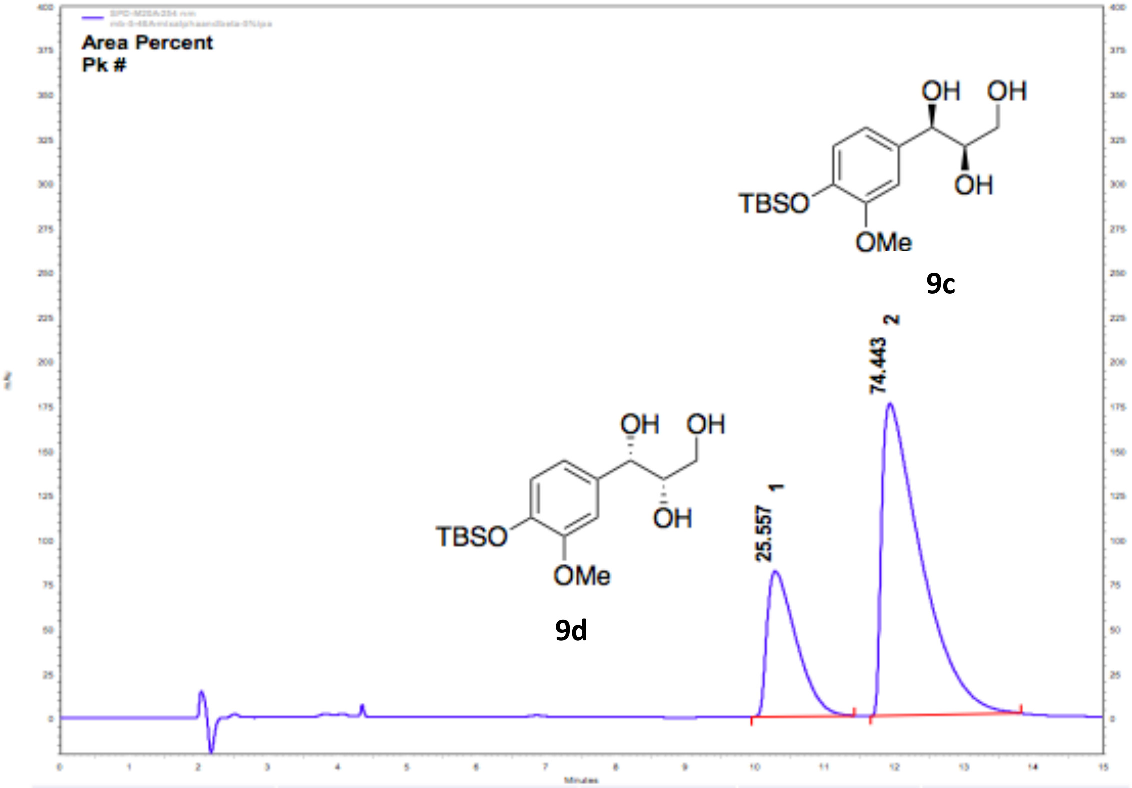

Retention Time	Area	Area %	Height	Height %
10.292	2388953	25.56	81703	31.77
11.940	6958570	74.44	175439	68.23
Totals				
	9347523	100.00	257142	100.00

Column Info: RegisReflect C-Amylose A, 5μm, 250mm*4.6mm
Mobile Phase: 5% IPA/Hexanes
Flow Rate: 1.5 ml/min

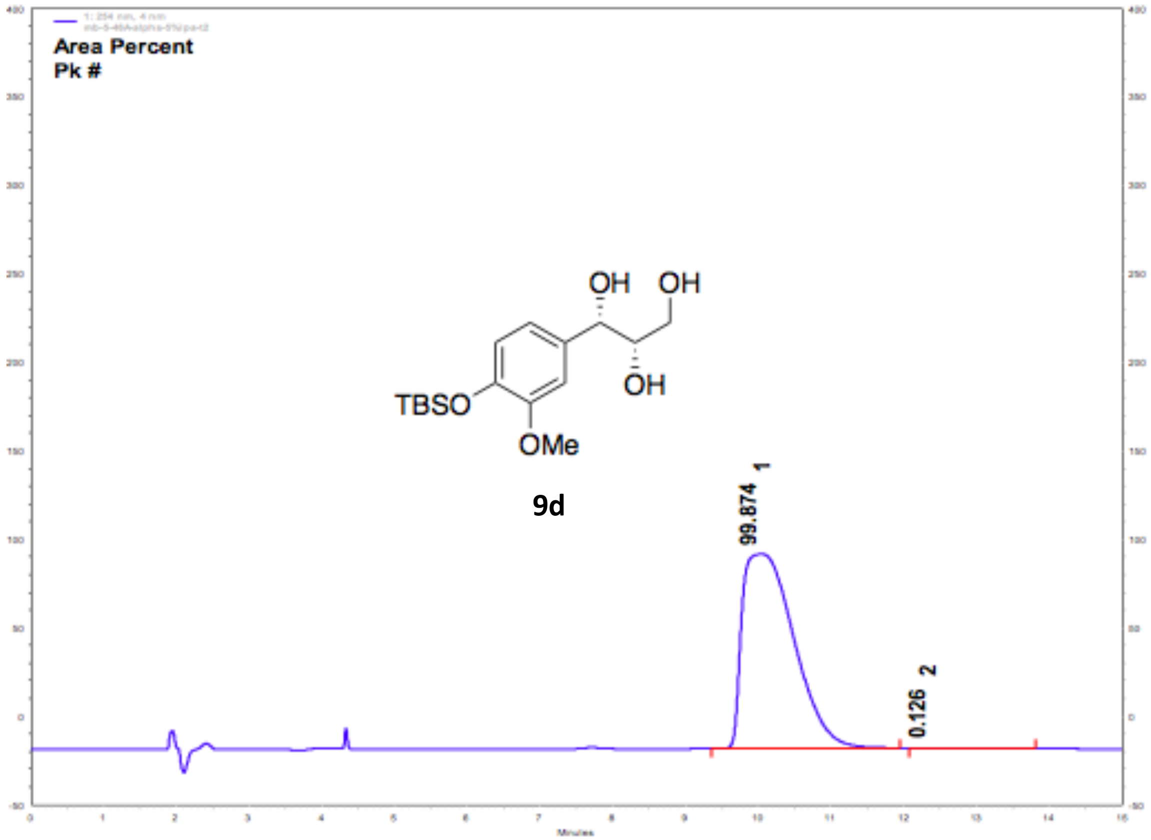

Retention Time	Area	Area %	Height	Height %
10.128	5424868	99.87	109798	99.93
12.392	6841	0.13	80	0.07
Totals				
	5431709	100.00	109878	100.00

Column Info: RegisReflect C-Amylose A, 5μm, 250mm*4.6mm
Mobile Phase: 5% IPA/Hexanes
Flow Rate: 1.5 ml/min

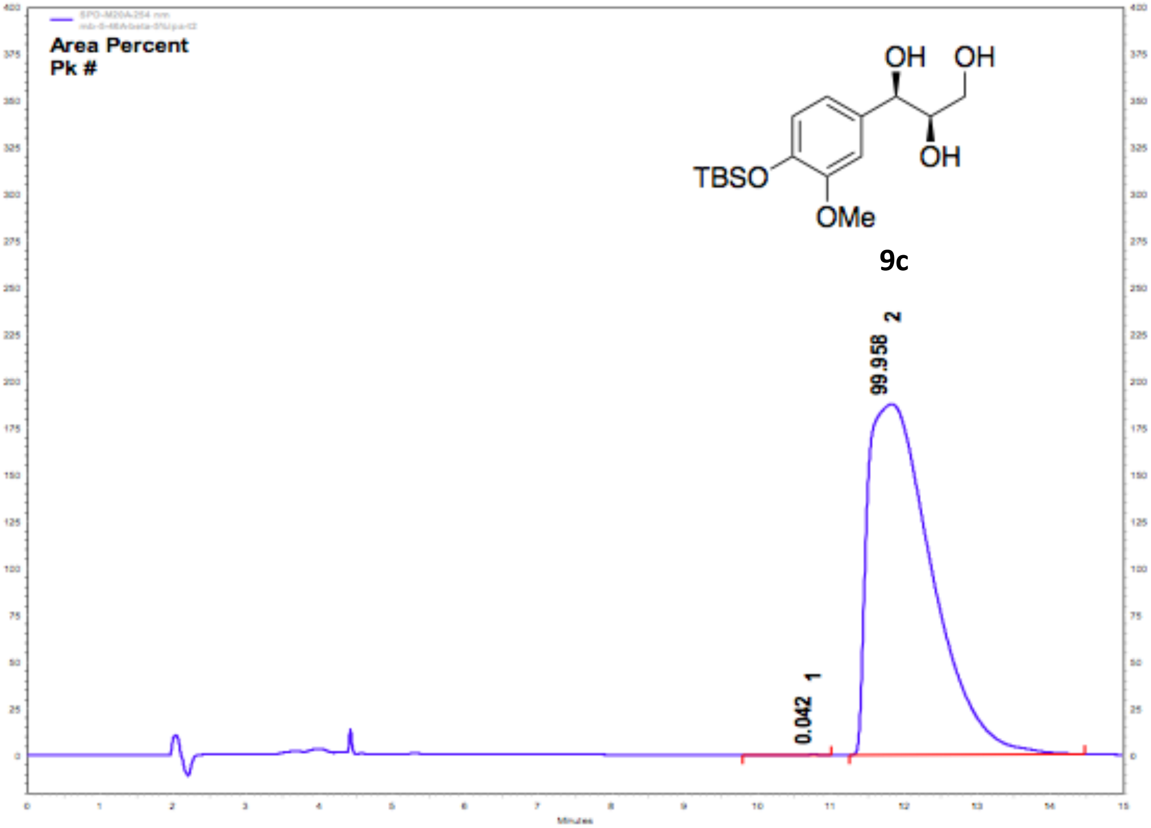

Retention Time	Area	Area %	Height	Height %
10.748	4755	0.04	167	0.09
11.836	11360411	99.96	187259	99.91
Totals				
	11365166	100.00	187426	100.00

C. Asprenol B (**4**) and *ent*-Asprenol B (**4’**)

Column Info: RegisReflect C-Amylose A, 5μm, 250mm*4.6mm
Mobile Phase: 50% EtOH/Hexanes
Flow Rate: 1.5 ml/min

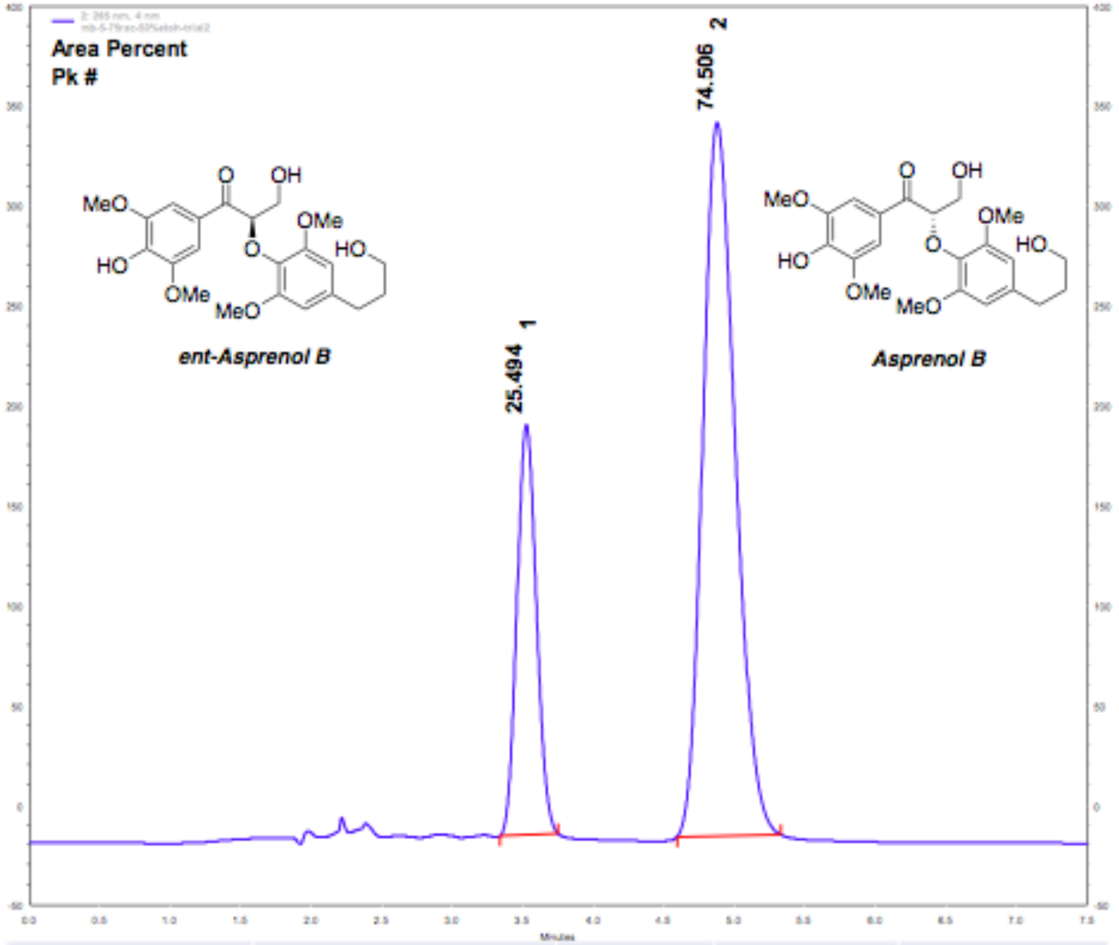

Retention Time	Area	Area %	Height	Height %
3.604	1964607	25.49	205307	36.51
4.956	5741401	74.51	357099	63.49
Totals				
	7706008	100.00	562406	100.00

Column Info: RegisReflect C-Amylose A, 5μm, 250mm*4.6mm
Mobile Phase: 50% EtOH/Hexanes
Flow Rate: 1.5 ml/min

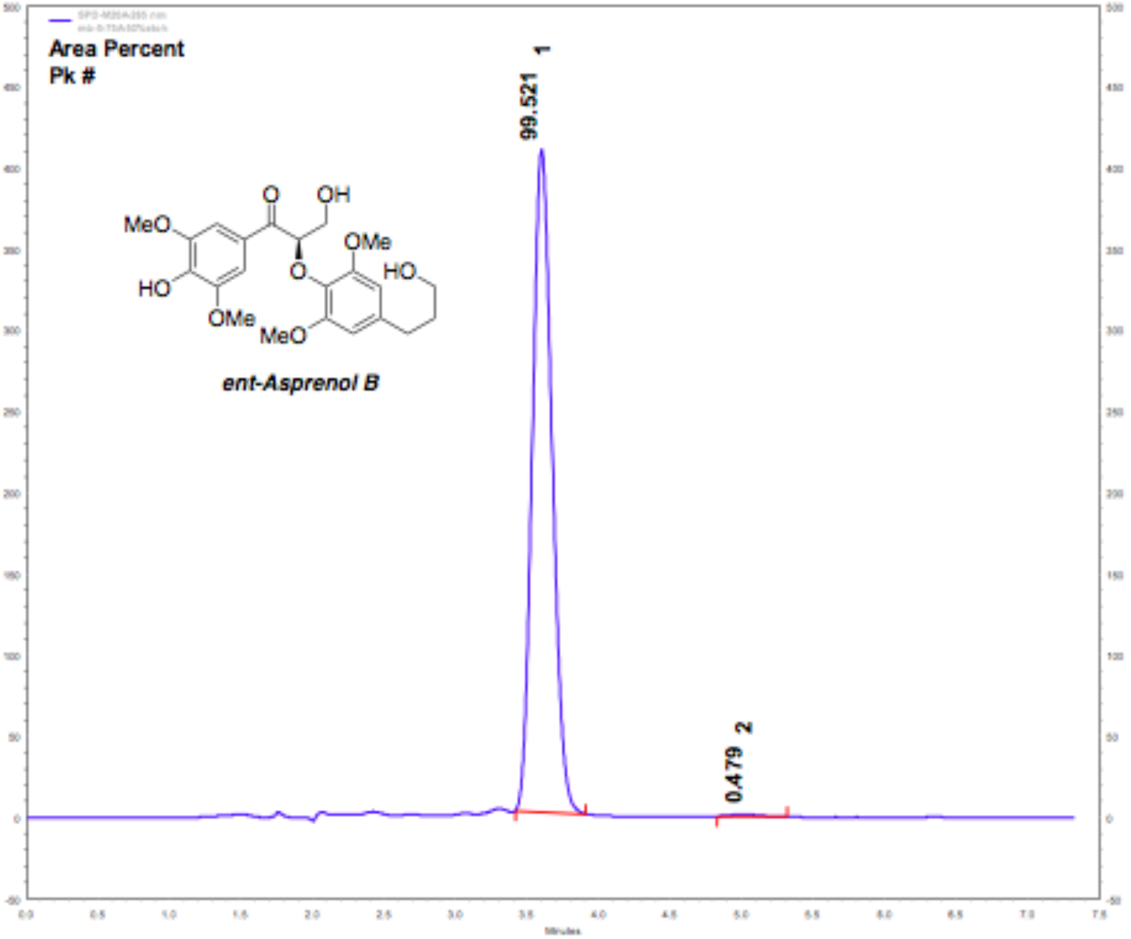

SPD-M20A-265 nm Results				
Retention Time	Area	Area %	Height	Height %
3.600	3930145	99.52	407789	99.67
5.004	18900	0.48	1363	0.33
Totals				
	3949045	100.00	409152	100.00

Column Info: RegisReflect C-Amylose A, 5µm, 250mm*4.6mm
Mobile Phase: 50% EtOH/Hexanes
Flow Rate: 1.5 ml/min

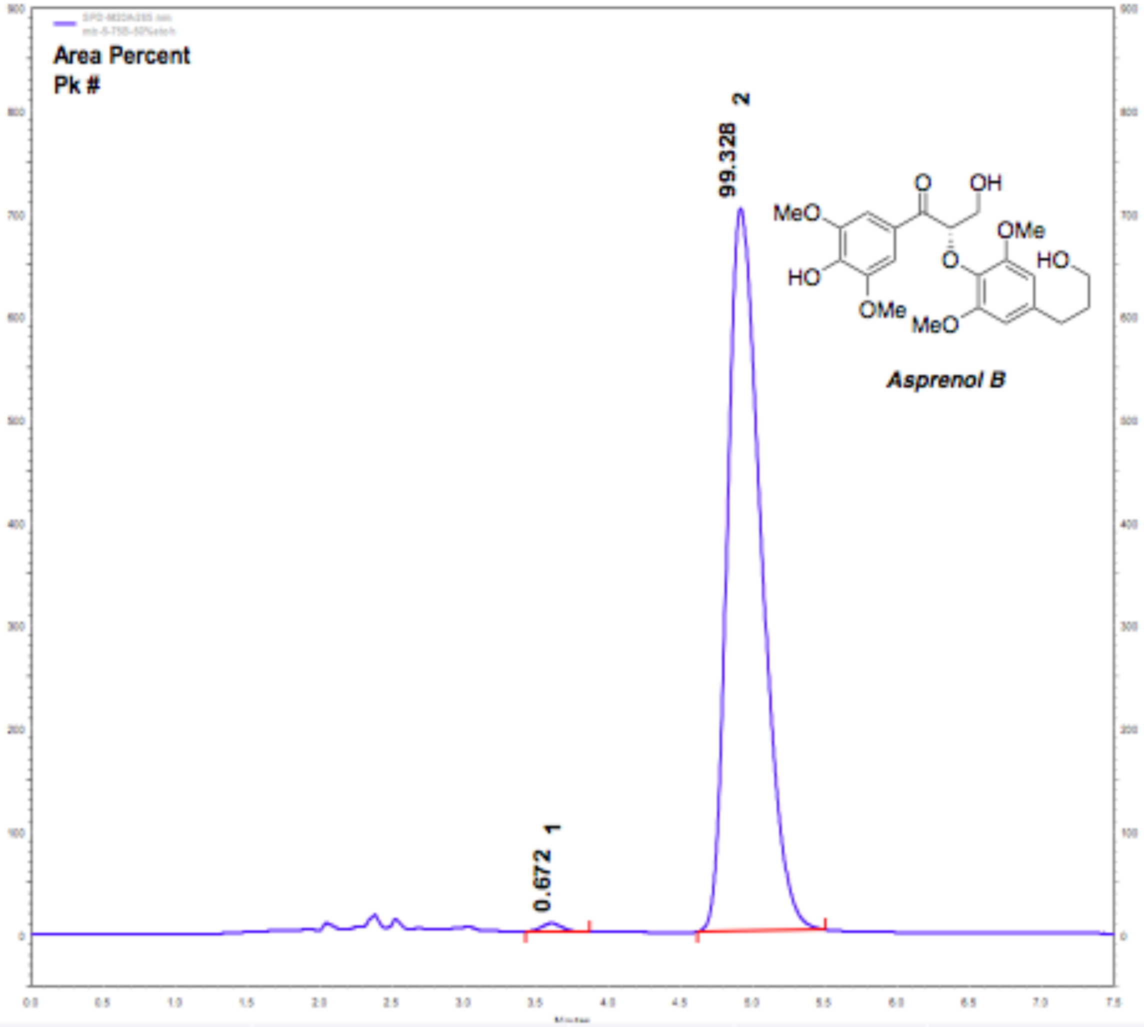

Retention Time	Area	Area %	Height	Height %
3.608	77851	0.67	8135	1.15
4.916	11504434	99.33	701759	98.85
Totals				
	11582285	100.00	709894	100.00

D. (*S*) and (*R*)-Icariol A_1 (**19**)

Column Info: RegisReflect C-Amylose A, 5μm, 250mm*4.6mm
Mobile Phase: 12.5%EtOH
Flow Rate: 1.5 ml/min

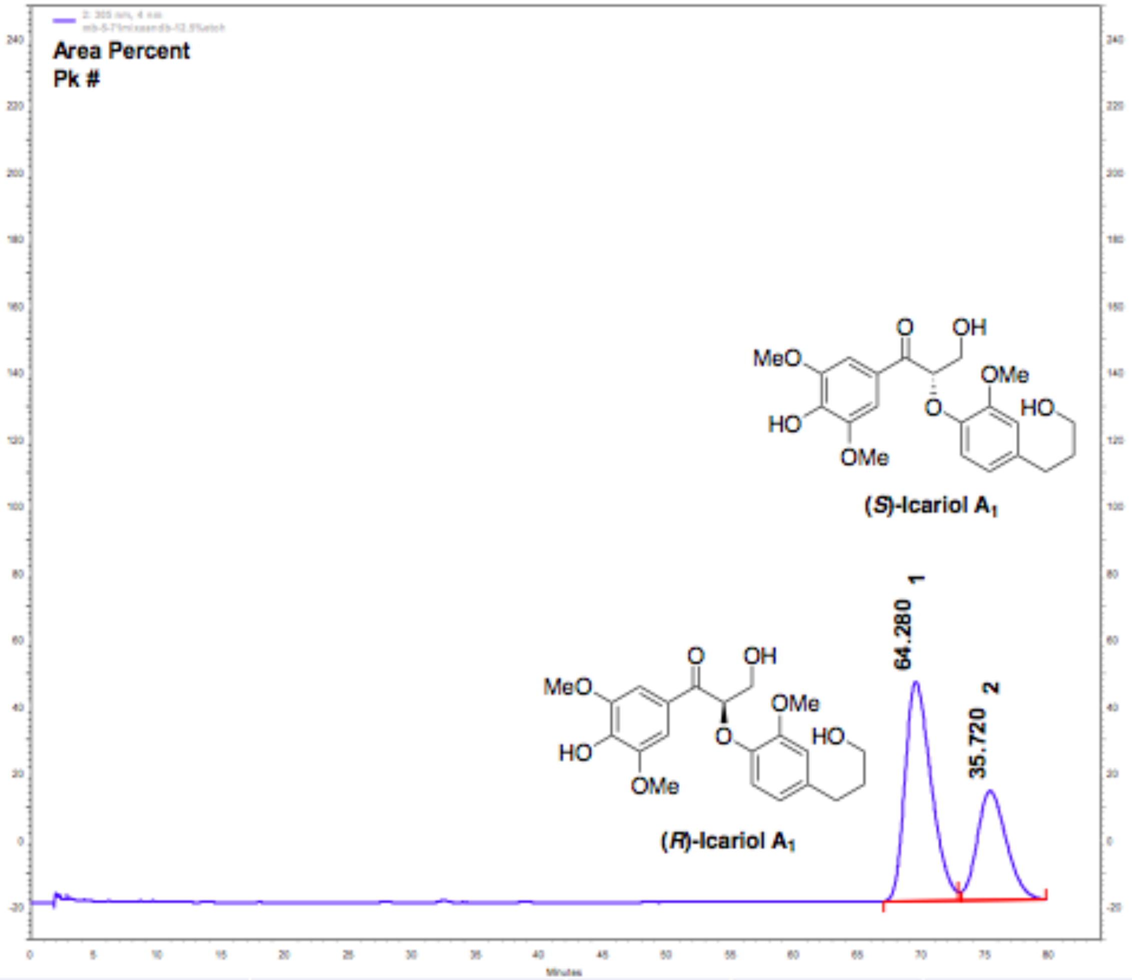

Retention Time	Area	Area %	Height	Height %
69.648	9397133	64.28	65675	66.73
75.488	5221860	35.72	32742	33.27

Column Info: RegisReflect C-Amylose A, 5μm, 250mm*4.6mm
Mobile Phase: 12.5%EtOH
Flow Rate: 1.5 ml/min

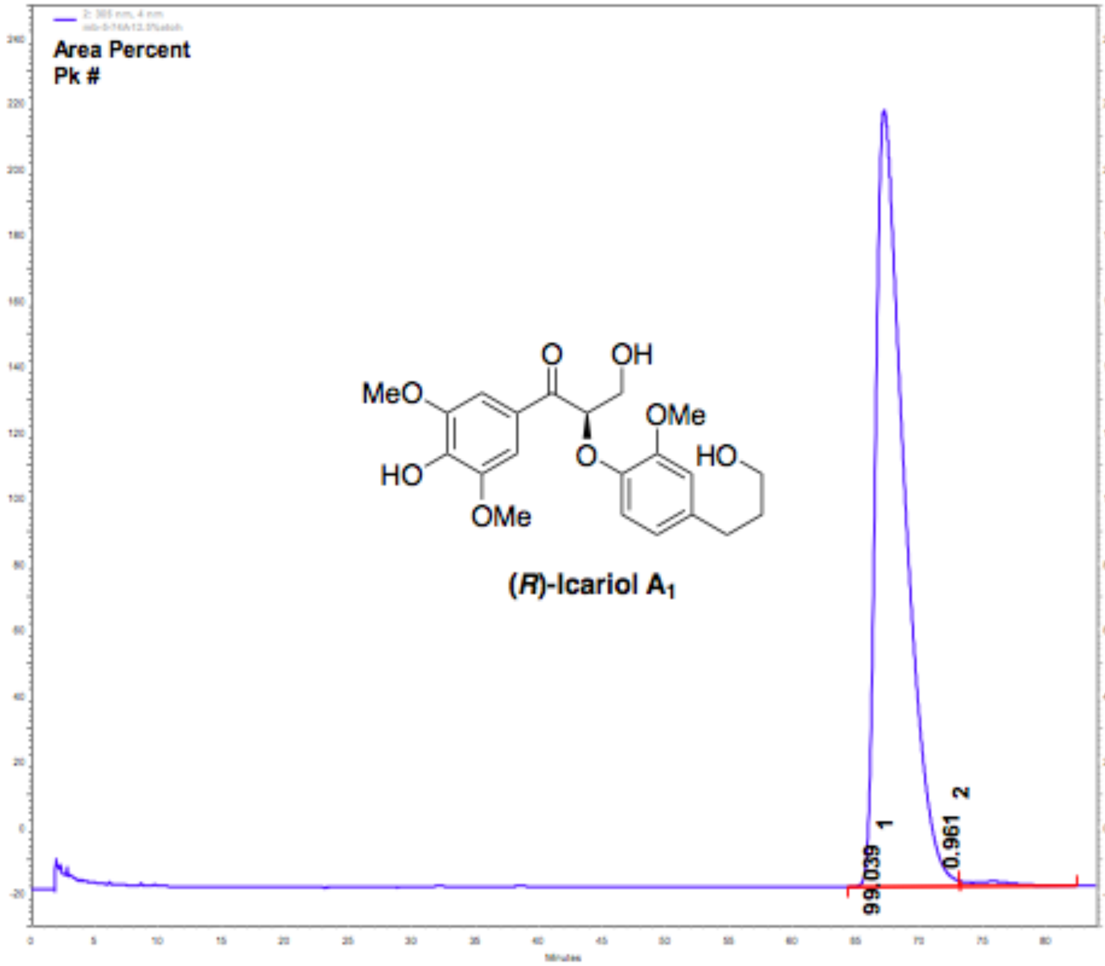

Retention Time	Area	Area %	Height	Height %
67.328	37630876	99.04	236099	99.34
73.296	365110	0.96	1559	0.66
Totals				
	37995986	100.00	237658	100.00

Column Info: RegisReflect C-Amylose A, 5μm, 250mm*4.6mm
Mobile Phase: 12.5%EtOH
Flow Rate: 1.5 ml/min

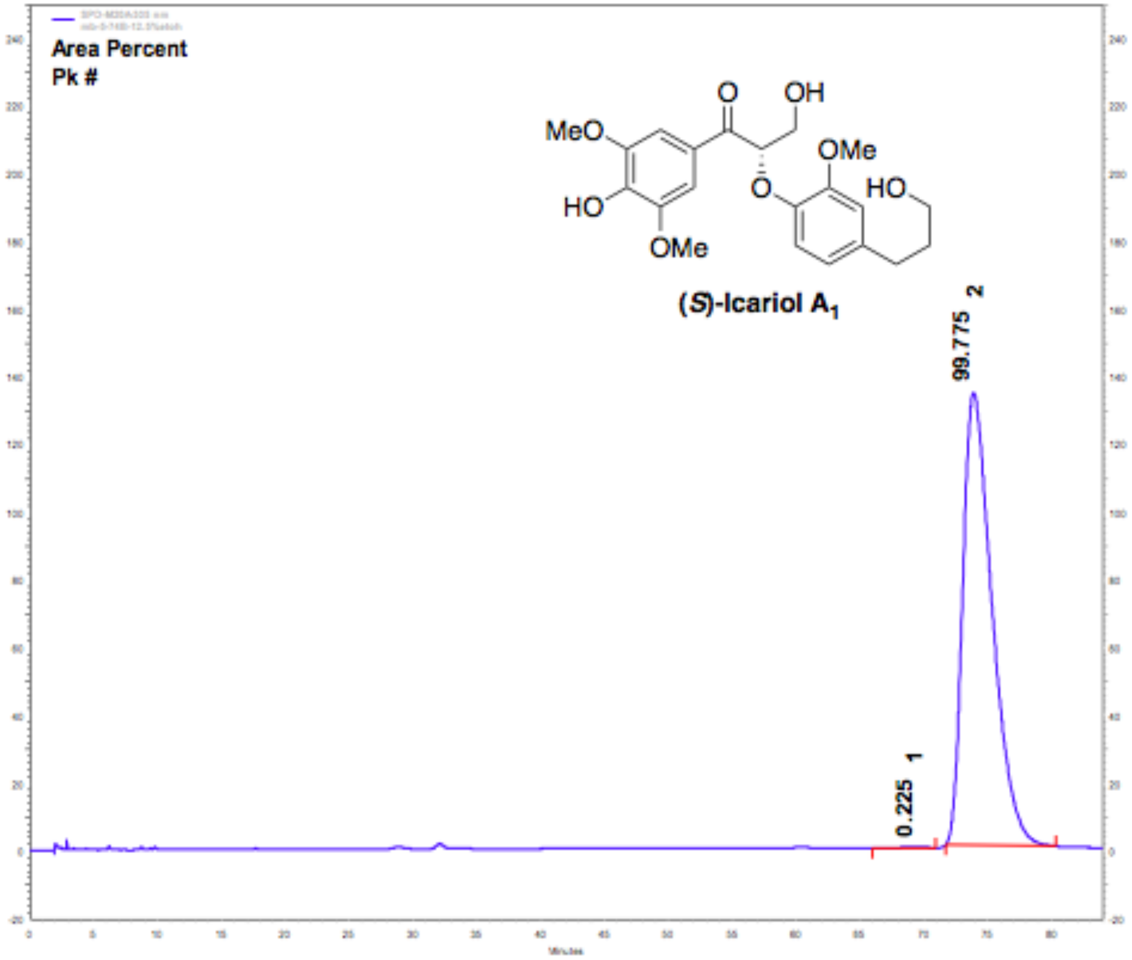

Retention Time	Area	Area %	Height	Height %
69.204	48186	0.23	391	0.29
73.948	21351506	99.77	133571	99.71
Totals				
	21399692	100.00	133962	100.00
